# Communicating with mechanically ventilated patients who are awake. A qualitative study on the experience of critical care nurses in Cyprus during the COVID-19 pandemic

**DOI:** 10.1371/journal.pone.0278195

**Published:** 2022-12-01

**Authors:** Maria Kyranou, Chariklia Cheta, Eliada Pampoulou

**Affiliations:** 1 Department of Nursing, Cyprus University of Technology, Limassol, Cyprus; 2 American Medical Center/American Heart Institute, Strovolos, Cyprus; 3 Department of Rehabilitation Sciences, Cyprus University of Technology, Limassol, Cyprus; Federal University of Acre (UFAC), BRAZIL

## Abstract

**Background:**

Modern protocols for light sedation in combination with the increased number of COVID-19 infected patients hospitalized in Intensive Care Units (ICUs) have increased the number of patients who are mechanically ventilated and awake. Nurses require specific skills to care for this vulnerable group of patients. At the same time, nurses report feeling inadequate and frustrated when they attempt to establish communication with mechanically ventilated, conscious patients.

**Study objectives:**

The purpose of this study was to explore the strategies nurses use when taking care of conscious, intubated patients in the intensive care unit and the barriers they encounter in their effort to communicate.

**Methods:**

For this study, a qualitative design was employed. Data were collected using in-depth semi-structured interviews with 14 intensive care nurses working at ICUs in four different hospitals of Cyprus. The data were analyzed by applying thematic analysis.

**Results:**

We identified several strategies of unaided (movements—lips, hands, legs—facial expressions, gestures, touching) and aided forms of communication (pen and paper, boards, tablets, mobiles) used by nurses to communicate with patients. However, barriers to communication were reported by the participating nurses mainly pertaining to patients and nurses’ characteristics as well as the ICU environment. The health protocols imposed by the pandemic added more obstacles to the communication between nurses and patients mostly related to the use of protective health equipment.

**Conclusions:**

The results of this study point to the difficulties nurses in Cyprus face when trying to communicate with conscious patients during mechanical ventilation. It appears that the lack of nurses’ training and of appropriate equipment to facilitate augmentative and alternative communication leave the complex communication needs of critically ill patients unaddressed. However, further research including patients’ opinions, after they recover, would bring more clarity on this topic. Our study adds evidence to the communication crisis created by the protective health protocols imposed by the pandemic. As such, it highlights the need to educate nurses in augmentative and alternative ways of communication to address communication with mechanically ventilated, conscious patients during their ICU stay.

## Background

Patients hospitalized in an ICU, who are mechanically ventilated, but awake, have complex communication needs, which need to be addressed in a holistic and respectful way to achieve standards consistent with ethical nursing care [1, 2]. This group of patients face temporary communication disability, which, if not addressed appropriately, might leave a negative imprint in their memories [3]. Hospital accreditation standards dictate that communication difficulties acquired as a result of endotracheal intubation during critical illness require provider assessment and accommodation [4].

Modern practices of light sedation in the ICU have increased the number of intubated patients potentially able to communicate, while mechanically ventilated and awake [5, 6]. The use of targeted light sedation is associated with increased [7, 8] and improved survival [9, 10], a reduced rate of tracheostomy placement, shorter time for extubation [5] as well as decreased ICU length of stay [8–11]. Despite the benefits of light sedation during mechanical ventilation, patients experience difficulties in communicating their needs and fears about their survival to health care professionals [12–14]. Additionally, findings from previous studies have consistently associated patients’ communication problems due to endotracheal intubation with distressing emotional experiences, such as anxiety, depression and disturbed memories [3, 15–19]. What is most important is that complications derived from ineffective communication with patients, who are intubated, but awake [20], can be prevented with the use of communication strategies tailored to groups with complex communication needs [21].

At the outset of the COVID-19 pandemic, the rapidly rising number of patients with the disease requiring intensive care created an extraordinary demand on ICU beds across the world. According to a systematic review, 20.3% of patients with COVID-19 require intensive care unit (ICU) admission and almost 75% of patients with COVID-19 admitted to the ICU need mechanical ventilation [22]. We already know from previous studies that nurses in the ICU face serious challenges in their communication with patients who are mechanically ventilated, mainly due to the characteristics of the ICU environment [23, 24], patients’ physical [17, 20, 23–25] and emotional status [17, 20, 23–26] as well professionals’ lack of training in alternative communication strategies [17, 20, 24, 27]. The increase in ICU admission rates in combination with the unpredictable course of the virus, and the lack of effective treatment guidelines [28, 29] posed new barriers in the communication with ICU patients which merit more attention.

Approximately 18 to 54% of mechanically ventilated patients are awake, alert, able to respong to verbal communication [30–32] and could benefit from augmentative and alternative ways of communication [32]. Augmentative and alternative communication (AAC) is a clinical area that addresses the needs of individuals with complex communication disorders, characterized by impairments in speech-language production and/or comprehension, including spoken and written modes of communication [21]. The term augmentative signifies that the person requires supplementary ways to effectively communicate their needs. Alternative methods of communication imply that the person does not possess any oral communication and thus, alternative ways of communication must be utilised [21]. The tools and strategies proposed by AAC have been found effective when used by clinicians to transmit or receive messages to ICU patients [33–36]. However, despite the development of evidence-based tools and techniques to assist communication with awake, mechanically ventilated critically ill patients, these are not yet systematically applied [27, 37]. Furthermore, there is lack of nurses’ education on AAC methods of communication with special patient groups [27, 38].

Communication is a fundamental human right [39] and needs to be ensured for people with disabilities or those with temporary impairments [40]. Importantly, communication is an essential component of the nurse-patient relationship and facilitating patient communication in the ICU has been critical during the COVID-19 pandemic due to their isolation from their families. Most studies on the communication of critical care nurses with intubated patients have been quantitative and have involved evaluating barriers this communication [27, 41–43]. Nevertheless, most of these studies took place in non-Mediterranean countries, where different professional standards apply for critical care nursing practice. Furthermore, nurses’ communication with ICU patients is complex and cannot be understood solely by using quantitative designs. To study the phenomenon in depth, we explored, for the first time in Cyprus, critical care nurses’ communication experiences with mechanically ventilated and conscious patients using a qualitative research methodology. Specifically, this study was aimed at identifying: i. the strategies that nurses use to communicate with patients who are ventilated and conscious; and ii. the barriers encountered in the communication between nurses and patients.

## Theoretical framework

The theoretical framework of this study is informed by the communication theory of Watzlawick et al. (1967) [44], which defines communication as all behaviors in an interactional situation. Specifically, the fourth axiom describes how human beings communicate both analogically and digitally [44]. We found the theory of Watzlawick particularly helpful to understand a complex setting, such as the ICU, where patients experience impaired ability to communicate both verbally and nonverbally. We combined this approach with the scientific field of Augmentative and Alternative Communication (AAC) [21], which encompasses the communication methods used to supplement or replace speech or writing for those with impairments in the production or comprehension of spoken or written language. The Participation Model (Beukelman & Mirenda, 1998) is a systemic process for implementing AAC based on the functional participation requirements of peers [21]. It describes the communication between the sender (e.g., patient) and the receiver (e.g., nurse) within the environment where communication takes place, taking into consideration pre-existing practices as well as communication partners’ (e.g., nurses) attitudes, knowledge and skills [21]. The hypotheses underlying the study design stem from the recognition that patients who are mechanically ventilated and awake in an ICU have complex communication needs [32]. As a result, AAC strategies can be used to address these needs.

Practices of AAC were shown to be useful when used by clinicians and ICU patients with temporary disability to improve communication [30–32]. These practices include both unaided and aided forms of communication as either a permanent addition to a person’s communication or a temporary aid. Unaided forms of AAC do not require an external tool, only some degree of motor control [45] and are paralleled to analogical forms of communication in Watzlawick’s theory. Examples of unaided symbols are manual signs, gestures, and finger spelling, aimed at helping the individual to express thoughts, wants and needs, feelings, and ideas. Aided forms of AAC require some form of external support, which can be categorised as synthesized, hybrid or digitised generating devices [46]. The two theories combined offer an informative framework to study the strategies that nurses use to communicate with patients when they are mechanically ventilated, but awake, as well as the perceived barriers in their communication with patients with complex needs. This framework was used to inform the development of the interview questions and guide the analysis of the data.

## Methods

### Design

For this study, a qualitative descriptive design was used with individual interviews of participants and thus, conforms with the Standards for Reporting Qualitative Research (S1 Checklist report). Our research was conducted during the first wave of the COVID-19 pandemic, when restrictive health protocols were in place in ICU settings. During this period, little was known about the challenges critical care nurses faced in their communication with intubated, conscious patients. For this reason a qualitative descriptive design was employed that has been proven useful for uncovering experiences and perceptions in areas where little is known about the topic under investigation [47]. Phenomenology, grounded theory, and ethnography were not selected since they are based on specific methodological frameworks that emerge from specific disciplinary traditions [47]. Qualitative descriptive studies are not limited by a pre-existing philosophical commitment and “are the design of choice when a straight forward description of a phenomenon is desired” [48].

### Setting and participants

The participants consisted of 14 nurses working in ICUs at four different hospitals of Cyprus, who were purposively identified and selected. Nine out of the 14 nurses worked on units where patients with Covid-19 received care. It was a convenience sample and the participants were recruited from the second researcher after getting permission and informing the ICU staff about the study. The inclusion criteria were: i. working in an ICU, and ii. having worked before with mechanically ventilated and awake patients in an ICU. The exclusion criteria were: i. nurses working in paediatric ICUs or neonatal ICUs, and ii. nurses not willing to participate. Restriction policies in the ICU in combination with the burnout faced by nurses during the pandemic did not allow for the recruitment of more nurses. Nevertheless, the interviews included sufficient richness to allow for analysis of relevant codes and the sample size was considered adequate according to methodological studies assessing saturation in qualitative research [49–51].

### Data collection

#### Interview guide

Qualitative interviewing provides an in-depth exploration of an aspect of life about which the interviewee has substantial experience [52] and was selected to capture the richness of the nurses’ experiences while communicating with mechanically ventilated, conscious patients. To develop the interview guide we followed the interview protocol refinement (IPR) framework [53], including a list of main questions directly related to the research questions as well as follow up questions and probes that could contribute to an inquiry-based conversation. This guide was reviewed by a nursing professor with extensive experience in qualitative research and two critical care nurses with more than 10 years of ICU experience (S1 File).

#### Conducting individual interviews

Data collection took place between September 2020 and May 2021. All interviews were conducted by the second author, some in physical presence (eight participants) and others online (six participants), for those concerned about physically present interviewing due to the pandemic. Prior to the meeting, the second author contacted the participants by telephone and explained the objectives of the study and what their participation would entail. Participants were informed about the methods used to ensure their anonymity and data protection. All the nurses agreed to participate. A consent form was emailed to them and once returned signed, an appointment was made at a day and time that was convenient to the participants. Additionally, consent was granted for the recording of the interview with a digital tape recorder during the physically present meetings and electronically during the online ones. The recordings were deemed necessary to ensure that the researcher was focused on the interview and not on taking notes.

At the beginning of the meeting the interviewer reminded the participants of the aims of the study. The interview guide was employed and follow-up questions were put when collecting further information on a particular topic was deemed as likely to be beneficial. The interviewer (second author) was prepared to depart from the planned interview guide following the interviewee’s interest and knowledge due to the beneficial potential of digressions [54]. The interviews lasted 30 minutes on average.

#### Ethical considerations and investigator preparation

The study was conducted according to the ethical principles of medical research involving human subjects [55] and the law for the safeguarding of personal data processing [56]. The proposed research was reviewed and approved by the National Bioethics Committee of Cyprus (Project-ID: ΕΕΒΚ ΕΠ 2020.01.40) and the Scientific Council of the Research Committee of the State Health Services Organization (email communication: 27.08.2020). Prior to data collection, ethical approval was obtained from the Institutional Review Board of each hospital as well as from each hospital administrator and ICU manager. All participants were informed about the study and the option to withdraw their consent before, during or after the interview. Written informed consent was obtained from all subjects and in addition, in was obtained for the recording of the interviews (physically present and online). The anonymity and confidentiality of the participants was assured, and data were treated as strictly confidential. To protect participants’ privacy and anonymity we herein report nurses’ quotations using pseudonyms.

Two authors of this study are nurses with ICU experience and one has an academic position. The third author is an academic speech and language therapist with experience in qualitative research. The principal investigator took a qualitative research course in graduate school and has conducted multiple studies in ICUs.

### Data analysis

Data analysis involved repeated close reading of the interview data using constant comparative analysis to discern patterns and codes related to the study questions (thematic analysis) [57]. All interviews were transcribed verbatim using non-identifying signifiers (to ensure confidentiality) and all data was stored in a secure location. The transcriptions of the recordings were read several times until the researcher (second author) managed to record the utterances participants shared. Subsequently, the second and third authors focused on the coding of the material. Information from the interviews relating each research question was grouped together. During this process, several codes and subcodes emerged, which were recognized and agreed upon (Table 1).

**Table 1:** Example of the process of analysis from quote to category.

Quote	Codes	Sub-categories	Categories
In order to communicate you have to yell, because you have a mask that is thick with a filter in front of you; a face-silt which is a barrier to communication. The COVID-19 uniform is all over you and it’s covering your ears.	Difficult communication	Protective dressing equipment	COVID-19 communication barriers

The next step of the analysis included the extension of the codes and sub-codes intended to expand the research team’s understanding about the experiences of nurses who care for people who are mechanically ventilated and conscious. The researchers were aware that there would be both singularities as well as universalities among participants’ experiences. For this reason, they paid attention to the codes and sub-codes related to the purpose and specific aims of the study [58]. All authors were actively involved in the process of data analysis: reading the interviews, interpreting the data and summarizing the codes that emerged during the data analysis process [59]. All three authors ensured that there were no coding discrepancies during the coding procedure. Regular meetings occurred between the three authors during which the trustworthiness of the data analysis was ensured.

## Findings

As shown in Table 2, four of the nurses were female and ten were male. The nurses’ ages ranged between 28 and 45 years (mean age = 34.5 years). The years of clinical nursing experience ranged between 2 and 24 years (mean = 11.5 years), and their ICU clinical experience from 1 to 15 years (mean = 5.5 years).

**Table 2:** Demographic characteristics of the nurses.

Nurses (n = 14)	
Sex	
Female	4
Male	10
Years of Nursing Experience	
Mean/median	11.5 / 11.25
Range	2–24
Years of Intensive Care Nursing Experience	
Mean/median	5.5 / 5.5
Range	1–14

The study questions focused on the strategies nurses used and the barriers they faced in the communication with intubated, conscious patients. The findings for these research questions are presented in the following two sections.

### Communication strategies

The first research question focused on the strategies that nurses use to communicate with patients when they are mechanically ventilated, but awake. The findings reveal that they use a variety of strategies to communicate with patients that can be grouped as unaided and aided forms of communication (Table 3). Unaided pertains to those for which the person does not require anything external to his/her body to communicate, whilst the opposite relates to aided forms of communication [60, 61].

**Table 3:** Categories and Sub-categories of communication strategies.

Unaided forms	Aided forms
Eye contact Head nodding Hands or legs movements Touching Close-ended questions	Pen and paper Mobiles Frames Boards with markers Tablets

#### Unaided forms of communication

According to the nurses who were interviewed, unaided forms of communication were employed to respond to patients’ cues, such as eye contact, head nodding, hand or leg movements (e.g., lift their hand upwards or grasping the hand of the nurse), pointing, lip moving, and using gestures. For example, Antria commented “when he is awake, he can communicate and collaborate with others. He can blink, grip someone´s hand, make face and limb movements, let you know when he is in pain, using nodding and gestures.” Participants commented that they encouraged patients to use unaided methods of communication as ventilation acted as a barrier to verbal communication. For instance, Thalia said: “When I ask him if he can hear me, [I instruct him that] he may hold my hand tight or close his eyes [to respond]”.

Other examples of unaided communication used by the participating nurses were closed-ended questions and touching. As Stratos said: “There are many things that might disturb patients hospitalized in an ICU. You may ask them questions, and wait for them to respond, for example: Are you in pain? Does anything bother you? Can you breathe properly?” Additionally, Angelina shared: “…quite often, we use touch as a form of communication, just to reassure them by holding their hands so that they don’t feel alone.”

In addition, nurses often attempted to improve their communication with patients by seeking support from other communication partners, such as relatives and/or other staff. As Achilleas stated: “When patients don’t speak Greek or English, then a relative was sought to help us understand”. Similarly, Angelos said: “I would fetch another nurse and ask him, if he understood what the patient was trying to say”. At other times, when one nurse managed to understand what a patient meant with a specific movement or facial expression (such as pain or thirst), they made sure to communicate it to nurses on the next shift. Angelos said: “Maybe a specific nurse worked more with a specific patient and understands him better, then I ask him what is the best way to communicate with this patient. It’s team-work, you learn to ask for help in an ICU, you can’t work alone”.

As explained in more detail later in this paper, the COVID-19 pandemic has added several restrictions to the effective communication between nurses and patients. However, it seems that nurses are doing their best to utilize unaided strategies for communication with patients. Angelina said: “Now you have these masks and the hats. I think the eyes are really important to make eye contact all the time, because they can’t see if you’re smiling anymore. They can’t see if you’re smiling or what your facial expression is, because you have the masks on. So, it’s important to make eye contact and try to be reassuring with your eyes”.

#### Aided forms of communication

Communication can take place with the support of a variety of aided methods. Participating nurses shared that the most commonly used methods were pen and paper, e-tran frames, mobiles, ipads. Antria said: “We use the pain expression scale. We had a patient with Guillain-Barre, who could not move their face at all, so we slowly started using the letter table. A speech and language therapist came and explained how to use it and the meaning of its colors. But we have rarely used it. In the last few years we have used tablets or pencil and paper; not so much their mobile phones”.

Nurses spoke about paper and boards and how they could be of help in their efforts to communicate with patients. Angelina said: “So, you get them like a piece of paper on a clipboard. And they can write things down. Loizos added: [We had] a board with a marker and there was a glass window in front of him, but we could see him and he could see us. We would write messages for him like “Everything will be ok” or draw a smiley face or write “We will make it, things like that”. This was particularly evident when patients were infected with COVID-19 and distance was warranted. In such cases, nurses were using alternative ways of communication with patients, such as written speech; they were showing their messages to patients through the protective glass.

### Barriers to communication

The second research question focused on the barriers encountered during nurses’ efforts to establish communication. A number of barriers to successful communication were identified and the findings reveal that the COVID-19 pandemic has added additional ones to those previously reported. These are summarized in Table 4 and elaborated upon further in the following section.

**Table 4:** Categories and Sub-categories of communication barriers.

Patient variables	Nurses variables	ICU characteristics	Assistive technology	COVID-19 communication barriers
Physical condition Mental condition Psychological condition Medication(s) Language	Attitudes Lack of knowledge and training on AAC Lack of ICU experience Burn-out	Noise and lights Lack of privacy Foreign environment Time limitations Staff shortages	Lack of equipment Lack of patients’ knowledge in the use of assistive technology	Protective dressing equipment Problems with unaided communication (i.e., glass doors/windows) Limited time in patients’ room due to safety protocols

#### Patients’ variables

The patients’ physical, mental, emotional condition as well as the medication regimen impacted the communication between nurses and patients, who were mechanically ventilated and awake. Focusing on the physical condition, one participant opined that patients might be difficult to communicate with due to chronic medical conditions. Constantinos commented: “We had a patient who was blind and had a hearing impairment. Communicating with him was very difficult, really [difficult], since he could barely hear you and could not see you either”. Muscle strength related to the medical condition was also an issue during communication. As Angelos said: “you could not really tell the numbers, because when intubated they cannot hold the board properly; they don´t have the strength to do so”. Additionally, cognitive impairments could make communication difficult and lead to abandoning all efforts. Antreas said: “If someone has a brain injury, well OK, at the beginning you try, but slowly you realize that a lot of time is required”. Georgia added: “Yes, there are patients who have cognitive impairments, and those cases are generally very difficult… for me, very stressful since you don´t know what the specific person can actually understand”.

Regarding medications Angelina said: “Sometimes if they’re on medication for pain relief, they can be a bit confused and that is an issue, because they want to pull the tube out”. Furthermore, psychological factors, such as depression might deprive patients of any desire to engage in communication. As Agamemnon said: “one moment you see them being reactive, the next moment they might stop talking at all. They may be depressive, it’s different for everyone”. Furthermore, language can act as a barrier, albeit participants seemed to be able to find solutions. Achilleas commented: “In cases when the patients can´t understand Greek or English, a relative may help us”.

#### Nurses’ variables

Some nurses mention that communication depends on nurses’ personal attitudes (such as willingness and compassion). As Thalia said: “all nurses don’t have the same willingness or the same compassion for the patient”. Additionally, nurses identified lack of ICU experience as another barrier; even if they are willing to communicate with patients they need to prioritize their activities, and focus on what they consider most important. As Angellina said: ‘‘sometimes, especially if you’re a new nurse, it’s very difficult to balance the technology and understanding the medications and the pumps, but also the patient’s psychological needs”.

Furthermore, nurses identified work overload, which leads to burn out, as a barrier in the communication. As Thalia said: ‘‘The main obstacle in this situation is the [subsequent] burnout, because you have to support yourself. If I am exhausted, [if] I am dismayed how would I help the other person in front of me, when I myself am tired. I’m just trying to go to do my job, do the basics and leave, it may sound cold and raw, what I’ve just said, but it does happen”.

#### ICU environment

The participants referred to the physical environment of the ICU, which occasionally acted as a barrier to the communication with patients. Specifically regarding noise and lights, Antria commented: “Since the ICU is now an open space, patients are next to each other, there is a lot of noise, fuss, lights… They can´t use their voice to tell you [something], so the quieter it is the easier it is for you to understand, that’s all”. The lack of windows and physical light posed an indirect challenge in the communication since they contributed to altering the patients’ mental status. On this note, Georgia added: ‘‘For patients that stay a long time in place like an ICU, it is important to see windows and sun light, because if they don’t see them, they become very confused and it’s a pity to stress them”.

In addition, nurses mentioned that when patients realized they were in the ICU they experienced distress due to the unfamiliar environment, which made them reluctant to engage in communication. Thalia said: ‘‘First, they open their eyes and they see a foreign place with strangers around them… where they cannot understand what is happening. I definitely think that their first thought is My Lady [Mary], where am I now?”. Another barrier identified related to an open ICU is the lack of privacy, which could lead patients experiencing negative feelings and a lack of motivation for communication. Agamemnon shared: “You might be performing weaning, trying to wake up a patient, and after a while, the patient may see someone else dying, which causes even more terror. So, they might react in a strong way or they might even stop talking; become depressed”.

#### Assistive technology

As mentioned above, communication interaction can take place both in unaided and aided forms. Regarding the aided forms of communication, participants commented that ICUs in Cyprus might not have such a variety of equipment to support the interaction between nurses and patients. Thalia commented: “The facilities do not have the necessary means. I believe that, even though I haven’t looked into it, I’m sure that in foreign countries there are several means of communication. But in Cyprus we know nothing”.

The lack of nurses’ knowledge and training on how to use assistive technology acts as a barrier to communication. As Alexandros said: ‘‘ We have insufficient knowledge, that is, if you were to ask me a question, I’ll tell you I don’t know. I was not taught anything, that is, they gave me a tool and they presented it to me and told me that [for] this patient at this given moment you can work on him [using] these things, and it will have a good result. Namely, this is what we do, act by experience”. Participants also mentioned that patients’ lack of knowledge on how to use assistive technology to communicate can also act as a barrier. Loizos commented: “There are very few patients who can use a tablet since they are a bit old and I think that it’s more difficult for them”.

#### COVID-19 related communication barriers

The findings reveal that the COVID-19 pandemic added communication barriers between nurses and patients mainly due to the compulsory use of protective health equipment. Participants mentioned that health protocols impaired their efforts to communicate, imposing limitations on nurses’ speech output, facial expressions and ability to touch patients as a method of unaided communication. Focusing on speech output Angelos shared how the nurses’ uniforms and the mask prohibited the communication interaction that usually takes place between them and a patient. As he said: “Since we had to wear a very thick mask with a filter, we had to shout so that we could be heard. But having a shield mask on the front stops the sound. Also, we had to wear a full body uniform covering even our ears. So, there were three obstacles, which inhibited the communication between the nurses, between nurses and physicians, and between nurses and patients. Having all of our senses intact helps [nurses], but don’t forget that patients don’t”.

Regarding facial expressions, Agamemnon acknowledged their importance in the communication with patients and shared the barriers that COVID-19 restrictions imposed: “[the pandemic impacted everyday practice] very much, since the patient cannot see your face. This does not allow you to communicate or express your feelings of support, so that [the patient] can understand that you are really there. They can only see your eyes and nothing else. They can’t see your face expressions, and this is bad for a patient who wakes up and what they see is a faceless person”.

Gentle touch is a communication strategy used by nurses, but the findings show that due to COVID-19 restrictions this is no longer practiced. As Loizos shared: “When you know that a patient does not have something like COVID for example, the communication is easier. Meaning that you can go near the patient, talk to him/her from a short distance, touch them. Even with a glove on, this can calm them a bit. Dealing with COVID patients is very different”.

Furthermore, nurses stressed that the protective dressing equipment caused time limitations, which combined with the work overload and staff shortages put communication efforts lower down on the list of their priorities. Agamemnon commented: “It is usually about personal safety measures that exist and the issue of time, because there is currently a lot of work in the ICU. There is less staff, you may have 3–4 patients, and usually you will pay less attention to the patient when he´s awake, since he has fewer needs". The theme of “safety” returned when Thalia said: “In the other ICU, since we now have two, since, well you cannot be inside all the time, for your own safety”. What is worse, some nurses were concerned that the protective equipment might make patients afraid of health care professionals. Georgia mentioned: “We don´t want patients to be scared when they see us. Our appearance is scary, because you see a person standing over your head, wearing a mask, a shield, a scrub cap, gloves, a robe”.

## Discussion

This study is the first to explore the strategies that nurses in Cyprus use to communicate, while taking care of conscious, intubated patients in the Intensive Care Unit, and the barriers encountered in the communication between nurses and patients. Our findings confirm those reported by others, which adds to the generalizability of previous research in Mediterranean countries. The use of a qualitative descriptive design to collect information from the 14 nurse participants in the midst of the pandemic adds to the commitment of nursing research to describe phenomena as they arise, with the use of an appropriate research design [62] involving frontline nurses. Thematic analysis was utilized for the data analysis and the codes were presented according to the two research questions. The findings reveal that participants used a number of strategies to communicate with intubated, conscious patients. These can be summarized as unaided forms of communication (e.g., eye contact, head nodding and touching) and aided forms (e.g., pen and paper, frames and tablets). A number of communication barriers were also identified. These were related to the patients and nurses’ variables, ICU characteristics, the assistive technology and the barriers that the COVID-19 pandemic has imposed. The findings are further elaborated upon in the following paragraphs, with a special emphasis on new strategies and/or barriers associated with the COVID-19 pandemic.

### Communication strategies

Nurses who participated in this study reported using both unaided and aided forms of communication to understand the needs of mechanically ventilated patients who were awake. Unaided forms of communication are natural ways of interacting with others and are used in daily interactions with each other. For this reason, it is not surprising that the unaided forms of communication used in this study align with the findings from other research projects. The various strategies of unaided communication mentioned by nurses in Cyprus were eye contact [23, 27, 63, 64], head nodding [20, 23–25, 65, 66], hands or legs’ movement, pointing [27], lip moving [20, 23–25, 65, 66] and gestures [17, 25, 64, 65]. In fact, eye contact between nurses and patients was found to be one of the most common unaided methods utilized, which is not surprising as this is a natural communication method that people use during face-to-face communication. Similarly, Karlsson et al. (2012), Tingsvik et al. (2013), Happ et al. (2011) and Magnus et al. (2006) reported head nodding as a communication strategy [23, 27, 63, 64]. Again, this is a natural way of communication since people usually move their head to express consent or rejection of something (e.g., yes/no reply).

Consistent with the existing literature [20, 23–25, 65, 66], the nurses of this study engaged in lip reading as another communication strategy, with patients who were ventilated and conscious. However, what is intriguing is that they reported that lip reading was used as a communication strategy by patients to understand messages conveyed by nurses. This is a rather unusual finding that needs to be interpreted in light of the health protocols imposed by the COVID-19 pandemic. When isolation is recommended for infection prevention full glass walls are instituted, which means that communication is even more challenging. In such cases, there is distance and physical barriers between nurses and patients. As a result, patients cannot hear what the nurses are saying and use lip reading as an alternative method. What is worse, the COVID-19 health protocols introduced face masks in ICUs, which obstructed patients from reading nurses’ lips. Using transparent masks was not reported by the nurses in this study and we cannot be certain whether they were not readily available or just not popular amongst them. Ideally, nurses should wear transparent masks to allow patients to see their lips and facilitate the communication with conscious patients during mechanical ventilation.

Close-ended questions were also reported by nurses to be of use when communicating with patients. These types of questions require a yes/no response, which is easily conveyed by the patient with head nodding or the eyes and has been reported as a communication strategy by others [23, 24, 27, 65, 67]. Pointing was another strategy mentioned by our participants, which was also reported by Happ et al. (2011) [27]. Gestures [17, 25, 64, 65] were mentioned as an unaided communication strategy employed by nurses in their effort to communicate with conscious, mechanically ventilated patients similar to touching as was touching [24, 27, 64, 66].

Nurses often attempt to improve their communication interaction with patients by seeking support from other communication partners, such as relatives and/or other staff. In the studies by Bergbom et al. (1993), Karlsson et al. (2015) and Rodriquez et al. (2015) nurses sought help from patients’ relatives [25, 26, 66], whilst those in our study did not report similar practices. This contrasting finding can be attributed to the restrictive visitation policies in the ICU during the pandemic, which meant patients’ family members were unavailable to staff. To address this shortcoming nurses in our study resorted to intense collaboration amongst themselves. As reported by participants in our study, when one nurse managed to understand what a patient meant with a specific movement or facial expression (such as pain or thirst), they made sure to communicate it to others on the next shift. This is in agreement with findings by Rodriguez et al. (2015), who found that when patients could not clearly communicate their needs, nurses had to “figure out the clues” and then, pass those on “nurse to nurse, shift to shift [25]”. These findings point to the importance of good communication between ICU staff and the opportunity created by the pandemic to bring back attention to standard practices that ensure safe nursing care.

Furthermore, nurses employed aided methods to facilitate their communication with patients who were mechanically ventilated, and awake. Similar to others, nurses in this study stated that they often used pen and paper, markers and frames [17, 24, 25, 27, 65, 66]. These are considered low-tech products and are widely used in ICU settings, because they are relatively inexpensive. In addition, communicating via pen and paper is familiar to most people. Naturally, easy ways of communication are selected first since AAC strategies require training. However, low-tech strategies themselves also require training, such as the e-tran frame, which is a piece of transparent plastic (Perspex) on which different types of symbols (e.g., photographs, pictures, traditional orthography) are attached. Patients need good control of their eyes and arms in order to point to the symbols and convey their answers to their communication partners.

As reported by the nurses in this study, whilst training to use the e-tran frame is imperative, it is not included in their education in Cyprus. This is probably the reason why it was not frequently reported by nurses in our study as a communication strategy. Nevertheless, e-tran frames were used by nurses in other studies [23–25, 65–67], potentially reflecting differences in nursing education across countries. Lack of exposure and training to AAC methods can be facilitated by bringing together communication partners (i.e. nurses) with speech and language therapists/ AAC experts, who can provide appropriate support to improve communication with ventilated and awake ICU patients. Our intention is that the findings generated by this report will be used to inform educators at the undergraduate and post-graduate level regarding the content of courses aimed improving communication with patients with complex communication needs.

### Barriers to communication

Nurses in our study identified several barriers in their efforts to communicate with patients who were mechanically ventilated and awake. These are presented according to the Participation Model, which guides the assessment of ACC practices and was used as a framework in this study. According to this model, *access barriers* are factors that are internal to the person using AAC, such as attitude, capabilities and constraints (e.g., motor, literacy, cognitive-linguistic, and sensory-perceptual skills) [22]. In our study, these were mainly related to patients’ medical condition, medication regimen as well as language. Cognitive dysfunction was identified as an important barrier similar to the findings of other research [23, 27, 34, 65]. In addition, we found that muscle weakness made it extremely difficult to even use a pen to write on paper or hold a board to identify appropriate symbols. Similar findings have been reported by Holm et al. (2017), who elicited that patients’ level of fatigue, muscle strength, consciousness, cognitive ability and participation in care were of great importance for communication [65]. Moreover, according to the participating nurses, the patients’ psychological state acted as a barrier to the communication. This is consistent with findings by others, who reported that many patients refused to engage in any communication due to feelings of frustration from not being able to speak [25, 26]. Similarly, patients’ feelings of anger, anxiety and depression have been reported as communication barriers between nurses and patients in the ICU [23].

Furthermore, according to the Participation Model, *opportunity barriers* are those imposed on the person using AAC by outside sources, such as policies and practices that are set in place as well as the attitudes, knowledge and skills of the family, and therapists that can deter communication. Nurses in our study acknowledged their own attitudes (such as willingness and compassion) along with their lack of knowledge and training on how to use assistive technology as important opportunity barriers to communication with mechanically ventilated patients who are awake. Our findings are in line with those of others, who found that nurses’ attitudes towards sedation (lack of patience) was a defining factor that affected communication with patients [63]. Similar to our findings, Mortensen et al. (2019) reported that nurses in their study recognized their lack of knowledge of ACC practices and expressed their willingness to improve it [20].

Additionally, the ICU environment imposed serious challenges in the quality of the communication between nurses and awake, mechanically ventilated patients. Nurses in our study agreed with others [68, 69] that constant noise and lack of privacy make nurses and patients’ orientation towards communication extremely difficult. Furthermore, we found that assistive technology for aided forms of communication was not readily available in ICUs in Cyprus, thus imposing further limitations to patients when trying to express their needs. To make matters worse, ICU nurses reported that their feelings of exhaustion due to work overload contributed to their unwillingness to use new methods in communication, which in turn, led to communication breakdowns.

Our findings are in agreement with the Participation Model, which holds that skills, knowledge and attitudes are important features of the communication partner when facilitating AAC [21]. For instance, if the person does not have a good attitude towards AAC forms of communication or they lack relevant experience, then communication breakdowns will occur. Importantly, previous work has shown significant improvements in nurse-patient communication in the ICU with training and the use of AAC [70, 71]. This provides evidence for the efficacy of implementing structured approaches involving AAC use for the improvement of communication with mechanically ventilated patients who are awake in an ICU.

#### COVID-19 health protocols

The COVID-19 restrictions added communication barriers between nurses and patients mainly due to the compulsory use of protective health equipment, which greatly diminished the use of unaided communication strategies. Participants mentioned that health protocols imposed limitations on the way they talked (speech output), used facial expressions and touching to communicate with patients. Nurses in our study recognized the importance of all three strategies for successful communication with patients and elaborated upon the difficulties that the pandemic was imposing on their practice. This is one of the first studies to portray the challenges that the pandemic has brought in the communication between nurses and patients who are ventilated and awake. What is worse, nurses reported that without the unaided methods of communication as basic tools (speech, face, touch), communication in the ICU could fail, resulting in feelings of distress to both patients and nurses.

Furthermore, the findings show that the COVID-19 pandemic has exacerbated the problem of limited time devoted to communication with mechanically ventilated patients who are awake. When patients are in a life threatening situation, nurses will tend to focus primarily on addressing, rather than attempting to communicate effectively with them. Whilst reduced staffing is a well-known problem in the ICU associated with reduced patient outcomes [72], during the pandemic it reached unprecedented levels [73]. Staffing shortages combined with isolation precautions due to safety health protocols further threatened communication breakdowns with patients, which could have contributed to medical errors. What the consequences, clinical and/or emotional outcomes of these communication impairments might be for critical care patients is still too early to tell. Patients might take some time to be able to talk about their experience in the ICU during the pandemic, which, in turn, makes it hard to understand how this experience affected their quality of life during and after their stay. Finally, the impact of communication breakdowns with mechanically ventilated, conscious patients on nurses’ feelings would be worth exploring in the future.

### Strengths and limitations

The in-depth information that was collected during the interviews led to interesting findings regarding the experiences of nurses when trying to communicate with ventilated, awake patients during a pandemic. In terms of rigor in qualitative research, this study conforms to the Standards for Reporting Qualitative Research (S1 Checklist report). Additionally, during the data analysis all transcripts were analysed in their original language, which adds to the richness of the findings. Furthermore, the composition of our research team included an academic, a clinical nurse as well as a speech and language therapist, which allowed for an interdisciplinary approach to investigating patients’ complex communication needs involving different perspectives.

The sample was relatively homogeneous, with Cypriot-speaking nurses from three urban areas. Participants worked in various types of ICUs in Cyprus (i.e., public versus private, open versus closed), which adds to the variety of experiences that nurses might have had and covered a wide range professional experience, with both short and long clinical placements. The interviewer’s experience caring for critically ill patients facilitated a trustful relationship with the participants, but could also be seen as a limitation, if her pre-understanding of the context inhibited her being open to unexpected revelations. The interviewer was aware of this risk and strived to be open minded during the interviews, posed clarifying questions, and avoided drawing conclusions without ensuring that she had clearly captured the participants’ responses. Finally, an important strength of this study and its primary contribution is the fact that data was collected during the period of the COVID-19 pandemic. This is one of the first studies in the Mediterranean, and the first in Cyprus, exploring communication barriers caused by the COVID-19 pandemic. As such, its findings can contribute to the exploration of solutions for the consequences that COVID-19 inflicted on both nurses and patients regarding their communication.

The main limitation of the study concerns the size of the sample. Even though there is no fixed number for the ideal sample size in qualitative studies, the authors believe that a larger sample size would have produced a deeper exploration of the topic. Despite the universality of experiences amongst people, there might also be singularities from one person to another [74], which a larger sample would allow us to explore. However, data was collected during the first wave of COVID-19 pandemic, when nurses were exhausted and time limitations were an important consideration. Nevertheless, the data obtained has identified the barriers that COVID-19 has imposed on communication with patients, who are mechanically ventilated but conscious and the ways in which nurses have had to adapt to facilitate communication during this challenging period.

### Recommendations for clinical practice

Patients who become suddenly voiceless due to intubation regard high-tech AAC devices as a useful, reliable, and acceptable alternative communication choice in the ICU [75]. Thus, it is imperative to find ways to implement evidence-based practice in everyday clinical practice and respond to consumer demands for improved services. Every implementation effort of evidence based practice needs to combine patient values with the best research evidence and clinicians’ expertise [76]. The present study was focused on clinicians’ expertise in the ICU and aimed to provide an in-depth view on the strategies employed by nurses in Cyprus working in critical care aimed at communicating with patients who were mechanically ventilated, but awake. Whilst the findings have revealed the active role that nurses take in assisting patients who are intubated and conscious to communicate, their remains the need for their receiving formal education on aided and unaided methods of communication. This research could inform undergraduate nursing programmes to equip future professionals with the skills required to efficiently communicate with mechanically ventilated patients who are awake. Also, the findings could function as a baseline measurement for assessing improvement to these practices after the implementation of educational programs with clinicians.

Patients who are admitted to the intensive care unit and undergo mechanical ventilation can only express themselves nonverbally or via communication tools. For this reason, close collaboration with speech and language therapists can be achieved, if they are invited in the ICU for the assessment and management of a patient with complex communication needs. This would set the stage for interdisciplinary collaboration during which speech and language therapists could share with nurses (and caregivers) their expertise on unaided methods of communication (such as visible masks) and aided tools. Currently, similar interventions are being developed that employ a multi-component bundle [77]. It remains to be seen how these interventions will be received by nurses and patients. Through this study our aim has been to improve communication between nurses and mechanically ventilated, conscious patients, in Cyprus, and elsewhere.

## Conclusions

Fear of the unknown and lack of contact with caregivers can make patients who are awake, while mechanically ventilated, experience feelings of isolation and anxiety. It is critical to use all available methods to address obstacles to communication in these circumstances. Nurses play a crucial role as facilitators of communication and our results attest to their determination to select various strategies to sustain it with patients even during extremely difficult circumstances, such as the COVID-19 pandemic. Communication with the patient lies at the heart of nursing care and as noted by many participants, making eye contact became even more important during the pandemic, because smiling or facial expressions were not available with the masks on.

The composition of our research team provides an inter-professional example of multidisciplinary collaboration, which makes the exploration of patients’ complex communication more complete. Hopefully, our study will contribute to future research that will include (AAC) methods with mechanically ventilated patients who are conscious, but unable to speak due to the presence of an endotracheal tube. Particularly for Cyprus and other Mediterranean countries, where no previous research is available, our study aspires to foster collaboration between multinational research teams and national nursing bodies to improve education and guidelines for the communication between nurses and conscious, mechanically ventilated patients.

## Supporting information

S1 File Interview guide. (DOCX)

S1 Checklist SRQR checklist. (DOCX)

S1 Data (DOCX)
