# Supplementary material for: Communicating with mechanically ventilated patients who are awake. A qualitative study on the experience of critical care nurses in Cyprus during the COVID-19 pandemic
Source: PLoS One. 2022 Dec 1;17(12):e0278195. doi: 10.1371/journal.pone.0278195 (PMC9714938; doi:10.1371/journal.pone.0278195)
Supplement: S1 File — (DOCX) [file pone.0278195.s001.docx]

**Interview Guide**

Demographic information

1. How old are you?

2. How long have you been working as a nurse?

3. How long have you been working in the ICU?

4. What is the level of your education (e.g. Bachelor, Masters, PhD)?

Main questions

1. How is it when you work with people who can't talk because they are intubated but awake?

2. Can you please describe your experience when you attempt to communicate with a patient who is intubated but he/she is conscious? Can you please describe a specific experience you had at some point?

3. While communicating with people who are intubated and awake can you identify their problems and their needs? If so, how? Can you please give me an example?

4. How do you think people who are intubated and awake feel when they want to communicate but are not able to do so?

5. From your experience, what do you think patients who are intubated but awake need or are trying to communicate to you when you care for them?

6. What barriers do you encounter in your attempt to communicate with an intubated patient who is awake?

7. How do you feel about that?

8. What strategies/ ways do you use to communicate with this group of patients?

9. In which ways do you manage to communicate with patients who are ventilated and awake?

10. In the ICU you work, do you have any complementary means for communication with intubated patients, such as pen and paper, communication boards – boards where you chose a button and a message appears, etc?

a. If yes, can you please tell me which ones?

b. Do you use them?

c. What do you think about these means of communication?

d. Do you experience any difficulties in using these means of communication?

11. Have you previously received any training in communication strategies using different aided forms of communication for patients who are intubated but awake?

a. If yes, in what context? For instance, during your bachelor studies, continuous education in the ICU, conferences etc.?

b. No

12. Would you wish to receive training in various aided forms of communication for patients who are intubated but awake?

a. Yes and why?

If yes, what is your preferred training method? For example, by the bedside, as a workshop, online courses, at conferences, during focus groups with patients who were hospitalized in the ICU you work etc.

b. No

13. Is there anything else you wish to add about your experience communicating with patients who are intubated and awake?

Thank you for your participation in this research project.

We greatly appreciate your time.
