# Supplementary material for: Communicating with mechanically ventilated patients who are awake. A qualitative study on the experience of critical care nurses in Cyprus during the COVID-19 pandemic
Source: PLoS One. 2022 Dec 1;17(12):e0278195. doi: 10.1371/journal.pone.0278195 (PMC9714938; doi:10.1371/journal.pone.0278195)
Supplement: S1 Data — (DOCX) [file pone.0278195.s003.docx]

**INTERVIEWS**

**(all names appearing in this document are pseudonyms)**

**Νο 1**

***R: researcher***

***A: Antreas***

***R: Antrea, να έρθω έτσι λίγο πιο κοντά (είμουν λίγο μακριά από το τραπέζι), όπως σου εμίλησα και πριν κάνω μια έρευνα που αφορά τους διασωληνωμένους ξύπνιους ασθενείς και θέλω να μιλήσουμε έτσι λίο να μου πεις τις εμπειρίες σου όσο αφορά τούτο το θέμα. Να σου κάμω μερικές ερωτήσεις, αρχικά δημογραφικές για να μπουμε ετσι λιο, να δουμε τα δικα σου τα θέματα στην αρχή***

A: OK

***R: … και ύστερα να σου κάμω τις ερωτήσεις, λοιπόν, πόσο χρονών είσαι;***

A: 29

***R: ..29,πόση εμπειρία έχεις ως νοσηλευτής;***

A: 3 χρόνια

***R: Στην ΜΕΘ πόση εμπειρία έχεις;***

A: 2

***R:.. 2 χρόνια,μπράβο α,αρκετά, εμένα τα κριτήρια μου είναι από ένα και πάνω. Λοιπόν,από όσο ξέρω εκαμες και μεταπτυχιακό ενεν;***

A: Nαι

***R: Σε τι αφορά to μεταπτυχιακό σου αν επιτρέπετε;***

A: Eπείγουσα νοσηλευτική και μονάδες ενταντικής θεραπείας.

***R: πολύ ωραία…,οκ.., που το εκαμες;***

A: Στην ελλάδα

***R: Που συγκεκριμένα;***

A: Στην Πάτρα και το μεταπτυχιακό στην Αθήνα …..και εργάστηκα ένα χρόνο στην Πάτρα, στα επίγοντα

***R: οκ.. ντάξει***

A: … και δυο χρόνια δαμέ στο American

***R: .. Στην εντατική***

A: ..ναι

***R: λοιπόν,έτσι μια γενική ερώτηση για να ανοίξουμε λίο το κλίμα, πως είναι όταν εργάζεσαι με διασωληνωμένα,εεε, άτομα τα οποία είναι ξύπνια όμως, μπορείς να σκεφτείς ασπούμε, μπόρεις να φέρεις στην μνήμη σου έτσι ένα περιστατικό, να σκεφτείς, ο οποίος έχει ενας ασθενής τον σωλήνα, οι τραχειοστομημένος,διασωληνωμένος***

A: ..ναι ναι

***R: .. αλλα εν ξύπνιος, έτσι να μου αρκέψεις, έτσι που το τίποτε να μου πείς τι νιώθεις ή πως είναι όταν εργάζεσε***

A: Κόιτα ενεν κάτι εύκολο

***R: ..ωραία***

A: τζαι εν κάτι δύσκολο, τζαι ψυχολογικά δύσκολο

***R: .. για ποιο λόγο δηλαδη;***

A: Γιατί εχεις να αντιμετωπίσεις ένα άρρωστο ο οποίος έχει,ουσιαστικά έχει ένα σωλήνα μέσα στο στόμα του τον οποίο δεν ανέχεται, στην πλειοψηφία, θέλει να τον φκάλει τζαι πρέπει να τον υποστηρίξεις και ψυχολογικά αλλα τζαι να του διασφαλίσεις το safety του, τον να μεν φκάλει τον σωλήνα και να μεν μπορεί μετά να αναπνεύσει

***R: μάλιστα, οκ.., εεεε,με πιο τρόπο κατφέρνεις να επικοινωνήσεις με αυτά τα άτομα, γενικά, δηλαδή πως καταφέρνεις να επικοινωνήσεις που την στιγμή που δεν μπορούν να μιλήσουν;***

A: Συνήθως μιλώ τους ασπόυμε τζαι με νοήματα τζίνοι καταλαμβαίνω αν με καταλαμβαίνουν, ε πολλές φορες δεν μπορείς όμως να συνενοηθείς όταν ο ασθενής είναι ακόμα υπο την επείρια κάποιων εε αναισθητικών

***R: .. μχχ***

A: .. δηλαδή μπορεί να παίρνει ασπούμε ultiva και να είναι διασωληνωμένος, ενοώ και να είναι μισοξύπνιος ασπούμε,οπότε τζιαμέ δεν έχεις πλήρη επικοινωνία,εεε, συνήθως γίνεται με νοήματα, λαλείς του να σου σφύξει το χέρι να δεις αν σε καταλαμβαίνει και που τζιαμε τζαι τζι αν υπάρχει συνενόηση

***R: .. μάλιστα, ενας τρόπος τωρά που μου είπες είναι ασπούμε με νοήματα και το σφύξιμο το χέρι,υπάρχει κάποιος άλλος τρόπος, κάποιος που μπορείς να σκεφτείς, ο οποίος καταφερνεις να επικοινωνήσεις με τούτο τον ασθενή,αμαν σκεφτείς έτσι στην μνήμη σου το περιστατικό, τι άλλο κάμνεις για να δεις, πως επικοινωνάς με τούτο τον ασθενή;.. δηλαδή νοήματα, τι νοήματα; με το χέρι του, με τα μάτια του;***

A: .. ένα του πεις κλείσμου τα μάτια σου,εεε ή …. Να σου κουνήσει ξερω γώ τα πόδια

***R: χμμ δηλάδή εσύ διας του,λαλεις του μιαν ετώτηση για παράδειγμα..***

A: .. αν με ακούει,εξηγώ του ότι έκαμε την τάδε επέμβαση,το τάδε χειρουργείο ασπουμε,το συγκεκριμένο χειρουργείο…εεε ……

***R : Ok,ντάξει και είπες μου και κάτι άλλο,που θέλω να σταθώ,είναι τα, ένα εμπόδιο από ότι εκατάλαβα που βρίσκεις είναι όταν παίρνει ultiva, το ultiva τι είναι δηλαδή; Είναι ένα φάρμακο το οποίο κρατά τους κοιμισμένους για παράδειγμα;***

A: ναι προκαλέι τζαι αναισθησία και ελαφρώς μυοχάλαση.

***R: OK,υπάρχει περίπτωση δηλαδή να εν τέλια ξύπνιος με τον σωλήνα στο στόμα;***

A: Ναι υπάρχει περίπτωση

***R: ..εεε εν με τούτα τα άτομα που παραπάνω που θέλω να ασχολήθώ, με τούτα τα άτομα πως επικοινωνείς τότε;***

A: …………Πάλε με τον ίδιο τρόπο.

***R: Με τον ίδιο τρόπο,οκ***

A: εε υπάρχει και περίπτωση να μεν μπορείς να επικοινωνήσεις,δηλαδή να έχουν ένα πρόβλημα στο mental status ασπούμε ή και ενας λόγος που μπορεί να ανέχονται και τον σωλήνα είναι γιατί, ..οκ μπορούν να αναπνεύσουν,αλλά νευρολογικά, η νευρολογική τους ασπούμε.. δυνατότητα εν τους επιτρέπει το να σηκώσουν το χέρι ασπούμε να πιασουν τζαι να φκάλλουν τον σωλήνα..εεεε

***R: οκ..***

A: Οπότε ενας ασθενής διασωληνωμένος νομίζω ενεν πάντα, εεε.. στο 100% του … mental status του

***R: OK, όπως τούτο το εμπόδιο που μου είπες τωρά, ενας ασθενής ενεν 100% του mental status του, τι άλλα εμπόδια βρίσκεις στο να επικοινωνήσεις με τούτους τους ασθενείς; Μπορείς να σκεφτείς να σκεφτείς καποια που εν ετσι.., μάλλον θέλω να μου περιγράψεις, σκέφτου μια συγκεκριμένη εμπειρία, ενας ασθενής ο οποίος ασπούμε σου έμεινε στον νου τζαι περίγραψε μου τζίντο, χωρίς να σου κάμνω οτιδήποτε ερωτήσεις,περίγραψε μου πως ηταν η εμπειρία σου με τζίνο τον ασθενή ασπούμε,τι μπορέις να σκεφτείς για τζίνο τον ασθενή;***

A: Ένα τελευταίο,το τελευταιο ασπούμε περιστατικό που είχα ηταν πολλά καλός*.. (γέλιο)*

***R: Δηλαδή;***

A: Δηλαδή.. εκαταφέραμε ασπούμε να επικοινωνήσουμε πλήρως τζαι λεκτικά,ενοώ, εμιλούσα του τζαι ακολουθούσε με πλήρως, εε σε φάση που του είχα πλέον πλήρη εμπιστοσύνη στο ότι έννα φρόνιμος και εν θα τραβήσει τον σωλήνα. Εεε..

***R: οκ.. άλλα εμπόδια που έβρισκες στο να επικοινωνήσεις με ασθενείς, για παράδειγμα ασπούμε είπες μου ηταν το ότι επαίρναν φάρμακα ασπούμε, για παράδειγμα το ultiva ή ότι εν ηταν εμ σταθεροί, πως το είπαμε, mental ασπούμε, mentality σταθεροί, είχε οποιοδήποτε άλλο εμποδιο που σε έκαμε να πεις ότι με τούτο τον ασθενή δεν μπορώ να επικοινωνήσω ας πούμε;…***

***Να σου δώκω παράδειγμα ασπούμε επονούσε και εν εμπορούσε να απικοινωνήσει; ή είχε κάποιο άλλο θέμα; Εεεε τζίνος συναισθηματικά πως ήταν ασπούμε; τι ηταν τα εμπόδια που εντόπισες;***

A: …………………………. Δύσκολη ερώτηση,

***R: ok, απλα ότι θυμάσε***

A: ………………Νομίζω εν αντιμετώπισα κάποιου είδους εμπόδια ασπούμε, δηλαδή αν, ειτε μπορείς να επικοινωνήσεις,είτε εν μπορείς να επικοινωνήσεις

***R.. δηλαδή τα μόνα εμπόδια που εντόπισες ως τωρά ήταν ασπούμε όταν δεν ηταν ψυχικά καλά ο ασθενής τζαι τα φάρμακα που παίρνει; Τούτα ηταν τα εμπόδια που αντιμετώπισες;***

A: τζαι ότι επροσπαθούσε να τραβήσει τον σωλήνα

***R: οκ δηλαδή ηταν εξαγριωμένος;***

A: Οι εξαγριωμένος, εεε , εν το αντανακλαστικό νομίζω του κάθε διασωληνωμένου το να θέλει να φκάλει τον σωλήνα που το στόμα του

***R: οκ..μάλιστα***

A: όποτε πρέπει να τον κρατάς χέρια πόδια

***R: Καθώς επικοινωνέις με αυτά τα άτομα τα διασωληνωμένα τα οποία είναι ξύπνια ποιες είναι οι ανάγκες του,καταλαμβαίνεις ποιες είναι οι ανάγκες του;***

A: Κάποιες φορέςς ναι

***R: .. αν ναι με πιο τρόπο να εντοπίσεις ποιες είναι οι ανάγκες του;***

A: Κάποιες φορές καταλαμβαίνεις,ρωτά τον ερωτήσεις, εεε , τυφλές ερωτήσεις ασπούμε τζαι προσπαθείς να πάρεις την σωστή απάντηση είτε με κάποια νοήματα όπως, αν πονεις τζιαμέ ..κλείσμου τα μάτια σου ασπούμε ή αν θέλεις τούτο το πράμα σφίξε μου το χέρι, ενοώ με έτσι τρόπους

***R: .. Δηλαδή τι ερωτήσεις τους κάμνεις; Ενοώ εν πάντα κλειστού τύπου ερωτήσεις που τους κάμνεις ή κάμνεις τους και ανοιχτού τύπου, δηλαδή ρωτάς τον πονείς; Πεινάς ασπούμε;***

A: Εν κλειστού τύπου συνήθως..

***R: …Κλειστού τύπου συνήθως,άρα χρησιμοποιάς τούτη την στρατιγική***

A: Nαι

***R: Υπάρχει κάποιο άλλο είδος στρατιγικής που χρησιμοποιάς για να καταφέρεις να επικοινωνήσεις με τούτα τα άτομα;.. για παράδειγμα να σου δώσω, μπορεί κάποιος ασθενείς ασπούμε να πονεί τζαι εσύ επροσεξες ότι κάθε φορα που πονεί , εε ,παράδειγμα κλήνει σου τα μάτια του***

A: μμχχμμ

***R: τούτο ασπούμε εσύ μεταφέρεις το με τους άλλους τους νοσηλευτές,κωδικοποιάς τούτο το μήνυμα,λέεις του ότι αμαν πονεί κλήνει μου τα μάτια,αρα εχε το υπόψη;***

A: οι οι οι, οι ενεν στανταρ

***R: oι***

A: Νομίζω ότι ο κάθε ασθενής είναι διαφορετικός,δηλαδή καποιος άλλος μπορεί να σου, δήξει ασπούμε, δηλαδή ενας καρδιοχειρουργιμένος, το τελευταιο περιστατικό που θυμούμε

***R: μμχχμμ***

A: Έδειχνε μου ο ίδιος,ότι..,έδειχνε μου το στήθος του

***R: ..ναι***

A: τζαι ερωτούσα τον, τι; πονείς ασπούμε;

***R: Ναι***

A: τζαι έκαμνε μου νόημα με το δάχτυλο ότι πονεί

***R: ok***

A: εσυμφωνούσαμε, έδειχνε μου ο ίδιος.

***R: μάλιστα, τι πιστευεις ότι νιώθουν αυτά τα άτομα τα οποία είναι διασωληνωμένα και ξύπνια αλλα δεν μπορούν να επικοινωνήσουν; Τι συναισθήματα πιστεύεις ότι νιώθουν;***

A: …………… Πιστεύω ότι ενεν και το πιο καλό πράμα, να είσαι διασωληνωμένος και να μεν μπορείς να επικοινωνήσεις, τζαι να είσαι τζαι ξύπνιος,εεε

***R: Τι πιστευεις ότι νιώθουν τζίνη την ώρα ή ότι αισθάνονται ασπούμε για παράδειγμα***

A: …….κοίτα εν μπορώ να.., μπορώ να φαντστώ ασπούμε

***R: ναι ενοείται***

A: εν είμουν στην θέση ποτέ κάποιου, εεε, αν εν κάποιος που καταλαμαβαίνει, δηλαδή αν το mental status εν καλό τζαι για κάποιο λόγο εν μπορεί ασπούμε να επικοινωνήσει,τούτο εν σιουρα παρα πολλά άσχημο, εεε….

***R: μπορείς να σκεφτείς εσυ κάποια στιγμή που επροσπαθούσες να επικοινωνήσεις με κάποιο ασθενή και δεν εκαταλάμβαινες τι σου έλεε; Ασπούμε έδειχνε σου***

A: ναι αρκετές φορές

***R: Μπόρεις να μου περιγράψεις μια συγκεκριμένη εμπειρία που σου έρχεται στο μυαλό ασπούμε;***

A: εεεε

***R: Πως ένιωθες εσύ όταν δεν μπορούσες να επικοινωνήσεις με τον ασθενή,όταν εκαταλάμβαινες ότι έκαμνες προσπάθειες και εκαταλάμβαινες ότι δεν εμπορούσες να καταλάβεις τι θέλει;***

A: Εσταματούσα να προσπαθώ

***R: Μάλιστα***

A: τζαι επροσπαθούσα να εξασφαλήσω το.., την ασφάλεια του κυρίως

***R: Μάλιστα***

A: Προσέχωντας τον να μεν κάμει κάποια κίνηση που μπορεί να τον βλάψει ασπούμε

***R: ok, εσυ όμως πως ένιωθες που δεν εμπορούσες να επικοινωνήσεις με τον ασθενή; Ένιωθες κάτι ασπούμε είτε θετικό είτε αρνητικό;***

A: οι, εν ενιωθα τίποτε

***R: ok, απλα εσταματούσες να προσπαθείς***

A: Ναι εσταματούσα να προσπαθώ ασπούμε,από ένα σημείο και μετά

***R: ok***

A: Eξαρτάτε βέβαια και τι ασθενής ένει,δηλαδή αν ξερείς ότι εν κάρδιοχειρουργημένος και στην πορεία ασπούμε ένα βελτιωθεί, ναι οκ, προσπαθείς και ξαναπροσπαθείς,αλλα εν κάποιος ο οποίος έχει μια εγκεφαλική βλάβη , εεε, ντάξει οκ,εεε ,προσπαθείς στην αρχή,μετα ντάξει, θέλει πολύ χρόνο

***R: Μάλιστα, εεε, από την εμπειρία σου, τι πιστεύεις ότι αυτά τα άτομα προσπαθούν να σου πουν όταν επικοινωνήσουν; Τι εν τζοινα που σου λεν συνήθως; οι τι πιστευεις, τι βλέπεις ασπούμε ότι ζητούν συνήθως ή προσπαθούν να σου πούν;***

A: ότι πονούν

***R: ναι***

A: εεε… ισως να διερωτούντε που βρίσκονται

***R: μμχχμμ***

A: τζαι εντάξει τούτο εν κάτι που το εξηγούμε τζαι εμείς πριν καν μας το ζητήσουν οι ίδιοι

***R: μμχχχμμ***

A: εεε….θέλουν να αλάξουν θέση και εν μπορούν

***R: μμχμμμ***

A: Κυρίως τούτα

***R: ok, στο τμήμα που δουλευεις υπάρχουν μέσα βοηθητικά επικοινωνίας, δηλαδή ασπούμε χρησιμοποιάτε πέννα χαρτί ή ταμπλετ ή κάτι ασπούμε το οποίο σε βοηθά να επικοινωνήσεις με τον ασθενή***

A: ..οι εν χρησιμοποιούμε κάτι,μόνο λεκτικά, τζαι…, δηλαδή μόνο μια φορά έτυχε νομίζω να δώσουμε σε ασθενή να γράψει, ο οποίος ηταν, δεν ηταν διασωληνωμένος, νομιζώ είναι πολλά,είναι extreme νομίζω, ενεν λιο extreme;

***R: Για ποιο λόγο εν extreme;***

A: το να έχεις ένα ασθενή διασωληνωμένο,ξύπνιο, τζαι να του δώσεις να γράψει

***R: ok, δεν…***

A: Θα εμπορούσε

***R: .. μιλώ μόνο για κόλλα και χαρτί, μπορεί ασπούμε να εν κάποιος υπολογιστής,δηλαδή να εν κάτι πιο εξειδικευμένο για παράδειγμα***

A: οι εν μου έτυχε

***R: Οι εν σου έτυχε,οκ,εεμμ, ποια είναι η άποψη σου για αυτά τα μέσα, δηλαδή για παράδειγμα, αν είχες κάποιο τάμπλετ τζαι έδινες το του ασθενή τζαι έδειχνε σου για παράδειγμα κάποιες εικόνες ή εβοηθα σε στο να επικοινωνήσεις με τον ασθενή,ποια εν η άποψη σου για τούτα τα μέσα;***

A: Νομίζω ηταν να δούλευκε,θα εμπορούσε να δουλέψει, εεε ιδίως ασπούμε τούτο με τις εικόνες που εν κάτι έυκολο και άμμεσο ασπούμε

***R: OK***

A: το να γράψει νομίζω εν λίο δύσκολο,θα ηταν λιο δύσκολο

***R: Για ποιο λόγο;***

A: Γιατί, νομίζω πρέπει να έχεις πλήρη διαύγεια στο να μπορέσεις ασπουμε να γράψεις και να σε φουλ στο

***R: άρα ακόμα ένα εμπόδιο που εντοπίζω δαμέ που μου μιλάς είναι η διαύγεια του ασθενή, δηλαδή ότι δεν έχει διάυγεια ασπούμε***

A: ναι

***R: ok, εμμ, έχεις δεχτεί προηγούμενη εκπαίδευση οσο αφορά τα μέσα διαφορης αα,βασικά ,σόρρυ, έχεις δεχτεί προηγούμενη εκπαίδευση οσο αφορά τεχνικές επικοινωνίας με διασωληνωμένους ξύπνιους ασθενείς;***

A: οι ποτέ.

***R: ποτε,οκ,θα ηθελες να εκπαιδευτεις σε αυτές τις τεχνικές, θα ηθελες να μάθεις τρόπους ασπουμε να αντιμετωπίζεις τον ασθενή πως να επικοινωνάς μαζί του όταν είναι ξύπνιος και διασωληνωμένος;***

A: ναι ναι ναι, θα μου άρεσκε ασπουμε

***R: ok,αν ναι ασπουμε όπως μου είπες τώρα, ποιο τρόπο ένα προτίμας; Ένα προτίμας διαδυχτιακά, με σεμινάρια, δίπλα που το κρεβάτι του ασθενή, εεεμ***

A: Οι απαραίτητα δίπλα από το κρεβάτι,νομίζω και διαδυχτιακά μπορεί να γίνει

***R: ok***

A: και σε ξεχωριστό χώρο μπορεί να γίνει

***R: Έχει κάτι άλλο τωρά που θυμήθηκες ή που θέλεις να μοιραστείς μαζι μου,κάποιο περιστατικό ιδιαίτερο που σου έμεινε να μου το περιγράψεις για παράδειγμα, εντζε θέλω, μεταξύ μας τωρά, οσο αφορά, μπορείς να σκεφτεις ένα περιστατικό ο οποίος ηταν διασωληνωμένος ξύπνιος και τι έκαμνες ουλλη νύχτα, τι εν τζόινο που επρόσεχες εσύ τι εν τζοίνο που σε ανησυχούσε.. ;***

A: Νομίζω ξέρεις καλύτερα (*ελαφρύ γέλιο*) πως ένει να περνάς μια νύχτα ασπούμε με ένα διασωληνωμένο,εν πολλά δύσκολο,ενοώ,εντάξει οκ, ένα τον κρατάς να τον προσέχεις συνέχεια, εν τον αφήνεις ποτέ που τα μάτια σου

***R: ok, μάλιστα***

A: Eιδικά στην εντατικη μας ασπούμε

***R: Γιατί συγκεκριμένα στην εντατική σας;***

A: γιατί… τους ασθενείς εν τους δίνουμε, απαγορεύεται, ενώ σε άλλες εντατικές, δίνουν τους συνήθως

***R: μάλιστα, οκ Σ μου, σε ευχαριστώ για τον χρόνο σου***

A: να σε καλά

Νο 2

**S: Stratos**

**R: researcher**

**R: strato**

S: Νίωθω όπως τους βουλευτές..

**R: Strato γεία σου, όπως είχαμε πει και από το τηλέφωνο,εμμ κάνω μια έρευνα που αφορά τις εμπειρίες επικοινωνίας των νοσηλευτών με διασωληνομένους ασθενείς,ξύπνιους στις ΜΕΘ**

Ναι..

**R: Θα συζητήσουμε έτσι λίγα πράματα όσο αφορά την δική σου οπτική γωνία, όπως ξέρεις και εσυ καλύτερα δεν έχει σωστό και λάθος,ότι μου πεις θα είναι για καλό, για να δω από πολλές απόψεις νοσηλευτών, να συλέξω διάφορες πληροφορίες, όπως γνωρίζεις και εσυ είναι ανώνυμα και σε αυτές τις πληροφορίες έχω μόνο πρόσβαση εγω και η καθηγήτρια μου,οποαδήποτε στιγμή αν θέλεις μπορούμε να διακόψουμε,λοιπόν,έτσι μερικές γενικές πληροφορίες,εκείνο που ήθελα να σε ρωτήσω πρώτα είναι, πόση εμπειρία έχεις γενικά ως νοσηλευτής;**

S: Ως νοσηλευτης εργάζομαι σε δημόσιο, ιδιωτικά περίπου 15 χρόνια,αναφορικά τώρα με τους διασωληνομένους ασθενείς,τα τελευταία δέκα χρόνια, σχεδόν δέκα χρόνια, δηλαδή τον φεβράρη του 2021 κλείνω δέκα χρόνια με διασωληνομένους ασθενείς

**R: Άρα αν αντιλαμβάνομαι σωστά, εργάζεσε δέκα χρόνια στην μονάδα**;

S: Στην ΜΕΘ ναι..

**R: Μπράβο,πολύ ωραία,πόσο χρονών είσαι;**

S: 37

**R: 37, ok, που έχεις τελειώσει αν επιτρέπεται;**

S: Στην νοσηλευτική σχολή κύπρου και ΤΕΠΑΚ

**R: Φαντάζομαι έκαμες μεταπτυχιακό, από ότι γνωρίζω; Αν θυμούμε καλα (;)**

S: Δυο μεταπτυχιακά, ένα στην διοίκηση μονάδων υγείας και ένα στην προηγμένη επείγουσα εντατική καρδιολογική νοσηλεία και ένα διδακτορικό στον ΤΕΠΑΚ όσο αφορά, με κατεύθυνση τέλος πάντων όσο αφορά την πρόληψη λοιμώξεων στην ΜΕΘ

**R: Μπράβο! Έτσι αστέρια θέλουμε να έχουμε σαν και εσένα. Το πρώτο πράμα που θέλω να σε ρωτήσω γιατί εγώ είμαι εκτός Μονάδων, έχει πολύ καιρό, παρόλο που έκανα το μεταπτυχιακό μου στις Μονάδες, είναι σε ποιες περιπτώσεις βλεπουμε ασθενείς που είναι διασωληνωμένοι και ξύπνιοι; Mπορείς να σκεφτείς που την δική σου Μονάδα;**

S: Διασωληνομένοι και ξύπνιοι,στις περιπτώσεις που χρειάζονται μόνο μηχανική υποστήριξη,περίμενε, αμαν λέμε διασωληνομένοι εννοούμε και την τραχειοστομία; Γιατί..

**R: Όχι, μόνο διασωληνωμένοι**

S: Μόνο τραχειοσωλήνα….. εε συνήθως όταν τους αφυπνίσουμε για να δούμε ένα επίπεδο συνείδησης μετά από καρδιακή ανακοπή, που θέλεις, την δεύτερη τρίτη μέρα να αξιολογήσεις λίγο το επίπεδο συνείδησης τους, ή όταν κάνεις δοκιμασίες για να τους φκάλεις που τον , τουλάχιστο από ότι θυμούμε,που τον αναπνευστήρα μετά από ένα χειρούργειο ή μετα ,τέλος πάντων, που κάποιες ημέρες νοσηλείας διασωληνομένοι. Δηλαδή στην ουσία μπορεί να δεις ασθενείς οι οποίοι έχουν ένα χαμηλό επίπεδο συνείδησης λόγω της προηγούμενης καταστολής που εδόθηκε, ξέρεις ώσπου να κάψει τα φάρμακα, ειδικά αν είναι και dormicum ή βενζοδιαζεπίνες που κάνουν ενεργούς μεταβολίτες, πρέπει να περασούν δυο και τρεις φορές από το συκώτι τους ώστς να μεταβολιστούν και να μην είναι effective πλεόν όσο αφορα την καταστολή, αλλα συνήθως είναι, αν τα πάρουμε έτσι, δυο τρεια πράματα, συνήθως είναι, όταν αφυπνίζεις τον άρρωστο μετά από ένα χειρουργείο το οποίο μπορεί να έκανε τρεις τέσσερις μέρες που είναι διασωληνομένος ή ασπουμε και λίγο μετα το χειρουργείο τελός πάντων, απλά είρτε για κάποιο λόγο στην Μονάδα,που χρειάστηκε να νοσηλευτεί στην Μονάδα, εε είτε μετα που νοσηλεία άσχετη με χειρουργείο, δηλαδη μια ανακοπή ή κατι το ,η οποία επροέκυψε και έπρεπε εκείνη την δεδομένη στιγμή να βοηθηθεί αναπνευστικά και να υποστηριχτεί αναπνευστικά και απλά ξυπνάς τον για να δεις ένα επίπεδο συνείδησης και αν εν καλός τότε να τον φκαλεις που τον, να του φκάλεις τον σωλήνα αλλά εντάξει, συνήθως παίρνει και μια διαδικασία , ενεν ετσι απλό .. εε τούτα

**R: Οκ, αν αντιλαμβάνομαι σωστά από τις δικές μου γνώσεις, γιατί όπως σου είπα και πριν δεν έχω και τόση εμπειρία στην Μονάδα, δηλαδή είναι η φάση του weaning**

S: Ναι ναι

**R:..οταν θα ξυπνίσεις ένα ασθενή, ε και στις δυο περιπτώσεις, απλα εν δυο διαφορετικές φάσεις του weaning**

S: Εν δυο διαφορετικές ,ναι

**R: Ασπούμε, εν μια που χειρουργείο και εν μια που οξεία φάση τέλος πάντων**

S: Ναι, κάτι.., βασικά είναι ένα αίτιο το οποίο τους οδήγησε να μπουν και διασωληνωθούν και απλά εν μια διαδικασία weaning, ναι εν τούτο ή απλά μπορείς να τον ξυπνίσεις για να δεις ένα επίπεδο συνείδησης, δηλαδή, όπως είπα και πριν, γ’αυτό ανέφερα και την ανακοπή, αν έκαμε μια ανακοπή, θέλεις να τον ξυπνήσεις για να δεις αν έχει εγκεφαλική λειτουργία, αν βήχει, ανν, βασικά, ξυπνάς τον , αξιολογάς το επίπεδο συνείδησης να δεις αν έμεινε κάποια νευρολογική βλάβη ή καποια νευρολογική ζημιά μετά από ένα οξύ επεισόδιο ασπούμε

**R: Οκ, μάλιστα, ευχαριστώ γιατί ήθελα να το διευκρινίσω αυτό, να δω σε ποιες περιπτώσεις σην Κύπρο έχουμε αυτά τα περιστατικά γιατί εν πολλά συγκεκριμένο τούτο που ρωτώ ασπούμε και ήθελα να δω σε διάφορες Μονάδες κάποια άλλη αιτία ή λόγος να έχουν ασθενείς διασωληνομένους χωρίς καταστολή, εντάξει με κάλυψες νομίζω για την δική σας Μονάδα**

S: Ή και άτομα τα οποία μπορεί να κρατήσουν αεραγωγό, δηλαδή σε μια τραχειομαλακία, αλλά δεν ξέρω αν φτάνει τόσο βαθεία, συνήθως προχωρούν σε άμεσες τραχειοστομίες, δηλαδή τούτο το πράμα μπορεί να κάμει δυο τρείς, τέσσερις ώρες άτε να κάμει το πολύ μια μέρα αλλά ενταξει,επειδή δεν είναι ευκολο να νιώθεις ένα σωληνα στον λαιμό σου,συνήθως αμαν χρειαστεί παράταση της μηχανικης υποστήριξης τότε προχωράς έτσι άμμεσα σε τραχειοστομία που μπορείς να κόψεις και τα φάρμακα πιο εύκολα,μπορείς να βάλεις μια βαλβίδα φώνησης να σου μιλήσει ο άρρωστος και τούτα, αλλάα.. εν μπορώ να σκεφτώ κάποιες άλλες περιπτώσεις εκτός που τούτες που σου είπα,δηλαδή ένα weaning μετά που κάτι που έτυχε,ή πουκάτι το οξύ παθολογικό ή κάτι το οξύ χειρουργικό ή μια ανακοπή ασπούμε ή κατι που χρειάστηκε έτσι νοσηλεία

**R: Μάλιστα, ωραία,εμμ ετσι γενικά τώρα, πως είναι, εμμ να προσπαθείς να επικοινωνάς με αυτούς τους ασθενείς, αν θέλεις μπορείς να μου περιγράψεις ένα , αν σου έρχεται κάποια συγκεκριμένη εμπειρία στο μυαλό μπορείς να μου το περιγράψεις, ενόω δεν έχει, να μου περιγράψεις μια εμπειρία σου γενικά προσπαθώντας να επικοινωνήσεις με αυτούς τους ασθενείς**

S:Ντάξει, συνήθως το πρώτο πράμα που νιώθουν οι ασθενέις, βασικά , όταν πλέον κόψεις τις καταστολές και ξυπνήσεις έναν άρρωστο καταλαμβαίνεις λίο τον άρρωστο ότι ξύπνησε που τις σφίξεις, λιο που την ταχύπνια,λιο που την αγωνία γιατί εν τούτο που σας είπα πριν, νιώθεις ένα σωλήνα μέσα στον λαιμό σου ο οποίος κατεβαίνει στην τραχεία,ενεν έυκολο και συνήθως τούτος ο σωλήνας ένεν μόνος του, συνοδεύεται με άλλα πράματα, δηλαδή, ρινογαστρικούς,όρογαστρικούς που πάλε προκαλούν, ένα αίσθημα έτσι δυσφορίας, γ αυτό και παίρνουν κάποιες καταστολές και την αναλγισία, παραπάνω την αναλγισία, αλλά όταν προασπαθείς να ξυπνήσεις τους άρρωστους το πρώτο πράμα που νιώθω, όταν περάσει τζινη η δράση της, από ότι τουλάχιστο ξέρω και από ότι βλέπω, δήχνουν σου τον σωλήνα μεσα στο στόμα, προσπαθούν να σου μιλήσουν και εν φκένει η φωνή τους και ανησυχεί τους τούτο το πράμα, βλέπεις τους ότι ανησυχούν και έτσι εν τζιαμε που, εν ενας που τους ρόλους του νοσηλευτή να του εξηγήσει, το τι έγινε, το πως εσυνέβηκε, γιατί μπορεί να έγινε κάτι στο δρόμο, έναν τροχαιο ή μια ανακοπή, κάτι ή να μπήκαν χειρουργίο να περάσουν τρείς τέσσερις μέρες και να ξυπνήσουν και να μην ξέρουν που είναι ή τι εσυνέβηκε, ε μα γιατί αφου είμουν στο σπίτι μου, πως εβρέθηκα στο νοσοκομείο με τον σωλήνα στο στόμα, ε τζιαμέ πιστεύκω εν ο ρόλος του νοσηλευτή, δηλαδή, να ξέρει να γνωρίζει πότε ξύπνησε ενας άρρωστος, τα σημάδια που όταν ξυπνά, λιο τις σφίξεις λίο την αγωνία, βλέπεις τον ότι κάμνει μια ταχύπνια,βλεπείς τον ότι ασπόύμε ,ξέρεις, τι συμβαίνει,καταλάβεις έτσι την έκφραση του προσώπου, την αγωνία βασικά που νιώθει τούτος ο άρρωστος, πάεις κοντά στον άρρωστο και τότε εξηγάς του, κύριε τάδε ασπούμε έχεις ένα σωλήνα μεσα στο στόμα σου και έτυχε τάδε, ντάξει το πιο πιθανό, ειδικά αν παίρνουν βενζοδιαζεπίνες όπως το dormicum εν θα θυμούντε κάτι μετά, μπορεί να τους το .. θυμίσεις δέκα φορές κατά την διάρκεια της βάρδιας και πάλε εν ενας καλός τρόπος, εφόσον ενεν σε θέση ακόμα να αφερεθεί ο σωλήνας, να είσαι κοντά του , να τους μιλάς ούτως ώστε να τους πάρεις, να τους επαναφέρεις στην πραγματικότητα που ζουν για να αντέξουν τον σωλήνα χωρίς να χρειαστεί να τους δώσεις κάτι επιπλέον, γιατί αν εξύπνησε ,εν ξέρει που βρίσκεται,κάμνει μια ταχύπνια, κάμνει μια ταχυκαρδία,ανεβάζει σφύξεις, ανεβάζει πιέσεις,ανεβάζει αναπνοές, τότε καταλαβαίνεις ότι κάμνει μιαν αιμοδυναμική αστάθεια η οποία μπορεί να οδηγήσει σε άλλα πράματα, δηλαδή μια τυχαία αποσωλήνωση αν είναι ανήσυχος και πάμε λέγοντας και έτσι τι χρειάζεται(;) , να τον ξανακοιμήσεις και είναι ενας φαύλος κύκλος, δηλαδή ξυπνάς τον, εε εν ανήσυχος ,αρα εν τζιαμέ που πιστευκώ ότι εν ο ρόλος του νοσηλευτή, να σταθεί λίο κοντα του να του υπενθυμίζει,μπορεί να χρειαστεί να κάμει δέκα φορές αλλά εν καλά να του υπενθυμίζει γιατι γλυτώνεις και διάφορα τυχαια ***(δεν είμαι σιγουρη τι λέει λογω της φασαρίας στο background)*** ….που να μπορούσες να τον φέρεις σε ένα στάδιο που να είναι ασφαλές. Αυτά!

**R: Ωραία, είπες μου πάρα πολλές πληροφορίες που θέλω να πιαστώ έτσι μερικά που τούτες. Εεε είπες μου κάποιους τρόπους που επικονωνάς ασπούμε αν αντ.. , προσπαθώ να τα κατηγοριοποιήσω λίγο στο μυαλό μου,εε θα ήθελα να μου ξαναπέις αν μπορείς να μπου εμπλουτίσεις λίγο τους τρόπους που καταφέρνεις να επικοινωνήσεις με αυτούς τους ασθενείς, δηλαδή εσύ ασπούμε πως επικονωνάς με αυτους τους ασθενείς, πως αντιλαμβάνεσαι τις ανάγκες του για πάρδειγμα, σεε οτιδήποτε, ασπούμε ξυπνά ένας ασθενής λεεις μου, πως αντιλ.., πάει να σου πει κάτι, με ποιο τρόπο πάει να σου πει κάτι, με ποιο τρόπο αντιλαμβάνεσαι τι θέλει, ή με ποιο τρόπο επικοινωνάς μαζί του;**

S: Συνήθως..

**R: Όταν ο ασθενής είναι για παράδειγμα λίο πιο ξύπνιος**

S: Συνήθως αμαν δεις ένα άρρωστο ξύπνιο την ώρα που ένα κοντέψεις το πρώτο πράμα που θα κάνει είναι να γυρίσει προς τα πάνω σου, ένα σε δει στο οπτικό του πεδίο, θα γυρίσει προς τα πάνω σου, όταν γυρίσει προς τα πάνω σου , αν θέλει κάτι να σου πει ένα προσπαθέι να σου μιλήσει, αν προσπαθεί να σου μιλήσει καταλαμβαίνεις ότι εν φκαίνει φωνή του και αρχίζει και ο αναπνευστήρας και ,ειδικά αμαν εν πάνω στον αναπνευστήρα και δεν είναι σε Τ *(* ***ενοεί t piece, που μπαίνει στην τραχειοστομία)*,** αρκέφκει ο αναπνευστήρας και παίζει ότι κάτι συμβαίνει ή αναιβάζει Pip/PEEP ; (***Mode στον αναπνευστήρα που αφορά τις πιέσεις στους πνευμονες***) ή προσπαθεί να δήξει κάτι, τότε καταλαμβαίνεις ότι προσπαθεί να σου μιλήσει, ε τι ένα κάμεις; Ένα πάεις πάνω του, ένα του πείς κάτι , κυριε εν μπορείς να μιλήσεις,ξαναεξηγάς του ότι εν μπορείς να μιλήσεις γιατί έχεις ένα σωλήνα μέσα στο στόμα,σιγά σίγα ένα φκεί τζίνος ο σωλήνας, έχεις τον γιατί προς το παρόν είναι αναγκαίος και ότι εν πρέπει να τον φκάλεις, ένα τον φκάλουμε εμεις γιατί αν τον φκάλεις ένα έχουμε άλλο θέμα αν φκει πριν την ώρα του κτλ, τώρα ,πως επικοινωνάς μαζί του, μιλάς του , λέεις του πονάς κάπου; Ενοχλεί σε κάτι; τι σε ενοχλεί; και συνήθως αμαν τους ρωτήσεις τι σε ενοχλεί, δείχνουν σου τον σωλήνα, άρα καταλαμβαίνεις ότι τα, τουλάχιστο τζίνη η δυσφορία τζίνη η ανησυχία ή τζίνο που θέλει να σου πει εναςς άρρωστος που ένα ξυπνήσει , εν τζίνο που τον ενοχλεί, τζίνο που τον ενοχλεί συνήθως εν ο σωλήνας και εν τζίνο που μας δείχνουν σχεδόν τις πλείστες των περιπτώσεων εχτός και αν έχουν κάτι άλλο ασπούμε πιο οξύ, αν πονούν την κοιλία τους, δηλαδή , αν βασικά λέουν σου τζίνο που τους ενοχλέι περισσότερο, αν πονούν την κοιλία τους ένα σου πουν την κοιλία τους και ύστερα ένα σου πουν και τον σωλήνα,αν είναι ο ουροκαθετήρας ένα σου πουν τον ουροκαθετήρα και ύστερα ο σωλήνας, άρα προσπαθούν να σου δήχνουν, ρωτάς και εσύ διάφορα, έχει πολλά πράματα τα οποία μπορούν να τους ενοχλούν, εν ο σωλήνας εν η κοιλία, εν ο καθετήρας, εν η θέση, εν μια φλέβα, εν κανένας κεντρικός, εν και έχει και πολλά πράματα που μπορεί να τους ενοχολούν τους αρρώστους μέσα σε μια μονάδα

**R: Οκ**

S: Εεε εντάξει τούτα συνήθως θέλουν να μας πουν τις πρώτες στιγμές που φκαίνουν εχτός αναπνευστήρα

**R: Κάτι που τους πονεί δηλαδη; Κατι που τους ενοχλά ;**

S: Συνήθως κάτι που τους πονεί κατι που τους ενοχλεί, κατι που τελος πάντων τους προκαλέι έτσι μια δυσφορία, είτε προς τον πόνο ,είτε προς το κάτι εν μου αρέσκει ή κατι νιώθω

**R: Οκκ, εε δηλαδή αν αντιλήφθηκά σωστά , ένα από τα εμπόδια που βρίσκεις να επικοινωνήσεις με αυτούς τους ασθενείς, το πρώτο που εν λογικό, είναι ο σωλήνας..**

S:Ναιι

R: **Βρίσκεις κάτι άλλο το οποίο σε εμποδίζει να επικοινωνάς με αυτούς τους ασθενείς, η ολή κατάσταση, δηλάδή,ποια εν τα εμποδια που βλέπεις εκείνη την στιγμή τα οποία εμποδίζουν σε να επικοινωνήσεις;**

S: Αμαν λέμε επικοινωνία, ενοούμε μια άμμεση επικοινωνία, γεια σου κ. Αντρέα, είσαι καλά, πονεί σε ο σωλήνας, νιώθεις κάτι;

**R: Γενικά, γενικά.. Ενοώ γενικά που την εμπειρία σου**

S: Συνήθως τζίνο το άλλο που εν σε αφήνει τόσο πολλά να επικονωνήσεις μαζί τους είναι ο χρόνος, δηλαδή αμαν είμαι πρωι και έχω τρεις αρρώστους διασωληνομένους και έχω χίλιες δυο παρεμβάσεις να κάνω, αμαν φκάλω και έναν άρρωστο .. αμαν προσπαθώ να ξυπνήσω και ένα άρρωστο αλλα έχω ακόμα δυο ααρρώστους υπο την ευθύνη μου καταλαμβαίνεις ότι εν λίγο δύσκολο να του δώσω τόση ιδιαίτερη σημασία και να τον ρωτήσω αν πονεί ή αν δεν πονεί ή αν νίωθει δυσφορία ή εστω και να κάτσω ασπόυμε δέκα λεπτά μαζί του γιατί έχω ακόμα δύο (***γέλιο)*** αντιλαμβάνεσαι εχτός που τον σωλήνα τον πόνο ή την δυσφορία πιστεύκω και η έλλειψη χρόνου εν μεγάλο θέμα ή και ο φόρτος εργασίας.

**R: Νομίζω τούτο είναι παντού η αλήθεια, εεε πως νιώθεις γενικά όταν δεν καταφέρνεις να επικοινωνήσεις; Αν νίωθεις κάτι ενοείται.. εε δεν θέλω να κατευθύνω την ερώτηση, δηλαδή αν μπορείς, να σου το κάμω διαφορετικό , οκ, έρχεται σου στον νου κάποια συγκεκριμένη εμπειρία που εκατάφερες ή δεν εκατάφερες να επικοινωνήσεις με ένα ασθενή διασωληνομένο ξύπνιο και θέλεις να μου την περιγράψεις;**

S: Συνήθως ένας άρωστος ο οποίος ένα κάμει αρκετό καιρό μέσα στην μονάδα ,ντάξεια, αναπτύσει ένα ντελίριο, στην ουσία έχει πολλούς παράγοντες που επηρεάζουν και συντηνουν στην ανάπτυξη τούτου του ντελίριου, δηλαδή φώτα ούλη μέρα, δεν καταλαμβαίνει πότε εν νύχτα και πότε εν μέρα ,φασαρία, άγνωστο περιβάλλον, άγνωστος κόσμος γυρό, κόιτα πολλές φορές εν σε θέση να επικοινωνήσει, το θέμα όμως είναι ότι αμαν επηρεαστεί τόσο πολλά που το ντελίριουμ και γίνεται, καταρχήν βλέπει διάφορα πράματα αρα αντιλάμβάνεσαι ότι που την στιγμή που βλέπει ο άλλος κάποιον να έρχεται να τον σκοτώσει, λέμε τωρά, επειδή τυχαίνει, εξαναέτυχε μας, α ετον που έρχεται να με σκοτώσει, αρα αντιλαμβάνεσαι ότι εν λίο δύσκολο να του εξηγήσεις ότι ξέρεις είσαι μέσα στην μονάδα που την στιγμή που βρίσκεσαι σε ένα χώρο ο οποίος είναι εχθρικός ή σε ένα χώρο που νομίζει ότι εν μια αποθήκη ή σε ένα χώρο που νομίζει εν το σπίτι του ή ξέρω γώ εν η δουλεία του και λέει σου φώναξε του κωστάκη που τζικάτω ή δήχνει μου , συνήθως το ντελίριουμ εν μεγάλο κομμάτι, ίσως εντάξει, ίσως οι αρρώστοι με τους σωλήνες δεν μπορούν να επικοινωνήσουν τόσο πολλά όπως, ξέρεις αν έχει κάποιον ο οποίος, αλλά καταλαμβαίνεις κάποιον ο οποίος νιώθει ότι απειλείται, κοντευκεις του και νομίζει ότι κάτι συμβαίνει, ότι και μετά που τους ξυπνάς, να το πάρουμε και ένα βήμα πιο κάτω, λέεουν σου ποιος εν τζίνος που έρκεται, νιώθω ότι δεν είμαι καλά ή νιώθω ότι είμαι σε ένα χώρο όπως σου ειπα πριν εν εχθρικός ή νιώθω όπως σου είπα πριν ότι εν το σπίτι μου τουτο ή που είσαι ασπούμε; Εν το σπίτι μου, τούτος εν ένας , μια άλλη μορφή που εμποδίζει την επικοινωνία, είτε διασωληνομένοι είτε αποσωλήνοτος, αλλα στο διασωληνομένος εν ακόμα χειρότερα, προσπαθείς να τον ξυπνήσεις, που την στιγμή που προσπαθείς να τον ξυπνήσεις και είναι ήδη σε ντελίριουμ , τότε εν επικύνδινο να φκάλει άλλα πράματα, να φκάλει τον σωλήνα πριν την ώρα του, μια αποσωλήνωση τυχαία ή σκόπιμη εκ μέρους του ή κάτι που τον ενοχλεί, τούτο εν ένα κομμάτι που πάλε εν ασχημο και εμποδίζει την επικοινωνία. Επέλανα σε;;

**R: Οι οι αρέσκει μου,εεε απολαμβάνω το γιατί εισαι ο μόνος που , η αλήθεια , είσαι το δευτερο μου άτομο που κάνω την συνέντευξη αλλά χαίρομαι πάρα πολλά γιατί λεεις μου πάρα πολλές πλούσιες πληροφορίες και χαίρομε το, εν μου είπες το δικό σου κομμάτι, δηλαδή είπες μου που την πλευρά του ασθενή, ότι μπορεί να μην αισθάνεται καλά, να είναι αγανακτησμένος, ντάξει μπορεί να μην εχρησιμοποίησες την λέξη αγανακτησμένος, ενοώ τούτο μου δήνει εμενα στο μυαλό,που την δική σου πλευρά πως νιώθεις γι αυτό το πράμα, δηλαδή όταν δεν καταφέρνεις να επικοινωνήσεις, τι νιώθεις εκείνη την ώρα; Τι είναι οι ενέργειες σου που θα κάνεις για παράδειγμα;**

S: Αν είναι τόσο ανήσυχος, αν προσπαθείς να επικοινωνήσεις και απλά εν συνενοάσε λόγω του ντελίριου ή εν τόσο επικυνδινό εν μπορείς να κάνεις κάτι περα που το να κάνεις ενέργειες ουτως ώστε να τον κατά, οι να τον καταστήλεις ήπια, να τον φέρεις σε ένα στάδιο το οποίο δεν θα είναι επικύνδινο για τον ευατό του εφόσον μιλούμε για επικοινωνία η οποία έχει σχέση με το ντελίριο

**R: Μάλιστα..**

S: Αρα τι ένα κάμεις, ένα δεις, ίσως χρειάζεται μια δόση περισσότερη Seroquel ίσως χρειάζεται ασπούμε μια Haldol, γιατί η Haldol εν που τ πιο καλά φάρμακα για το ντελίριουμ, και το Seroquel βέβαια αλλά το hadol, ίσως εν κάτι που τον ενοχλει και μπορώ να κάμω, δηλαδή καμιά φορά βλέπεις τους αρώστους να προσπαθούν να σου μιλήσουν ή να μην συνενοάσε μαζί τους απλά επειδή τους ενοχλά κάτι πολύ απλό, δηλαδή ασπούμε μπορεί να εν διπλωμένο λιο το σεντόνι τζιαμέ στην πλάτη τους και να νιώσει ασπούμε ότι κι προσπαθείς απλά και ήπια να τους βάλεις σε μια θέση η οποία να εν άνετη ούτως ώστε να μπορείς και εσυ να συνενοηθέις μαζί του, δηλάδή αν είναι κάτι που τους ενοχλέι τόσο πολλά πέρα από τον σωλήνα θα εν καλό να δούμε και όλα τα υπόλοιπα, ρε έχει κάτι, εκατου.., μηπως εν καμιά φλέβα και κάυκει τον αλλά λόγω της όλης κατάστασης εν μπορεί να μου το περιγράψει, ξέρεις εν διάφορα πράματα, που πιστεύκω αμαν φτίαξεις τα άλλα και αφήνεις εκείνα τα πράματα που πραγματικά ενοχλούν τον αλλά εν μπορείς να κάμεις κάτι για τζίνα , νομίζω βελτιώνεται λίο η επικοινωνία, εε αλλά και πάλε ενεν πάντα, τωρά σίουρα το καλύτερο πράμα για τους αρώστους εν να τους φκάλεις που τον σωλήνα , εν δεδομένο, αμαν εν εφικτό γιατί να μεν το σκεφτούμε να τους φκάλουμε , συνήθως προσπαθούμε να αφήνουμε τους αρώστους όσο πιο λίγο διασωληνομένους και οσο πιο γρήγορα να τους φκάλουμε για να αποφεύγουμε και τούτο που σου είπα, εκείνο το ντελίριουμ, να αποφεύγουμε άλλα που έχουν σχέση με την ίδια την παρουσία του τραχειοσωλήνα ασπούμε, σαν λοιμώξεις, σαν κάτι, εε προσπαθούμε να τους, οσο πιο λιο να τους έχουμε διασωληνομένους και με τον σωλήνα ίσως εν γ΄αυτό που, έχουμε αρκετές εμπειρίες με τους διασωληνομένους αλλά όσο αφορά την επικοινωνία συνήθως ,λιο μετα που ένα τους ξυπνήσουμε για να τους φκάλουμε που τον σωλήνα

**R: Αντιλαμβάνομαι βέβαια,εγώ τις εμπειρίες τις δικές σου θέλω να μάθω, έστω και εκείνο το λίο..**

S: Λιοοο, εε αν τον δείς ότι δεν είναι σε θέση να φκεί και γίνεται επικύνδινος , τότε ξαναπάεις πίσω και ξαναδοκιμάζεις τον , άρα τούτο το κομμάτι δεν διαρκεί πάρα πολλά, τωρά αν μου λέτε άτομα με τραχειστομία ηταν να είναι εντελώς διαφορετικό, γιατί, εφόσον θα έχει μια παράταση του χρόνου της νοσηλείας και της αναπνευστικής υποστήριξης και προχωρήσεις σε μια τραχειοστομία τούτο εν πιο έυκολο, εν πιο έυκολο που άποψης τι, εν έχει κάτι να τον ενοχλεί και συνήθως η τραχειοστομία δεν ενοχλεί άρα μπορείς να επικοινωνήσεις πολλά ωραιά με τζίνο τον ασθενή, μπόρει να σου μιλά με βαλβίδα φώνησης, τούτα νομίζω… και εσείς ξέρετε καλύτερα, ρωτάτε με κάτι άλλο γιατί έπιασα την κουβέντα.

**R: Οι αρέσκει μ ,αρέσκει μ πάρα πολλά.εμμμ να σε ρωτήσω κατι διαφορετικό που δεν έχουμε πει νομιζώ ως τωρά. Ηθελα να σε ρωτησω στο τμήμα σας ,στην μονάδα σας έχετε μέσα εμμ επαυξητικής επικοινωνίας,δηλαδή τούτα τα μέσα μπορεί να είναι κάτι απλό,για παραδειγμα, εεε κάτι πολύ απλό που μπορεί να χρησιμοποιήσει οποιοσδήποτε είναι πένα και χαρτί.. εδοκιμάσετε ποτέ πένα και χαρτί;**

S: Πάρα πολλές φορές..

**R: Άρα ενας τρόπος εν τούτος να επικοινωνήσετε, καταφέρνουν να επικοινωνήσουν με την πένα και το χαρτί;**

S: Όχι πάντα. Συνήθως ζητούν μας το οι ίδιοι, αμαν ,προσπαθούμε την ώρα που εν το επισκεπτήριο αμαν ενας άρρωστος εν στην θέση να επικοινωνήσει εστω και λίγο,για να προλάβουμε το ντελίριουμ,δηλαδή ένα από τα μέτρα πρόληψης του ντελίριου που σας είπα πριν είναι η πιο συχνή επαφή με την οικογένεια του, δηλαδή επαφή με την οικογένεια,πέρα από τα φαρμακευτικά, εεε γιατί είναι μια δέσμη/bundle μέτρων, ενεν απλα ένα μέτρο, ή τουτο κάμνει ,τούτο εν κάμνει,συνήθως λαλούν σου ότι εν σε θέση να επικοινωνήσουν,το μεσημέρι λίο πριν το επισκεπτήριο κόφκουν την καταστολή ούτως ώστε να μιλήσουν με τους συγγενείς τους, αν δύνατε(γίνεται) και συνήθως έρχονται και λέν μας εν καταλαμβαίνω τι μ λέει και συνήθως οι ίδιοι ή οι συγγεννεις ζητούν μας εν απλό ένα χαρτί και μια πέννα να γράψουν,συνήθως όμως εν τα γράφουν τόσο εύκολα,γιατί καταλαμβαίνεις αμαν παίρνεις ένα σωρό καταστολές και σταματήσου σου το μισή ωρα πριν, λίο η αδυναμία,λίο η δυσκολία

**R: εμμ οκ, εχτός που την πέννα και το χαρτί που εν ένα απλό μέσο επικοινωνίας,χρησιμοποιήσατε ποτέ πίνακες με εικόνες ή γράμματα ή κάτι όπως το τάπλετ για παράδειγμα, εβοήθησε σας; κάποια άλλα μές να επικοινωνήσετε;**

S: Aν το κάμνουμε σαν ρουτίνα ή αν το κάμνουμε πάντα ή αν έτυχε να το κάμουμε;

**R: και τα δύο**

S: Πάντα δεν το κάμνουμε,τούτο εν δεδομένο,τωρά όμως αν έχεις ένα μικρό ο οποίος εν δεκαοχτώ δεκαεννιά χρονών, ο οποίος έχει την ανάγκη να επικοινωνήσει με ένα δικό του, ή δεκαέξι χρονών,που καταλαμβαίνεται ότι δεκαέξι χρονών εν ανήλικος αλλά δεν μπορεί να είναι συνέχει η οικογένεια του σε μια εντατική,άρα μπορεί τουτός ο άρρωστος που μας έτυχε να εν σε θέση να μιλήσει, έχουμε τάμπλετ στην δουλειά.

**R: Aρα παρέχουν σας το οκ..**

S: Παρέχουν μας το εν σχετικό.

**R: Εν για τούτο τον λόγο ή παρέχουν σας το για το προσωπικό για παράδειγμα;**

S: Οι για το προσωπικό, εν για τους ασθενείς.Βασικά εβρέθηκε μια δυο εταιρίες και .. επιχορηγούν τα για τούτο το σκοπό, δηλαδή ενας άρρωστος π εν σε θέση έστω και με τραχειοστομία να μπορεί να επικοινωνήσει, ε έχουμε ωραίο προγραμματάκι πάνω, το οποίο μπορούμε να βάλουμε και τηλεόραση να βλέπει,…οι πάντα

**R: Aρα εν για ψυχαγωγία του ασθενή δηλαδή το..**

S: Πρώτο για ψυχαγωγία,δευτερο μπορούν να μιλήσουν με την οικογένεια τους ή βασικά να τον δουν να τους δεις,τζιαμέ που μπορούμε και δέχονται, εν και θέμα προσωπικών δεδομένων,εν μπορώ ασπούμε να τον δείχνω παντού χωρις να το θέλει ο ίδιος

**R: Ναι ναι ναι ενοείται**

S: Έτυχε όμως να το χρησιμοποιήσουμε σε αυτό τον σκοπό

**R: Mάλιστα, εν έχεται όμως κάποιο συγκεκριμένο πρόγραμμα που να πείτε, από ότι εκατάλαβα δηλαδή,που να το χρησιμοποιάτε για την επικοινωνία σε εσας με τους ασθενείς, όχι του ασθενή με την οικογένεια,δηλαδή δεν έχετε κάποιες εικόνες ή οτιδήποτε για να δείχνετε..**

S: Όχι όχι

**R: Άλλο που ήθελα να σε ρωτήσω,στην εκπαίδευση σου εσύ,στο πτυχίο σου ή στο μεταπτυχιακό σου ή στο phd σου ,που εν νομίζω να έκαμες κάτι τέτοιο,στο phd ενοώ, έχεις εκπαιδευτεί για την επικοινωνία, με τους ασθενείς;**

Σ: όχι αλλά έχεις εκπαιδευτεί στο να αναγνωρίζεις έναν άρρωστο ότι εξύπνησε,ότι εν ξύπνος και ότι κάτι τον ενοχλέι, τωρά το τι ακριβώς τον ενοχλεί,πρέπει να τον ρωτήσεις,άρα όι.

**R: ok, πιστεύεις αν σου δινόταν η ευκαιρία να εκπαιδευτείς λίγο περισσότερο με αυτούς τους ασθενείς, τον τρόπο επικοινωνίας σου,θα το έκανες, θα ήθελες να εκπαιδευτείς ή οχι;**

S: Γιατί οχι; Αν και νομίζω ότι, αν είχαμε τον απαραίτητο χρόνο να κάτσουμε κοντά στον άρρωστο, δεν θα χρειαζόταν κάποια μορφή εκπαίδευσης,μόνο και μόνο να κάτσεις κοντά του να του μιλήσεις, να τον ρωτήσεις, δηλαδή μπορεί να μεν μπορεί να σου απαντήσει, και να του πεις κλείστα μάτια σου αν πονείς,άνοιξε τα αν δεν πονείς ή ένα σε ρωτώ διάφορα και αμαν νιώθεις κάτι και συμφωνείς πές μου, δηλαδή πονείς το κεφάλι σου ασπούμε μεν μ κλήσεις τα μάτια σου,πονείς την κοιλία σου κλείσμου τα μάτια σου,εκατάλαβες , εν νομίζω να χρείαζεται και τόση πολλή εκπαίδευση στο να επικοινωνήσεις ασπούμε με τους ασθενείς, χρόνος χρειάζεται, όρεξη και προσπάθεια.τούτα πιστευκω ότι εν αρκετά..

**R: Μάλιστα.ωραία, σε περιπτώση ασπούμε που εγινόταν τούτη εκπαιδευση αν και αντιλαμβάνομαι τούτο π μ λέεις ότι ίσως κατεσένα να μην χρειάζεται..**

S: O οποιοσδήποτε μπορεί να είναι ο πιο εκπαιδευμένος που ούλλα δεν σημαίνει όμως ότι τζίνα που ξέρει ένα εφαρμοστούν στην πράξη ή αν μπορεί να τα εφαρμόσει στην πράξη ή αν θέλει να τα εφαρμόσει στην πράξη,αρα η εκπαίδευση που μόνη της δεν οδηγεί απαραίτητα σε τζίνα ούλλα τα πράματα που έμαθες στην πράξη

**R: Σωστό**

S: Eεε νομίζω σίουρα μια εκπαίδευση εν αρκετά καλό πράμα, αλλά αν εκπαιδευτώ εγω να επικοινωνώ με τους αρώστους ή τύχω μιας πιο προχωρημένης εκπαίδευσης και να επικοινωνώ με τους αρρώστους, όμως, έχω δέκα αρρώστους αντι δυο, καταλαμβαίνει ότι εν πρόβλημα, εν θα μπορώ να μιλήσω με κανένα..

**R: Με πιο τρόπο πιστευεις θα ηταν ο καλύτερος τρόπος για εσένα να εκπαιδευτέις,ασπούμε με σεμινάρια, εεε με τους ασθενείς δίπλα στο κρεβάτι, με πιο τρόπο, ένα ηταν καλά κάποιος να σου έδειχνε δίπλα από ένα ασθενή και να σ εξηγά τον τρόπο, ποιος πιστευεις ένα ηταν ο καλύτερος τρόπος; ΑΝ σε περίπτωση που**

S: Ένα πρόγραμμα το οποίο πιστεύω ότι θα εμπορούσε να βοηθήσει, εν ένα πρόγραμμα μεικτό,δηλαδή και επι κλίνης και μιας εκπαίδευσης κάπου αλού, δηλαδή, αν πάω εγω που είμαι ουρανοκατέβατος και είμαι σε άλλο τμήμα, εγω είμαι στην εντατική, ή εκτος και αν το πρόγραμμα θα γίνεται μονο για τα άτομα της εντατικής

**R: Μόνο για τα ατομα της εντατικής**

S:… ναι αλλα ξέρετε εμεις στην εντατική μας δεν έχουμε και πολύ εμπειρία με δισωληνομένους ασθενείς,άρα μιλούμε με άτομα,για διασωληνομένους ασθενείς,αρά το πρόγραμμα πρέπει να ξέρεις σε ποιους απευθύνεται, αρα όπως το σκέφτομαι εγώ θα απευθυνεται σε άτομα που έχουν εμπειρία με τους διασωληνομένους αρρώστους και έργάζονται τωρά σε τμήμα με διασωληνομένους ασθενείς,οκ, ένα μεικτό πρόγρμμα για να εν καλό,δηλαδή μια εκπαίδευση κάπου αλλού ή σε ένα αμφιθέατρο ή κάτι και μια επι κλίνης εκπαίδευση και πρακτική,τωρα ένα μ πεις γιατι εν μπορούμε να το πούμε μπροστά στον αρρωστο, ναι αλλα εν λιο δύσκολο να τα πεις ούλλα που πάνω π τον αρρωστο,νομίζω καθήμενος σε ένα ωραιο χωρο και να πεις διαφορα πραματα,για παραδειγμα τι εμποδίζει την επικοινωνία, τι εν εκινο που πιστευετε ότι εμποδίζει την επικοινωνια, μια ωραία βιβλιογραφεία,που να λεει συμφωνα με την βιβλιογραφεια τουτο τουτο και τουτο, εχετε εσεις κατι αναλογο όπως κατι σαν συνεντευξη να το δούμε και μετά, μια θεωριτική ας το πούμε κατάρτιση και μετά μια κατάρτιση επι κλίνης, ή πως ένα καταλαβαιτε ότι ενας άρρωστος εε θέλει να σας μιλήσει για κάτι ασπούμε,εεμμ τούτο που με ρώτησες πριν, θα ηταν ωραία να τα ακούσεις ετσι μέσα στο αμφιθέατρο και μετα να πανε επι κλίνης, νομίζω έτσι θα ηταν καλό το πρόγραμμα

**R: …. EYXAΡΙΣΤΩ…**

ΝΟ 3

R:Recearcher

C: constantinos

R: constantinos

C: Παρακαλώ

R: Όπως σου είχα πει και από το τηλέφωνο, κάμνω μια έρευνα που αφορά τις εμπειρίες επικοινωνίας των νοσηλευτών με διασωληνομένους ασθενείς στην ΜΕΘ. Λοιπόν, όπως ξέρεις δεν έχει σωστό και λάθος, ότι μ πεις, όσα πιο πολλά μου πέις τόσο το καλύτερο για εμένα, προσπαθώ να δώ πολλές οπτικές γωνίες που νοσηλευτές, δηλαδή πως το βλέπουν , τι εμπόδια βρίσκουν, πως αντιλαμβάνονται τούτη την εμπειιρία όλη για να φκάλω και εγω κάποια συμπεράσματα. Το πρώτο πράμα που ήθελα να σε ρωτήσω, είναι πόσο χρονων είσαι; Να κάνουμε έτσι λίγο διαδικαστικά ..

C: 31

R: Που σπούδασες;

C: Αγγλία

R: Που συγκεκριμένα;

C: Στο πανεπιστήμιο του ΣΑΛΦΟΛΤ, Manchester

R: OK, έχεις κάνει κάπου μεταπτυχιακό;

Oχι, εχω ειδίκευση στις πληγές, tissue viability, αλλά δεν θεωρείται μεταπτυχιακό, είναι ενδονοσοκομειακή εκπαίδευση

R: Mάλιστα, πόσα χρόνια εργάζεσαι ως νοσηλευτής;

C:8

R: Πολύ ωραία και πόσα χρόνια είσαι στις μονάδες;

C: 2,5

R: 2,5 χρόνια ,τέλεια, το πρώτο πράγμα που θέλω να σε ρωτήσω είναι, σε ποιες περιπτώσεις στην μονάδα σου μπορούμε να βρούμε ένα διασωληνωμένο ξύπνιο ασθενή;

C:Δηλαδή;

R: Έναν ασθενή που να έχει τον σωλήνα και να είναι ξύπνιος, υπάρχει περίπτωση να έβρουμε κάποιο έτσι ασθενή, σε ποια φάση θα εβρω έτσι ασθενή;

C:Yπάρχει, την φάση που προσπαθείς να αποσωληνώσεις τον ασθενή που τον αναπνευστήρα, πάντα ο ασθενής, σβήνεις τις καταστολές, περιμένεις τον να ξυπνήσεις να επικοινωνήσει , να έχεις ένα κάποιο τρόπο να τον αξιολογήσεις για να μπορέσεις να κάμεις την μετάβαση στο extubation.

R: OK, τι περιστατικά δέχεστε στην μονάδα σας;

C: Ως επι το πλείστων καρδιολογικά και μερικά με καρκινοπαθείς, πολλά λίγα εν τα περιστατικά που έχουν να κάμουν με άλλα θέματα

R: Είδες κάποια άλλη φορά κάποιον ασθενή να είναι διασωληνομένος και ξύπνιος εχτός που τούτη την φάση που μου λέεις που προσπαθείτε να τον αποσωληνωσετε; … αν υπάρχει

C:Εεε όχι.. Εν μπορώ να θυμηθώ περιστατικό που να ήταν ξύπνιος ασθενής χωρίς να υπάρχει άλλος λόγος , ενοω που το να προσπαθείς να τον κάνεις extubation

R : Τούτη είναι η λεγόμενη φάση του weaning αν λεω σωστά;

C: Nαι κάμνεις wean off

R: Eμμ οκ, μπόρεις να μου περιγράψεις την εμπειρία σου γενικά προσπαθώντας να μιλήσεις με τούτους τους ασθενείς την ώρα που είναι ξύπνιοι και προσπαθείς να τους αποσοληνώσεις; Aν σου έρχεται κάποια συγκεκριμένη εμπειρία στο μυαλό, περίγραψε μου την

C: Εμμ να σου πω, αρχικά ήταν πολλά περίεργο, να βλέπεις έναν άνθρωπο με ένα σωλήνα μέσα στο στόμα και να προσπαθείς να επικοινωνήσεις εν πολλά περίεργο, εν μια κατάσταση όμως η οποία συνηθίζεται, δηλαδή ξέρεις ότι υπάρχουν κάποια βήματα αξιολόγησης, προσπαθείς να επικοινωνήσεις με τον ασθενή να δεις κατά πόσο νευρολογικά εν καλά, ανταποκρίνεται , ακολουθεί κάποιες εντολές χωρίς να υπάρχει το κομμάτι της ομιλίας ,το δύσκολο κομμάτι εν το κομμάτι του καθησυχασμού του ασθενή, δηλαδή να τον έχεις καθησυχασμένο ότι εν εντάξει εκείνο που έχεις μέσα στο στόμα σου, βοηθά σε να αναπνεύσεις εν για το καλό σου, χρειάζεται κάποιος χρόνος ούτως ώστε να μπορέσουμε να βγάλουμε τον σωλήνα και να μπορείς να αναπνέεις μόνος σου, εε θέλει πολύ υπομονή.

R: Yπομονή, γιατί ;

C: Eεε διότι ο άλλος αντιλαμβάνεσαι με ένα σωληνά μεσα στο στόμα, ξεκινούν να δουλεύουν τα αντανκλαστικά,μπορεί να σου έρχεται αναγούλα, ο άλλος εν ανυπόμονος,μπορεί πολλές φορές να πιάσει τον σωλήνα, αν νευρολογικά ενεν ετυπος,είναι επικύνδυνο για τον ίδιο γιατι δεν έκτελει οδηγίες, η μόνη του έννοια είναι τραβήσει εκείνο που έχει μέσα στο στόμα του για να μπορέσει να φκάλει το εμπόδιο όπως το νιώθει ο ασθενής, άρα θέλεις πολλή υπομονή για να το αντέξεις τουτο το κομμάτι ώσπου να είναι καλά ο ασθενής σου..

R: Οκ, είπες μου πάρα πολλά ωραίες πληροφορίες τωρά, να πιαστώ έτσι που μερικές, εμμ, τι κάμνεις ακριβώς για να καταφέρεις να επικοινωνήσεις, πως κατ.. , μάλλον πως καταφέρνεις ν δεις ποιες είναι οι ανάγκες του ή τι θέλει να σου πει εκείνη την ώρα; Μπορείς να μου δώσεις έτσι μερικά παραδείγματα;

C: To τι θέλει να σου πει ακριβώς δεν μπορείς να το καταλάβεις, προσπαθείς μέσω των κινήσεων που κάνει με τα χέρια του και το κεφάλι του να καταλάβεις τι γίνεται και ως επι το πλείστον πρέπει να του κάμεις ερωτήσεις και οι ερωτήσεις είναι ας πούμε εε πονείς; Ενοχλεί σε κάτι; αναπνέεις καλα; Νιώθεις το στόμα σου ξηρό; Συνήθως έχουν και την αίσθηση ότι θέλουν νερό, έχουν πολύ ξηροστομία διότι ο σωλήνας εν μέσα στο στόμα αρκετές ώρες,μπορεί και μέρες σε αυτούς τους ασθενείς, αρα προσπαθείς μέσω ερωτήσεων να περισυλλέξεις οσες παραπάνω πληροφορίες γίνεται με το να τον ρωτάς πράγματα διοτι που την στιγμή που δεν υπάρχει ο λόγος οι κινήσεις που μπόρει να κάμει είναι να σου δήξει κατι αν είναι νευρολογικά καλός αλλα πέραν τούτου δεν μπορεί να κάμει κάτι παραπάνω για να καταλάβεις αρα πρέπει εσυ να δώσεις τις πληροφορίες και να σου δήξει ο ασθενής με άρνηση ή κατάφαση για το τι ακριβως θέλει

R: Ok, δηλαδή με το κεφάλι του και τα χέρια του τι ακριβώς κάνει; Ειπες μου δήχνει, ασπουμε..

C: Μπορεί αν σου δήξει το στόμα του, μπορεί να σου δήξει το στήθος του, αν τον ρωτήσεις πονείς,μπορεί να σου πει ασπουμε ναι, να σου δήξει το χέρι του, να σου κάμει το κεφάλι του κάτω, αν πονεί ένα του πεις ασπούμε που πονείς, ένα σου δήξει το χέρι του που πονει, θα προσδιορίσει την περιοχή που είναι ο πόνος, αν διψά μπορεί να σου δήξει το στόμα του που μόνος του ή μπορεί να σου δήξει ότι ενοχλεί τον ο σωλήνας με το δάχτυλο του, ότι ενοχλεί σε κάτι; Ναι.. τι σε ενοχλεί, ο σωλήνας, δήχνει σου τον σωλήνα με το δάχτυλο… παίρνεις μια καθοδήγηση.

R: ok, μάλιστα, τι πιστεύεις είναι οι ανάγκες των ασθενών εκείνη την ώρα, τι χρειάζονται περισσότερο, τι σου ζητούν;

C: Ως επι το πλείστο εφησυχασμό, τζίνο που χρειάζονται παραπάνω είναι εφησυχασμός για την κατάσταση διότι ξυπνούν από μια καταστολή και κάπως συγχυσμένοι, εν αποπροσανατολισμένοι, αρα θέλουν μια καθοδήγηση και το ότι ναι είσαι καλά μεν ανησυχείς εν μέρος της διαδικασίας και ένα περάσει σε κάποια λεπτά όταν ένα μπορέσουμε να φκαλουμε τον σωλήνα, πέρα που τούτον των αναγκών εν έχουν κάποια ιδιαίτερη ανάγκη εκείνη την στιγμή για το συγκεκριμένο κομμάτι, τώρα αν υπάρχουν άλλα παράπλευρα προβλήματα εν άλλο θέμα αλλά δεν αφορά την διαδικασία της αποσωλήνωσης

R: Αρα εσυ εντοπίζεις τούτο το πρόβλημα παραπάνω για τους ασθενείς,τούτες τις ανάγκες, να τους καθησυχάσεις..

C: Ναι ναι, αν έχει αιμοδυναμική αστάθεια ή οτιδήποτε άλλο, δεν έχει να κάμει με τον ασθενή , ο ασθενής δεν το ξέρει, εν κάτι που το ξέρουμε μόνο εμείς και διαχειριζούμαστε το χωρίς να το μεταφέρουμε στον ασθενή, εν θέμα δικό μας

R: Μάλιστα, οκ, τι άλλο μπορώ να σε ρωτήσω.. εε τι εμπόδια αντιμετωπίζεις κατά την διάρκεια που προσπαθείς να επικοινωνήσεις με τον ασθενή, δηλαδή ασπούμε μπορείς να θυμηθείς κάποιο συγκεκριμένο περιστατικό που προσπαθούσες να επικοινωνήσεις και δεν τα εκατάφερνες μαζί του;

C: Έχουμε αρκετά περιστατικά που οι ασθενείς ξυπνούν και εν συγχυσμένοι και δεν ακολουθούν καθόλου τις εντολές και τις οδηγίες απλά το μόνο που κάμνουν είναι να προσπαθούν να τραβήσουν τον σωλήνα και δεν υπάρχει καμία επικοινωνία, δεν σε ακούει, δεν ακολουθά καθόλου τις εντολές σου, αρα τζιαμέ πραγματικά δεν μπορείς να κάμεις κάτι εχτός που να τον περιμένεις να μπορέσει να επανέλθει νευρολογικά, να μπορέσει να επανέλθει το κομμάτι της κατανόησης, του λόγου, του τι του λαλέις, να μπορέσει ο εγκέφαλος του να μεταφράσει εκείνο που το λαλείς σε εκείνο που πρέπει να καταλάβει για να μπορέσεις να προχωρήσεις αλλίως δεν μπορείς να κάμεις κάτι, είναι απλά.. περιμένεις

R: ok, θυμάσε κάποιο περιστατικό που σου έμεινε στον νου για οποιοδήποτε λόγο που προσπαθούσες να επικοινωνήσεις γιατι δεν τα κατάφερνες, ή εκατάφερες τα, κάτι που να σου έμεινε στον νου και να μ το περιγράψεις; Ή κάποιο πρόσφατο περιστατικό για παράδειγμα.

C: Εεε είχαμε ένα περιστατικό που ήταν περίεργο με την έκβαση της όλης κατάστασης, ένας ασθενής ο οποίος έκανε χειρουργείο καρδίας , ήταν πλήρως επικοινωνιακός νευρολογικά, εμπορούσαμε να συνεχίσουμε και στην διαδικασία της αποσωληνωσης αλλά λόγω του ότι είχε πολύ αιμοδυναμική αστάθεια και ήταν υποθερμικός παρόλο που επικοινωνούσε πλήρως ο άνθρωπος και είμασταν έτοιμοι , αποφασίσαμε να τον βάλουμε σε καταστολή και ως το πρωί έπαθε εγκεφαλικό, χωρίς να αναγνωριστούν τα συμπτώματα, διότι επήγε πίσω σε καταστολή. Τζίνο ηταν πολλά περίεργο και το πρωί που προσπαθείς να τον ξυπνήσεις ο άνθρωπός ξαφνικά δεν μπορεί να κινήσει την μισή του την πλευρά αρα εν μια εμπειρία η οποία σου μένει.

R: Mάλιστα, άλλο που ήθελα να σε ρωτήσω, είπες μου πριν αμαν οι ασθενείς ξυπνούν προσπαθούν να τραβήσουν τον σωλήνα εν ανήσυχοι και ότι εν μπορούν, εσυ εκείνη την ώρα πως νιώθεις όταν προσπαθείς να επικοινωνήσεις και δεν τα καταφέρνεις

C: Πως νίωθω..

R: Αν νίωθεις κάτι

C: Να σου πω ,πλέον εν μέρος της διαδικασίας, εν μου προκαλεί κάποια ιδιαίτερά αισθήματα τούτο το πράμα, ξέρω ότι εν μέρος της διαδικασίας και πρέπει να κάμω υπομονή, τίποτε άλλο, εντάξει κάποιες φορές, όταν τούτη η διαδικασία γίνεται έτσι πολλά αργή και βλέπεις ότι ο ασθενής έχει πολύ δρόμο μπροστά του, μπορεί σε κάποιες φάσεις να νιώθεις ότι λίφκει η υπομονή σου, ότι γίνεσαι λίγο ανυπόμονος, ότι ατε να τελειώνουμε, αλλά εν μπορείς να κάμεις κάτι παραπάνω , παρά το να περιμένεις

R: Ok, παρατήρησες άλλα εμπόδια που σε αφήνουν πίσω στο να επικοινωνήσεις με τούτο τον ασθενή; Ειπες μου για παράδειγμα τον σωλήνα, που εν το πιο σημαντικό, που εν στο στόμα του και δεν μπορεί να μιλήσει ή αμαν εν συγχυσμένος, φαντάζομαι που την καταστολή που ακόμα να φέρει καλά τον νού του, εχει οτιδήποτε άλλο που πρόσεξες στην μονάδα που σε εμποδίζει να επικοινωνήσεις;

C:Oι εξωτερικά εμπόδια δεν υπάρχουν στο θέμα της επικοινωνίας, εν καθαρά το νευρολογικό κομμάτι, τα εμπόδια τα άλλα που υπάρχουν εν η νευρολογική κατάσταση του ασθενή, το αναπνευστικό κομμάτι, αν μπορεί να διαχειριστεί αναπνοές που μόνος του, μπορεί να εν ξύπνιος ο ασθενής αλλά το αναπνευστικό σύστημα να μην λειτουργεί πλήρως άρα να έχει ανάγκη τον αναπνευστήρα άρα να μην μπορείς να φκάλεις τον σωλήνα παρόλο που νευρολογικά κάπου προχώρησε ο ασθενής

R: ok, κάτι άλλο που μπορείς να σκεφτείς ας πούμε; Εγώ μιλώ κατά την διάρκεια που ο ασθενείς εν διασωληνομένος και προσπαθείς να επικοινωνήσεις, πριν να βγάλεις τον σωλήνα, πρόσεξες κάτι άλλο το οποίο σε δυσκολεύει να επικοινωνήσεις μαζί του; Ειπες μου το αυτονόητο που εν ο σωλήνας για πάραδειγμα.. ή νευρολογική κατάσταση

C:Οι οι οι, εν έχει κάτι άλλο, για έμενα εν τούτα, εν καθαρά τούτα, εν είχα οποιδήποτε πρόβλημα άλλο εκτος που τούτα.

R: ok εμμ

C:Μπορεί να εν ούλλα καλά τα περιστατικά μου, δεν ξέρω,

R: οι οι έχω και άλλους που μου είπαν το ίδιο πράγμα ασπούμε,

C:Χαχαχαχα

R: εχω και άλλους που μου είπαν διάφορα άλλα, εξαρτάτε που τον καθένα και που δουλεύει φαντάζομαι, τι περιστατικά και πόσα αναλαμβάνει, το άλλο που ήθελα να σε ρωτήσω, έχετε μέσα επικοινωνιας στο τμήμα σας, δηλαδή ασπούμε, εχρησιμοποίήσετε ποτέ, το πιο απλό ας πούμε, πένα και χαρτί, ένα άλλο παράδειγμα εν ενας πίνακας με σχήματα που μπορεί να σου δήξει ο ασθενής, απλά σχήματα, ας πούμε ότι πονώ πεινώ, δίψω ή ταμπλετ να εν κάτι πιο εξελιγμένο

C:Το μόνο που χρησιμοποιήσαμε για ασθενείς οι οποίοι ηταν στον αναπνευστήρα για αρκετό διάστημα και τούτο έχει να κάμει με ασθενείς που έχουν τραχειοσωλήνες (***τραχειοστομίες ήθελε να πει)*** οι με διασωληνομένους με σωλήνες, εχρησιμοποιούσαμε την κόλλα και την πέννα να μας γράψει ένα μαρκαδόρο πάνω στην κόλλα εκεινο που θέλει για να μπροούμε να επικοινωνούμε

R: ok, με ασθενεις που έχουν σωλήνα δηλαδη εν δύσκολο να γινεί τούτο το πράμα, εν το κάμνετε;

C:Συνήθως η διαδικασία αμαν έχουν τον σωλήνα εν πολλά πιο μικρή σε διάρκεια και η επικοινωνία εν σχεδόν άμμεση, αμμεση με την έννοια της μισής μιας ώρας.. σύνηθως, εν θελει πολλά παραπάνω που τζίνο σε θέμα επικοινωνίας και εκεινη την ώρα ο ασθενής εν σε μια φάση μέθης.. μπορώ να το περιγράψω, άρα το να του δώσεις κόλλα και πέννα να γραψει ή ένα τάμπλετ εν σχεδόν αδύνατο, απλά εκείνο που θέλεις είναι να εκτελέσει βασικές οδηγίες οι να μπορέσει να γράψει να κάμει οτιδήποτε που τούτα

R: Πολλά ωραία, αρα από ότι κατάλαβα δεν εε, υπάρχει κάτι το οποίο εδώσετε στους ασθενείς με τραχειοστομία; Που μου είπες, οι τάμπλετ. κολλα και πένα είπες μου συγνώμη

C:Ναι ,μαρκαδόρο και πέννα

R: Eν υπάρχει κάτι άλλο εχτος που μαρκαδόρο και πέννα , σε θέμα επικοινωνίας με τέτοιους ασθενείς;

C:Eεε εν εχρειάστηκε να χρησιμοποιήσουμε κάτι άλλο, τουλάχιστο στα περιστατικά που είχα εγώ , το μόνο που μπορώ να θυμηθώ εν τούτο, να έχουμε ασθενή που εν με τραχειοστομία στον αναπνευστήρα και λογω του ότι δεν μπορεί να επικοινωνήσει και να μας πει εκέινα που θέλει να του δώσουμε κόλλα και πέννα να μας γράφει

R: Ok, αν σου δίναν την ευκαιρία να εκπαιδευτείς σε τούτο τον τομέα δηλαδή σε θέματα επικοινωνίας με τέτοιους ασθενείς που δυσκολεύονται που έχουν τον σωλήνα στο στόμα τους, που δυσκολευονται να επικοινωνήσουν, θα ήθελες να το κάμεις;

C:Ναι γιατι οχι; Ότι πάρει εν κέρδος ενεν;

R: Σωστά, αν είχες την ευκαιρία να διαλέξεις με πιο τρόπο θα εδιδάσκεσουν , θα προτιμούσες να είσουν δίπλα που τον ασθενή για παράδειγμα, να τα κάμεις σε μια αίθουσα με project ,να τα κάνεις διαδυχτιακά; Πως θα προτιμούσες να το κάνεις; Τι πιστευεις θα σε ωφηλούσε εσένα;

C:Δυο πράγματα μαζί, εεε το αρχικό πλάνο να γίνει σε μια αίθουσα και η δευτερη φάση να γίνει πρακτικά με τον ασθενή. Δεν θέλω να αφήσω το πρακτικό κομμάτι έξω, αλλα νομίζω εν πιο σωστό πρώτα η βασική εκπαιδευση να γίνεται με κάποιον τρόπο σε κάποια αίθουσα ούτως ώστε να μπόρεις να λύσεις τις απορίες σου πριν να φτάσεις στον ασθενή και μετέπειτα να έχεις μια καθοδήγηση τζιαμέ που εν ο ασθενής μαζί με ένα άτομο που θα σε εκπαιδεύει στα αρχικά στάδια ώσπου να υλοποιήσεις την εκπαίδευση σου

R: Mάλιστα, νομίζω έλυσες μου όλες τις απορίες μου, εμμ δεν ξέρω, θέλεις να προσθέσεις εσύ κάτι που σου έρχεται στον νου, μια συγκεκριμένη σου εμπειρία

C:Το μόνο που είχαμε, που θυμήθηκα, είχαμε ένα ασθενή ο οποίος ηταν τυφλός και πολύ βαρύκουος

R: Πολλά θέλω να το ακουσώ τούτο..

C:Χαχα Εν μπορώ να το περιγράψω

R: Eίχες τον ποτέ εσύ τούτο τον ασθενή; Ήσουν ο νοσηλευτής του;

C:Ναι είχα τον, εεε ήταν πολλά δύσκολο το κομμάτι της επικοινωνίας, πραγματικά ,δίοτι να σε ακούσει σχεδόν δεν εμπορούσε καθόλου, να σε δεί ,να δει τις κινήσεις σου δεν εμπορούσε και έπρεπε να έβρεις κάποιο τρόπο να επικοινωνήσεις και ο μόνος τρόπος ήταν με το άγγιγμα στην ουσία..

R: Mάλιστα και;

C:Kαι έπρεπε να κάνεις την κίνηση να του πίασεις το χέρι του να του το πάρεις πάνω, να του δήξεις ότι σφίγγεις το για να μιμηθεί την κίνηση που κάμνεις εσύ ουσιαστικά.

R: Τούτος ο ασθενής πως επικοινωνούσε μαζί σου; Αφού δεν εμπορούσε να μιλήσει

C:Ούτε να μιλήσει εμπορουσε, ούτε να δει εμπορούσε και το να ακούσει ήταν πολλά λίγο. Αρα ο μόνος τρόπος που μπορούσε να επικοινωνήσει ηταν με τις κινήσεις των άκρων του, τιποτε άλλο

R: Δηλαδή τι σας έκαμνε ακριβώς; Περίγραψε μου έτσι λίγο..

C:Επροσπάθαν να μας δήξει πράγματα με τα χέρια του, δηλαδή, να πάρει το χέρι στο στόμα, να πάρει το χέρι στην κοιλιά να πάρει το χέρι στο στήθος, να μας χτυπήσει πάνω του εκει που επονούσε, σιγά σιγά εκαταλαμβαίναμε τι ενοούσε, δηλαδή αν εχτύπαν σε ένα σήμειο, ξέραμε ότι πονούσε

R: Αρα κωδικοποιούσατε μια πληροφορία που σας έδινε

C:Ναι μπράβο.. Αν έβαλε το δάχτυλο του μέσα στο στόμα του, καταλαβαίναμε ότι ήθελε νερό

R: Tουτο εμεταφέρετε το που τον ένα νοσηλευτη στον αλλο;

C:Nαι ναι , εμεταφέρνετουν η πληροφορία στον επόμενο που ηταν να αναλάβει, ηταν να του πεις ξέρεις αν σου καμει ετσι, σημαινει έτσι κλπ για να μπορεί και ο άλλος να καταλάβει τι γίνεται, ηταν ένα από τα πιο δύσκολο περιστατικά που είχαμε

R: Τούτος ο κυριος ηταν πολύ καιρο διασωληνομένος και ξύπνιος;

C:Ηταν για κάποιες μέρες

R: Hταν από τα μόνα περιστατικά που ηταν τόσο χρόνο;

C:Hταν από τα πολλά σπάνια περιστατικά και σε χρόνο και να έχει και τούτα τα προβλήματα επικοινωνίας σε θέμα όρασης και ακοής…

R: Mάλιστα , με τους άλλους ασθενείς έτυχε να κωδικοποιάτε έτσι πληροφορίες ή εν χρειάστηκε;

C:Oι ιδιαίτερα, διότι που την στιγμή που σε βλέπει και ακούει σε ο άλλος αντιλαμβάνεται το μήνυμα που περνάς τις πλείστες φορές.

Νο 4

R:Researcher

A: Achilleas

**R: Achilleas καλημέρα, όπως σου έχω πει και από το τηλέφωνο κάνω μια έρευνα που αφορά τις εμπειρίες των νοσηλευτών που δουλεύουν σε ΜΕΘ όσο αφορά τους διασωληνομένους αλλά ξύπνιους ασθενείς, να σου κάμω μερικές ερωτήσεις, βασικά εν μια συζήτηση που θα κάνουμε, όπως ξέρεις δεν έχει σωστό και λάθος, θέλω να πάρω εμπειρίες που διάφορους νοσηλευτές, που διάφορα επίπεδα, εννοώ που μικρούς μεγάλους, γυναίκες άντρες κλπ για να δώ έτσι τις απόψεις σας, όσο πιο πολλά μου πείς τόσο το καλύτερο και απλά για να ξέρεις τυπικά σου λέω τώρα ότι σε αυτές τις πληροφορίες έχω μόνο εγώ πρόσβαση και η καθηγήτρια μου και θα είναι με ψευδώνυμα όταν παρουσιαστεί τούτο το πράμα, δεν θα αναφερθεί κάπου το όνομα σου.. το πρώτο που θα ήθελα να σε ρωτήσω είναι έτσι γενικές ερωτήσεις. Καταρχάς πόσο χρονών είσαι;**

A: 26

**R: 26.. που σπούδασες αν επιτρέπετε;**

A: Στο ΤΕΠΑΚ

**R: Στο ΤΕΠΑΚ, έκαμες κάποια εκπαίδευση μετά περετέρω ασπούμε, έκαμες κάποιο μεταπτυχιακό ή οτιδήποτε;**

A: Oχι

**R: Οκ, πόσο καιρό έχει που εργάζεσαι γενικά ως νοσηλευτής;**

A:2 χρόνια

**R: 2 χρόνια, στην Μονάδα πόσο καιρό έχει που εργάζεσαι;**

A:2 χρόνια

**R: Άρα καθαρά ξεκίνησες που την Μονάδα; Εντάξει οκ, εμμ, πως είναι γενικά να εργάζεσαι με…, μάλλον να σε ρωτήσω κάτι άλλο, στην μονάδα σας σε ποιες περιπτώσεις βλέπουμε ένα ασθενή να είναι διασωληνομένος και ξύπνιος; Όχι τραχειοστομημένος, δηλαδή εσύ σε ποια περιστατικά είδες αυτό το πράμα;**

A:Κατά την διάρκεια που προχωρήσουμε τον άρρωστο για μια αποσωλήνοση

**R: Οκ, άρα δεν είδες κάποια άλλη περίπτωση να είναι κάποιος διασωληνομένος και ξύπνιος;**

A: Δεν έχει κάποιο λόγο, κατά την άποψη μου να είναι κάποιος ξύπνιος με τον σωλήνα μέσα στο στόμα.

**R: Mάλιστα, πόση ώρα περίπου παίρνει τούτη η διαδικασία;**

A: Εξαρτάτε..

**R: Πάνω κάτω..**

A: Έχει άρρωστους που θα πάρει και μέρες έχει άρρωστους που θα πάρει και ώρες. Δεν είναι κάτι το συγκεκριμένο, εξαρτάτε που την γενική κατάσταση του αρρώστου, που τα φάρμακα που πείρε ή που οποιοδήποτε άλλο παράγοντα που μπορεί να τον επηρεάσει, ενεν κάτι συγκεκριμένο.

**R: Οκ, γενικά που την εμπειρία σου έτσι μια γενική εικόνα τωρά, πως είναι να προσπαθείς να επικοινωνείς με τούτους τους ασθενείς κατά την διάρκεια που είναι ξύπνιοι και διασωληνομένοι;**

A: Δύσκολή η επικοινωνία αφού ο άρρωστος δεν μπορεί να σου μιλήσει, οτιδήποτε θα σου δήξει. Ένας άρρωστος που δυσανασχετά που έχει ένα σωλήνα μέσα στο στόμα τον οποίο δυσκολεύει τον στο να επικοινωνήσει στο να σου πει πολλές φορές θέλει να πιεί νερό ή να θέλει να αναπνεύσει κατά την άποψη του πιο εύκολα, είναι δύσκολο πράμα για τον άρρωστο και δύσκολο και για εμάς που προσπαθούμε να επικοινωνήσουμε μαζί του, αρκετά άβολο.

**R:Οκ, έχεις κάποια συγκεκριμένη εμπειρία που σου έρχεται στον νου τωρά με κάποιο ασθενή να μου την περιγράψεις, απλά να μου περιγράψεις κάτι που σου έμεινε στον νου οτιδήποτε;**

A:Δεν μου έρχεται κάτι συγκεκριμένο, είναι όλες παρόμοιες εμπειρίες, απλά βλέπεις ένα άρρωστο που δυσανασχετά κάτι που τον δυσκολεύει και εκείνο και εμάς αλλά δεν χρήζει τα δεδομένα για να μπορεί να γίνει αποδιασωλήνωση, πρέπει να περάσει που εκείνη την κατάσταση που εν δύσκολη για τον ίδιο και για εμάς

**R:Οκ, πως καταφέρνεις να επικοινωνήσεις με τούτο τον ασθενή;**

A: Εεεε..

**R: Με ποιους τρόπους, περίγραψε μου τους, όσο μπορείς.**

A:Εμείς ένα μιλήσουμε του ασθενή, αφού μπορούμε να επικοινωνήσουμε μαζί του, εφόσον εν φουλ ξύπνιος και δεν έχει οποιοδήποτε πρόβλημα με την επικοινωνία ο ίδιος, ο ίδιος προσπαθεί με νοήματα, με τα μάτια με τα χέρια που εννοείται εν δύσκολο, έχει αρρώστους που μπορούμε να τους καταλάβουμε εύκολα, έχει που δεν θα καταφέρουμε καν να καταλάβουμε τι εν τζίνο που προσπαθούσε να μας πει.

**R:Ωραία άρα είπες μου τώρα μέχρι στιγμής ότι επικοινωνά ο ίδιος ο ασθενής με νοήματα και συγνώμη με, είπες μου με νοήματα**

A:Με τα μάτια, ένα προσπαθήσει να μας δήξει κάτι

**R:Με τα μάτια ή ένα σου δήξει κάτι, εσύ έχεις κάποιους τρόπους που κατάφερες με τον καιρό να επικοινωνάς λίγο καλύτερα ή κάποιες συγκεκριμένες στρατηγικές ασπούμε, τι ερωτήσεις του κάνεις για να δεις τι θέλει ασπούμε;**

A:Εεεε πολλές φορές, βασικά ρωτούμε τους τα βασικά που τους ξέρουμε ότι τους ενοχλούν, ξέρουμε ότι τους ενοχλά ο σωλήνας που έχουν στο στόμα, πολλές φορές μπορεί να έχουν αναγούλα, πολλές φορές να διψούν, εν τα συνηθισμένα τα πράματα που μας ΄΄ρωτούν΄΄ ή και εννοείται πάντα ένα τους ρωτήσουμε για πόνο, επειδή μπορεί να έχει πόνο σε ένα σημείο και να μεν μπορεί, τούτα εν τα .., απλά εν και έχει κάποιο συγκεκριμένο τρόπο να επικοινωνήσουμε μαζί τους γ αυτό προσπαθούμε να πηγαίνουμε στα πιο …

**R: Βασικά**

A:Βασικά που θέλουν συνήθως οι αρρώστοι, που ένα μας τα πουν μόλις φκάλουμε τον σωλήνα, τα πρώτα πράματα που ένα συζητήσουν, βλέπουμε εν και τα πράματα που τους ενοχλούν και κατά την διάρκεια που έχουν τον σωλήνα, γ αυτό εν τα πράματα που ένα τους ρωτήσουμε πρώτα για να μας πουν με ένα ναι, το πιο εύκολο..

**R:Οκκ ,εε με τον καιρό τι εκατάλαβες ότι εν οι ανάγκες τούτων των ασθενών, ή τι τους ενοχλεί ασπούμε, τι εν τζίνο που βλέπεις εσύ ως νοσηλευτής..**

A:Ο σωλήνας..

**R:Τους ενοχλεί ο σωλήνας, τι εν τζίνο που θέλουν να σε ρωτήσουν η να σου πουν πρώτα, ασπούμε τι είδες ότι..**

A:Εν ανησυχία, επειδή οι παραπάνω ξέρουν ότι ή θα περάσαν που κάποιο χειρουργείο ή σε άλλη περίπτωση αν έπαθαν κάποια ανακοπή ή οτιδήποτε και δεν ξέρουν γιατί εβρεθήκαν εκεί, τζίνος που πέρασε που χειρουργείο έχει έννοια κατά πόσο επέρασε το χειρουργείο του και εν καλά, τζίνος που μπορεί να έπαθε μια ανακοπή και να ξυπνά, εν θα ξέρει γιατί είναι εκεί και με εκείνον τον σωλήνα μέσα στο στόμα ,άρα οι ανησυχίες του μόλις ξυπνήσει και καταλάβει ότι έχει ένα σωλήνα μέσα στο στόμα,, που εννοείται εμείς ένα του εξηγήσουμε την κατάσταση γιατί βρίσκεται σε εκείνη την θέση και τι εν εκείνο το πράμα που έχει μέσα στο στόμα του το οποίον τον δυσκολεύει.

**R:Μάλιστα οκ, εε… τι άλλο να σε ρωτήσω, λοιπόν, πρόσεξες κατά την διάρκεια που επικοινωνείς τι εν τζίνο που σε εμποδίζει να επικοινωνήσεις με τον ασθενή; Tο πρώτο πράμα και κυρίως που είπες εν ο σωλήνας, υπάρχει οτιδήποτε άλλο εμπόδιο που σε εμποδίζει να επικοινωνήσεις με τούτο τον ασθενή, γενικά ,μπορείς να σκεφτείς;**

A:Eν μπορώ να σκεφτώ κάτι το διαφορετικό..

**R:Που τον ίδιο τον ασθενή ,που τον τόπο που δουλεύεις για παράδειγμα**

A:Οι οι

**R: Άρα καθαρά εκείνο που σε εμποδίζει να..**

A:Εν θέμα του σωλήνα και το ότι ο ασθενής που έχει τον σωλήνα δυσανασχετεί, οι πλείστε περιπτώσεις πολλά λίγοι ασθενείς εν ήρεμοι με τζίνο τον σωλήνα μέσα στο στόμα

**R:Οκ, τί κάνεις στις περιπτώσεις που ο ασθενής είναι αναστατωμένος, ασπούμε και εν δέχεται αν του εξηγήσεις ή δεν σε ακούει**

A:Εεε πάντα εννοείτε ότι

**R:Πως καταφέρνεις να επικοινωνήσεις μαζί του ή να του πεις εκείνα που θέλεις να του πεις, απλά μιλάς του, τι του κάμνεις ασπούμε;**

A:Προσπαθούμε να τον κάνουμε να καταλάβει σε κάποια βρίσκεται για να ηρεμίσει, γιατί δεν έχει κάποιο άλλο τρόπο, εννοείται είσαι κοντά του και μιλάς του για να καταλάβει την κατάσταση για να ηρεμίσει, να καταλάβει ακριβώς τι του γίνεται και να ηρεμήσει, να μεν έχει τουλάχιστο άγχος που τούτη την κατάσταση, εντάξει εννοείται έχει αρρώστους που ούτε με τούτο τον τρόπο δεν θα ηρεμίσουν

**R: Μμμ, μάλιστα, εεε , γενικά όταν δεν καταφέρνεις να επικοινωνήσεις με ένα ασθενή, πως σε κάνει να νιώθεις τούτο το πράμα ασπούμε, σκέφτου μια συγκεκριμένη περίπτωση ,όταν θέλεις να επικοινωνήσεις με κάποιο ασθενή και δεν τα καταφέρνεις γιατί είτε εκείνος εν ανήσυχος, είτε γιατί η** **κατάσταση του ασπούμε εν του επιτρέπει να καταλάβει, έχει κάποιο τρόπο ασπούμε, έχει κάτι που σε κάνει εσένα να νιώθεις για τούτο το πράμα;**

A:Δύσκολο εννοείται, εν πολλά κουραστικό, γιατί ένας άρρωστος που δυσανασχετά, θέλει να φύγει τον παράγοντα που τον κάνει να δυσανασχετά, άρα ο παράγοντας που τον κάμνει να δυσανασχετά, είπαμε τις πλείστες φορές, οι πάντα, εν ο σωλήνας, άρα εν ένα πράμα που χρειάζεται να αναπνέει και συνήθως αμαν εν σε τζίνο το σημείο προσπαθεί να το φκάλει, άρα εννοείται ότι εν μια δύσκολη κατάσταση και για εμάς, εν ένα βάρος ασπούμε, έχεις εκτός που την υπόλοιπη κατάσταση του ασθενή να προσέχεις να μεν τον φκάλει ή οτιδήποτε, αρά και για εμάς εν και κουραστικό και ένα βάρος ασπούμε.. παραπάνω

**R:Οκ θέλω να ξαναστραφώ λίγο σε εκείνα που είπαμε πριν, είπες μου ότι ο ασθενής προσπαθεί με νοήματα ή με τα χέρια του ή να σου δήξει κλπ, εσύ είπες μου ότι τον ρωτάς ερωτήσεις ,ασπούμε είπες μου ρωτάς τον τα βασικά πράματα ,υπάρχει κάποιος άλλος τρόπος που χρησιμοποίησες μέχρι τώρα ή που έκαμες για να καταφέρεις να επικοινωνήσεις μαζί του ασπούμε, για παράδειγμα ασπούμε, χρησιμοποιάτε όπως κόλλα και πέννα ή πίνακες ΄**

A:Έχει που μας ζητούν ναι ναι

**R:Καταφέρνει ένας ασθενής τέτοιος να επικοινωνήσει**

A:Προσπαθεί, εφόσον εν έχει κάποιο άλλο θέμα ,έχει πολλές φορές που μπορεί να προτιμήσουν να γράψουν με κόλλα και με πέννα ,για να γράψουν ακριβώς εκείνο που θέλουν

**R:Οκ και τι μου είπες ότι σας ζητούν τις περισσότερες φορές μόλις ξυπνήσουν**

A:Εεε είπα σου, σε ποια κατάσταση βρίσκονται, πολλές φορές νερό , αναγούλα, πόνος που εν κάτι γενικό, εντάξει εν φυσιολογικό, οποιοσδήποτε ασθενής να έχει πόνο , απλά αν δεν μπορούν να μας το πουν , ένα προσπαθήσουν να μας το δείξουν και ότι δυσανασχετούν με τον σωλήνα

**R:Οκ, εε εντάξει, κάτι άλλο εκτός από την κόλλα και πέννα, ασπούμε χρησιμοποιήσετε ποτέ τάμπλετ για παράδειγμα ή τηλέφωνο ή οτιδήποτε**

A:Οχιι

**R:Εντάξει οκ, με τους συγγενείς γενικά των ασθενών , τί γινεται ,μπορούν να σας βοηθήσουν να επικοινων.. καταλαβαίνουν τι θέλουν, είναι στην φάση καταρχάς που …**

A:Οι, μόνο σε περιπτώσεις που εν ξενόγλωσοι οι ασθενείς και δεν καταλαβαίνουν ούτε ελληνικά ούτε αγγλικά μπορεί να μας βοηθήσει κάποιος συγγενής, κατά τα αλλα ενμμ, τουλάχιστο εν είχα εγώ κάποια εμπειρία που να μας βοήθησε κάποιος συγγενείς με κάποια έτσι περίπτωση

**R:Οκ, γενικά τωρά που μιλούμε, είρθε σου στον νου κάτι ,κάποιαα συγκεκριμένη εμπειρία με κάποιον συγκεκριμένο ασθενή ασπούμε , έτσι απλά για να μου την περιγράψεις , εννοώ οι κάτι ξεχωριστό απαραίτητα, κάτι το οποίο μπορεί να σου έμεινε στον νου, με ένα ασθενή ο οποίος σε δυσκόλεψε ή σε έκαμε να καταλάβεις κάτι διαφορετικό ή που σε βοήθησε με τους επόμενους ασθενείς ασπούμε ή κάτι δύσκολο, για να μου περιγράψεις γενικά πως ήταν**

A:Δεν ξέρω, νομίζω όλες μου οι εμπειρίες ήταν παρόμοιες, δεν βρίσκω κάτι ξεχωριστό σε κάποια, εντάξει εν διαφορετικές περιπτώσεις που διήρκησε μέρες τούτο το πράμα

**R:Όταν εδιη.. σορρυ ναι. Όταν δηλαδή το περιστατικό που μου είπες διήρκησε μέρες, ερωτούσετε δηλαδή και επικονωνούσετε κάθε μέρα με τον ίδιο τρόπο ασπούμε, ερωτούσετε τα ίδια πράματα κάθε μέρα, ο ασθενής..**

A:Εννοείται ότι για να διάρκεσε λίγες μέρες ότι εν ηταν.. φουλ η επικοινωνία με τον άρρωστο για να μπορούμε να είμαστε σίγουροι για να προχωρήσουμε στην διαδικασία να γίνει αποδιασωλήνωση , έχει περιπτώσεις που ήταν ξύπνιος ο άρρωστος αλλά εν είχαμε επικοινωνία, εν είχε φουλ ξυπνήσει ,να μπορεί να επικοινωνεί φουλ και να είμαστε σίγουροι

**R:Οκ, άρα ήταν ένα εμπόδιο και τούτο, η νευρολογική του κατάσταση δεν σας επέτρεπε να επικοινωνήσετε από ότι αντιλαμβάνομαι φυσικά**.

A:Ναι

**R:Οοκ, για να δω τι άλλο ήθελα να σε ρωτήσω, τα άτομα γενικά που είναι διασωληνωμένα και προσπαθούν να επικοινωνήσουν, όταν εσύ και εκείνα δεν καταφέρνετε να επικοινωνήσετε, δεν καταφέρνεις να καταλάβεις, τι πιστεύεις ότι νιώθουν τούτα τα άτομα ασπούμε που είναι διασωληνωμένα και δεν μπορούν να μιλήσουν**

A:Πρώτα από όλα ανησυχία επειδή δεν καταφέρνουν να επικοινωνήσουν μαζί μας δεν ξέρουν σε τι κατάσταση βρίσκονται , εεε δυσανασχετούν αφού προσπαθούν να μας πουν ένα πράμα που μπορεί να θέλουν να νιώθουν ανάγκη ότι χρειάζονται κάποιο πράμα, και εν μπορούν να επικοινωνήσουν μαζί μας για να τους πούμε αν μπορούν να κάμουμε κάτι για τζίνο το πράμα ή αν δεν μπορούν , αν δεν μπορούμε να κάμουμε κάτι τουλάχιστο να τους καθησυχάσουμε ότι εν εντάξει εκείνο το πράμα που νιώθουν, άρα εν καταφέρουν να επικοινωνήσουν σίγουρα νιώθουν ανησυχία και δυσχέρεια εν δύσκολο πράμα να πεις ότι έχεις κάποιο πρόβλημα και να μεν το καταλάβει, εννοείται εν δύσκολο.

**R:Οκ και που την πλευρά την δική σου τώρα;**

A:Και εμάς το ίδιο, αφού και εμείς έχουμε ένα άρρωστο , είπα σου και πριν εν ανήσυχος και εν μπορούμε να τον βοηθήσουμε γιατί δεν μπορούμε να επικοινωνήσουμε

**R:Άρα εν και για εσάς δύσκολο ουσιαστικά, οκ ήθελα να σε ρωτήσω στο τμήμα σας έχετε ταμπλετ και κινητό και ή για παράδειγμα κάποια μέσα άλλης επικοινωνίας πιο ψηλής τεχνολογίας για να επικοινωνήσετε με τέτοια άτομα ή όχι**

A:Έχουμε ταμπλετ αλλά εν έτυχε να το χρησιμοποιήσουμε για τέτοια περίπτωση

**R:Οκ , στο πανεπιστήμιο σου γενικά έχεις εκπαιδευτεί για θέματα επικοινωνίας;**

A:Ναι

**R:Οκ , γενιά με τούτους τους ασθενείς αν σου δινετουν η ευκαιρία να κάνεις ένα μάθημα με τους διασωληνομένους ασθενείς και πως να χειρίζεσαι καταστάσεις και αν είναι ξύπνιοι όταν είναι ξύπνιοι εννοώ θα ήθελες ή όχι να εκπαιδευτείς σε αυτά τα θέματα**

A:Εεεμμ εξαρτάτε ,εννοείς για καινούργια μέσα που μπορούμε να χρησιμοποιήσουμε;

**R:Ναι για καινούργια μέσα ή τρόπους επικοινωνίας με διασωληνομένους και ξύπνιους ασθενείς**

A:Εντάξει , εε, εν ξέρω, εξαρτάτε που την περίοδο

**R:Βεβαίως, οκ, εάν έλεγες στο τέλος να πεις ασπόυμε να εκπαιδευτείς, πως θα ήθελες να ήταν τούτη η εκπαίδευση, να ήταν με σεμινάρια να ήταν δίπλα που τον ασθενή να ήταν με σεμινάρια και δίπλα που τον ασθενή, να ήταν διαδικτυακά να ήταν που κοντά πως θα προτιμούσες;**

A:Νομίζω δίπλα που τον ασθενή εν δύσκολο να γίνει έτσι πράμα, άρα νομίζω ο μόνος τρόπος εν να είναι με σεμινάρια επειδή εν δύσκολο να έχεις άρρωστο που ξυπνά για να οργανωθεί το, εν η φύση της κατάστασης

**R:Οκ**

A:Εννοείται πάντα εν καλύτερο σε οτιδήποτε κάμεις ,να το κάμεις πιο πρακτικά, απλά θεωρώ ότι δεν είναι τόσο εφικτό

**R:Εντάξει οκ, εντάξει, νομίζω τέλειωσα εκείνα που ήθελα να σε ρωτήσω, απλά αν σε ρωτήσω ακόμα μια φορά σκέφτηκες καμιά εμπειρία διαφορετική ή κάτι άλλο θέλεις να μοιραστείς μαζί μου στο οποίο απλά έτσι για συζήτηση όσο αφορά την εμπειρία σου ασπούμε**

A:Οι εν μου έρχεται κάτι συγκεκριμένο, είπα σου, όσο παραπάνω διαρκεί τούτο το πράμα ένας διασωληνομένος ξύπνιος ασθενής εν κουραστική η κατάσταση και για τον ασθενή και για τον νοσηλευτεί που εν κοντά του, εν τούτο το χαρακτηριστικό που μου έρχεται, εν δύσκολο και για τον ένα και για τον άλλο, εν δύσκολη κατάσταση

**R:Οκ thank you constantino ευχαριστώ για τις πληροφορίες, να είσαι καλά.**

No 5

R: researcher

A: aggelos

R: angelos, όπως σου είχα πει, κάνω μια έρευνα για τους διασωληνομένους ξύπνιους ασθενείς και τις εμπειρίες των νοσηλευτων που εργάζονται μαζι με αυτους τους ασθενεις γενικα, θα ηθελα να σου κανω μερικες ερωτησεις οσο αφορά τα προσωπικά σου δεδομένα τελος πάντων,όπως μου είπες και πριν λίγο θέλω να μου τα ξαναπείς, πόσα χρόνια εργάζεσε ως νοσηλευτής γενικά

A:8

R: 8 χρόνια και στην εντατική πόσο καιρό είσαι

A: Σχεδόν 5

R: Σχεδον 5 οκ, που εχεις σπουδάσει αν επιτρέπετε

A: Αρχικά στην σχολή και μετά έκανα την εξομοίωση πτυχίου στο ΤΕΠΑΚ, αυτά δεν προχώρησα παρακατω

R: Δεν εκαμες.. οκ, ντάξει δεν πειράζει,εεε, τι ηθελα να σε ρωτησω, συγνωμη, ειπες δεν εχεις προχωρήσει αλλου, ειπες είσαι 5 χρόνια στην εντατική, η εντατική σας, επειδή από ότι ξέρω στο Γενικό Νοσοκομείο έχει και CCU , έχει και ΜΕΘ , είσαι στην ΜΕΘ την γενική έτσι

A: Ναι

R: ΟΚ, εεεε, οσο αφορά τουτουτς τους ασθενείς, τους διασωληνομένους και ξύπνιους ασθενείς σε ποιες περιπτ’ωσεις το νοσοκομέιο σας, στην Μονάδα σας θα δώ τέτοιους ασθενείς, σε ποιες περιπτώσεις θα μπω στην Μονάδα σου και θα δω ένα διασωληνομένο αλλά ξύπνιο ασθενή, δηλαδή να έχει τον σωλήνα, όχι τον τραχειοστομημένο, διασωληνομ΄νέο αλλα ξύπνιο, δηλαδή σε ποια φάση ένα τον δω

A: Συνήθως στην φάση που επέρασε την κρίσιμη φάση ,την οξεία φαση, προσπαθουν να δουν πως αναπνεει και αντιμετωπίζει από μόνος του την κατάσταση ,εεε, δηλαδή ,τούτο εξαρτάτε πόσο καιρο ηταν διασωληνομένος απλά να δουν την κατάσταση και εμεις μαζί τους ,κατά πόσο ελέγχει την αναπνοη του από μόνος του,δηλαδη δεν εχει χρονική περιστάση ασπούμε το πράμα, εεε

R: Οκ, δηλαδή μετά που χειρουργείο ο ασθενής;

A: Nαι, Συνηθως έχουμε χειρουργεια πλεον, ενταξει πολυτραυματιες όχι τοσο συχνα, επιασαν τα πρωτία στην λευκωσια έχει καιρο δεν εχουμε πολυτρυτματιες,ο τελευταιος πολυτραυματιας ναι ηταν προσφατα, μπορώ να σου πω για τον τελευταιο πολυτραυματια ηταν γύρω στις τρεις μερες μετα΄το χειρουργειο του, για οτιδήποτε άλλο που κυρίωσς εν χρόνια περιστατικά, ξύπνιοι αρρωστοι, συνήθως έρχονται από λευκωσια ,δηλαδή μπορεί στην λευκωσια να καμουν 2-3 βδομάδες και να μας έρχονται σε εμας για απογαλακτισμο και να είναι ξύπνιοι αλλα διασωληνομένοι

R: Οκ, δηλαδή τούτοι όλοι που μου λες εν τρυαματίες οι περισσσότεροι

A: Οι περισσότεροι ναι

R: Που κάνουν ασπουμε ασπουμε ξύπνιοι;

A: Nαι απλα έρχονται από λευκωσία ως διακομιδή στην λεμεσο για απογαλακτισμό κυρίως

R: Αρα η φάση που θα τους δούμε εμεις ξύπνιους και διασωληνομένους εν η φάση που προσπαθειτε να τους βγάλετε από τον αναπνευστήρα και προσπαθείτε να τους αποσοληνώσετε; Αν δεν κάνω λάθος η φάση του weaning που λέμε

A: Ναι ναι

R: OK, υπάρχει οποιαδήποτε άλλη περίπτωση που να σου έτυχε να δεις ασπουμε, που να είναι διασωληνομένος και ξύπνιος ο ασθενής

A: Δεν νομίζω, δεν μου έρχεται κάποια στιγμή, δηλαδή ενταξει το να ξυπνήσει ενας ασθνεής ασπουμε ,εεμμ και να μην παιρνει ασπουμε τις καταστολές του και να ξυπνήσει τα μάτια κτλ εκεί δεν θα προσπαθήσω να επικοινωνήσω, απλα ένα του πω κυριε Ταδε, ήρεμα είσαι οκ, εμμ μην αντιδρας τόσο πολλά ,έχεις κατι στον λαιμό σου κτλ για να αναπνεις, μέχρι να τον κοιμήσω ασπουμε, δηλαδή γιατί πρεέπει να είναι κοιμησμένος ο ασθενής γιατι ακόμα είναι οξεία η φάση

R: Οκ..γενικά πως είναι να δουλευεις με τους διασωληνομένους ασθενείς, σε ξύπνια φάση, γενικά η εμπειρία σου ασπούμε

A: Κοίταξε εμενα επειδη, εγω ήθελα να πάω στην εντατική, ηταν δική μου επιλογή να παω στην εντατικη , απλά έτυχε να υπάρχει θέση για να πάω, ηταν η περιοδος που έφυγα από την Πάφο και ήθελα να κάμω κατι παραπάνω, δηλαδή τέσσερα χρόνια στα Παθολογικά, ήθελα λίο να προχωρήσω, αφου οικονομικά δεν εμπορούσα να κάμω μεταπτυχιακό, ήθελα ασπούμε να παω κάπου αλλού για εμενα, οπότε, έτυχε να ανοιξουν θέσεις στην εντατική γιατι εφευγαν ατομα γιατι ανοιγε μια άλλη κλινική στο νοσοκομείο της λεμεσου, άνοιγε η θέση και είμουν πάρα πολλά τυχερός, είχα και άτομα μαζί μου που ήθελα να με βοηθήσουν κτλ, ήταν όλα πάρα πολλά καινούργια κτλ, στην αρχή δεν εμπορόυσα να το κοντρολάρω, ηταν παντελώς διαφορετική φάση από το παθολογικό, όποτε εμένα ιντρικαρε μου τουτοτ το πράμα να μπω στην διαδικασία να δω ποια ηταν η όλη φάση του ασθενή παρά εκίνου του κτακεκλιμένου, μεγάλης ηλικίας κτλ, όχι τωρα οι παραπάνω δεν είναι μεγάλης ηλικιάς αλλα ενταξει εχει διαφορά, το ν επικοινωνείς μαζί με ένα ασθενη ο οποίος είναι διασωληνομένος, δηαλδή, κλείσε μου τα μάτια σου, σφύξε μου το χέρι σου, τοο, εε,, κούνα μου το κεφάλι σου, κούνα τα πόδια σου, τούτο το πράμα, ασπούμε τούτου η άλλου είδους εποικοινωνίας , κάμνει σε και εσένα να σκεφτεις, πως μπορεις να επικοινωνήσεις και εσυ λίγο καλύτεραα, μπορεί να εν ξένος, μπορεί να μεν σε ακούει, μπορει να εχει προβλημα όρασης κτλ, τουτα ουλλα βάλεις σε εσενα να σκεφτεις πως μπορείς να επικοινωνήσεις καλύτερα με ένα ανθρωπο που δεν μπορεί να μου μιλήσει, τουτα

R: Ωραία ,ωραία , είπες μου πολλές ωραίες πληροφορίες, θέλω να πιαστώ ένα προς ένα, εεμμ, εσυ ως νοσηλευτής πιστευεις ότι αντιλαμβάνεσε τις αναγκες των ασθενων ,όταν θα ξυπνήσουν και θα είναι στον σωλήνα, τι πιστευεις ότι χρειάζονται τούτοι οι ασθενείς και είναι διασωληνομένοι και ξύπνιοι, δηλαδή, τι πιστεύεις ότι προσπαθούν να ζητήσουν από εσενα, τι ενει η εννοια τους, τι έχουν στο μυαλό τους πιστέυεις;

A: Στο μυαλο τους καταρχάς, εγω θεωρω ότι το discomfort που έχουν εκείνη την στιγμή, προσπαθούν να καταλάβουν τι, γιατι έχω τούτο στον λαιμό μου και κυρίως, τούτοι οι ασθενείς που ξυπνούν, η πρώτη τους έννοια είναι να σηκώσουν τα χέρια και να τα πάρουν στον σωλήνα

R: Μαλιστα..

A: Οπότε προσπαθείς να τους το εξηγήσεις, τωρά παίζουν ρόλο και τι κατασταλτικά παίρνουν

R: Δηλαδη; θέλεις να μου μιλήσεις περισσότερο γιατί εγω δεν δουλέυω σε εντατική αρα..

A:Εμεις κυριώς δουλευουμε στην Λεμεσο με propofol, fentanyl και Dormicum

R:Μαλιστα

A: Συνδυασμός των τρειων, μπορει να μην τα παίρνει και τα τρεία αλλα εξαρτάτε και από τις δοσολογίες που θα τους δίνουμε, οι πιο νεαροί χρειάζονται πιο ψηλές δοσολογίες και λόγω μεταβολισμού

R:Μάλιστα

A:Ενώ ασπούμε κάποιοι πιο μεγάλης ηλικίας εε χρειάζονται μικρότερες για να κοιμούντε, σε ένα νερό,παραδειγμα ο νεαρος π είχαμε προσφατα , έπαιρνε ψηλές δόσεις τούτων ούλλων στην αρχη ηταν συγχυτικός, λόγω του dormicum,όποτε επρεπε να τον σιγουρευουμε κάθε φορά ότι εν ήρεμος,

R:Ο συγκεκριμένος νεαρός, συγνώμη, τί είχε ασπούμε

A:Ηταν πολυτραυματίας με την μοτοσυκλέτα κλπ, είχε bilau δεξιά, εε, είχε, έκαμε χειρουργέιο και είχε και θλάση, όχι ρίξη, νομίζω δεν ηταν ρίξη στο ηπαρ, εκαμε χειρουργειο στο ηπαρ τελος παντων

R: Σορρυ εδιέκοψα σε

A: Απλά ο συγκεκριμένος ηταν 1.90 100kg, όποτε και αναλόγως kg πάει ασπούμε και η καταστολή, οποτε τουτο τον ασθενή έπρεπε να του λέμε, Παναγίωτη είσαι εδώ, έπαθες τούτο, μεν προσπαθείς να φκάλει τον αυτοο, εε, είσαι στην Λεμεσο, είσαι διασωληνομένος, και θα φκλαουμε τον σωληναα σιγα σιγα

R: Πολύ ωραία

A: Υπάρχει και μια διαδικασία υποτίθεται στην Εντατική, να τον περασω, ασπούμε που ελεγχόμενο μοντέλο σε spontaneous μοντέλο, ένα τον βάλεις και σε tpiece πάνω στον σωλήνα

R:Επειδή εγώ δεν τα ξέρω αυτά, θέλω να σε ρωτήσω, αυτά δηλαδή επειράζουν την επικοινωνία, δηλαδή θα έχει κάποια διαφορά στον ασθενή ή σε εσένα

A:Εμμ, οι ιδιαιτερα

R:Ναι

A:Απλα καταλαμβαίνεις εσυ αν κατανοεί εκείνα που λές

R:Δηλαδή; πολλά ενδιαφέρον τούτο

A:Δηλαδή, ξέρεις ασπουμε στον συγκεκριμένο, ξέρεις τωρά κάνουμε τούτη την διαδικασία, είσαι καλός στον αναπνευστηρα, ένα φκει ο σωλήνας, είμαστε δίπλα σου ασπουμε και κουνούσε το κεφάλι ή εκλεινε τα μάτια ασπούμε, το να του λέεις τι έχει γιατι εν το σώμα του και τι επηρεάζει στο σώμα του μια μηχανή , νομίζω πρεπει να το ξέρει ενας ασθενης, με τουτη την ενοια το λεω

R:Μαλιστα, μπορείς είπες μου έτσι μερικούς τρόπους εποικοινωνίας που επικοινωνας με τουτους τους ασθενεις, να τους εξηγήσεις που είναι κλπ, το αγγιγμα, εε, έρχεται οτιδήποτε άλλο στο μυαλό το οποιο θα σε βοηθήσει να επικοινωνήσεις ή μπορείς /θέλείς να μου κάμεις μια αναπαράσταση ασπουμε, αν ειμουν εγω ασθενής, τι ηταν να έρχεσουν, πως θα επικοινώνας, αν σε βοηθά τουτου, να θυμηθείς τους τρόπους επικοινωνίας γενικά

A:Προσεγγίζεις τον ασθενή,προσπαθείς να δεις αν εστιάζει το βλέμμα του πάνω σου, μαλιστα, γιατι τουτο το πραμα, βλεπεις και νευρολογικα την κατασταση, μπορεί κάποιος να ξυπνά αλλα νευρολογικα να μην μπορεί να επικοινωνήσει μαζι σου

R:Εννοείς να αντιλαμβάνεται αλλα να μην μπορεί να εποικοινωνήσει μαζι σου;

A:Ναι, δηλαδή αν εστιάζει το βλέμμα πάνω σου σημαίνει ότι ακουει σε και επικεντρώνεται εκει που έρχεται η φωνή, καταρχάς, τα μάτια, εάν του δώσεις μια εντολή παράδειγμα, σφίξε μου το χέρι κλείσε μου τα μάτια ένα κταλάβεις νευρολογικά ότι μπορεί εκείνος ο ανθρωπος, ότι εν οκ

R:ΜΜΜ

A:Εεεε με τούτη την ενοια το ενοω τουτο

R:Οκκ…εεε ειπες, εξαναρώτησα σε αλλα συγνωμη δεν ειμαι σίγουρη αν μου απάντησες, γενικά οι ανάγκες, τι νομίζεις ότι προσπαθούν ν σου πουν οι ασθενείς μωλις ξυπνήσουν, ενοω

A:Το να μου πουν,εσυ ειπες μου που την πλευρα σου τι πιστευεις, ότι προσπαθεις να τους κατατοπίσεις από ότι εκαταλαβα που είναι κλπ να τους πεις τι έχουν πάνω τους

R:Ισως να μην εμπηκα και τόσο στην διαδικασία να καταλάβω απευθειας τι θέλουν να μου πουν όταν είναι διασωληνομένοι, γιατί δεν μου έρχεται κάτι, συνήθως εγω προσωπικά κάμνω προσωπικά ερωτήσεις και προσπαθω να καταλαβω, δηλαδή παίρνω εγώ την συζήτηση εκεί που θέλω

A:Πολύ ωραία, εν μια στρατιγική τουτη

R:Ναιι

A:Μάλιστα,πολύ ωραία, εε τουτοο

R:ΟΚ..

A:ΕΕΕ τουτο εμαθα το σιγα σιγα, γιατι μπορεί να θέλουν να σου πουν πολλά,ενοείται δεν μπορούν να σου τα πουν απλα καθοδηγείς εσύ την συζήτηση στο που ένα πάει για να καταλήξεις κάπου, γιατι αν, θεωρώ αμαν αφήσεις ένα ασθενή να προσπαθήσει να σου πει και δεν είναι καλός αναπνευστικα αλλα οι γιατροι θελουν να τον φκάλουν που τον αναπνευστηρα γιατι εν καιρος να φκει που τον αναπνευστήρα παράδειγμα λεω το πολλα απλα,για να το πω..

R:Ναι ναι

A:Και βλέπεις ότι εκείνη την ώρα προσπαθεί και χαλά ο τρόπος αναπνοής του με τον αναπνευστήρα, χαλά και γενικώς αναπνευστικά, οπότε πάει λίγο πίσω αν είναι ανησυχος, με τουτη την ενοια το λεω

R:Ναι εκατάλαβα, μαλιστα, τι νομίζεις ότι εν εκεινο που σε εμποδίζει να επικοινωνήσεις κάποιες φορές με τους ασθενείς, δηλαδή ποια εμποδία βρίσκεις στην επικοινωνία σου με τούτους τους ασθενείς; ΕΕ ειπες μου για παράδειγμα, το αυτονόητο που είναι ο σωλήνας στο στόμα,δηλαδή τουτο εν ένα καθαρά ένα εμπόδιο, είπες μου το άλλο ασπούμε εξαρτάτε που τα φάρμακα που παιρνουν, γενικά ασπουμε αν μπορείς να σκεφτεις την εικόνα που δουλευεις στην ε τνατική τι εν εκεινο που , που δεν σε αφήνει να επικοινωνήσεις με τούτους τους ασθενείς ασπούμε, εχτός που τούτα τα δύο για παράδειγμα, εε

A:Εεε εντάξει,

R:Αν μπόρεις να σκεφτείς κάτι

A:Ισως εεμμ,μπορεί να είναι και το language barrier ασπουμε

R:Μάλιστα, δηλαδή;

A:Μπορεί να είναι κάποιος τουρίστας , ο οποίος αγγλικά δεν ξέρει, ελληνικά δεν ξέρει, να εν ρώσσος ασπούμε

R:Αχαμ.. μάλιστα

A:Τούτο εν μου έτυχε πολλά συχνά και δεν μου έτυχε προσωπικά, αλλά ξέρω από συναδέλφους ασπουμε που είχαν κάποιο ρώσσο που δεν μπορούσαν να επικοινωνήσουν μαζι του, να του πουν ασπούμε, την διαδικασία που σου είπα πριν

R:Μαλιστα

A:Τουτο το πράμα εεενα προχωρήσεις με τον ασθενή μόνο με πρακτικά..

R:Δηλαδη;

A:Δηλαδή θα προχωρήσεις, ξέρεις, εν θα του πεις, όπως ένα του πεις κάποιου ασθενή, θα προχωρησουμε να κάμουμε τούτο το πράμα που επικοινωνεί και μπορεί να καταλάβει, θα τον δεις στον αναπνευστήρα και μέσω των αεριών αίματος ασπούμε, θα δεις αναπνευστικά εν καλά, κάμνει καλούς όγκους που μόνος του κάμνει 15-20 αναπνοές, αα ένα τον φκάλω

R:Δηλαδή θα αποφύγεις να του εξηγήσεις γιατι δεν καταλαμβαίνει ενοείς ασπούμε

A:Ναιι ναι, για την γλώσσα, μόνο τουτο μπορώ να εβρω ότι εν μπορώ να επικοινωνήσω.

R:Οκκ

A:Εν μπορώ να εβρω κατι άλλο γιατι μέχρι στιγμης δεν είχα καποιον ασθενή που δεν μπορώ να επικοινωνήσω, εχτος και αν είναι ασθενης με ανοια alzjeimer και τούτα ούλλα τα πράματα .και η γλώσσα, μόνο τουτα τα πράματα

R:Οκ, οι ασθνεις αμαν προσποαθουν να σου πουν κατι και εσυ δεν καταλαβαίνει ή δεν είναι σε φάση να σου εξηγήσουν καλά τι θέλουν κλπ, πως νομίζεις ότι νιώθουν εκείνη την ώρα

A:To να μην μπορείς να επικοινωνήσεις είναι το πιο δύσκολο πράγμα γενικώς , εε, βλέπεις την απογοητευση, βλέπεις το στο πρόσωπο τους, δηλαδή έχει κάποιους που χτυπούν π΄νω τους ας πούμε καμνουν τουτο το πράμα *(δειχνει από κοντά)*

R:Nαιι

A:Δεν με καταλαμβαίνεις, οσο και να προσπαθεις και να του λεεις όπως σου ειπα πριν ότι προσπαθω να παρω την συζητηση ασπουμε σε πράματα που είναι σε νοσηλευτική πλευρα παράδειγμα,πονείς, θελεις να γυρίσεις, πραματα που μπορώ να τον βοηθήσω νοσηλευτικά εκείνη την στιγμή, έτυχε μου απλά που στην τελική ήθελε να επικοινωνήσει με την οικογένεια του

R:Πως το αντιλήφθηκες τούτο το πράμα;

A:Ενεν εγω προσωπικα, εν μια ιστορία ασπούμε , κάποιος ασπούμε,εκαταλάβαμε ότι είρθε κάποιος συγγενής μέσα και έδειχνε με, εδειχνέ με όχι εμένα, κάποιο συνάδελφο και εν τούτο που ήθελε ασπούμε

R:Αντιλήφθηκες ότι ηθελε..

A:Ναι ναι αλλα εν μπορείς, πως να το καταλάβεις εκεινεη την ωρα; Εν τούτο που θέλω να πω, ότι ενας νοσηλευτης και γιατρός κυρίως εν την δουλεία του που θα κάνει, ναι έχει και μια πιο ανθρωπιστική πλευρα το επάγγελμα μας, απλά πάει πρώτα το επάγγελμα..

R:Βεβαίως αντιλαμβάνομαι

A:Δεν θα παει τοσο βαθιά ενοω, γιατι εκει, γιατι το να φευκεις εξω που το νοσηλευτικο εν κακό κιολας

R:Δηλαδή τι ενοεις ακριβως για να καταλάβω

A:Εεμμ, οι εν κακο που δεν φκενουμε τοσο που το νοσηλευτικο, οι ότι εν σωστο το ότι εεεε

R:Ενοείς ότι κάμνεις μόνο τις διαδικασίες ασπούμε και εν κάμνεις κάτι άλλο τούτο εν κακό (;) ενοεις

A:Ναιι… ναι, γιατι έχουμε να κάμουμε με ανθρώπους καταρχάς

R:Μάλιστα

A:Και δεν βγαίνουμε εμείς πολλές φορές που.. εν η δουλέια μου και θα κάμω την δουλεία μου και εν με κόφτει ασπούμε

R:Γιατί πιστεύεις όμως ότι κάποιοι νοσηλευτές το κάνουν αυτό; Δηλαδή κάμνουν την δουλεια, στην εντατική μιλώ

A:Στην εντατική; Εν δύσκολο το περιβάλλον για να μπεις και τοσο, για να κάμεις διαπροσωπικές σχέσεις, διαπροσψπικές σχέσεις θα κάνεις με ασθενείς οι οποιοί, όπως το είδα εγω ως τωρά, που ένα σε δουν και εσένα ως άνθρωπο, γι<τι τους νοσηλευτες δεν τους βλέπουν ως ανθρώπους πάρα πολλόι , να σου το θέσω έτσι

R:Δηλαδή ασπόυμε , ενοείς εν συμπεριφέρονται καλά;

A:Nαιι..

R:Οκ, έχετε και τούτο το ζητούμενο

A:Δηλαδή διαπροσωπικές σχέσεις εεε , παράδειγμα ένα , επειδή εγω έχω και στου; Διασωληνομένους στο COVID-19, στην απομόνωση

R:Έχετε τωρά τμήμα;

A:Οι εμείς εν έχουμε,στέλουμε το στην λευκωσία ή στο παραλίμνι, απλά τότε που ηταν η πρώτη φάση , είχαμε απομόνωση με τέσσετα κρεβάτια στην λεμεσό στον 5^ο^ όροφο και είμουν μέσα στην ομάδα

R:Οκ.. μάλιστα

A:Εεεε, όταν εκατεβήκαμε ξανά στην ΜΕΘ κάτω και εξεκίνησε να επικοινωνέι ,νομίζω είδες το βίντεο του αναστάση, ο μιχαηλ αναστασίου νομίζω που είναι, έκαμε και βίντεο στο FB Που μας ευχαρήστησε όλους τους νοσηλεύτες, βγήκε πρόσφατα και έκαμε ένα βίντεο

R:Νομίζω δεν το είδα, να το δω όμως, πες μου ναι, είναι πολύ ενδιαφέρον

A:Ο συγκεκριμένος, είναι χρυσός άνθρωπος, επέρασε τόσα και τόσα, εεε και ότι και να του λαλούσαμε του συγκεκριμένου ανθρώπου εε ηταν τοσο προθυμος να τα κάμει, δηλαδή εφτασε ως εδώ να πεθάνει , που το κοβιτ, είχε τοση θέλησει να μας βοηθήσει εμας κυρίως που και μετά τον ευατό του,δηλαδή, επηγαίναν φυσιοθεραπευτές, εβοηθούσε τους φυσιοθεραπευτές, ελαλούσαμε του αναστάσιο μου, εε το και το σήμερα, ατε και ένα τα καταφέρεις ασπούμε και εσυγκινίτουν κάθε φορά που τον εβοηθούσαμε. Δηλαδή εβλεπες το στο πρόσωπο του ακόμα και διασωληνομένος εκείνος ο ανθρωπος, έβλεπες στο πρόσωπο του ότι εταλεπορούσε εμάς

R:Αντιμετωπίζεται το συχνά τουτο το φαινόμενο δηαλδή που ασθενείς

A:Εν σπάνιο φαινόμενο να βλέπεις ένα ασθενή που να επικοινωνεί τόσο πολλά ξεκάθαρα μαζί σου που ότι κάμεις εε εκτιμά το γιατί είπα σου και πριν οι παραπάνω εν συγχυτικοί, εν το συνηδητοποιουν, ένα το συνηδιτοποιήσουν προς το τέλος, την ώρα που έχουν τον σωλήνα, το να επικοινωνας 100% μαζί τους δεν είναι εφικτό πολλές φορές.

R:Δηλαδή έχει εε, φαντάζουμε , λαλέις μου οι παραπάνω την παραπάνω ώρα εν συγχυτικοί, δηλαδή εν λίγη η ώρα που είναι ξύπνιοι εντελώς διαυγές ασπούμε το μυαλό τους και να είναι στον σωλήνα; Εν λίγο πριν την αποσωλήνωση τούτο το πράμαα;

A:Nαιι

R:Δηλαδή δεν θα είναι πολύ ωρα στον

A:Εν θα είαι παρα πολύ ώρα

R:Άρα έχετε να κάμετε παραπάνω με τους συγχυτικόυς , άσχετο πως εν ξύπνιοι, λόγω των φαρμάκων

A:Ναι ναι

R:Εεεμμ, τούτο που μου είπες τωρά, αντιμετωπίζεται συχνά ασπούμε έτσι καταστάσεις που τους ασθενείς να μην σας αντιμετωπίζουν σωστά ως νοσηλευτές; Να μην σας σέβονται να το πω, δεν κερω πως να το πω, εεε

A:Εεενεν συχνο φαινόμενο, ενεν συχνό φαινόμενο απλά διας τόσο πολλά μεσα στην εντατική, νομίζω διας παραπάνω πράματα, εν βάλω τους νοσηλευτες της εντατικής παραπάνω που τους άλλους νοσηλευτές, απλά θεωρω ότι ολλοι οι ασθενείς εκει μέσα εν στην κόψη του ξυραφιού.

R:Μάλιστα

A:Μια παρέμβαση να κάμεις, χάνεται.. ο ασθενής οπότε εε και επειδή εν κοιμησμένοι οι ασθενεις που οι παραπάνω παρεμβάσεις που γίνονται πάνω στον ασθενή , εν κοιμισμένοι, δεν συνηδιτοποιούν την δουλεία που κάνεις, γιατι η δουλεια μέσα στην εντατική είναι One to one , ενας νοσηλευτής ενας γιατρός ενας ασθενής, νταξει; Το να στέκεσε που πάνω που ένα ασθενή 6 ώρες, σε εισαγωγικά οι 6 ώρες, το να στέκεσε να κάμεις τόσα πολλά πράματα και να μην το ξέρει εν λόγικό να μεν το καταλάβει, ξέρω το απλα θέλεις και μια ηθική ευχαρίστηση που έκανες εκείνα τα πράματα για κάποιον αγνωστο,γιατι ασχετο αν είναι δουλεια, εν με ζωη που έχεις να κάνεις

R:Σωστό

A:Επειδή έβλεπα το τουτο στον Αναστάζιο που ηταν ξύπνιος , εε και ελαλε μας το ευχαριστώ και..

R:Πως σας ελάλε ευχαρι.. ξανα επιστρέφω πισω ασπουμε, με ποια..

A:Τότε που ηταν διασωληνομένος;

R:Nαι θέλω να μου κάμεις έτσι λίγη παραπάνω αναπαράσταση

A:Με κινήσεις, το ευχαριστώ ασπούμε, τούτο το πραμα, μόνο και μια κίνσηη το ευχαριστώ, τίποτε άλλο, εν και θέλεις κάτι..

R:Επιστρέφω πίσω που σε ρώτησα, πως νομίζεις ότι νιώθουν οι ασθενείς στην περίπτωση που δεν καταφλερνουν να επικοινωνήσεις και να καταλάβεις το τι θελουν να σου πουν, εσυ που την πλευρα σου ασπούμε,πως νιώθεις ασπούμε όταν υπάρχει περίπτωση για οποιδήποτε λόγο να μην καταφέρεις να καταλάβεις και να επικοινωνήσεις με τον ασθενή, τι,πως νιώ..

A:Ζητώ δεύτερη γνώμη

R:Ζητας δευτερη γνώμη,δηλαδή;

A:Δηλαδή ένα φέρω ένα άλλο νοσηλευτη και ένα του πω , καταλαμβαίνεις τι λέει; Ή πολλές φορές μπορει ναα είμαι σε τουτη την φάση, δηλαδή μπορεί να είμαι εγω ο δεύτερος, είναι συνεργασία τούτο το πράμα, δηλαδή μπορεί ενας νοσηλευτης να δουλεψε μαζί με ένα ασθενή παραπάνω που εμεένα, να ξέρει το χουι του που λέμε παραπάνω π εμένα, οπότε ένα ζητήσω βοήθεια, εμαθα να δουλευω και πιο ομαδικά, στην εντατική , η εντατική είναι team work, δεν μπορείς να δουλέψεις μονος σου ,οπότε αμαν εν μπορώ να καταλάβω ένα ζητήσω βοήθεια, μπορεί και που γιατρό μπορεί ακομα και ενας βοηθός, να ρωτήσω καποιον άλλο και να σου πει τουτο που θέλει κάθε φορα και να σου το πει και να το πεις του ασθενη και να σου πει ο ασαθενης και να σου πει ναι εν τουτο που θέλω,

R:Μαλιστα, εννοείς να σου νέψει ασπούμε, οκ, πολλά ωραιο τούτο που λες

A:Ναι, σπάνια θα γυρίσω να φύγω, δηλαδή εκαμα το και λάθος αλλά εεμμ το να γυρίσεις να φύγεις είναι εκει που καταλαμβαίνεις ότι εεν πως να το θέσω, ξέρεις ότι δεν μπορείς να του το δώσεις , δεν μπορείς και επειδή επιμένει μεσα στην σύγχυση του εν μπορείς να τον ηρεμίσεις

R:Ένα λεπτό, δηλαδή δεν μπορείς να ασπούμε,αντιλαμβάνεσε τι ζητά αλλα εν μπορείς.. σε τι συγκεκριμένα αναφέρεσε;

A:Ναι τούτο που σου είπα εν αμαν αντιλαμβάνομαι τι ζητά, εσύ εκείνο που θέλεις εν αμαν δεν αντιλαμβάνομαι τι ζητά

R:Ναι σε περίπτωση ασπούμε που δεν καταφέρνεις να επικοινωνήσεις

A:Ναι εν τούτο με τους συναδέλφους

R:Αν ο συνάδελφος σου δεν καταφέρει να καταλάβει; Εν τούτο που θέλω να πω , θέλω να δω τι νιώθεις ή ειπες μου τι κάνεις

A:Εν απογοητευση που νιώθεις κυρίως

R:Απογοήτευση οκ

A:Δηλαδη αμαν δεν μπορείς να δώσεις κάποιου ανθρωπου και ζητα΄εκεινη την στιγμή εν απογοήτευση

R:Οκ έχει κάποιο συγκεκριμένο περιστατικό που σου έμεινε στον νου για οποιοδήποτε λογο; Όχι μόνο γιατι δεν κατάφερες να επικοινωνήσεις, κάτι που σου έμεινε εσενα προσωπικά και σου έκανε εντύπωση

A:Πάνω σε τι εντυπωσή;

R:Ότι θέλεις ότι θέλεις ασπούμε, όσο αφορά την επικοινωνία σπουμε ή να εβρεις κάποιο εμπόδιο ή να εβρεις καποιο, κάτι που σε εβοήθησε και να πεις α μπράβο τούτο εβοήθησε με και κατάφερα να επικοινωνήσω, κάτι που να σου έμεινε εσένα στον νου βασικά, κατι που να σου εμεινε στον νου

A:Ναι, κάτι που θυμήθηκα τωρά, είχαμε ένα εξ ελλάδος ο οποίος ηταν με gullain Barraie εεε αλλά ήταν τραχειοστομημένος, πειράζει;

R:Κατακρίβεια προτιμώ διασωληνομένος,θυμάσε που πριν που ηταν διασωληνομένος που πριν να προχωρήσει σε τραχειοστομία;

A:Nαι έκανε το και πριν να γίνει τραχειοστομία anw εμμ χτυπούσε το κάγκελο του κρεβατιού, δύο , ένα,

R:Δηλαδή ηταν σύνθημα; το ένα σημαινει..

A:Nαι ηταν συνθηματικό

R:Δηλαδή το ένα σημαίνει τούτο το πράμα, το δυο τουτο..

A:Δεν θυμούμε ακριβώς τι ηταν το κάθε ένα, ηταν ασπούμε, ήταν συνεχόμενο, θέλω σε τω΄ρα αν ηταν τρεία μπορεί να ηταν το οτιδήποτε άλλο, επειδή ηταν νεαρός , ηταν ισως και λίγο πιο πανούργος να το πω (γέλιο), λίγο πιο έκοφκε ο νους του, δεν ξέρω ειβρε μια επικοινωνια λίγο πιο διαφορετική, να σου πω όμως, εμείς είμαστε και λίγο ανοιχτή εντατικη δεν είναι δωμάτια, εμεις έχουμε και λιγο, βλέπουμε τον ασθενή, δηλαδή ξέρεις ένα σηκωθώ εναα δώ , ένα βλέπω το μονιτορ ένα κοιτάξω και τον ασθενή, οπότε μπορεί και να μην χρειαστεί να κάμει κατι με τα χε΄ρια ή τα πόδια του γιατί ένα τον ακούσω, ένα τον ακούσω με ποιαν εννοια, ένα χτυπήσει ο αναπνευστηρας όταν ένα προσπαθήσει να καμει..

R:Α μπράβο και τούτο εν πολλά ενδιαφέρον

A:Δηλαδη με τον αναπνευστήρα ένα δεις, έκλεισε ο αναπνευστήρα, δεν παιρνει καλους ογκους; Ένα σου χτυπήσει ο αναπνευστήρα, την ακοή χρησιμοποιούμε την πολλά γιατί εν πολλά θορυβώδης χώρος και ξεχωρίζεις και τους ήχους και ένα παίξει ένα μηχανάκι και ένα πεις αα ρεε ενεν μηχανάκι οροόυ, εν μηχανάκι σίτισης, αν παίζει ο αναπνευστηρας αν παίζει το κρεβάτι, ξεχωρίζεις τους ήχους, έχουμε και τούτο το πράμα μεσα στην εντατική

R:Πολλά ενδιααφέρον, ευχαριστω που μου το είπες ,εε τι ηθελα να σε ρωτήσω; Κατι ελεγαμε για τουτο οκ, εε έχετε μέσααα επικοινωνίας στην μονάδα σας, δηλασή ασπουμε χρησιμοποιάτε που το πιο απλο ασπούμε, πέννα και κόλλα

A:Α τουτο ναι

R:Ή κάποιο πίνακα ασπουμε με σχήματα ,γράμματα κλπ;

A:Εεε τα σχήματαα, ναι , εχουμε ναι δηλαδή ένα τους δώσουμε να γράψουν κάτι, συνήθως εν τα πολύ κατά΄φερνουν αλλα κατι είναι, αα ναι ένα πρόσφτατο περιστατικό, ε΄γραψε μου ένα κινήτο κάποιος ασπούμε, αριθμους και ερωτήσα , εμμ ερωτήσα ποιου είναι ασπουμε και μετά εσυγκρινα το με τα νούμερα που έχουμε ήδη των συγγενών, ήθελε να επικοινωνήσει με την γυναίκα του, με την κόρη του εν θυμούμε τωρά

R:Σαν ηταν διασωληνομένος τωρά τούτος ο ασθενης;

A:Εεεε, ναι ηταν κον΄τα να αποσωληνωθεί, δεν εκαταλαμβαινες και επακριβως τους αριθμους, ηταν ασπουμε εκεινο το εν το κρατώ σωστά, γιατι όταν είσαι διασωληνομένος χάνεις παρα πολλά την, εεμμ πως το λαλουν ρε παιδί μου την λέξη, δεν κρατουν σωστά ρε παιδί μου το μα.. δεν έχουν την δύναμη να κρατήσουν

R:Την δύνααμη, μυικό τόνο ενοείς;

A:Nαι μυικό τόνο, ναι σωστό

R:Μαλιστα, εε αρα χρησιμοποιάτε κόλλα και πέννα είπες μου μεχρι στιγμής, χρησιμοποίατε κάτι άλλο πιο εξειδικευμένο ας το πω

A:Οιι

R:Οιι, εχετε; ασχετο αν το χρησιμοποιατε στο τμημα σας

A:… Νομίζω πως εν εχουμε

R:Εν έχετε

A:Εν εχουμε γιατι συνήθως στο, εν παραμένουν τοσο πολλά ξύπνιοι με σωλήνα

R:Οκ

A:Δηλαδή, θα είναι κατω που ώρα

R:Κατω που ώρα, μαλιστα

A:Δηλαδή η διαδικασία της αποσωλήνωσης γίνεται γρήγορα, αν παίρνει κάποιος καταστολές, dormicum,fentanyl, propofol ένα τα κόψεις για να ένα μισαώρο σαραντάλεπτο για να δείς το πως ξυπνά, ένα προσπαθήσεις μαζί του να του εξηγήσεις κάποια πράματα, ένα του αλλάξεις το μοντέλο του αναπνευστήρα, να δεις αν μπορεί να αναπνεύσει και από μόνος του ή μπορεί να είναι ήδη spontaneous που μπορεί να αναπνέει μόνος του που τον αναπνευστήρα με λίγο Push που τον αναπνευστήρα, μετα αμαν δούμε ότι με ένα αέριο αίματος εν οκ, η επικοινωνία που σου περιέγραψα αρχικά δήλαδή τούτο το πράμα είναι το πολύ ένα τέταρτο με ένα μισάωρο, δεν διαρκεί τόσο πολλά για να έχουμε τόσα πολλά μέσα γιατι δεν χρειαστηκε μεχρι στιγμης να έμεινε καποιος ασθενής για ένα 24ωρο πχ ξύπνιος ,θεωρούνε το και οι γιατροί μας και συμφωνώ μαζί τους, βασανίζετε ένας ξύπνιος ασθενής με τον σωλήνα, μπουκομέννο να το πω έτσι..

R:Μαλιστα, οκκ..

A:Ναι

R:Εεεε έχεις στο παρελθόν, που τέλειωσες ή γενικά στην καριέρα σου ασπούμε, εκπαιδευτεί σε θέματα επικοινωνίας;

A:Oχι

R:Όχι οκ, αν σου δίνετουν η ευκαιρία θεωρητικά να εκπαιδευτείς οσο αφορά την επικοινωνία να εκπαιδευτείς στο κομμάτι το δικό σου στους διασωληνομένους ασθενείς ή στους διασωληνομένους ξύπνιους ασθενείς θα ηθελες να το κάνεις;

A:Ναι γιατι όχι

R:Τι πιστευεις εσυ ως angelosβα ότι θα σε εβοήθα εσένα καλύτερα, να το έκανες ασπούμε , να είσασταν δίπλα που τον ασθενή, να εκπααιδεύεσουν δίπλα που τον ασθενή; να το κάνετε σε τάξη ας πούμε να το κάνετε δυαδικτιακά , τι θα σε βοήθαν εσε΄να καλύτερα;

A:Kοίταξε, επειδή το επάγγελμα μας είναι θεωρία και πράξη, πρέπει να ξέρεις την θεωρία και να την βάλεις σε πράξη

R:Μάλιστα

A:Το να κάμνεις την θεωρία και να σου πουν ξέρεις εν έτσι που μπορείς να εποικοινωνήσεις και να έχεις μια βάση και να πάεις μετά σε ένα διασωληνομένο ασθενή ο οποίος θα αποσωληνωθεί, νομίζω εν το πιο σωστό

R:Μάλιστα

A:Δηλαδή και μπροστά στον ασθενή αν ξέρεις εν ο ασθενής , πάει για αποσωλήνωση, γίνεται τούτη διαδικασία ναα εξηγήσουν ασπούμε στα άτομα που θα το δουν ή να το παρακολουθήσουν ξέρω γω, να γίνει πρώτη φορά να το δουν θεωριτικά που κάποιον να το κάνει και μετά ασπούμε να το κάνουν και μόνοι τους, νομίζω τούτο εν το πιο σωστό

R:Οκ μάλιστα, είπες μου πάρα πολλά ωραίες πληροφορίες πάντως, ευχαριστώ, θέλεις να μου προσθέσεις οτιδήποτε άλλο που σου είρθε τώρα στο μυαλό όσο αφορά τα εμπόδια της εποινοινωνίας ή αν έχεις κάποια στρατηγική που σε βοηθά, εε ή συναισθήματα ασπούμε που δεν καταφέρνεις να επικοινωνήσεις ή κατι άλλο γενικά να μου προσθέσεις που δεν είπαμε;

A:Στρατηγική προς επικοινωνίας..

R:Ασπουμε μια στρατιγική που μου εξήγησες που αντιλαμβάνομαι εγώ που κάνετε είναι ασπούμε, είδες τον ασθενή χτυπά μια φορά το κάγκελο, θέλει το τάδε πράμα, αμαν σου χτυπά δυο φορές το κάγκελο θελεί το τάδε πράμα ή ασπούμε λέεις μου ότι φαίρνεις κάποιο άλλο συνάδελφο σου να πάρεις δεύτερη γνώμη ασπούμε ή εε , έχετε οτιδήποτε άλλο, γενικά όχι μόνο στρατηγική, το οποίο σε ευκολίνει να επικοινωνήσεις, ή σε δυσκολεέυι;

A:Νομίζω….. εε, εν έκατσα ποτέ να σκεφτώ κάτι που χρειάζεται παραπάνω, γιατί με ότι, επείδη είχα ατόμα δίπλα μου τα οποία εδείξαν μου κ΄ποια πράματα και απλα εγω αναπτυξα τα προς την δική μου την πλευρά, νομίζω είμαι καλλυμμένος μέχρι στιγμή με τούτο, αν όμως κάποιος σκεφτεί μέχρι στιγμής κάτι παρα πολλά πιο έξυπνο , ενοείται ένα το υοθετήσω αλλά εγω προσωπικά δεν χρειαστήκε γιατί μπαίνεις σε τούτο το mode να επικοινωνήσεις με τον σωλήνα πάρα πολλά λίγο χρόνο, δεν μπαίνεις στην διαδικάσια να κάνεις εξτρα πράματα τα οποία ένα σε βοηθήσουν που την στιγμή που λειτουργούν

R:Σωστό, πολύ σωστό

A:Μέχρι στιγμής

R:Οκ, κάτι άλλο που ήθελα να σε ρωτήσω πολλά σημαντικό, τωρά εν περίοδο covid είπες μου ότι έτυχε να εργαστείς,πόσους μηνες είχετε τον θάλαμο με τα covid άτομα

A:Που τον Μάρτη που άρχισε το Lock down, πόσο πήγε, αναμίση δυο μήνες; Πόσο ήταν;

R:Ok, έτυχε είπες μου να εργαστείς με εκείνα τα άτομα, αν δεν κάνω λάθος και εννοώ, διόρθωσε με αν κάνω λάθος, για να μπείς στα covid, φαντάζόμαι βάζετε κάποια ειδκή στολή, μάσκες, την διαφάνεια μπροστά

A:Ναι

R:Τουτο πως τροποποιεί τον τρόπο που επικοινωνάς με τα άτομα, δηλαδή αμαν εν, σαν τωρά έχετε τις μάσκες τωρά και καιρό, ενώ πριν ασπούμε, επειδή έχει πέντε χρόνια που δουλευεις, πριν δεν είχατε τις μάσκες, δηλαδή εμπορούσαν να δουν το πρόσωπο σου τις εκφράσεις κλπ

A:Καταρχάς το να μπορέσει να βγει η φωνη σου εξω έπρεπε να φωνάζεις, γιατί είχες μάσκα η οποία ήταν πόσα εκατοστά παχία, με το φίλτρο μπροστά, να έχεις το shilt μπροστά αρα κόφκει τον ήχο πάλε, είχες την στολή την ολόσωμη που καλύφκει και τα αυτία σου, οπότε είχες τρία εμπόδια, καταρχάς επικοινωνία νοσηλευτη με νοσηλευτή και γιατρού, φαντάσου που έχουμε τις αισθήσεις μας

R:Ναι

A:Το να προσπαθήσεις να επικοινωνήσεις, δυστυχώς , δυο ηταν οι ασθενείς που είδα εγώ, τον Αναστάζιο που σου ειπα πριν και την Μαρίνα που ηταν το μεγαλύτερο διάστημα μέσα και ηταν και οι δυο ασθενείς που εφύγαν και εποικοινωνήσαμε μαζί τους, φωνές, εφκάλαμε φωνή και το να μπορέσουν ναα μας ακούσουν εεε και να μπορέσουν να καταλάβουν τι ζητούμε από εκείνους, εε τα υπολοιπ είρθαν έυκολα, ειρθαν ευκολα, ηταν εκείνα που σε περιεγραψα πριν, δηλαδή με τις κινήσεις, με το αυτό, έκααμε και ένα χρονικό διάστημα και οι δυο τους που ηταν αποσωλήνωτοι, δηηλα΄δη είχαμε καλύτερη επικοινωνία μετά ασχετο αν είμασταν ντυμέννοι εξωγίηνοι, που εφύγαν ηταν αποσωλήνωτοι, δηλαδή τούτο δια σου και μια ευχαρίστιση (κατι ψουψουρισε και δεν καταλαβα), αλλά, ναιι…

R:Οκ, μάλιστα, αρα χρησιμοποιέις τα ίδια στοιχεία που χρησιμοποιας και τωρά ασπουμε

A:Ναι ναι εν αλλάσει κάτι, το μόνο που άλλαξε ηταν η παραπάνω ένταση της φωνής γιατί δεν βγαίνει η φωνή σου

R:Παραπάνω ένταση

A:Δηλαδή εκείνη η περιόδος στα Γενικά, πάρα παρα πολλά δύσκολη με την επικοινωνία, γιατί ηταν γενική εποικοινωνία δύσκολη, ηταν απομονωμενοι σε δωμάτια, είχαμε κάμερες και είχαμε και μικρώφωνα και είμασταν εξω εμείς και παρακολουθούσαμε τους με κάμερες, οπότε πάλε εκείνο με τον ήχο που σου είπα πριν , με το μόνιτορ με τον αναπνευστήρα και το μηχανάκι κτλ, παρακολουθούσαμε τα που κάαμερες

R:Ειχατε φωνή όμως που τις κάμερες, αρα ισχυαν και τούτα οσο αφορα

A:Ναι ναι, δηλαδή αναπτύξαμε ένα άλλο τρόπο εποικοινωνίας και εμείς με τους συνεργάτες μας, ηταν άλλο πράμα εκείνο, αλλα μεσα στην απομόνωση δεν είχαμε τόσο πολύ καιρο, ηταν μόνο ο αναστάσιο που ηταν με τον σωλήνα

R:Kαι ξύπνιος, γιατί του πήρε τόσο πολύ καιρό να αποσωληνωθεί;

A:Δεν μπορούσε να βγεί, εν εμπορούσε ακόμα, εεε οοο κορονοιός έκαμε του τον πνευμονα

R:Πόσο χρονών ηταν το, εε εχτός της έρευνας τούτο, πόσο χρονών ηταν τοο

A:Ε 67;

R:Αα ε αρα ηταν μεγάλος, αρα αντιλαμβανομαι να ηταν τόσο πολύ καιρο ξύπνιος και με τον σωλήνα, ποσο καιρο πανω κατω μιλούμε, δηλαδή ποσο καιρο συγκεκριμένα;

A:Eεε εν θυμούμε συγκεριμενα

R: Αverage

A: Νομίζω ηταν πάνω που τρεις τεσσερις μέρες, δηλαδη νομίζω ηταν, ηταν…

R: Αρα ενας λόγος που τον αφήνατε διασωληνομένο και ξύπνιο ηταν επειδή εσυνεργάζετουν μαζί σας; δεν τραβούσε τον σωλήνα κλπ, ενώ σε άλλους ασθενείς ασπουμε, είπες μου, σορρυ εν θυμούμε, είπες μου εκοιμοίζετε τους αν δεν ηταν συνεργάσιμοι κλπ; Και επροσπαθούσαν να τραβήσουν τον σωλήνα, ή οι;

A: Eεε, κόιταξε όταν βλέπεις όταν είναι διασωληνομένοι και κοιμησμένοι και ξέρω γώ, την ώρα του μπάνιου πάεις να του γυρίσεις και μονομίας ξυπνουν και γυρίζουν χέρια προς τα πάνω, ξέρεις ότι εκείνος ο ασθενής έχει μια τάση να πίασει τον σωλήνα, οπότε συνήθως είναι δεμένα τα χέρια τους, τωρά σωστό ή λάθος πρέπει να γίνεται γιατι καλύτερα ναα είναι δεμένα τα χέρια τους παρα να τραβήσουν ένα φουσκωμένο σωλήνα και να κάνουν ζημιες στις φωνητικές χορδές ή γενικώς στην τραχεία

R: Δηλαδή δεν μπορείτε να τους δώσετε παραπάνω καταστολή παρά να τους δίνεται τα χέρια τους;

A: Ναι αλλά θέλεις να ξυπνήσουν, θέλεις τον ναα ξυπνήσει και που την άλλη σε κάποια φάση, όταν το φορτώνεις πάρα πολλές καταστολές, όταν είναι σε πάρα πολλά, σηπτικη καταπληξία ή ένα σου επηρεάσει νεφρά ή να σου επηρεάσει καρδιακή παροχή κλπ, οπότε αν τα νεφρά σου δεν κάνουν κάθαρση, κυκλόφορει ψηλές δόσεις dormicum Πχ, αργεί να μεταβολιστεί και τα νεφρά σου δεν το μεταβολίζουν, οπότε κλίνεις το dormicum και βλέπεις ο ασθενής σου δεν ξυπνά, γιατί δεν ξυπνα; Γιατί δεν μεταβολίζει το dormicum, κυκλοφορεί το dormicum, ξανακοιμάτε και ξερω γω, οπότε μειώνεις μειώνεις μειώνεις αφήνεις το να μεταβολιστεί , όταν και εφόσον μεταβολιστεί για να ξυπνήσει, φυσικά υπάρχουν και τα Seroquel που εν χάπια και μέσω του εντέρου κλπ και τούτα ούλα που τους καταστέλει για να εν πιο ήρεμοι και να ξυπνήσουν πολλά πιο έυκολα

R: Μαλιστα

A:Οπότε μειώνεις τις καταστολές τις ενδοφλέβιες και ξεκινάς και δίνεις ασπούμε που το levine, τον ρινογαστρικό, το Seroquel που δίνουμε συγκεκριμένα, σε συγκεκριμένες δόσεις, εξαρτάτε που τον ασθενή και τα κιλά του κλπ, εε και την συχνότητα, δηλαδή μπορεί σε ένα ασθενή να του δίνεις μόνο την νύχτα άλλους τρέις φορές, άλλους τέσσερις φορές, εξαρτάτε που τον ασθενή, ε τούτη όλη συγκέντρωση κατασταλτικών , πρέπει να δεις αμπα και εάν ενας ασθενής είναι με πολυοργανική ενοείται εν θα τα μεταβολίσει, εν τούτο το προβλημα, εάν ασθενής λόγω του μεταβολισμού μπορεί και να μην ξυπνήσει και πιο νωρίς από ότι τον θέλεις

R:Οκ, εσείς είσαστε ενας προς ένα συνήθως

A:Συνήθως ναι

R:Εντάξει, κάτι τελευταίο γίωργο μου που ήθελα να σε ρωτήσω, είπες μου ότι δουλευεις πέντε χρόνια στην εντατική, τον καιρό που μώλις πρωτομπήκες, πριν να έχεις τόση εμπειρία μέχρι τωρά, πιστεύεις ότι εξελίχθηκες ασπούμε οσο αφορά το πως διαχειρίζεσε τούτου του είδους τους ασθενείς

A: Nαι ναι

R:Βλέπεις κάποια διαφορά έντονη ασπούμε, δηλαδη

A:Ναι

R:Που δεν έκανες πριν και κάνεις τωρά; να μου δώσεις ένα παράδειγμα,Παράδειγμαα, όταν δουλευεις..,Η ενοια σου ηταν, ηταν οι ιδιες οι εννοιες σου την αρχη με τωρά

A:Όχι, η εννοια μου όταν επρωτοπηγα στην εντατική ηταν να μεν κάμω ματσραγκα (γελια) γιατι όταν μπαίνεις να δουλέψεις και έχεις να κοντρολάρεις αναπνευστήρα μόνιτορ, μηχανάκια τα οπόια πρέπει να είναι ειτε συγκεκριμένες δόσεις, ένα βλέπεις παραμέτρους στον αναπνευστήρα κτλπ, να βλέπεις συνολικά ένα ασθενή από την κορυφή μέχρι τα νύχια για να καταλάβεις τι τους συμβαίνει, τούτο θέλει χρόνο

R::Αρα έκανες εστίαση σε τούτα τα πράματα

A:Επικεντρώνουμουν στο να μάθω γρήγορα το τι πρέπει να κάμνω για να το κάμω σωστά

R:Μάλιστα οκ

A:Γιατι από ότι σου είπα, πήγαα με στόχο στην εντατική να μάθω πράματα, εν τα έμαθα, εννοείται ότι εν τα εματα, απλα θεωρώ τωρα ότι, είμι καλύτερος νοσηλευτής ότι μπορώ να δώκω σε ένα ασθενή πραματα τα οποία θέλω και εγώ που μόνος μου να του τα δώσω

R: Μάλιστα οκ, αρα ενοείς, αν το καταλαμβαίνω εγω σωστά , επειδή αναπτυχθήκαν και τα skills σου οσο αφορά την εντατική έχεις περισσότερο χρόνο ας το πω να ασχοληθεις και λιγο με το

A:Ναιι

R:Με το επικοινωνιακό κομμάτι;

A: Tούτο συμβαίνει τα τελευταί δύο χρόνια, τα πρώτα τρια χρόνια ηταν για να βεβαιωθώ ότι ξέρω τις βάσεις και τωρά μπορώ πάνω στις βάσεις να ανεβώ

R: Μάλιστα, είπες μου πάρα πολλά ωραιες πληροφορίες angelos, σε ευχαριστώ πάρα πολύ ,εεμμ το εκτιμώ ιδιαίτερα που είρθες……μπλα μπλα

NO 6

R: Researceher

A: Angelina

R: Okay,Angelina . Sorry but I have to record you in order to be able to listen to later what do what we spoke about right now.

A:
It's fine.

R:
First of all, I want to say a big, big thank you. Because I'm very appreciative that you took from your free time to come and speak with me.

A:
Oh bless you, it’s not a problem and thank you for my tea. (laughter)

R:
Thank you very much and secondly, I'm very grateful that you have, because I want to, see the opinions of many kinds of nurses. As I told you, am I doing a research about the communication experiences of the nurses who are working in the intensive care unit with non-sedated ventilated patients?

A:
Ah, Okay,

R:
I'm talking, right now to you. This is all between us, no one would write down your name, your name wouldn’t be found written down by someone or…

A :

I trust you

R:

Okay, this information here is only for my research. It is confidential. The only ones that can have access to it, are my professors. Okay?

A:

no problem.

R:

Additionally, I want to tell you that I am sorry for the inaccuracies in my English, and if you don't understand something, let me know, okay? And if I ask you to repeat something again, one more time, don't be mad with me, okay?

A:

Don’t apologise, and if you can't understand my Irish accent, then you can ask me to repeat myself, because my Irish accent, is difficult

R:
No it’s okay. And also, I want to tell you that there are no, wrong or correct, I mean, false or true answers to respond with, I just want you, to say to me, only your personal opinions, okay?

A:

It's fine, I understand.

R:

It’s very important to me, and the more you tell me, the better it is for me. I mean, anything you want to tell me you should tell me, okay? I'm going to ask you a few questions. So, I'm going to… How do I say that in English? Lead the conversation.

A:

Υes

R:

Yes, I'm going to lead…, you understand what I mean? Okay, A:e. First of all, I want to ask you about the cases in your intensive care unit, do you have, awake, non-sedated, ventilated patients?

A:

Quite often we have them, we have the ones that end up with tracheostomies, you mean like the tracheostomies?

R:

No, no,

A:

Oh, you mean like intubated.

R:
Intubated, only intubated. When do you see these patients?

A:

Not so often now...

R:
Okay.

A:But I guess with the… when we were busy with cardiac,

R:

yes,

A:

Sometimes, they're not quite ready to extubate, so we keep them up without sedation.

R:

Okay,

A:

maybe sometimes, if they're, depending on how much they tolerate the tube, it can be for a few days almost, which is a problem. It's difficult because obviously, they are not liking the tubes,

R:

okay,

A:

So… you're trying to balance keeping them from being too weak, but also reassuring them and stopping them from pulling out the tube as well.

R:
Okay, so do you mean that you have only cardiac patients in your Intensive Care Unit?

A:
More so cardiac, now we tend to have more of a mix, they’re taking maybe sometimes from the General Hospital if the General Hospital Is full , they will give us patients maybe that are intubated, and they want to wake them up to see if they can extubate them, before they take them back to the general again. So they will come intubated. And then our doctors will assess their sedation, assess their medical needs, and see if they are safe to start thinking about extubating them without needing to put a tracheostomy in.

R:

Okay,

A: And that does happen sometimes.

R:
So is this during the weaning time? Because I'm not working in the intensive care unit. When do you see this? I'm not not talking about only the cases, I mean, at what time would you see a non-sedated patient with a tube in? Only during the weaning prosses or on other occasions as well?

A:
It depends, like I said it's up to the doctors to decide. Because usually when you wake up a patient with an E t tube in,

R: yes

A:

you don't have that long to keep them intubated, because they will not tolerate like,

R:

okay,

A:

If it was your me. We are very neurologically alert hopefully. And we are very young, we're quite young. So, you don't have days to keep somebody awake with a tube in. unless they have a neurological problem. Or if they've had a stroke. If they are, if they are fully alert and orientated. You're talking maybe a couple of hours before they will start to want the tube out because it's bothering them.

R:

Okay,

A:

But if they've had like maybe a stroke or something that affects them neurologically, they will tolerate the tube better. So you can keep them awake without sedation with the tube in for longer, but if it was like a straightforward operation, and you were waking somebody up after the operation like cardiac surgery or gastric maybe surgery, then you have to be reasonably effective in waking them up and getting them off the ventilator, because otherwise they will start to want to pull the tube out because it bothers them.

R:
Okay,

A:
It makes them want to gag because it's right down the back of their throat sitting about here.

R:

Okay,

A:

So, it bothers a normal person. If they're not in any sedation, it will start to disturb them.

R:
Okay,

A:
Did that answer it? Did that make sense?

R:

Yes, of course

A:

Am I answering your question?

R:

Yes, yes, yes. Yes,

R:
If I have in any further questions, I am going to tell you. How is it to work with a non-sedated and ventilated patient?
How's it for you?

A:

For me, I would find it more difficult here, because of the language.

R:

Okay.

A:

But like I said, I would probably say I am more effective with speaking in Greek medically, because I've learned what to say, you know, not to worry, things like that, we’re beside you. So I know the terms say and great to try and reassure a patient. I would say it depends on the patient, some patients are very calm,

R:

okay,

A:

I think if so, if you got a patient who's maybe come in with a head injury, so they don't remember why they were intubated in the first place, then it's more difficult, because you're trying to tell them what has happened. If they've had an operation, they know to expect to have the tube in their mouth. But if somebody maybe fell off a ladder, or had a head injury, for whatever reason, they will not remember the situation that brought them to the intensive care, and then they panic. So, it's quite important to try and reassure them as quickly as possible as to why they're there and what the plan is and what to expect,

R:
okay.

A:
And quite often then, a lot of them, like touch as a form of communication, just to, to reassure them to be holding their hands so that they don't feel that they're by themselves. Because the problem with the intensive care is, you know, yourself, the rooms are big. If you have a tube in your mouth, you can't look sideways, you kind of feel like you're stuck on the bed. So I think it's important that they make eye contact that they have eye contact with somebody. And that's difficult now, as well, because of COVID. With the masks, they can't really see your lips. It's your eyes that they can see, isn't it?

R:

Yes.

A:

And that can be quite scary as well, if you suddenly are surrounded by people with hats on with masks with, gowns…

R:

Yes Okay.

A:

So, you're, you're trying to reassure them and not freak them out. Because, you know, it's a very frightening environment to be in intensive care. And sometimes the thing I find is, and this is what I would say to the students, is that we get very used to the alarms to the noises. But if you have somebody that's just come into intensive care as a patient, things that we take for granted that don't bother us and disturb us, like the pumps, alarming, the ventilator alarming, that could be very disturbing for a patient. So, I think it's important that you try and reassure them, the only things they are hearing that they're seeing as normal, and that can help to calm them down as well.

R:
Okay, you're giving me a lot of information, and I want to grab on each one separately.

A:

Okay,

R:

Firstly, because I was very excited at the beginning, I forgot to ask a few more questions about you personally and your personal life, how old are you, A:e?

A:
I'm 45.

R:
And how long is your experience as a nurse?

A:
But I'm gonna sound really old now. I graduated when I was What 22? I did my degree. So, what 22 - 23? So it's been many years. Do the math. It's a bit depressing, really, isn't it?
It's about 20,23, 24 years

R:

24 years in a nursing career?

A:

Yeah.

R:

Okay. And how long have you been working in the Intensive Care Unit.

A:
I haven't worked in intensive care all the time.

R:

Okay.

A:

I went into teaching for quite a while for about five years.

R:

You went?

A:

into teaching. I was a nurse trainer in England.

R:

Really, Wow. That's pretty good.

A:

So, I taught medical students and nurses and I did I was a resus trainer for about a year, two years. But I didn't like that

R:
You didn't like the research?

A:
I didn't…, No the resuscitation. I didn't like the resuscitation of patients because it wasn't always a good outcome. So, I did that for maybe about a year and a half, two years resuscitation training as a resuscitation officer.

R :
Okay,

A:

in the hospital,

R:

Is it something like ALS here in Cyprus?.

A 9:43
Yes, Yeah, you have the trainers that train the nurses train the doctors and then they go for the resuscitation. They're part of the resuscitation team.

R::

Okay.

A:

I think I've probably done the intensive care for about 15 years out of 23.

R: 10:00
Okay, that's very good.
Where did you study?

A 10:01
At Lancaster, and that is my nursing degree at Lancaster University in England. So, I left Ireland and went to England, which was very brave back then there was no social media and there was not internet, it was very brave, in those days,

R: 10:15
okay, did you do anything else apart from your bachelor degree?

A 10:21
I did my PGCE. I did my teacher training after that, but I did it part time while I was still working. So I did it in the evenings. They were very good. You knew what it's like, you can do your placements. You can do your training as part of your nursing. So, while I was doing one of my teaching jobs, I was able to use that for my PGCE as well. They were able to put the two together.

R:

Okay,

A::

so, I, I did, I did it at night college.

R:

Okay,

A:

Two or three, nights a week, and then maybe one or two days a month, I went to college full time. And then I also did it as part of my nursing. So, I was very lucky, I was able to do all as not to come out of nursing to do I was able to incorporate into my nursing to do it.

R:

11:03
Okay, okay, that's very nice.
So, how many years have you worked in England, in intensive care unit? If you remember,

A: 11:14
I’m not very sharp now, that’s the problem. I'm so old now

R:

11:16
Average I mean It’s not.

A: 11:17
I think I did about seven,

R: 11:20
seven years in the intensive care unit?

A:

Yes.

R:

Okay. And now how long here in Cyprus now?

A: 11:26
12. Part time, though. I was I was full time in England. I'm part time in Cyprus.

R: 11:33
Okay. so, you told me earlier that it's different in the intensive care unit, because there are a lot of noises, for e.g., the pumps that are ringing, so is that an obstacle for you? For the, communication between you and the patient?

A: 11:54
I feel awkward for the patient. But like I said, it's a conflict because you don't want to upset your colleagues, because I get along really Well. Everybody's lovely. I think in general, maybe it's a cultural thing. Cypriots don't realise how loud they are,

R: 12:07
I didn’t understand sorry tell me again.

A::

It's a cultural thing.

R: 12:09
Yes,

A:

Cypriots are very loud as a nation.

R:

Yes.

A:

And I think possibly, because obviously, in the UK, they have done a lot of noise studies in the UK, and the effect of noise on patients and sleep deprivation in the intensive care. And I think as well, sometimes because a patient is intubated, you forget that they can hear.

R: 12:31
Yes, that's true

A: 12:32.

And this is the one thing I would always say to new nurses, the last sense to go. And the first thing is to come back as hearing so so the patients can often hear but maybe cannot tell you that they can hear things. So it's important in my mind, it's the one thing I hope I never have forgotten is to respect a patient that they can possibly hear. Because I think that's so important. Even if they're dying, even if they're unconscious, I would never say something across the patient that I wouldn't want them to hear. And sometimes I have actually said to other other nurses or other people, you don’t know if they might be able to hear that. So just, you know, don't say it.

R::

Okay,

A:

I think that's really important.

R: 13:12
Okay. So, let's take it from the beginning, how do you communicate with these people. I’m refereeing to the, non-sedated ventilated patients.

A: 13:22,

a lot of touch,

R: 13:23
A lot of touch…

A:

A lot of touch, I would say as long as you feel that they are comfortable with that you've got to see sometimes patients may not want touch, but I think generally speaking and intensive care. Even if they can't understand what you're doing, if you're cleaning their eyes, if you're cleaning their mouth, if you're like washing them, I think I think it probably gives them some comfort that you're doing very basic things for them. But I think for if I put myself in their shoes, I would want my mouth to be clean, I would want my eyes to be clean, and they can't understand what else we're doing with the pumps with the dialysis but they can understand that you're giving them that you're cleaning their eyes or that you're helping to clean them. And I think that it's important for a patient that maybe hasn’t got a full understanding because of maybe sometimes this sedation that we give takes a while to wear off or they could be on morphine. So, they may not be fully able to understand what has been done but I think as long as you show compassion, through touch maybe just through eye contact even I think I think eye contact is quite important.

R:

Okay,

A::

yeah. And you gauge you are on the monitor, you can gauge their reaction looking at the monitor, you know, if their heart rate is going up, you know, you can gauge maybe they're uncomfortable with something that you're doing so you can try and explain better what you're doing. Or you can ask them as well if they've got any pain because usually, they can’t manage to communicate with you if they have pain and you can reassure them, we're going to give you something for analgesia you know, παυσίπονο and hopefully reassure them that you're looking after them.

R:

Okay

A::

I think

Am I waffling? Am I talking too much?

R: 15:09
No, I just want you to talk too much really; I want you to talk too much, you’re giving me a lot of good information that I want to hear.

A: 15:19
I think possibly and this is going to show my age. But it's what a lot of us would say even in England as well, not just in Cyprus. There's so much technology here, nowadays with intensive care, especially

R:

Okay,

A:

that sometimes especially if you're a new nurse, it's very difficult to balance the technology and understanding the medications and the pumps, but also with the patient's psychological needs.

R: 15:45
So you mean this is an obstacle because your mind is on the technology?

A: 15:52
Yes, I think so It's very hard to try and remember to do everything.

R:

Yes.

A::

And I think maybe that comes with experience that you then can relax a little bit from the technology, you know, getting the drug medications, right, the infusions, the blood gases, looking at the ventilator, when you are maybe a bit more experienced then you have the ability to look a little bit more at the patient.

R:

Okay,

A::

and see that maybe they have need separate to the, you know, the medicines that

R: 16:18
The thing that you do?

A:

Yes,

R:

you have to do,

A:

yeah,

R::

Okay. Okay. What I wanted to tell you? So, what do you think that these patients want to tell you when they are awake during that time?

A: 16:40
When they're still intubated? You mean?

R:

Yes

A::

I think they wanted to ask us, when will the tube come out?

R:

Okay.

A::

And that is something that we… and also where are they? Because they may be disorientated even though they may be prepared before the surgery. But if it's if it's a planned intubation,

R:

yes,

A::

I think probably maybe they want reassurance that the operation has gone well,

R:

yes.

A::

That everything went as was planned, there were no problems with the operation, and that we are planning to take the tube out as soon as we can. As soon as we know that they are able to breathe by themselves. I think they need a lot of reassurance on it, you find yourself repeating yourself a lot.

R:

Mm hmm.

A::

And sometimes I think Gosh, they must be bored of hearing me saying this but they seem to take reassurance from being told the same thing from being reminded

R::

Yes,

A::

of the situation, and that everything is okay. And that they will you know, we will take the tube out as soon as we are happy that they're breathing by themselves. And also, the reassurance we're asking them Have you got any pain?

R::

Okay,

A::

are you comfortable?

R:

Yes.

A::

Is anything, you know, and you do learn to lead them with the questions because obviously they can't tell you

R:

Yes,

A::

Cause they can't get up to say if something bothering you they will know just have you got pain is it your position do you want to sit up the bed, is your mouth dry, very often even with the tube in their mouth gets very dry, so we can get you know the little sponges of water and you just wet their mouths. While the tube is still in obviously, they can't swallow.

R:

Okay,

A::

But we can put a little bit of water on the sponge and just wet their lips and their mouth and I think all these things give them reassurance

R: 18:23
okay,

A: 18:24
because it's a very alien environment intensive care

R: 18:26
Its very?

A::

Alien

R::

Alien okay

A:: 18:29
even as it like, if you think as a student nurse or as a new nurse coming into it, it's very different to the wards or to other environments. So, I think for patients, you have to remember that it's completely strange for them.

R::

Okay.

R: 18:46
Okay, what do you think are the obstacles for you to be able to communicate with these patients. The ones that are intubated

A:: 18:54
obviously for me the main obstacle would be the language

R::

the language

A::

The language for me and that also stress me because I know that if I'm especially if I'm if I'm looking after very often, they give me the English-speaking patients.

R::

Yes

A::

Because they know that I can communicate

R::

Okay, yes,

A::

that's not to say that the Greek nurses English is are fantastic. It's really a high level.

R::

Yes.

A::

The nurses that work on you know your English and all the work nurses English is excellent. But they will often give me the English speaking nurses or English understanding nurses. Because I think that's important for the patient to have confidence in the nurse that's looking up to them that they can speak their language and understand their needs

R::

Okay,

A::

that would be the biggest obstacle. Sometimes if the patient is on like strong analgesia like morphine,

R::

okay,

A::

sometimes it can cause them to be a bit confused so they may not be on sedition, such a strong propofol or midazolam

A::

to knock…, you know, to make them asleep

R::

Yes, yes, yes, I understand

A::

but sometimes if they're on medication for pain relief, they can be a little bit confused and that is an issue. Because they're wanting to pull the tube out

R::

yes

A::

Because they’re not following that, and especially elderly patients,

R: 20:06
Yes, okay

A:: 20:06
they can. They can, they can be quite difficult. You know, if they've maybe they've been intubated for a couple of days. So they have a lot of buildup of sedation. If they've had a lot of blood products

R: 20:19
Couple of days, sorry that I interrupt you

A::

No, its okay,

R::

no, no, keep going, I'm going to ask you later

A: 20:24
going through like,

A: 20:25
well, just if like they had like a lot, a lot of products of blood, they're a bit confused, that can be an obstacle to communicating with them, because they’re not listening, they are in their own world, we're trying to wake them up. So, they're off their sedation, but they're not ready to extubate because they're confused. And they can be trying to get out of the bed with the tube in. And that can be difficult, that can be really difficult, for us.

R: 20:50
How do you feel about that, if you can’t communicate with this kind of patients,

A: 20:55
 It’s a bit, you get a bit nervous, because you're worried that you're going to turn your back on them for a second, and they're going to pull the tube out. And that's makes you quite anxious, because then obviously, you don't want to compromise their care. And obviously, if they pull their tube out, and they're not ready, it can be a risk to their care. So it's getting the balance. Sometimes we have somebody else rang one of the doctors to say can we start the sedation again?

R::

Can we start?

A::

Start the sedation again

R::

Yes

A:

Because they're trying to climb out of the bed.

R::

Okay.

A::

And I think that's that's hard getting the balance trying to decide when they become unsafe with being still with a tube in, but they're unsafe to keep them with a tube in, but without any sedition.

R: 21:37
Okay, so you find that your main obstacle… I'm talking about the language right now. You said to me that your biggest obstacle here in Cyprus, the main one, is the language. Do you find the same obstacle when you communicate with these kinds of patients? Both in English or in Greek? I mean, in exception to their language? Or do you have…

A: 22:00
probably no, more so the language would be my my personal biggest obstacle.

A: 22:05
But like I said, sometimes the other obstacles can be the patient is even though they even with the nurses that speal Greek you see it. They they're not listening; they don't understand they are awake. They're breathing by themselves, but they still have the tube in, but they're not understanding what they're being told because they're not ready yet to come off the ventilator. But sometimes, understandably, so doctor said earlier, our surgeon, he doesn't want to put the back on sedation. So, you have to just try and stay with the patient, and just sit with them or stand with them and just try and keep them safe. That becomes your priority is keeping them safe from harming themselves.

R::

Okay, okay

A::

Rather than me trying to communicate so effectively with them, sometimes, you can tell they're not listening to what you're saying. So you just have to try and keep them safe. And that means sometimes staying with them, a nurse on either side, and keeping them from pulling, sometimes they try and pull their central lines, they pull the feeding tubes, or they pull the things that they feel that are bothering them.

R: 23:11
Okay,

A: 23:12
so it becomes a physical rather than an emotional, you know, demands that you're trying to meet, you're trying to meet their physical demands, in keeping them safe.

R: 23:21
Okay.

R: 23:24
Okay, what else I wanted to ask you? allow me to see my questions please, to see if I forgot something.

A: 23:33
Ooh, hang on, let me just text my daughter and let her know that five minutes late, hold on.

R: 23:41
Do you use any strategies to speak with these patients if when they can’t communicate, or?

A: 23:46
if they can't talk? Yes, sometimes they can write things down,

R:

okay,

A::

So, you get them like a piece of paper on a clipboard. And they can write things down. We also have the iPads. And sometimes if they are, we can get them to type what they want to say. Or you have these picture boards.

R: 24:06
You have those in your unit?

A: 24:08
Yes, we have picture boards, which we don't we probably don't use them as much as we should do. But they have like the key phrases that they can point to in Greek or English I am hot. I am cold. I have pain. I am I have I am anxious so they can just point out what is bothering them. And then so that's a lot easier if they have sometimes what they're trying to ask us isn't on these boards. So then you have to ask, is it are you wandering about your wife? Are you wondering about your husband? So, you have to try and think of other things that might be bothering them, or they want to know about

R: 24:44
Okay, and

A: 24:47
I am this listening, keep talking. I'm just sending a text.

R: 24:49
Sorry.

A: 24:50
No, it's okay. Keep going. Keep going. It's okay I’m listening

R: 24:57
Why don't you use the tablets or the clip boards or anything like that as often as you would like to?

A: 25:07
I guess sometimes because we don't think to use them, we just don't think and sometimes trying to find them. As you know, things aren't always where they should be.

R: 25:16
Mm hmm.

A:

So sometimes

R::

trying to find them and not find them you mean,

A:

yes,

R::

okay.

A:

Exactly

A: 25:24
If you're busy, sometimes you might have two patients. So the time it takes for you to try and find something, it's time that you could be doing a blood gas or giving medication to the other patient. So admittedly, sometimes we don't always invest as much time as we should do and trying to sometimes it's easier, Just to ask them verbally, you know, έχεις πόνο or, you know, είσαι κρύος, είσαι ζέστη, you know, you're trying to ask them the basic questions yourself, and then they will either nod or shake their head.

R: 25:57
Okay.

R: 25:59
Okay.

R: 25:59
Do you have any special experiences you would like to tell me about? Or a particular case that you would like to talk to me about or something that you remember, that you are still think of, or anything else? It doesn't have to be special.

A: 26:17
There's one out she was quite recently had a lovely lady. She's American.

R::

Yes,

he's American?

A: 26:22
She's American.

A: 26:23
She she was young. I say young. She was my age. And she had a subarachnoid bleed.

R::

She has?

A::

a subarachnoid haemorrhage,

R:

yes,

A::

She came in with a horrific headache. And the next thing she knew she was going for surgery. And lovely Actually, she made a full recovery. But she said when she first woke up because of COVID, she hadn't been able to see her husband. He wasn't allowed in with her. And when she first woke up, all she could hear was the Cypriot voices. And she said to me, afterwards, she said, that was so hard because she didn't speak any Greek. And she said, then I heard your voice. They asked me to go in, because they were trying to extubate her. She had been quite confused, quite disorientated. And she said to me, after she said, it was so lovely to hear an Irish voice to recognise it as an Irish voice and to hear English as a first language. And she said, that was incredibly reassuring for her. Because it just meant she said, I knew that if I had a problem, there was somebody who I could speak to who would understand. Because sometimes you know, yourself, sometimes she will say things in Greek, that don't translate into a different language.

R::

Yes

A::

You use slang, words like for em... that are Cypriot that don't exist in any other language.

R::

Yes, yes.

A::

And she said, that was reassuring for her. And even after I looked after her quite a bit, as she recovered from her surgery after she was excavated, as well. But she said it gave her reassurance to know there was somebody there, who understood and would be able to communicate in a first language, what her needs were when she was extubated.

R: 28:03
Okay. And the last thing that I want them to ask…, you have to go?

A: 28:08
No, it's okay to finish.

R: 28:09
And another thing that I would like to ask you is; You mentioned something about the COVID pandemic. What is the difference between the non-COVID period of time and the COVID period of time, in regards to communication with the patients?

A: 28:21
The masks, I think you don't realise how much people look at your lips when you're talking to them. And especially when it can be very noisy, so maybe your hearing is not so good, because of the pumps or the machines or people talking, I don't think he realised how much reassurance should take from looking at people's lips to talk. And now you have these masks and the hats, and I think it's the eyes are really important now to make eye contact all the time to be trying to look at a patient and make eye contact with them because they can't see if you're smiling anymore. You know, they can't see if you're smiling or what your facial expression is, because you have the masks on. So, it's important to make eye contact and to try and be reassuring with your eye contact.

R: 29:09
So, do you do something differently, when you wear the mask and the COVID gowns and other personal protective equipment, in order to communicate with this kind of patients?

A: 29:20
I think I would touch more,

R::

you will touch more. Okay,

A::

I would like to sort of go for somebody's hand and say to them in Greek, my name is A:e,

A: 29:28
don't worry, I am looking after you today. And I would be more aware of the need to do that as long as they're comfortable with it. And also with eye contact as well. I think eye contact is really important and to spend if you have the time to spend a little bit longer with a patient to reassure them because you can't smile at them anymore like you used to. You know and it's difficult I think for patient is can be very difficult now,

R: 29:58
Yes okay, so the time is an obstacle to ah,

A: 30:01
yes, sometimes we can be very busy. Sometimes it's hard to make the time to reassure, I think you do need a little bit longer now to reassure patients because you don't have the ability to smile.

R::

Okay,

A::

you know, to look at them and smile and reassure them. I think, like I said, if a pump alarms, they panic because they don't know that it's just because they've moved their arm.

R::

Yes,

A::

so, they get anxious. So, you have to remember to turn right to them and say, don’t worry, it's nothing. It's just the monitor, it's not a problem with you.

R::

Okay,

A::

things that we take for granted can make a patient very anxious.

R: 30:36
Bravo A:, I'm very proud of you.

A::

Did I do good.

R::

Yes. I also have two more things before you go. At university while doing your bachelor’s degree and/or later on during your…

A::

studies

R:

studies Sorry,

A: 30:57
No, don't ever apologise please.

R: 31:00
Did you have a lesson about communication with people?

A: 31:06
Yes, yes, we did a lot with communication. And actually, funnily enough, I did my many years ago, when I was doing my dissertation for my degree. I did it on the role of communication with cardiac rehabilitation patients, and the importance of communication, effective communication, and all the different ways of communicating with patients, when they're doing a cardiac rehabilitation. Because, you know, as you would know, I think it's lacking in Cyprus, the, the heart attack, or whatever it is, the surgery, the heart is just the start of it. It's just the beginning, you have the whole fear, the anxiety, everything that comes after a heart attack or heart surgery that beyond the certainty of, you know, and in the UK, they have a lot more emphasis on occupational therapy and physiotherapy and cardiac support cardiac rehabilitation nurses, it's a much bigger role. And I think it's so important communication to reassure patients, whatever they've had done, you need to effectively communicate it in order to, to lessen their anxiety, whether or not it's an intensive care for whatever it is, they've had a stroke, whatever, they need effective communication, to reassure them.

R::

Okay,

A::

and that sticks with me the whole way through whatever job and whatever patient I look after. I think it's it's vital good communication.

R: 32:25
Okay, so you believe that these lessons are still helpful for you afterwards?

A: 32:29
I’m sure they've stuck with me, Yes, I'm sure they have on the fact that some patients have kept in touch with me, after I've looked after them, like people might, you know, women my age, I thought she made a couple of good friends for looking after. And they've always said the one thing they valued more than anything, is somebody to talk to them to reassure them. Because you forget, when you work in a hospital environment, you forget how strange it is. And it's not as if a patient can appreciate that you've managed to adjust their lasix infusion or their actrapid infusion, they don't appreciate the medical things. They appreciate the things that they can understand, like the communication, the time that you spend with them or talking to them and reassuring them that as what they appreciate more than the fact that you've managed to stop the norepi that they're on, you know that you've managed to get them off their blood pressure medication. They appreciate the basic things more.

R::

Yes. Okay.

R: 33:24
Final thing I promise…

A::

No way, okay, I'm talking too much.

R::

No, no, no, no, I like it that you're talking too much. I wish that I have too many like you to speak with me

A::

Aww bless you,

R::

Did you take any lessons about communication, specifically with this kind of patients? I mean, with intubated, non-sedated ventilated patients? Not with ordinary patients that are more able to speak.
A: 33:51
I think we were made more aware of that possibly in England. Yeah, when we were doing our orientation, we and I did the intensive care course, which was like a six-month course to give you like, you know, and I think they probably touched on it there and the importance of communication and intensive care with the family, not just with the patient, but with the family as well. We had a module where they focused more on the communication as part of it not only but the needs of the intensive care patients, the needs for effective communication. I think it was more I don't know if they touch on it here as much but I know in England, it became very important as part of the training when you're doing intensive care courses. And that's why now as well they also I know they do mention a lot the effect of noise on intensive care patients at nighttime and the levels of noise because they realise how important it is for patients. And they've done a lot of work now in the UK. They now have a lot of follow up clinics, patients that were in the hospital, they now have the nurses that do follow up clinics, to phone them, the patients to see how they're sleeping. To See what… once they get discharged from, because a lot of them suffer from Post Traumatic Stress Disorder. And they have issues with sleep with anxiety with disruption of their, you know, their psycho psychology is very screwed up. So they're now trying to deal with this. Okay, a lot of these patients go out of intensive care, and they have a lot of issues when they leave intensive care. And I'm sure it's in here. They just have the studies in place yet

R: 35:28
Okay, if you had the opportunity to do it again, to have a lesson about that, wouldn't you do it?

A: 35:33
I would. And I think I think it should be emphasised more for the nurses are starting in intensive care.

R: 35:39
Okay, if you had the opportunity to choose, what type of lesson would you like to take? In what setting for instance, right next to the patient? In a classroom or by the bedside, or both of them?

A: 35:51
primarily, I think, I think it should initially be in the class.

R: 35:55
Yes.

A: 35:56
Because if you're doing it by the bedside, you're doing it by the bedside you doing it with patients that can hear.

R::

Yes.

A: 35:59
So I think that they shouldn't hear everything that you're saying, because some of it might not be good news, you might be saying in the classroom, the patient is dying. They're intubated, they're dying. They shouldn't hear you saying that the bed space, I think that the bed space should just be making reminding them, the nurses that patients can possibly hear when you don't realise, they can hear and it's important to be aware of that and speak to them as if they can hear not to be saying things that you wouldn't want them to hear.

R: 36:29
Okay, Thank you very much, A:e. I appreciate that.

A: 36:33
No it was lovely to talk to you if you have any questions, just text me, I don’t mind.

R: 36:35
Okay. Thank you very much for coming. I'm very happy about that, thank you for all the information, that you’ve shared with me. Thank you.

A: 36:41
If you have any questions…

Συνέντευξη Νο 7

R: researcher

L: loizos

**R: Loizos όπως σου είπα , δεν ξέρω αν σου είπε ο, βασικά εγ δεν σου είπα ακριβώς τι κάνω, είπε σου ο φίλος σου φαντάζουμε ότι κάμνω μια έρευνα όσο αφορά την εμπειρία των νοσηλευτών που δουλεύουν στις Μονάδες με διασωληνομένους ξύπνιους ασθενείς. Όπως σου είπα και πριν λίγο ότι πούμε είναι μεταξύ μας , δεν έχει σωστό και λάθος, εγώ προσπαθώ να δω ούλλες τις οπτικές γωνίες διάφορων νοσηλευτών να δω τι σκέφτονται και τι αντιμετωπίζουν γενικά , όσα πιο πολλά μου πεις τόσο το καλύτερο για εμένα, δηλαδή όσο πιο πολλά ανοίγεσαι και πιο πολλά μου πεις εν πολλά κα΄λό για έμενα, εννοώ μεν σκέφτεσαι ότι μπορεί να φλυαρείς κλπ, εντάξει; Ένα σου κάμω μερικές ερωτήσεις αρχικά όσο αφορά εσένα**

L: Μάλιστα

**R: Πόσο χρονών είσαι;**

L: 40

**R:40 ok, πόσα χρόνια εργάζεσαι ως νοσηλευτής γενικά;**

L:Από τον Γενάρη του 2006, έχει 14 χρόνια , πάω για 15

**R:15 οκ, σχεδόν, ως νοσηλευτής της μονάδας πόσο καιρό είσαι;**

L:Που το 2012

**R: Που το 2012, 8 χρόνια, οκ**

L:Έκαμα ένα διάλλειμα 2 χρόνια που δούλεψα στο πνευμονολογικό

**R: Χμμμ, οκ, εε, που έχεις τελειώσει τις σπουδές σου;**

L: Η ηλικία η δική μου ήταν της σχολής της Λευκωσίας και εκαμαμε εξομοίωση μετά στο ΤΕΠΑΚ.

**R: Οκ έκαμες κάτι άλλο μετά που τούτο; Έκαμες κάποιο μεταπτυχιακό ή κάποια εκπαίδευση; Όχι απαραίτητα μεταπτυχιακό**

L: Οι

**R: Οι οκ, γενικά πως είναι εργάζεσαι στην Μονάδα με τέτοιου είδους άτομα, διασωληνωμένα και ξύπνια;**

L: Πως είναι ;

**R: Nαι**

L: Εμένα η φύση της δουλείας αρεσκε μου πάρα πολλά, η εντατική και το ότι έφυγα που το 2012 ως το 2016 πήγα δυο χρόνια πνευμονολογικό και ζήτησα να πάω πίσω πάλε για τούτο τον λόγο, εε , εν ένας θάλαμος ο οποίος, η εντατική είναι ένας θάλαμος ο οποίος έχει ιδιαιτερότητες πολλές, όμως βλέπεις πολύ αποτέλεσμα πάνω στην δουλεία σου

**R: Μάλιστα**

L: Εεε η φύση της δουλείας της εντατικής είναι και νομίζω όπως σε κάθε εντατική ο κάθε νοσηλευτής ένα πιάσει ένα δυο περιστατικά και θα δουλέψει πάνω τους εξολοκλήρου, που το μπάνιο τους την υγιεινή τους με τα φάρμακα μέχρι και την διασωλήνωση και αποσολήνωση του, εν τούτο που μου αρέσκει παραπάνω εμένα, ότι δεν μπλέκετε άλλος μέσα στην δουλειά την δική μου δλδ θα κάνω εγω το ταδε ένα πράμα, ένα μικρό κομμάτι στον άρρωστο μου και να έρτει ο άλλος να κάμει κάτι άλλο και ο άλλος κάτι άλλο, τζίνους τους άρρωστους ξέρουμε τους που το πρωί ως το μεσημέρι παραδώνουμε τους εμείς

**R: Δουλεύετε εξατομικευμένα δηλαδή δεν είναι όπως τους άλλους τους θαλάμους οκ, εκείνο που ήθελα να σε ρωτήσω συγκεκριμένα είναι σε ποια περίπτωση στην μονάδα την δική σας θα μπορώ να βρω ένα διασωληνομένο ξύπνιο ασθενή, δηλαδή να είναι off sedation που λέμε ασπουμε, σε ποιε, πότε θα το δω τούτο το πράμα**;

L: Πρόσφατα είχα ένα άρρωστο ο οποίος ήρθε με αναπνευστική λοίμωξη, ήταν χαπίτης (***ΧΑΠ, νόσος πνευμόνων***), ο οποίος έκαμνε, έμπαινε και σε Bipap σπίτι τις νύχτες και ο συγκεκριμένος εε ηταν διασωληνομένος και τελείως ξύπνιος, επικοινωνούσε πλήρως, διότι ο ίδιος ήξερε ότι δεν μπορούσε να αναπνεύσει μόνος του

**R: Οκ, είχες τούτο τον ασθενή, δηλαδή έβλεπες τον εσύ**

L: Nαι είχα τον

**R: ΟΚ, εκτός που τούτο τον ασθενή σε ποιες άλλες περιπτώσεις ένα δω, αν μπορείς να θυμηθείς να σκεφτείς τωρά, δηλαδή πότε κλείνετε την καταστολή και εν ξύπνιος ο ασθενής με τον σωλήνα**;

L: Εν λίγες οι περιπτώσεις, δηλαδή ως συνήθως όταν κλείσουμε την καταστολή και ξυπνήσει ο άρρωστος το επόμενο βήμα είναι η αποσωληνωση του

**R:Μάλιστα, δηλαδή ασπουμε στην λεγόμενη φάση του weaning αν δεν κάνω λάθος, που μου είπαν και οι συνάδελφοι σου;**

L:Nαι

**R: Οκ, Πως καταφέρνεις να επικοινωνήσεις με τούτους τους ασθενείς, με ποιους τρόπους;**

L:Ρωτάς τον κάποια πράματα και περιμένεις εσύ με ένα γνέψιμο του κεφαλιού του ή κλείσιμο του ματιού να καταλάβεις τι θέλει

**R: Μάλιστα**

L:Παραπάνω εν ερωτήσεις με απαντήσεις που γίνεται, δεν μπορείς να κάμεις συζήτηση

**R:Υπάρχει κάποιος άλλος τρόπος που σε βοηθά να επικοινωνήσεις, θέλεις ασπουμε να σκεφτείς ένα περιστατικό και να μου το περιγράψεις ή να σκεφτείς ότι εγώ είμαι διασωληνομένος , τι ένα έκαμνες , ένα ερτεις να μου πεις τι ασπούμε; Εε εννοώ απλά για να σε βοηθήσω να σκεφτείς και άλλους τρόπους**

L: Άρρωστοι οι οποίοι είναι διασωληνωμένοι και μπορούν να συνεννοηθούν, μπορεί να έχουν κάποιο πρόβλημα, εε πως να σου το πω, συγχύζει με λίο το (δείχνει το μαγνητόφωνο)

**R: Εν πειράζει, μεν αγχώνεσαι, μπορεί να έχουν λίγο πρόβλημα να πουν εκείνα που θέλουν εννοείς, νευρολογικά ασπουμε;**

L:Ναι από την στιγμή που εν μπορείς να μιλήσεις και βλέπεις ότι κάτι τον ενοχλεί τον συγκεκριμένο μπορεί να του ψηλώσει αρτηριακή πίεση ,μπορεί να κάμει ψηλές σφίξεις, μπορεί να ανήσυχος ή οτιδήποτε, αναγκαστικά πρέπει να κοντέψεις και να τον ρωτήσεις τι είναι εκείνο που τον ενοχλεί και να το σταματήσεις

**R:Άρα κάμνεις όπως μου είπες ως τωρά για να καταλάβω και εγώ καλά, γιατί εγώ δεν δουλεύω σε μονάδες για να ξέρω εεεε κάμνεις του κλειστού τύπου ερωτήσεις , όπως πονείς διψάς κλπ**

L:Ναι ναι

**R:Μάλιστα, οκ, τι πιστεύεις ότι θέλουν να σου πουν οι ασθενείς όταν προσπαθούν να επικοινωνήσουν μαζί σου;**

L:Eεξαρτάτε που τον κάθε ένα , τι τον ενοχλεί, τι δεν τον ενοχλεί , είχα συγκεκριμένο άρρωστο ο οποίος ,ο συγκεκριμένος που είπαμε πριν λίγο, ο οποίος ήταν τόσο άνετος με τον σωλήνα που του μιλούσες και γέλα, το μόνο του πρόβλημα είναι ότι δεν μπορούσε να φάει και συνέχεια ήταν το πεινώ πεινώ

**R: Έδειχνε σου δηλαδή**

L:Ναι έδειχνε ότι πεινά

**R:Οκ**

L:Έχει αρρώστους οι οποίοι οι περισσότεροι ενοχλά τους ο σωλήνας και μόλις ξυπνήσουν το πρώτο πράμα που κάνουν είναι το χέρι στον σωλήνα στοχευμένα για να τον τραβήξουν

**R:Μάλιστα, άρα εν ένα εμπόδιο επικοινωνίας ο σωλήνας**

L:Μεγάλο, το μεγαλύτερο νομίζω, δεν έχει..

**R:Παρατήρησες οτιδήποτε άλλο εμπόδιο στο να επικοινωνήσεις με τους ασθενείς , δηλαδή τι εν εκείνο που σε εμποδίζει να καταλάβεις, οι να καταλάβεις, να επικοινωνήσεις μαζί τους, τι σε δυσκολεύει, αν σε βοηθά να σκεφτείς ένα περιστατικό, το οποίο ένα κάμεις αναπαράσταση στον νου σου και ένα το σκεφτείς**

L:Που άλλους ασθενείς που έχω, έχει μια κλίμακα γλασκώβης 15 και εν με τον σωλήνα και νευρολογικά δεν έχει οποιοδήποτε άλλο πρόβλημα το μεγαλύτερο του πρόβλημα είναι ότι δεν μπορεί να μιλήσει ,

**R Μάλιστα**

L:Έχουμε ασθενείς ότι δεν βγαίνουν τέλια από την καταστολή, μπορεί αν τις κλίσουμε αλλά παίρνουν και μια δυο μέρες να κάψουν τα φάρμακα που λέμε και εν πιο δύσκολο να μιλήσεις μαζί τους, έχει άλλους που εν συγχυτικοί και πάλε δεν μπορείς να επικοινωνήσεις μαζί τους, απλά προσπαθείς εσύ να προσφέρεις ένα ήρεμο καλό περιβάλλον στον άρρωστο για να δεις αν καταφέρεις που εκείνα που πιστεύεις εσύ ότι εξαλείφθηκε ο παράγοντας που τον κάνει ανήσυχο

**R:Ωραία, έχεις κάποια συγκεκριμένη εμπειρία να μου περιγράψεις η οποία φάνηκε σου εσένα που σου έκανε εντύπωσε με τούτο τον ασθενή να μου περιγράψεις την καθημερινότητα που είχες μαζί του;**

L:Tον συγκεκριμένο που..

**R:Ναι που μου είπες ότι ήταν ξύπνιος και διασωληνομένος**

L:Λεω σου ήταν ένας άρρωστος ο οποίος ήρθε με λοίμωξη αναπνευστικού συστήματος και ψηλά διοξείδια, ηταν χαπίτης, που ένα χωρίο ο άνθρωπος, την περασμένη βδομάδα, γι αυτό το θυμούμαι καλά, υπέρβαρος εε και συγγενείς φίλων νοσηλευτών ο οποίος ήρθε με λοίμωξη αναπνευστικού, ψήλωσε τόσο πολλά το διοξείδιο που αναγκαστικά έπρεπε να τον διασωληνόσουμε τον, εδιασωληνώθηκε την πρώτη φορά με καταστολές, ήπια καταστολή, διοτι ηταν πολλά ήρεμος, εξαρχής αποσωληνώθηκε μια φορά έκαμε δυο μέρες εξαναδιασωληνώθηκε επειδή δεν τα κατάφερε και την δεύτερη φορά που τον δισωληνόσαμε ηταν χωρίς καταστολές χωρίς τίποτε, ήταν πολλά ήρεμος, όσο αφορά την επικοινωνία του ,έδειχνε σου κάποια πράματα, ήθελε νερό ήταν να σου δείξει κάποια πράματα ήθελε νερό ηταν να σου δείξει με το χέρι του νερό ή το πεινώ έδειχνε την κοιλιά του, εε ήταν ένας άρρωστος ο οποίος έλαλες του μια κουβέντα και εγέλαν και ήταν με τον σωλήνα αλλά ο ιδιος αντιλαμβανετουν επειδή δεν μπορώ να αναπνεύσω μόνος μου τούτο το πράμα χρειάζουμε το και συνεργάζετουν πλήρως

**R:Οκ, σε άτομα, επιστρέφω πίσω και ξαναρωτώ σε, τα οποία είπες μου πριν εν μικρή, τουτο το άτομο ήταν συνεργάσιμο, σε άλλα άτομα τα οποία εν ξύπνια και διασωλομένα στην , φαντάζουμε είπες μου εν μικρό το διάστημα που μένουν ξύπνια και διασωληνομένα την ώρα που θα τους αποσωληνώσετε, εεε πως είναι γενικά να προσπαθάς να εποικοινωνάς μαζί τους και να είναι σε εκείνη την φάση;**

L:Δύσκολο διότι έχεις ένα άρρωστο ο οποίος μόλις ξυπνήσει που τις καταστολές σηκώνει σφίξεις , ψηλώνει πιέσεις εν ανήσυχος εν μένει μέσα στο κρεβάτι ήσυχος, εεε το μόνο που κάμνει εν πάντα να εξαλείψεις τον παράγοντα που τον επηρεάζει, δηλαδή το χέρι του εν πάνω στον σωλήνα να τον τραβήξει , εν δύσκολο πολλά

**R:Οκ, εε χρησιμοποιάς οποιουσδήποτε άλλους τρόπους για να επικοινωνήσεις με τούτον ασθενή, ασπούμε έχεις κάποια στρατηγική που σε βοηθά να επικοινωνήσεις, για παράδειγμα, κάμνεις του κλειστού τύπου ερωτήσεις για να δεις τι τον ενοχλά, ήβρες κάποιο άλλο τρόπο που σε βοηθά να επικοινωνήσεις μαζί τους ασπούμε;**

L:Είχαμε άρρωστο ο οποίος δώσαμε του μαρκαδόρο και χαρτί και έγραφε μας πάνω τι ήθελε ή ένα πιο μικρό εε ο οποίος εε δώσαμε του πινακακάκι με μαρκαδόρο και έγραφε κάποιες λέξεις για να καταλάβουμε εμείς τι ήθελε ακριβώς

**R:ΟΚ μιας και αναφέρθηκες σε τούτα τα πράματα, έχετε οτιδήποτε άλλο στην μονάδα σας δηλαδή μέσα επικοινωνίας εχτός που μαρκαδόρο και χαρτί ασπούμε που εν το πιο απλό, ταμπλετ, πίνακες με εικόνες ή κάτι άλλο το οποίο σας βοηθά στην επικοινωνία**

L:Οι τούτα έχουμε τα για ψυχαγωγία του αρρώστου, για επικοινωνία εν παραπάνω εμείς, εν πολλά λίγοι και οι αρρώστοι οι οποίοι μπορούν να χρησιμοποιήσουν τα τάμπλετ διότι εν μιας ηλικίας και πάνω που νομίζω εν πιο δύσκολη για τζίνους

**R:Οκ, σωστό και τούτο, εε ερώτησα σε ξανά και πριν, είπες μου εν που το κάθε ασθενή αναλόγως ,τι πιστεύεις ότι προσπαθούν να σου πουν εκείνη την ώρα; Μήπως σκέφτηκες κάτι άλλο τωρά, δηλαδή ξυπνούν , κλείνεις τους την καταστολή και προσπαθούν να μιλήσουν, τι νομίζεις προσπαθούν να σου πουν τι είναι οι ανάγκες τους, ποιες είναι οι φόβοι τους;**

L:Πολλές, οι παραπάνω έχουν φοβίες, πρώτα από όλα οι παραπάνω δεν ξέρουν για ποιο λόγο είναι εκεί που είναι , εεε το πρώτο πράμα που κάνουν μόλις κοπούν οι καταστολές πριν αποσωληνωθούν πριν καλά καλά ξεκινήσουν να αντιλαμβάνονται κάποια πράματα, να τους ενημερώσουμε, γεια σου κύριε τάδε είσαι στην εντατική του νοσοκομείου Λεμεσού, διότι φέρνουν μας και που λάρνακα φέρνουν μας και Λευκωσία περιστατικά και πρέπει να το πούμε, εεε είμαι ο τάδε νοσηλευτής, ότι χρειαστείς είμαι κοντά σου, σε παρακαλώ μείνε ήρεμος, συνεργάστου λίγο και θα βγει ο σωλήνας εντός της ώρας ή εντός της ημέρας ή οτιδήποτε άλλο, θέλουν ενημέρωση γιατί ξαφνικά που ένα βαθύ ύπνο ανοίγεις τα μάτια σου και βλέπεις μπροστά σου δέκα νοσηλευτές και άλλους τόσους γιατρούς να πηγαίνουν και να έρχονται, μόνιτορ , καμπανούθκια να παίζουν, αναπνευστήρες να παίζουν, εν δύσκολο

**R:Αφου ανάφερες τούτο το περιβάλλον της εντατικής, πιστεύεις εσύ ότι επηρεάζει σε στο να καταφέρεις να επικοινωνήσεις με τούτους τους ασθενείς;**

L:Πολλά

**R:Δηλαδή με ποιο τρόπο;**

L:Πολλά, εν ένα περιβάλλον το οποίο έχει θόρυβο συνέχεια αν και προσπαθούμε να τον εξαλείψουμε με κάποιους τρόπους όμως δεν παυει να έχει θόρυβο, εε αποπροσανατολίζει τον άρρωστο και μόλις ξυπνήσει κάμνει τον και πιο ανήσυχο

**R:Οκ**

L:Φαντάστου εσένα ασπούμε μόλις ξυπνήσεις που τον ύπνο σου να ακούεις φωνές, δηλαδή κάπου, εν πιο δύσκολο να εστιάσεις στο ξύπνημα σου

**R: Τι άλλα εμπόδια εντοπίζεις στο να επικοινωνήσεις με τούτος τους ασθενείς; Τούτο ασπούμε το περιβάλλον είπες μου ότι εν ένα εμπόδιο τωρά, εε τι πιστεύεις ότι επηρεάζει τον ασθενή ή εσένα στο να..**

L:Η γλώσσα, αν ο ασθενής είναι ξένος, εν μπορώ να σκεφτώ κάτι άλλο τωρά

**R:Ντάξει οκ, εεμμ όταν δεν καταφ, ή μάλλον άμαν σου λέει κάποιος ασθενής κάτι και εσύ δεν καταλαβαίνεις τι θέλεις να σου πει, τι ένα προσπαθήσεις να κάμεις εκείνη την ώρα για να σε βοηθήσει να καταλάβεις τι θέλει ή ..**

L:Το πιο εύκολο που μπορείς να κάμεις είναι τούτες οι ερωτήσεις με τις απαντήσεις, γνέψιμο, δηλαδή ένα ξεκινήσω να τον ρωτώ κάποια πράματα που πιστεύω εγώ ότι μπορεί να τον επηρεάζουν και να περιμένω μια απάντηση , μια κατάφαση μια άρνηση με το κεφάλι του.Το πιο εύκολο που μπορεί να κάμεις.

**R:Εντάξει, όταν φτάσεις εκείνο το σημείο, πες ότι προσπαθείς να κάμεις τούτες τις ερωτήσεις και πάλε δεν αντιλαμβάνεσαι τι θέλει ο ασθενής , ο ασθενής τι πιστεύει εκείνη την ώρα, όταν θέλει να πει κάτι και δεν τα καταφέρνει**

L:Το τι νιώθει ακριβώς δεν μπορώ να ξέρω πιστεύω όμως ότι νιώθει πολλά άσχημα, σκέφτου να σε ενοχλά κάτι να προσπαθείς να πεις του αλλού και δεν μπορείς να κάμεις κάτι για τον εαυτό σου εσύ ότι ενοχλά με τούτο το πράμα και να μην σε καταλάβει, εν δύσκολο

**R:Για εσένα ασπουμε όταν προσπαθας να επικοινωνήσεις, δεν ξέρω πόσο συχνά συμβαίνει**

L: Συμβαίνει αρκετά συχνά διότι αποσωληνόμουμε αρρώστους , δηλαδή πριν τους αποσωληνωσεις πρέπει να κόψεις καταστολή

**R:Ωραία**

L:Και οι αρρώστοι οι οποίοι εν συνεργάσιμοι πλήρως εν δύσκολοι έως δεν υπάρχει , ο συγκεκριμένος που σου είπα πριν εδιασωληνώθηκε , κοπήκαν οι καταστολές του γλίορα , ξύπνησε και γι΄αυτό εσυνεργάζετουν

**R:Οκκ, εε όταν προσπαθείς να επικοινωνήσεις και δεν καταφέρνεις να καταλάβεις τι θέλουν , έστω και μετά από πολλές προσπάθειες εσένα τι σε κάνει να νιώθεις τούτο το πράμα ή τι είναι οι ενέργειες σου που θα κάνεις μετά**

L:Εξαρτάτε από τον βαθμό ανησυχίας του αρρώστου, δηλαδή αν είναι πάρα πολλά ανήσυχος ξεκινάς να γίνεσαι και εσύ ανήσυχος, πανικοβάλλεσαι λίγο, σκέφτου να έχεις ένα άρρωστο ο οποίος δεν μένει μέσα στο κρεβάτι πως να το πω και κυπριακά, ένα πέσει που το κρεβάτι κάτω γιατί έχει κάποιο πρόβλημα, ξεκινάς αν συνεχίσεις με τις ερωτήσεις και δεν πάρεις μια ξεκάθαρη απάντηση να δεις τι τον ανησυχεί πάεις στο επόμενο βήμα που για εμένα είναι να κοιτάξεις το περιβάλλον του , ασπούμε το κρεβάτι του

**R:Πολλά ωραία τούτα που μου λαλείς**

L:Που ένα πέσει μέσα μπορεί να τον ενοχλά κάτι, μπορεί να εν λερωμένος, να θέλει καθάρισμα ,μπορεί να έκλεισε ο καθετήρας να μεν φκάλει ούρα, να φουσκώνει η κύστη , νια νιώθει δυσφορία, τούτα ούλλα, εε αφού τα δεις ούλλα και δεις ότι δεν υπάρχει πρόβλημα και δεν υπάρχει τίποτε άλλο, εε το επόμενο βήμα νομίζω εν το παυσίπονο

**R:Ωραία, οκ, τι άλλο ήθελα να σε ρωτήσω, είπες μου πριν για τους πίνακες που έχετε και τους μαρκαδόρους, στην σχολή ή τέλος πάντων στην εξομοίωση που πήγες, εκπαιδεύτηκες ποτέ σε θέματα επικοινωνίας;**

L:Όχι,Αν θυμούμαι καλά όχι, ή αν έγινε εκπαίδευση έγινε μια αναφορά τίποτε άλλο

**R:Μια αναφορά οκ**

L:Εε εν το θυμούμαι να το εδιδακτικα και στην εντατική το είδα τούτο το πράμα, είχα δουλέψει και έξι χρόνια χειρουργικό πριν την εντατική , εν το ξαναχρησιμοποιήσαμε, στην εντατική το εχρη…

**R:Εννοείς τον μαρκαδόρο με τους πίνακες, οκ, εντάξει στην εντατική γιατί χρειάστηκε**

L:Και το χαρτί με την πέννα και τον μαρκαδόρο

**R:Σε περίπτωση που θα σου δίνετουν να εκπαιδευτείς σε θέματα επικοινωνίας, θα ήθελες να εκπαιδευτείς ή όχι;**

L:Φυσικά θα ήθελα

**R:Εσένα ως loizos τι σε βοηθά καλύτερα να στο να σε βοηθήσει διδαχτείς να είσαι δίπλα που τον ασθενή, να κάμνετε ένα μάθημα δίπλα που τον ασθενή, να κάμνετε ένα μάθημα μέσα στην τάξη , να το κάνετε διαδικτυακά, να το δεις μέσω βίντεο, τι νομίζεις θα σε βοήθαν καλύτερα στο να εκπαιδευτείς εσύ ως άτομο ασπουμε**

L:Νομίζω άμα έχεις με επαφή με το αντικείμενο εν πιο εύκολο για εσένα, δηλαδή εγώ θα επροτίμουν να μου το, κάποιος ο οποίος εν πιο εκπαιδευμένος που εμένα να μου το διδάξει και μετά να το εφαρμόσουμε πρακτικά, δηλαδή δίπλα στον άρρωστο

**R:Μάλιστα, άλλο που ήθελα να σε ρωτήσω, εε είπες μου πριν για τούτο τον ασθενή τον χαπίτη (ΧΑΠ) πόσες μέρες τον είχες τούτο τον ασθενή περίπου**

L:Κοντά μας ήταν δυο βδομάδες νομίζω

**R:Δυο βδομάδες, μπορείς να μου κάμεις μιαν αναπαράσταση για παράδειγμα ,πες ότι εγώ ήμουν ο ασθενής ήταν να έρθεις να μου μιλήσεις ασπουμε τι ήταν να μου πεις, προσπαθώ να σε βάλω έτσι λιο στο περιβάλλον που είσαι για θυμηθείς έτσι λια παραπάνω πράματα να μου πεις , αν θέλεις, πως ήταν να προσπαθήσεις να μου μιλήσεις ή τι ένα σου έλεγε τζίνος ασπουμε..**

L:Εννοείς όταν ήταν διασωληνομένος;

**R:Nαι ,εμένα ενδιαφέρει το κομμάτι, όταν επικοιν..**

L:Όταν επικοινωνεί αλλά εν με τον σωλήνα

**R:Ναι και ξύπνιος**

L:Ναι, εε λέω σου για τον συγκεκριμένο ασθενή ήταν σαν και δεν είχε σωλήνα, ήταν τόσο πολλά εε συνεργάσιμος και επικοινωνούσε πλήρως έστω και χωρίς λόγια που εν πολλά εύκολο να σου πω κάποια πράματα, έβλεπες τον όταν περνούσες που μπροστά του, επειδή είναι ανοιχτή τούτη η εντατική δεν είναι κλειστή , ένεφκέ σου με το χέρι του πήγαινες εκεί έδειχνε σου πονώ..

**R:Ναι**

L:Ή Θέλω νερό, ήταν πολλά εύκολο

**R:Με τους, οι άλλοι ασθενείς, που κόβεται τις καταστολές για να τους αποσωληνώσετε εν με τον ίδιο τρόπο που επικοινωνούν**;

L:Οι καμία σχέση, έχει αρρώστους οι οποίοι, υπάρχει το σύνδρομο της εντατικής, το ντελίριο, το οποίο μετά τις καταστολές που κοπούν και αφού ξυπνήσουν λίγο οι άρρωστοι ξεκινούν και έχουν παραισθήσεις

**R:Ωραία..**

L:Και εν πολλά δύσκολη η επικοινωνία, δηλαδή μπορεί να σε βλέπει και να μην θέλει καν να σε βλέπει ή μπορεί να εν εχθρικός απέναντι σου

**R:Χμμ**

L:Εεε και τούτο με το ντελίριουμ εβίωσα το πάρα πολλά με ένα συνάδελφο φίλο ο οποίος έπαθε εγκεφαλίτιδα, ιογενή εγκεφαλίτιδα, πριν 3-4 χρόνια και είχαμε τον στην εντατική

**R:Ο συνάδελφος σου εννοείς ήταν στην εντατική**

L:Ναι , 3 χρόνια πιο μικρός μου και φίλος καλός κάναμε και παρέα εκτός, όταν του κόψαμε τις καταστολές και ήταν διασωληνομένος ήταν πάρα πολλά δύσκολη η επικοινωνία μαζί του

**R:Οκ**

L:Και αφού εφκήκε ο σωλήνας και έκαψα τα φάρμακα που λέμε και ήταν πολλά καλύτερα είπε μου ότι νόμιζε ότι ήταν μέσα στην παράγκα και ετρέχαν νερά πράσινα που το ταβάνι, δηλαδή έχουν τόσες πολλές παραισθήσεις και νομίζουν εν αλήθεια και δυσκολεύει πάρα πολλά το κομμάτι της επικοινωνίας.

**R:Ξαναεπιστρέφω πίσω στο, εσένα ασπουμε όταν δεν κατάφερνες να επικοινωνήσεις , είπες μου ότι προκαλεί σας ανησυχία ως νοσηλευτές**

L:Ναι εννοείται

**R:Λόγω του ότι ειδικά με τον ασθενή , φοβάστε για την υγεία του τέλος πάντων, τι άλλα συναισθήματα σου προκαλεί εκείνη την ώρα, τι νιώθεις εκείνη την ώρα ασπούμε; Αν νιώθεις κάτι άλλο εχτός που ανησυχία**

L:το παραπάνω ανησυχία, ντάξει λυπάσαι που δεν μπορείς να βοηθήσεις ένα άνθρωπο ο οποίος θέλει βοήθεια, καμιά φορά βάλω την θέση μου στον τόπο τους να δω τι θα με ενόχλαν εμένα για να προσπαθήσω τουλάχιστο να έβρω ένα τρόπο να τον βοηθήσω, που δεν πετυχαίνει, εν διαφορετικές οι ανάγκες οι δικές μου εν διαφορετικά εκείνα που με επηρεάζουν εμένα και διαφορετικά εκείνα που επηρεάζουν τον καθένα ξεχωριστά

**R:Μάλιστα**

L:Εε άλλα το κυριότερο συναίσθημα εν η ανησυχία, εμένα τουλάχιστο τούτο με ενοχλά, ανάλογα με τον βαθμό ανησυχίας του αρρώστου και ο βαθμός ανησυχίας ο δικός μου

**R:Ωραία, αντιληπτό νομίζω**

L:Ντάξει έχει άλλους που εν λίγο πιο ήρεμοι , πιο κουλ πάνω στο συγκεκριμένο θέμα

**R: Εν ανάλογα με τον χαρακτήρα, εε ήθελα να σε ρωτήσω κάτι άλλο επίσης, τώρα εν καιρό covid, έχετε ασθενείς με covid κοντά σας**;

L:Οι, έχουμε αρρώστους οι οποίοι έρχονται με υποψία covid, μπαίνουν σε ένα δωμάτιο μόνοι τους και αφού βγει το covid και εν αρνητικό μεταφέρουν τους στον θάλαμο έξω

**R:Οκ, τούτο που μάλλον που ήθελα να σε ρωτήσω, φαντάζουμε ότι με το covid ντύνεστε έστω και με την υποψία, για να μπείτε μέσα, βάζετε τις μάσκες τα shields**

L:Ναι με το πρώτο κύμα, είχαμε εντατική covid , εντεινόμασταν συνέχεια

**R:Τούτο σε σχέση με τον τρόπο που δουλεύεις τωρά πως επηρεάζει την επικοινωνία το να ντύνεσαι που πάνω ως κάτω, κάμνεις κάτι διαφορετικό, ήβρες..**

L:Κάμνω

**R:Δηλαδή ;**

L:Εε πρώτα από όλα φορείς την μάσκα και την προσωπίδα

**R:Μάλιστα**

L:Εν πολλά πιο δύσκολο να επικοινωνήσεις με τον άρρωστο ειδικά οι προσωπίδες οι οποίες θολώνουν και εν βλέπεις τίποτε, εν πολλά πιο δύσκολο με τον άρρωστο και σκέφτου ένα άρρωστο ο οποίος ξυπνά ένα βαθύ ύπνο και έχει ένα σωλήνα μέσα στο στόμα και βλέπει σε εσένα που πάνω ως κάτω, ανησυχεί ακόμα παραπάνω

**R;Χμμ οκ, άρα έχεις να κάμεις με πιο ανήσυχα …**

L:ότι έχει να κάμει και σχέση με το περιβάλλον, διότι αν ανοίξει τα μάτια του και δει ένα πιο φιλικό περιβάλλον νομίζω εν πολλά πιο καλύτερη η επικοινωνία παρά αμάν ξυπνήσει και δει ένα άνθρωπο ντυμένο που πάνω ως κάτω

**R:Δηλαδή πως επηρεάζει διαφορετικά σε σχέση αμαν δεν ήσουν ντυμένος, δηλαδή ένα κάμεις κάτι διαφορετικό να σε βοηθήσει να επικοινωνήσεις μαζί τους; Aν σκεφτείς τώρα ένα ασθενή και είσαι ντυμένος και εν υποψία covid ή με ένα ασθενή που δεν είναι υποψία covid κάμνεις κάτι πιο έντονα κάμνεις κάτι διαφορετικό**

L:Έχουν πολύ διαφορά, δηλαδή με ένα άρρωστο ο οποίος ξέρεις ότι δεν έχει κάτι το, το covid που το πιάσαμε σαν παράδειγμα εν πολλά πιο εύκολη η επικοινωνία, δηλαδή μπορεί να κοντέψεις του αρρώστου, να του μιλήσεις λίγο πιο κοντά , να τον αγγίξεις , έστω και με το γάντι να τον ηρεμίσεις λίγο , οι άρρωστοι με covid εν πολλά διαφορετική η αντιμετώπιση τους

**R:Δηλαδή τι ακριβώς, ένα μείνεις πίσω εν θα τους κοντέψεις;**

L:Ένα τους κοντέψεις λίγο , εν θα κοντέψεις τέλια όπως κοντέψεις κάποιου άλλου αρρώστου, διότι πρέπει να έχεις λίγο και έννοια τον εαυτό τον δικό σου εε επειδή ώς συνήθως οι άρρωστοι με covid ήταν λοίμωξη αναπνευστικού και η λοίμωξη αναπνευστικού εε εν δύσκολη, έμεναν πολλές μέρες διασωληνωμένοι, πολλές μέρες διασωληνωμένοι έπαιρναν και πολλές καταστολές , για το covid που λέμε είχαν ένα θέμα οι άρρωστοι , έκαμνε σπασμό το διάφραγμα τους και δεν τους εβοήθαν στην αναπνοή στον αναπνευστήρα και εκτός που τα κατασταλτικά τα φάρμακα έδιναν και μυοχαλαρωτικά και χειροτέρευε την κατάσταση και το να ξυπνήσει ήταν πολλά πιο δύσκολο και πολλά πιο χρονοβόρα και ταυτόχρονα το ίδιο.

**R:Άρα στην φάση που τους είχατε χωρίς καταστολές και ξύπνιους τούτους τους ασθενείς ήταν χειρότερα που τους άλλους αντιλαμβάνομαι λόγω των φαρμάκων που έπαιρναν και της νόσου**

L:Ναι ήταν χειρότερα, ήταν πιο δύσκολο το να ξυπνήσουν τέλια και να αντιληφθούν κάποια πράματα

**R:Με τούτους τους ασθενείς χρησιμοποιήσετε τούτα τα εργαλεία όπως πέννα και χαρτί μαρκαδόρους κλπ ή εν βοηθούσαν**

L:Εν εβοηθούσε πολλά, διότι ως συνήθως έβγαινε ο σωλήνα πολλά πιο γρήγορα πριν ξυπνήσουν, δηλαδή , μόλις ξεκινήσαν και εκάυαν τα φάρμακα και ξέραμε ότι είχαν αναπνοή δική τους ο σωλήνας έβγαινε, διότι ήταν ήδη δέκα μέρες διασωληνωμένοι, αν το κρατήσεις λίγο παραπάνω , εν κάποιες ισορροπίες, εν το κρατήσεις παραπάνω, πρέπει να γίνει τραχειοστομία, αν βλέπεις ότι βγαίνει ο άρρωστος που τον αναπνευστήρα έστω και αν δεν ξυπνήσει τέλια δεν θα του κάμεις τραχειοστομία

**R:Νταξει οκ, ξαναπάω ξανά σε εκείνο το κομμάτι που εν ξύπνιος και διασωληνομένος, είτε με τους ασθενείς που είναι covid είτε με τους ασθενείς που δεν έχουν covid , είτε με τους ασθενείς που χρειάζεται να ντυθείς είτε με τους ασθενείς που δεν χρειάζεται να ντυθείς, εεμμ είπες μου ότι, τρόποι επικοινωνίας αν θυμούμαι και εγώ καλά, ρωτάς τους, δείχνουν σου, αγγίζεις τους , υπάρχει κάτι άλλο που μπορείς να σκεφτείς που σε βοηθά επικοινωνήσεις με τούτους τους ασθενείς εχτός που τούτα που είπαμε, που σου έρχεται στο μυαλό ή να μου περιγράψεις για κάποιο άλλο περιστατικό , εχτός που το ΧΑΠ που σου έκανε εντύπωση ασπούμε, αν σου έκανε εντύπωση**

L:Εν μπορώ να σκεφτώ κάτι άλλο τώρα, κάποιο άλλο περιστατικό, τρόποι επικοινωνίας εν τούτοι που σου είπα παραπάνω, εν λίγο δύσκολη η επικοινωνία του τελείως ξύπνιου και διασωληνομένου, διότι εν έχουμε πολλά περιστατικά έτσι

**R:Διότι εν και μικρός ο χρόνος που βλέπεις τούτους τους ασθενείς ασπουμε, τα εμπόδια ασπουμε είπες μου εμπόδια ότι μπορεί να εν το περιβάλλον της εντατικής**

L:Τα εμπόδια πολλά , το περιβάλλον , ο ίδιο π άρρωστος αν αντιλαμβάνεται η όχι , δηλαδή αν μιλά την γλώσσα την δική σου, αν δεν μιλά την γλώσσα την δική σου αν μιλάς εσύ την δική του όσο μπορείς ν του μιλήσεις, εεε ο σωλήνας που έχει στο στόμα, ο ρινογαστρικός που πολλές φορές ενοχλά τους παραπάνω που το σωλήνα, εξαρτάτε που τον άρρωστο, τούτα νομίζω..

**R:Οκ τούτα ήθελα να σε ρωτήσω…**

Συνέντευξη Νο 8

Bold: researcher

Normal letters: thalia

**thalia μου, όπως σου είχα πεί κάνω μια έρευνα που αφορά τους διασωληνομένους ξύπνιους ασθενείς, βασικά η έρευνα μου κατά ακρίβεια αφορά τους νοσηλευτές και και τις εμπειρίες επικοινωνίας με τούτους τους ασθενείς, από ότι κατάλαβες, ένας που τα δείγματα που έπιασα ήσουν εσύ γιατί δουλεύεις σε μονάδα εντατικής θεραπείας, θα σου κάμω μερικές ερωτήσεις αρχικά για εσένα ,για να έχω λίγα δημογραφικά στοιχεία, να ξέρεις οτιδήποτε αφορά τα στοιχεία που μου διας και τις απαντήσεις μένουν μεταξύ μας, οφείλω να σου το πω τούτο να το ξέρεις και πρόσβαση έχουν σε αυτά τα στοιχεία μόνο οι καθηγήτριες μου και εννοείται δεν θα γραφτεί κάπου το όνομα σου, αν μπούν κάποια κομμάτια ασπούμε , αποσπάσματα που μου λεν οι νοσηλευτές εννοείται δεν θα φανεί το όνομα σου. Νταξει ; απλά να το ξέρεις τούτο**

Μάλιστα

**Έτσι λίγες ερωτήσεις, καταρχάς πόσο χρονών είσαι;**

28

**Χμμ, πόσα χρόνια εργάζεσαι ως νοσηλεύτρια;**

6 χρόνια

**Ως νοσηλεύτρια σύνολο;**

6 χρόνια

**6 χρόνια οκ, στις μονάδες εντατικής θεραπείας πόσα χρόνια έχει που εργάζεσαι;**

Περίπου 4,5 – 5 χρόνια , περιπου

**Το πτυχίο σου επειδή σε ξέρω προσωπικά έκαμες το στο τεπακ από ότι γνωρίζω και κάνεις μεταπτυχιακό που;**

Eτέλειωσα το μεταπτυχιακό μου, διοίκηση μονάδων υγείας στο ανοιχτό πανεπιστήμιο Κύπρου

**Μπράβο σου, εμμ, μια έτσι αρχική ερώτηση, στις μονάδες σας σε ποιες περιπτώσεις θα βρώ ένα διασωληνομένο ξύπνιο ασθενή; Όχι τραχειοστομημένο απλά για να το , δηλαδή πότε σε ποιες περιπτώσεις θα έχεις ένα ασθενή που να έχει τον σωλήνα και να είναι εχτός καταστολών ασπούμε;**

Εχτός καταστολών; σπάνιο σχεδόν ανέφικτο τούτο που μου λες να είναι εκτός καταστολών, βασικά δεν υπάρχει για εμάς, εεε οι ασθενείς που έχουν ενδοτραχειακό σωλήνα επιβάλλεται να είναι υπό καταστολή και αναλγησία γιατί σκέφτου τώρα να είναι ένας ξύπνιος ασθενής και να έχει ένα ξένο σώμα στο στόμα του πόσο δύσκολο είναι για εκείνον, άρα επιβάλλεται να τον έχεις σε καταστολή σε αναλγησία και όταν τα κριτήρια του ασθενή δείχνουν ότι μπορεί να ξυπνήσει τότε διακόπτουμε καταστολή , βλέπουμε αν εκτελά οδηγίες και αν μπορεί να είναι προσανατολισμένος κλπ και χρειάζεται πολύ χαμηλές ανάγκες σε οξυγόνο και εκτελεί, τότε θα βγει ο σωλήνας, δηλαδή εκείνα τα λίγα λεπτά, που μπορεί να είναι εκτός καταστολής με τον ενδοτραχειακό, δηλαδή στην διαδικασία που θα βγει ο σωλήνας.

**Ok, δηλαδή αν κατάλαβα καλά, επειδή εγώ δεν εργάζομαι σε εντατική, στο λεγόμενο weaning θα βρούμε έτσι περιπτώσεις ασθενείς;**

Που την εμπειρία που έχω ναι

**Δηλαδή δεν εξανατυχε να δεις εσύ σε άλλη περίπτωση οκ, ας πιαστούμε που τζίνα τα λίγα λεπτά τότε γιατί εν τζίνους τους ασθενείς που αφορά τούτο το θέμα που κάμνω, εννοώ τζίνη την λίγη την ώρα έστω στην εντατική σας που τους έχετε χωρίς καταστολή μέχρι να ξυπνήσουν κλπ, γενικά πως είναι να επικοινωνείς με τούτου του είδους ασθενείς, πως είναι να δουλεύεις βασικά με τούτους τους ασθενείς; Έτσι μια γενική ερώτηση**

Eεε για εμένα προσωπικά το ίδιο είτε δουλεύω σε θάλαμο είτε με τούτους τους ασθενείς , δεν ξεχωρίζω τον ασθενή, προσπαθώ να έβρω επικοινωνίας, σε συγκεκριμένες περιπτώσεις μπορεί να τον ρωτήσω αν με ακούει να μου σφίξει το χέρι μου ή να κλίσει τα μάτια του, ή να μου βγάλει την γλώσσα του έξω ή να μου κατεβάσει το πιγούνι του κάτω ή να μου κουνήσει τα δάχτυλα των ποδιών του, εντάξει, επειδή σκέφτου έχεις τον σωλήνα μέσα στο στόμα εν μπορείς να μιλήσει λεκτικά άρα προσπαθώ να επικοινωνήσω κάπως πιο εικονικά μαζί του για να καταλάβω ότι ανταποκρίνεται και μπορεί να εκτελέσει

**Χμμ, μάλιστα, πιστεύεις ότι αντιλαμβάνεσαι τι ανάγκες έχουν εκείνη την ώρα οι ασθενείς; ότι μπορείς να καταλάβεις τις ανάγκες τους;**

Πιστεύω ότι η πρώτη ανάγκη είναι ότι νιώθουν δυσφορία αν νευρολογικά είναι σε κατάσταση που μπορεί να βγει ο σωλήνας, η πρώτη ανάγκη που έχουν είναι η δυσφορία που έχουν, έχοντας ξένο σώμα στο στόμα, η δεύτερη ανάγκη είναι η ανησυχία τους, καταρχάς ανοίγουν τα μάτια τους και βλέπουν ένα ξένο χώρο με ξένους ανθρώπους γύρω τους , με πολλούς ήχους και μηχανήματα που δεν μπορούν να καταλάβουν τι συμβαίνει, άρα η πρώτη μου ανάγκη είναι τους πιάσω το χέρι τους να τους εξηγήσω να τους πω ποια είμαι , να τους καθησυχάσω να τους πω ότι όλα θα πάνε καλά, ότι είμαστε δίπλα τους και να μην αγχώνονται και δεν θα τους αφήσουμε, όσο μπορούμε να τους βοηθήσουμε θα το κάμουμε , άρα προσπαθώ να επικοινωνήσω με τούτο τον τρόπο να τους εξηγήσω την κατάσταση που βρίσκονται και ποια είμαι , εεε και τι τους εσυνέβηκε, εεμμ , η Τρίτη ερώτηση είναι να τους ρωτήσω αν πονούν , να μου σφίξουν το χέρι , να μου κλείσουν τα μάτια για να μπορώ να τους καλύψω που τον πόνο να μεν πονούν να νιώθουν πιο άνετοι, άρα για εμένα για εμένα εν τρεις ανάγκες, εν τούτες, να τους εξηγήσω ότι δεν μπορώ να μιλήσουν γιατί έχουν τον σωλήνα στο στόμα και ότι αν είναι καλά νευρολογικά και είναι πιο ξύπνιοι θα το βγάλουμε και να μην ανησυχούν, ε και να τους εξηγήσω και τον λόγο που έχουν, δηλαδή τούτος ο σωλήνας βοηθά σε τωρά, βοηθά σε αναπνευστικά γιατι δεν μπορούσες να τους εξηγήσω ασπούμε την κατάσταση, τον πόνο και να τους συστήσω με το περιβάλλον και τον εαυτό μου

**Οκ είπες μου πολλά ωραία πράματα που θέλω να κρατηθώ, έπιασα πολλές πληροφορίες εγώ, εχτός που τούτους τους τρόπους που μου είπες τώρα, να τους εξηγήσεις να τους πεις να κουνήσουν τα χέρια πόδια τους, να βγάλουν γλώσσα και κλπ , μπορείς να σκεφτείς οποιουσδήποτε άλλους τρόπους που σου έρχονται στο μυαλό που χρησιμοποίησες εσύ μέχρι στιγμής για να επικοινωνήσεις με τούτους τους ασθενείς; δηλαδή τι άλλο κάνεις ασμπ, εάν ένας ασθενής δεν καταφέρνει να, μάλλον τούτο που σου είπα, χρησιμοποίησες οποιοσδήποτε άλλους τρόπους που κατάφερες να επικοινωνήσεις με τους ασθενείς τούτους;**

Προσπαθώ να σκεφτώ γιατί ο κύριος τρόπος επικοινωνίας, τουλάχιστο εκεί που βρίσκομαι τώρα τούτος είναι

**Χμμ**..

Εεεε, εννοείς εε, μπορεί να με διευκολύνεις ακόμα λίγο να καταλάβω ..που θέλεις να το πάρεις;

**Nαι είπες μου ..**

Για να σου πω αν συμβαίνει ή όχι τουλάχιστο.

**Νταξει, να σε ρωτήσω διαφορετικά, αν έχεις ένα ασθενή ασπούμε ο οποίος δεν μπορεί να συνεννοηθείς μαζί του κάνεις κάτι άλλο; Προβαίνεις σε κάτι άλλο ασπούμε για να καταλάβεις, ζητάς ασπούμε παράδειγμα, την άποψη κάποιου άλλου νοσηλευτή, έλα να δεις μπορεί να καταλάβεις κάτι εσύ που δεν κατάλαβα εγώ για παράδειγμα να του πεις, παράδειγμα τούτο, χρησιμοποιάς κάποιες άλλες στρατηγικές για να καταφέρεις να επικοινωνήσεις με τον ασθενή;**

Nτάξει στο ΓΝΛ πάντα υπάρχουν γιατροί, πάντα, εε, δεν είναι όπως κάποιους άλλους χώρους που δούλευα, εε και οι νοσηλευτές πάντα έρχονται να βοηθήσουν ο ενας τον άλλο, γι’αυτό δεν πήγε ο νους μου, γιατί είναι δεδομένο τούτο ότι εε κάποιος θα είναι εκεί κοντά μου και σίγουρα θα υπάρχει και γιατρός, δηλαδή στην διαδικασία που θα αφυπνίσουμε κάποιον, σίγουρα θα είναι γιατρός εκεί για να δει και εκείνος τι συμβαίνει και για να δει κατά πόσο εκτελέσει ο ασθενής κτλπ, αρα σίγουρα η συνεργασία και η ομαδικότητα εε εν πολλά σημαντικό στην επικοινωνία γιατί εγώ μπορεί να αντιληφθώ κάτι άλλο, είναι ένας τρόπος επικοινωνίας η αντίληψη η δική μου με την αντίληψη κάποιου άλλου, άρα όσο πιο πολλά μάτια υπάρχουν και τόσο πιο πολλά μυαλά , τόσο πιο κοντά θα είσαι στο αποτέλεσμα και στο πόρισμα που πρέπει να βγάλεις

**Οκ, σωστό, εμμ , άλλο που ήθελα να σε ρωτήσω, σε περίπτωση που δεν καταφέρεις να καταλάβεις τι θέλει ο ασθενής είτε γιατί διάφορους λόγους είτε γιατί εν συγχυσμένος είτε γιατί εν συνενοήσε δεν βλέπεις, τι πιστεύεις ότι νιώθουν τούτοι οι ασθενείς όταν θέλουν να πουν κάτι και δεν καταφέρνουν να πουν αυτό που θέλουν;**

Nαι εν κάτι που με στεναχωρεί πάρα πολλά εμένα προσωπικά, εεε να μην μπορώ να καταλάβω τι θέλουν ν μου πουν , σίγουρα θεωρώ ότι η πρώτη τους ανάγκη είναι παναγία μου που βρίσκομαι τωρά , εε σίγουρα αισθάνονται άγχος θεωρώ, ανησυχία , εεε εε σίγουρα τα συναισθήματα που θα νιώθουν δεν θα είναι όμορφα και είναι κάτι που με στεναχωράει να μην μπορώ να επικοινωνήσω μαζί τους γιατί δεν μπορείς να επικοινωνείς πάντα δυστυχώς, υπάρχουν περιπτώσεις που δεν μπορείς να επικοινωνήσεις πάντα γιατί ασπούμε μπορεί να μην ακούει καλά και το ακουστικό να είναι σπίτι και να μην προλάβαν να μας τα φέρουν, άρα εγώ όσο και να φωνάξω δεν με ακούει γιατί υπάρχουν διάφορα τέτοια περιστατικά, το μόνο επειδή εγω δεν μπορω να επικοινωνήσω μαζι τους και δεν μπορεί να καταλάβει, θα του πιάσω το χέρι και θα τον χαιδέψω και θα του δείξω ασπούμε με τούτο τον τρόπο ότι είμαι κοντά σου και δεν θα σου κάνω κακό, εε να του χαμογελάσω ασπούμε, ντάξει τώρα με τις μάσκες δεν μπορούμε αλλά πριν μπορούσες να χαμογελάσεις για να του δήξεις ένα θετικό μήνυμα, εμμ, τούτα κυρίως

**Οκ , εσύ είπες μου ότι σε στεναχωρεί πάρα πολλά τούτο το γεγονός ,γενικά τι , ασπούμε , πως νιώθεις εσύ προσωπικά, είπες μου για τον ασθενή τώρα τι πιστεύεις ότι νιώθει, εσύ σαν thalia πως νιώθεις ασπούμε όταν προσπαθείς να επικοινωνείς με τούτου του είδους ασθενείς και δεν τα καταφέρνεις στην τελική, κάνει σε να νιώθεις κάτι τούτο το πράμα; Είπες μου ότι νιώθεις στεναχώρια, πως νιώθεις εκείνη την ώρα**

Σίγουρα στενοχωρούμαι, γιατί εγώ προσωπικά προσπαθώ να βλέπω τον ασθενή σαν ένα δικό μου άτομο και βάλω στην θέση του ασθενή την μαμά μου ή τον παπά μου και νιώθω πάρα πολύ στεναχώρια αμαν δεν μπορώ να τους βοηθήσω, γιατί αμαν είναι να κάνεις τούτο το επάγγελμα πρέπει να διας ψυχή και τούτο προσπαθούμε να κάνουμε, σο αμαν νιώθεις ότι μπορείς να μην έκανες ή μπορεί να μην ξέρεις το 100% που μπορείς να προσφέρεις, στεναχωριέσαι, εν λογικό, νομίζω, για κάποια άτομα, για εμένα τουλάχιστο ισχυεί

**Θα σταθώ και σε κάτι άλλο , θα επιστρέψω και σε κάτι άλλο που μου είπες πριν , ότι ο ασθενής ξυπνά και βλέπει ένα ξένο περιβάλλον, ότι ακούει μηχανήματα , τα φώτα κλπ, εσύ τι εμπόδια βρίσκεις να επικοινωνήσεις με ένα διασωληνομένο ξύπνιο ασθενή , είπες ασπούμε τα αυτ, μπορεί να εν κάποια αυτονόητα και να μην σου έρχονται στον νου , το αυτονόητο ασπόυμε εν ο ίδιος ο σωλήνας που εν στο στόμα του ασπουμε και δεν τον βοηθά να μιλήσει, τι άλλο μπορείς να σκεφτείς που σε εμποδίζει στην επικοινωνία σας ασπούμε;**

O πόνος ,εεμ το άγχος του ,μπορεί να είναι σε Panic εκείνη την ώρα, γιατί ανοίγει τα μάτια του και βλέπει δέκα πλάσματα γύρω του που δεν γνωρίζει όλο φώτα μηχανήματα, μπορεί να έκαμε και μια επέμβαση και να πονάει και να σκέφτεται τώρα, παναγία μου τι συμβαίνει και δεν μπορώ να κινηθώ, άρα τούτους ούλους ο πανικός , το ξένο περιβάλλον, ο πόνος, η ανησυχία, το ότι ανοιγούν τα μάτια τους και δεν είναι εκέι η οικογένεια τους τα πιο κοντινα τους άτομα, εεε,η αναπνευστική δυσχέρια, παραπάνω αυτά, αυτά μπορώ να σκέφτω τωρά

**Οκ και κάτι άλλο που μου είπες , που μια και συμβαίνει τούτο τον καιρό με την πανδημία του covid , είπες μου ότι εν διαφοετικό τωρά με τους ασθενείς που ντύνεστε για να πάτε για τα covid, δηλαδή πως διαφοροποείται η επικοινωνία σας με τούτους τους ασθενείς , να τα πιάσουμε ένα ένα, για παράδειγμα ασπούμε, τι διαφέρει στην στολή σας, δηλαδή αν είχες ένα ασθενή ο οποίος δεν είχε covid και ένα ασθενή που είχε covid τι θα εφορούσες ασπούμε;**

Eεε, επειδή βγήκε νόμος και σε όλους τους κλειστούς χώρους επιβάλλεται να φορείς μάσκα ,χειρουργίκη μάσκα, αναγκάστικά η στολή μου είναι η ίδια απλα πρέπει να φορώ συνέχεια την χειρουργική μου μάσκα , εε επειδή επέρασα την πανδημία και την δευτερη φορά , τωρά τουτη την στιγμή περνώ την εε εν πολλά διαφορετικό και πραγματικά στεναχωρεί με πάρα πολλά για ψυχολογική κατάσταση τουτων των ατόμων ε και για εμας , γιατι θεωρώ ότι θα έπρεπε να στηριζόυμασταν από αυτή την κοινωνια , ενοω να μας βοηθούσαν γιατί εν πάρα πολλά δύσκολο και μόνο εκείνος που το βιώνει μπορεί να το καταλάβει, η διαφορά με εκείνους που έχουν covid , ντύνεσε που πάνω ως κάτω με σκουφάκια καπελάκια, το μονο που φαινεται είναι τα μάτια σου, βλέπουν σε σαν τον αστροναυτη, να μιλήσω λίγο λαικά για μπορώ να εξηγήσω τι θέλω, βλέπουν σε σαν τον ας, να βουράς να μεν προλαβαίνεις να κάμεις τα βασικά, γιατί είμαστε υποστελεχωμένοι και να βλεπουν εκείνοι με απορία και να διερωτούνται τι συμβαίνει τωρά και να αγχώνονται και να ανησυχούν γιατί νομίζουν είναι για εκείνους που βουράς

**Χμμμ**,

θεωρώ με κάποιο τρόπο τούτα τα άτομα, εχτος που εμάς γιατι εμεις είμαστε νοσηλευτες , δεν είμαστε ουτε ψυχολόγοι ούτε ψυχίατροι , ούτε τούτα ούλλα τα επαγγέλματα και όμως και όμως εν πολλά μεγάλο το έργο μας γιατι οφείλουμε να κάνουμε και τον ψυχολόγο κααι τον νοσηλευτη και τον φροντιστή και τον καθαριστή και τα πάντα, αρα θεωρώ ότι θα έπρεπε, επειδή εμείς εκάμαμε το μάθημα της ψυχολογίας και οσο εξελίσεται ο καθένας μας μπορεί να μάθει πεντε δέκα πράματα παραπάνω αλλα δεν είναι η ειδικότητα μας, θα έπρεπε να υπάρχουν σε όλες τις δομές ,σε όλων των ιδιωτικών και δημόσιων νοσηλευτηρίων ειδικοί ανθρώποι,ψυχολόγοι ψυχιάτροι για να δουν τούτα τα άτομα , γιατι τούτα τα άτομα τωρά υποφέρουν,γιατι εν τους κανεί ο πόνος τους , δεν κάνει που δεν μπορούν να αναπνευσουν, το αναπνευστικό τους εν χάλια , η ψυχολογία τους εν στα πατώματα, δηλαδή εν πάρα πολλά δύσκολο να είσαι σε ένα δωμάτιο με δέκα είκοσι άλλους γιατί είναι ενιαίος ο χώρος, δεν έχουν την ιδιωτικότητα τους , άλλο πρόβλημα, δεν έχουν τον προσωπικο τους χώρο, δεν είναι το δωμάτιο ασπούμε 209, είναι ένα δωμάτιο μεγάλο, που έχει όλους τους ασθενείς ο ένας δίπλα που τον άλλο , αρα σκέφτου εκείνος ο ανθρωπος πόσο άσχημα αισθάνεται , να υπάρχει επείγον δίπλα του , εκέινος να αγχώνεται αν είναι ξύπνιος, γιατι έχει που εν ξύπνιοι και να μην ξέρει τι συμβαίνει τωρά, να ακούει και τον πανικό των νοσηλευτών και των γιατρώ να προσπαθήσουν να σώσουν τι ζωή και εκείνος ο άνθρωπος να αγχώνεται και να τον πιάνει ο πανικός και να πρέπει να τον καθησυχάσω και εκείνο , εεε εν πολλά δύσκολη η κατάσταση

**Mμμ, κατάλαβα, οσο αφορά την επικοινωνια ,συγκεκριμένα για την επικοινωνία, είπες μου πριν με τους ασθενεις γενικά ένα τους πεις που είναι ένα τους πιάσεις το χέρι τους, ένα τους πεις κλείσε μου άνοιξε μου τα μάτια, τροποποιείτε κάτι στον ασθενή που έχει covid και πρέπει να είσαι ντυμένη που πάνω ως κάτω και φαίνονται μόνο τα μάτια σου με ένα ασθενή που δεν έχει covid, δηλαδή αλλάσει κάπως η επικοινωνία σου, χρησιμοποιάς κάτι άλλο; Για να επικοινωνήσεις με τούτους τους ασθενείς.**

Εχρησιμοποιήσαμε πίνακα με μαρκαδόρο, εκάμαμε το τούτο η αλήθεια

**Στους ασθενείς που έχουν covid ενοείς;**

Στην άλλη την εντατική γιατί τωρά έχουμε δυο εντατικές, επειδή, εντάξει δεν μπορείς να είσαι συνέχεια μέσα, για την δική σου προστασία ,εμπορούσε απέναντι μας να ηταν ενας ξύπνιος ασθενής ,εε και εμπορούσαμε να του γράψουμε κάτι ασπούμε για να τον καθησυχάσουμε, να του πούμε η ώρα είναι τάδε,είναι ωρα να προσπαθήσεις να κοιμηθείς, αν πονάς σήκωσε το χέρι σου ηταν δηλαδή ασθενείς που ηταν ξύπνιοι,δεν μιλουμε για ασθενεις που δεν ηταν ξύπνιοι ή ασθενείς ηταν ξύπνιοι απλά ηταν θετικοί ακόμα και δεν εμπορουσαν να φύγουν από την εντατική και εμπορούσαμε να επικοινωνήσουμε μαζί τους πολλά καλά, εχρησιμοποιούσαμε δηλαδή εκείνα τα μέσα

**Οκ για να καταλάβω γιατί εσυγχύστηκα λίγο εγώ ,δηλαδή τούτοι οι ασθενείς που ήταν θετικοί για να μπείς μεσα στο δωμάτιο ενοείς έδειχνες τους την ταμπέλα; ή είσουν κοντά τους και εδειχ..**

Ένα πίνακα με μαρκαδόρο, είχαμε γυαλί απέναντι αλλα έβλεπε μας και εβλέπαμεν τον, εε και εμπορούσε να του γράφαμε κάτι ασπούμε, όπως όλα θα πάνε καλά, να του βάλουμε χαρούμενη φατσούλα ή θα τα καταφέρουμε, κάτι έτσι πράματα

**Ενοεις να αποφευγετε να μπαίνετε και εσείς στο δωμάτιο τόσο συχνά ενοείς;**

E ντα μπορεί να μώλις εβγήκαμε και ο ασθενής να λίγο έτσι, ντάξει έχει διάφορων ειδών ασθενείς

**Οκ, κάτι άλλο που είχες με τούτους τους ασθενείς διαφορετικό με τούτους τους ασθενείς ή διαφορετικό τρόπο εποικοινωνίας σε σχέση με τους ασθενείς που δεν είχαν covid;**

Με τους ασθενεις που δεν είχαν covid

**Ναι εγω τωρά προσπαθώ να δω αν υπάρχει κάποια διαφορά στον τρόπο που επικοινωνείς με τούτους τους ασθενείς που είσαι ντυμένη που πάνω ως κάτω και έχουν covid ή με τους ασθενείς που ηταν πριν το covid και είσουν απλά τζιαμε, εννοω και είσουν κοντά τους και δεν..**

Να το θέσουμε λίγο διαφορετικά

**Ναι**

Δηλαδή με τους ασθενείς που αναγκαστικά φορώ μάσκα και τους ασθενεις που δεν εφορούσα μάσκα;

**Nαι βασικά την μάσκα και την στολή και τους ασθενείς που δεν ,εννοώ έσυ βλέπεις να σε εμποδίζει τούτο το πράμα, είπες μου ένα πράμα είναι η ταμπέλα ασπουμε, κάμνεις κάτι άλλο διαφορετικό που, επειδή σε εμποδίζει η στολή ασπουμε στο να επικοινωνήσεις;**

Nταξει εμπορούσαμε να,τουτοι οι ανθρώποι σίγουρα θέλουν να επικοινωνήσουν με την οικογένεια τους, ενας τρόπος που είβραμε ηταν να βάλουμε ένα κινητο σε σακουλάκι ασπούμε, γιατι ηταν μπει μέσα στο δωμάτιο και να πιάμε μέσω βάιπερ την οικογένεια του για να τους δει γιατι ηταν απαγορευμένες οι επισκέψεις

**Αχαα**

Αρα ενας τρόπος επικοινωνίας ηταν και η τεχνολογία, είχαμε ασύρματους που έξω μέσα και εμπορούσαν να μας ακούσουν την ώρα που πατούσαμε εμεις το κουμπι που μέσα και να επικοινωνήσουμε εμεις μαζί τους,γιατι είπαμε είναι ένα δωματίο που ηταν ολοι μέσα και στην παλιά την εντατική και στην καινουργια, το ιδιο πράμα είναι, εχουμε πάλε μονόκλινα αλλα που το 2012 εως το 2017 είναι ενοιέως ο χώρος

**Οκ κατάλαβα , τους ασθενείς που δεν είχαν covid, που πριν να πάεις στην εντατική των covid εχρησιμοποιούσετε τον πίνακα τουτο με τον μαρκαδόρο**

Ναι σπάνια όμως, έκαμαμε το όμως ναι

**Εεεε είδες να σας εβοήθησε ασπούμε η όχι**

Κάποιες φορές ναι , κάποιες φορές επειδή ο ασθενης είναι σε ντελίριουμ πραγματικο , δηλαδή ότι και να του γράψεις εκείνη την ώρα δεν μπορεί να συγκεντρωθεί, όχι, αλλα θεωρώ ότι κάποτε πρέπει να χρησιμοποιέται, εεε ότι μπορείς δηλαδή που θέμα επικοινωνίας να προσφέρεις, επιβάλλεται να το κάμει ενας σωστός νοσηλευτής

**Οκ εχτος που τον πίνακα με τον μαρκαδόρο, χρησιμοποιάτε οτιδήποτε άλλο ασπουμε, είπες μου για παράδειγμα το τηλέφωνο που σε βοηθά να επικοινωνήσετε ,εχετε οποιδήποτε τάμπλετ που να έχει κάποιο πρόγραμμα που να σε βοηθά με την επικοινωνία ή κάποιο πίνακα με εικόνες**

Εε όχι,όπως ειπα πριν η τεχνολογια και εγενικευσα το ασπουμε, μπορεί να είναι κινητό μπορεί να είναι ταμπλετ, εε που μπορεί να είναι μέσο επικοινωνίας, μπορεί να είναι τα ακουστικά μέσα ,μπορεί να εν ενας πίνακας, ένα κομμάτι χαρτί και να του γράψεις κάτι ή να σου γράψει κάτι γιατί και τούτο εκάμαμε το, να μας γράψει ο ασθενής κάτι, πάνω σε μια κόλλα, σε ένα χαρτόνι γιατι δεν μπροούσε να μας μιλήσει, εεε η αφή, η επικοινωνία που θέμα αφής ακόμα και η σιωπή, κάποτε ενας τρόπος επικοινωνίας είναι και η σιωπή, δηλαδή μπορεί να μεν πω κάτι απλα να κοιτάξω τον άλλο, παλε επικοινωνω,πάλε θα δώσω το μήνυμα μου, έχει περιπτώσεις που πρέπει να σιωπάς, που είναι και αυτος ένα τρόπος επικοινωνίας

**Οκ, ηθελα να σε ρωτησω, γενικα στην καρίερα σου που εκπαιδευτηκες εως νοσηλευτρια ή στο μεταπτυχιακό σου κλπ, εκπαιδευτηκες ποτέ σε θέματα επικοινωνίας με τους διασωληνομένους ασθενείς**

Στο τεπακ, νομιζω έτσι επειδή επεράσαν αρκετά χρόνια εε θυμουμε ότι κάμαμε μάθημα, ότι είχαμε μάθημα επικοινωνίας εν δεδομένο, εε θυμούμε όμως ότι, εν θυμουμε για διασωληνομένους ασθενείς, θυμουμε ότι κάμαμε μάθημα επικοινωνίας όταν έχει πένθος κάποιος, όταν πεθάνει κάποιος πως πρέπει να το χειριστούμε, εεε τα μέσα επικοινωνίας, τουτα που είχα πει, εν που το τεπακ που τα θυμούμε αλλα και που τα βιώματα μου,γιατι αναπτύσεις τα, κάποια αναπτύσεις τα παραπάνω , κάποια πιο λιγα, αλλα δεν πολλοθυμούμε, επεράσαν και τόσα χρόνια

**Οκ θεωρητικά αν σου εδίνετουν η ευκαιρία να εκπαιδευτέις σε αυτά τα θέματα επικοινωνίας ειδικά οσο αφορά την δουλεια σου με διασωληνομένους ασθενεις ή με δισωληνομένους ξύπνιους ασθενείς θα ηθελες να το κάμεις ή όχι;**

Εννοείς να μου παραχωρήσει η δουλεια μου κάποιο σεμινάριο

**Οτιδήποτε αν σου εδίνετουν η ευκαρία για παράδειγμα**

Εεεε θεωρώ ότι ναι, ότι μαθω εν κέρδος

**ΟΚ, ως thalia τι πιστευεις ότι θα σε εβοήθαν, να έκανες το μάθημα διαδυχτιακά , να το έκαμνες σε μια αίθουσα να το κάμνετε δίπλα που τον ασθενή, ασπούμε να σας εξηγούσαν κάποια πράματα δίπλα που τον ασθενή να το κάμνετε και στην αίθουσα και στον ασθενή, εννοω πως ένα σε πιστευεις ότι θα σε βοήθαν να κάμεις το μάθημα, να αφομιώσεις παραπάνω πράματα**

Νταξει, ο κάθε ανθρωπος διαφέρει και ξεχωρίζει, δεν μαθαίνουν ολοι με τον ίδιο τρόπο, απλα επειδή τωρά η κατασταση είναι τέτοια όπως είναι τωρά, σίγουρα δεν θα προτιμούσα να είμαι σε αιθουσα γιατι εν ξέρω , θελω να προστατέψω τον ευατο μου για να μπορώ να εργάζομαι, αρα το πιο σωστό αυτή την στιγμη είναι το διαδυχτιακό, επειδη εκαμα και το μάστερ μου διαδυχτυακα, ηταν εξ αποστάσεως έχω μια εξοικίωση αν και το θεωρώ πολύ πιο δύσκολο το διαδυχτιακο εγω προσωπικά, επειδη εκαμα και τα δυο είδη,δυαδιχτιακα και ενταξει,μπορει ασπουμε μετα αν υπάρχει κάποιο είδος πρακτικης να έχω επαφή με κάποιον ασθενή για να εξασκήσω εκείνα που θα μάθω ασπουμε,μπορεί ασπούμε να βαθμολογηθώ που κάποιον ανώτερο μου ή που κάποιον που να πρέπει να επιβαιβεώσει ότι υπήρχε τούτο το περιστατικό και ότι όντως εγίνικαν τούτα τα πράματα κλπ κλπ, δηλαδη να υπάρχει ενας τρόπος αξιολόγησης μου

**Μαλιστα, γενικα από την εμπειρια σου, έχει κάτι που σου εμεινε στον νου, κάποιο περιστατικό που σου έκανε εντύπωση,που θυμάσε για κάποιο λόγο που θέλεις να το μοιραστείς μαζί μας;**

Eχω πολλά που

**Ναι ενοείται αν έχει κάτι που σου εμεινε στον νου ή κατι που οσο αφορά το θέμα επικοινωνίας που σου εκανε εντυπωση με κάποιον,για κάποιο λόγο ασπούμε, που να σου έμεινε, αν έχει καποιο φυσικα και να σου έχει μπορεί να μην σου έρχεται τωρά κάποιο στον νου**

Μπορει να μην μου έρχεται γιατι εν πράματα που συμβαινουν καθημερινά,ετσι δεν μπορω να τα βαθμολογήσω , να πω τα πιο σικοριστικα

**Οκ**

Νταξει το αγχος παραπανω και ο πανικος που βιωνουν τούτα τα ατομα, ετσι λιο τις πρώτες φορές που μπορεί να με επηρέασε κάπως, αλλα με την επικοινωνία, καθησυχάζετε τουτο το πραμα, τουτος ο πανικος τούτο το άγχος, δηλαδή το αγγιγμα το να του μιλήσεις του αλλου , καθησυχάζει τον δηλαδή καμια σχέση,πολλά σημαντική η επικοινωνία στο επαγγελμα μας , παρα πολλα σημαντική και μπράβο σας που το θαιτετε ,πολλα σημαντικος ο ρόλος μας και πρέπει να βελτιωνόμαστε

**Συμφωνω….**

Ααα τα εμποδια εν πάρα πολλά, είπα εμπόδια;

**Είπες μου εμπόδια,ο χρόνος , το αγχος, πες μουυ**

Ο χρόνος, η υποστελέχωση εμμ το να πρεπει να περάσουν πάρα πολλές δουλειες που τα χέρια σου και δυστυχώς δεν μπορείς να ασχοληθείς με τον ασθενη, δυστυχώς , συμβαινει και τούτο,εεε δηλαδή τα υπερβολικά καθήκοντα που σου αναθέτουν δηλαδή εε κάμνουν σε πολλές φορές να μην μπορείς να ασχοληθεις με τον ασθενη και να μην του δώσεις εκεινο που του αξίζει εε αρα για εμενα το κυριο εμποδιο σε τούτη την κατάσταση είναι η επ. εξουθένωση γιατι, γιατι πρέπει να στηρίξεις και τον ίδιο σου τον ευατό, εάν εγω είμαι εξανλημένη, είμαι διαλλυμένη πως θα βοηθήσω τον απεναντι μου, όταν εγω η ιδια είμαι κουρασμένη,απλα προσπαθώ να πάω να κάμω την δουλεια μου ασπούμε, να κάμω τα βασικά και να φύγω, μπορει να ακούγονται ψυχρα και ωμα τούτα που λεω αλλα συμβαινουν και κάποιος πρεπει να τα πει

**Σωστο**

Λοιπον, η επ. εξουθένωση, η ψυχολογία του προσωπικού, γιατρων νοηλευτων καθαριστων, εν παρα πολλά σημαντικά, ο χρόνος η υποστελέχωση, οι μειωμένες εγκαταστάσεις, δηλαδη θεωρω ότι χωρις να το ψάξω αλλα είμαι σίγουρη , στις χώρες του εξωτερικού υπάρχουν πάρα πολλά μέσα επικοινωνίας αλλά εμείς στην κύπρο δεν έχουμε ιδέα και ειμαι σίγουρη για τούτο που λέω ,αρα πρέπει να ανοιξουμε λίγο τα μυαλα μας να απαιτείσουμε περισσότερο για την υγεία , να σκεφτούμε ότι ο κάθε ασθενης θα μπορούσε να ηταν δικός μας ανθρωπος και για να μπορέσουμε να κάμουμε επιτέλους εκείνα όλα που προσπαθούμε να διδάξουμε, εε και να μην είναι μόνο εικονικά αλλα και πραγματικά, τα βασικα εν τούτα και το άλλο που ήθελα να πω εν ότι πολλά μαθαίνουμε και στο πανεπηστίμιο και μεταπτυχιακά κτλπ, εε αλλα κάποτε τα πιο απλα εν τα πιο σημαντικά ,το να κάτσω να σου μιλήσω τωρά πάρα πολλά επιστημονικά εε μπορεί ουτε καν να χρησιμοποιούντε τούτα τα πράματα, εγω εμιλησα σου απλα αληθινά με τούτα που πραγματικά βιώνουμε και εν τα καθημερινά μας και η ημιμάθεια εν χειρότερη που την αμαθεια, αρα καλύτερα να επενδύσουμε στα απλα να γινουμε τέλιοι στα απλα και σιγα σιγα να προσθέτουμε νέους τρόπους επικοινωνιας, με τα απλα ας είμαστε τέλιοι και μετα προχωρούμε και στα υπολοιπα, για εμενα , γιατι κάποτε τα απλα δεν τηρουντε και δεν γίνονται από όλους, αρα θα ηταν καλύτερα να τελοιοποιήσουμε τα απλα και υστερα να προχωρησουμε παρακατω

**Όταν λες ότι τα απλα δεν τηρουντε και δεν γινονται από ολους ενοεις αφορα λογω των εμποδιων που μου ειπες εσυ, της υποστελεχωσης , του burn out;**

Όχι μόνο , είναι και η αντιληψη του κάθε νοσηλευτη γιατρου κλπ, δεν εχουν ολοι την ιδια αντιληψη , ουτε την ιδια ενσυναισθηση ουτε ένας βουλευτης εν ιδιος με τον άλλο, ενας αστυνομικος εν ιδιος με τον άλλο, όχι, όπως και σε εμας , ενας νοσηλευτης δεν είναι ο ιδιος με τον άλλο και εκεινοι που ξεχωρίζουν , ξεχωρίζουν στις λεπτομέριες

**Μαλιστα thalia μου…**

Νο 9

Bold: researcher

Unbold-normal letters: antria

**..Που λες όπως σου έχω πει κάνω μια έρευνα που αφορά τις εμπειρίες των νοσηλευτών σε μονάδες εντατικής θεραπείας με διασωληνομένους ξύπνιους ασθενείς**

Οκ

**Τούτη εν η έρευνα, να σου πω να συζητήσουμε μετά, αρχικά θέλω να μάθω το background σου αν γίνεται, πόσα χρόνια εργάζεσαι ως νοσηλεύτρια σύνολο;**

Εεεε 21

**21 χρόνια μπράβο, ούτε που σου φαίνεται, εεμμ, που έχεις σπουδάσει νοσηλευτική;**

Στην Κύπρο

**έχεις κάμει κάποιο μεταπτυχιακό;**

Όχι

**Έχεις κάνει οποιαδήποτε άλλη εκπαίδευση γενικά στην νοσηλευτική;**

Ναι έκαμα το μεταβασικό της εντατικής , μετά εξειδίκευση πάλε στην εντατική του ΤΕΠΑΚ, του υπουργείου τέλος πάντων ,εεε και εκπαίδευση στην αιμοκάθαρση, εε αυτά, μέσα στην εξειδίκευση της εντατικής έχει εντατική παιδιατρική , νεογνολογία κλπ, εχτός που τους ενήλικες

**Οκ το άλλο που ήθελα να σε ρωτήσω, πόσα χρόνια δουλεύεις στις μονάδες γενικά;**

Στις μονάδες γενικά δούλεψα που το 2008 έως τώρα

**12 χρόνια δηλαδή**

12 χρόνια αλλά και πριν ακόμα δυο χρόνια στην πρώτη εντατική της Λεμεσού

**Δηλαδή σύνολο 14;**

Ναι

**Σύνολο 14 χρόνια οκ, μάλιστα , το πρώτο πράμα που θέλω να σε ρωτήσω , στην μονάδα την δική σας σε ποιες περιπτώσεις να έβρω εγώ, ας πούμε ένα άτομο που δεν ξέρω (αναφέρομαι σε εμένα), ένα διασωληνωμένο ξύπνιο ασθενή; Πότε βλέπεις εσύ ένα διασωληνωμένο ξύπνιο ασθενή;**

Βασικά εεμ τα τελευταία χρόνια που έχει εντατικολόγους στις μονάδες στην Λεμεσό βρίσκεις όλο και πιο πολλά ξύπνιους διασωληνομένους γιατί δεν παίρνουν τόση καταστολή όσο έπαιρναν πριν, άρα στην φάση του απογαλακτισμού που ένα βγουν που τον αναπνευστήρα σίγουρα θα είναι ξύπνιοι και μπορείς να επικοινωνήσεις μαζί τους φουλ, απλά εν θα ακούεις την φωνή τους , όμως μπορείς να επικοινωνήσεις άνετα, ενώ πιο παλιά ας πούμε ήταν πιο πολλές μέρες σε καταστολή οι άρρωστοι και ήταν πάρα πολλά δύσκολο να επικοινωνήσεις σαν ήταν διασωληνωμένοι

**Δηλαδή ας πούμε η φάση που ένα βρούμε στην εντατική την δική σας εν μόνο στην φάση του απογαλακτισμού που τυχαίνει να έβρω ένα διασωληνωμένο ξύπνιο ασθενή, υπάρχουν οποιεσδήποτε άλλες συνθήκες που ένα έβρω ένα διασωληνωμένο ξύπνιο, off sedation ;**

όταν, ναι, στην φάση του απογαλακτισμού σίγουρα ,στην φάση που είναι ένας χρόνιος ασθενής ο όποιος για κάποιο λόγο δεν μπορεί να φκει, δεν παίρνει καταστολή και αν είναι ξύπνιος , προσπαθείς να είναι όσο πιο ήρεμος γίνεται αλλά μπορεί να είναι με τον σωλήνα και να επικοινωνεί , εε, αλλά εντάξει τούτο δεν συμβαίνει πολλά συχνά

**Οκ**

Μετά, αν ένας άρρωστος γίνεται χρόνιος και δεν απογαλακτίζετε συνήθως θα γίνει τραχειοστομία άρα δεν θα έχει σωλήνα , θα είναι σε αναπνευστήρα αλλά δεν θα είναι διασωληνωμένος

**ΟΚ, μάλιστα, γενικά πως είναι να δουλεύεις με τούτους τους ασθενείς , μιλώ σχετικά με τούτους που εν ξύπνιους, επειδή η μονάδα , εν διάφοροι ασθενείς, εν τραχειοστομημένοι, εν διασωληνωμένοι, εν κοιμισμένη , εν με CPAP, εγώ μιλώ για τούτη την ομάδα των ασθενών. Των ξύπνιων , πως είναι να δουλεύεις;**

Ναι , γενικά θέλουν πιο ήπιους χειρισμούς, θέλουν να τους εξηγείς τα πάντα γιατί ανησυχούν πάρα πολλά εύκολα επειδή δεν μπορούν να σου μιλήσουν , εε εν μπορούν ας πούμε, ξέρουν ότι έχουν το σωλήνα μέσα στο στόμα και πρέπει να εν ήρεμοι και πρέπει να τους λες συνέχεια, δηλαδή όσο πιο ήρεμος είσαι εσύ , μεταφέρεις τους το πράμα αν εσύ βιάζεσαι να τα κάμεις όλα, συνήθως τις μονάδες, υπάρχει, σαν και κάποιος μας βουρα που πίσω, χαχα, πολλές φορές εν χρειάζεται αλλα σαν και γίνεται τούτο το πράμα που μόνο του γιατί εν μονάδα , εε άρα θέλουν πιο πολλή επικοινωνία σίγουρα , διαφορετικά γίνονται ανήσυχοι συγχυτικοί και το πιο πιθανό να μεν μπορούν να φκουν και θα πρέπει να τους ξανα καταστείλεις

**Οκ να στραφώ λίγο πίσω στην προηγούμενη ερώτηση,, βασικά τούτη η διαδικασία που μου είπες στην φάση του weaning ή σε ένα χρόνια ασθενή, περίπου , δηλαδή, να πιάσω πρώτα την φάση του weaning πόση ώρα ώρες παίρνει τούτο το πράμα δηλαδή, πόση ώρα εν ένας ασθενής τόση ώρα ξύπνιος**

Μπορεί στην φάση του weaning να είναι μια ώρα που ένα φκει , εν λιες ώρες ας πούμε ένας άρρωστος που χειρουργείο διασωληνωμένος ένα τον ξυπνήσεις και ένα τον φκαλεις γλίορα , έχεις φάσεις που εν πιο πολλές, όσο πιο πολλές μέρες εν ο άρρωστος στον αναπνευστήρα το weaning Πάει πιο πολλά σε χρόνο, έχει φάσεις που μπορεί ο άρρωστος να ξυπνά κάθε μέρα λίγο να ξανακοιμάται και την επόμενη να ξαναξυπνά, εξαρτάται, εν εξατομικευμένο πράμα εν έχει ώρα ας πούμε, μπορεί να εν που μισή ώρα μέχρι 2 μέρες ας πούμε, λέω εγώ τώρα

**Μάλιστα κατάλαβα οκ, πως επικοινωνείς εσύ με τούτους τους ασθενείς, πως καταφέρνεις να επικοινωνήσεις με ποιους τρόπους**

Απλά επειδή τούτες τις μέρες έτυχε να έχω επικοινωνία με ένα άρρωστο που ήταν covid άρρωστος και έκαμε πάρα πολύ καιρό στην εντατική και έγινε ένας χρόνιος άρρωστος που ήταν ξύπνιος στον αναπνευστήρα και μετά ξύπνιος τραχειοτομημένος, μιλήσαμε στο viber τούτες τις μέρες και θυμάτουν με, βασικά εγώ προσωπικά εε συνήθως εεε μιλώ τους λέω τους ποια είμαι εε λέω τους κάποιες οδηγίες γιατί χρειάζονται να τους πεις τι να κάμουν ακριβώς γενικά όμως

**Όπως για παράδειγμα, συγνώμη Antria μου που σε διακόπτω, επειδή θέλω να μάθω πιο λεπτομερώς τούτα τα πράματα**

Όπως για παράδειγμα πως να αναπνέουν , όπως πχ να σου , για να καταλάβεις ότι εν ξύπνιος ότι επικοινωνεί και ότι συνεργάζεται να σου ανοιγοκλείνει τα μάτια να σου σφίξει το χέρι σου να κάμει κίνηση το πρόσωπο του τα άκρα του να σου πει αν πονάει με νοήματα, πολλές φορές χρησιμοποιούμε κλίμακες για τον πόνο αλλά σπάνια συνήθως άμαν εν ξύπνιο βρίσκουν τρόπο και με τις εκφράσεις τους να σου πουν πόσο πονούν ή όχι, βασικά εν παραπάνω κάποιες οδηγίες που θα τους δώσεις αλλά το πιο συχνό είναι πως να αναπνέουν για να μπορούν να φκουν πιο εύκολα, εε αυτά

**Οκ**

Να ξέρω εγώ να προσπαθούν να κρατούν το στόμα τους ανοιχτό να μεν δαγκώνουν τον σωλήνα, τούτα τα πράματα, δεν μπορώ να σκεφτώ κάτι άλλο τώρα

**εντάξει δεν έχει πρόβλημα, τι πιστεύεις ότι εν οι ανάγκες των ασθενών που θα ανοίξουν τα μάτια τους και που θα είναι off sedation και θα αντιλαμβάνονται και θα έχουν τον σωλήνα στο στόμα τους, τι πιστεύεις ότι προσπαθούν να σου πουν;**

Προσπαθούν να μιλήσουν και σου πουν ότι ενοχλά τους, ότι βασικά, που αρρώστους που ζήτησαν να μας γράψουν, ρωτούν που είναι, εε ρωτούν τι έπαθαν, εεε ζητούν τους δικούς τους, εεε ας πούμε ένα πχ τούτος ο άρρωστος που σου έλεγα πριν, ζητούσε τον σκύλο του και όντως μετά που συνήλθε Θυμάτουν ότι εζήτησε τον σκύλο του εε μια έχουν ανάγκη που να νιώσουν ασφάλεια, να εν κάποιος δίπλα τους να τους μιλάει και να τους ακουμπά, μόλις το κάμεις τούτο το πράμα ηρεμούν αμέσως

**Εννοείς να τους ακουμπήσεις και να τους μιλήσεις πχ**

Ναι εχτός αν όσο πιο πολλές μέρες είναι και συνήθως τα πιο νεαρά άτομα, ξυπνούν πιο συγχυτικά

**Πιο συγχυτικά οκ**

Βασικά ξυπνούν και εν λίγο πιο, θέλουν πιο πολλή ώρα να μπορέσουν να επικοινωνήσουν μαζί σου σαν και οι πιο μεγάλες ηλικίες εν πιο ήρεμα τα πράματα

**Μάλιστα οκ, εε ποια εν τα εμπόδια που πιστεύεις ότι βρίσκεις στο να επικοινωνήσεις με ένα τέτοιο ασθενή, γενικά που..**

Τα εμπόδια εν η ΜΕΘ σαν χώρος , δηλαδή η ΜΕθ πλέον επειδή εν ανοιχτές εν ένας άρρωστος δίπλα που τον άλλο, υπάρχει μια γενική φασαρία ανησυχία, φώτα κλπ που δεν μπορείς να ελέγξεις, πχ εσύ μπορείς να έχεις τον άρρωστο σου που θα απογαλακτίζετε και δίπλα να έχει πολυτραυματία πχ, που θα γίνεται χαμός, δεν μπορείς να περιορίσεις το στρες τον χώρο για να απογαλακτίσεις εσύ τον άρρωστο σου και να επικοινωνήσεις φουλ μαζί του ή μπορείς να έχεις 2 ασθενείς και να είσαι μια στον ένα μια στον άλλο άλλα να μεν μπορείς να διαθέσεις τον χρόνο που χρειάζεται ο συγκεκριμένος άρρωστος που θέλει επικοινωνία ή ξέρω εγώ το να πρέπει να βοηθήσεις τους συναδέλφους σου, ό φόρτος εργασίας, ο θόρυβος το φως εμμ τούτα ούλα νομίζω εν εμπόδια, συν το γεγονός ότι μπορεί κάποιες φορές ο γιατρός που σου , που απογαλακτίζει τον άρρωστο , μερικές φορές να εν λίγο πιο επιθετικός, έλα έλα γλίορα να τον φκάλουμε να τελειώνει άρα να μεν μπορείς καν αν επικοινωνήσεις, συμβαίνουν τούτα, γενικά τούτα θεωρώ εγώ, ίσως ας πούμε αν ήταν ένας χώρος πιο private και ήξερες ότι ένας άρρωστος ένα φκει που τον αναπνευστήρα και ένα βάλω την Antria μόνο σε τούτο τον ασθενή για να έχει παραπάνω χρόνο να γίνει η διαδικασία πιο ομαλή, να μεν υπήρχαν τόσο πολλά να μεν υπήρχαν τούτα τα εμπόδια, αλλά εν γίνεται τις παραπάνω φορές

**Μάλιστα, ήθελα να μου πεις κάτι άλλο που εν πολλά σημαντικό, άρεσε μου πάρα πολλά ας πούμε τώρα είπες μου ότι έχετε ασθενείς με covid σωστά;**

Τώρα δεν έχουμε, είχαμε όμως

**Είχατε τέλος πάντων , φαντάζουμε επειδή είμαι και εγώ στον χώρο υγείας τέλος πάντων, ότι πρέπει να ντυθείτε να βάλετε τις μάσκες κλπ κλπ,**

Ναι

**Διαφοροποιείται, πρόσεξες ότι χρησιμοποιείς άλλους τρόπους σε ασθενείς οι οποίοι, πριν το covid πχ που ήσασταν εκεί στην μονάδα χωρίς να φορείτε τούτα ούλα τα πράματα για να επικοινωνήσεις σε σχέση με τους ασθενείς που φορείς τούτα ούλα τα πράματα, τροποποιείται η επικοινωνία;**

Σίγουρα

**Δηλαδή τι διαφορετικό προσέχεις;**

Βασικά, γενικά επειδή ντυμένος covid είσαι πιο λίγη ώρα στον χώρο, αναγκαστικά έχεις πιο λίγο χρόνο να δώσεις στον ασθενή, δηλαδή πες ότι έχεις 6 ασθενείς και θα μήνες πχ τρεις ώρες στο τμήμα, πρέπει να γίνουν κάποια πράματα άρα δεν έχεις χρόνο να επικοινωνήσεις, άρα εν μειωμένος ο χρόνος σου, δεύτερο δεν βλέπεις καλά τρίτο δεν ακούεις καλά και νομίζω ότι γενικά όταν είσαι έτσι ντυμένος και το στρες εν πιο ψηλά δυσκόλευσε να επικοινωνήσεις, τώρα μιλώ πολλά σε λίγο μπορεί να μεν αναπνέω και καλά, εγώ προσωπικά τον πρώτο καιρό που ακόμα δεν είχαμε συνηθίσει επειδή έχω και άσθμα προσπαθούσα να μεν μιλώ όταν έμπαινα μέσα, επικοινωνούσαμε με άλλους τρόπους ας πούμε, επειδή με τούτο τον άρρωστο πήρε πάρα πολύ καιρό μέσα και ηταν σε καταστολή πάνω από ένα μήνα και μετά ετραχειοστομήθηκε ,όταν ξεκινήσαμε να επικοινωνούμε πχ καταλάβαινα ότι πονούσε τα άκρα του πάρα πολλά ότι είχε νευρομυοπάθεια λόγω της καταστολής, αντί να του μιλώ πολλά έκαμα του μασάζ

**Οκ**

Και ήταν ένας άλλος τρόπος επικοινωνίας, ή ξέρω εγώ μετά που μιλήσαμε

**Πως μιλήσετε δηλαδή τι κάμνετε για να καταλάβεις τι θέλεις, τι σου έκαμνε ένας ασθενής και τι έκαμνες εσύ**

Εε βασικά εε έπιανα να του βάλω το πόδι του πάνω σε μαξιλάρι και καταλάβαινα τον ότι πονούσε υπερβολικά

**Με τις εκφράσεις του πχ**

Ναι έκανε γκριμάτσες πόνου έντονες κλπ, οπότε έλεγα του θέλεις να σου κάμω μασάζ; Και αμέσως έπεφταν οι σφίξεις του , οι πιέσεις του, δηλαδή έβλεπες μια τεράστια αλλαγή και χωρίς να μιλήσει και χωρίς να μου πει ναι η όχι, και επομένως ήταν , ένιωθα εγώ αφού δεν μπορούσαμε να μιλήσουμε πολλά ή να μου απαντήσει και ότι φόρτιζε τον τούτο το πράμα ότι αν του έκαμνα μασάζ να εν πιο κλά, αυτά και επειδή οι φυσιοθεραπευτές είχαν πάρα πολλά λίγο χρόνο να μπουν μέσα άρα πολλά πράματα έπρεπε να τα κάμουμε εμείς

**Ενώ αν δεν εντεινόσουν στους άλλους σου τους ασθενείς πριν τον καιρό του covid που δεν χρειαζόσουνα να ντυθείς θα έκαμνες περισσότερα πράματα για να επικοινωνήσεις πιστεύεις αν είχες παραπάνω ώρα**

Βασικά ναι που την στιγμή που είσαι στον ίδιο χώρο συνέχεια, τυχαίνει πολλές φορές επειδή εν το τραπέζι του αρρώστου μπροστά στο κρεβάτι του που εν τα διαγράμματα του πάνω που θα γράφεις διάφορα μπορείς εκείνη την ώρα που γράφεις να επικοινωνείς ταυτόχρονα, εννοώ εν καιρό covid ήταν όλη η γραφική ύλη εκτός της μονάδας, αρα δεν είχες χρόνο μπροστά στον ασθενή, έκαμνες ότι ήταν να κάμεις και μετά έφευγες που κοντά του και για να μειώσεις και το υιικό φορτίο υποτίθεται, να μην είσαι πολλή ώρα μπροστά του, αυτά

**Μάλιστα**

Ναι

**Όταν χρησιμοποιείς οποιαδήποτε, να σου το πω λίγο διαφορετικά, στρατηγική για να επικοινωνήσεις με κάποιος ασθενείς, ήβρες εσύ ως Antria κάτι που σε βοηθά ας πούμε με τον καιρό ή .. τρόπους ας πουμε που σε βοηθήσαν με τον καιρό πχ να βοηθάς πιο καλά με τούτους τους ασθενείς**

Εεε γενικά επειδή αρέσει μου πάρα πολλά να τραγουδώ εε τραγουδώ τους , εε νιώθω ότι τούτος είναι ένας , βασικά αποφορτίζει τους πάρα πολλά και είχε φορά που είχαμε ασθενή που όταν είδαμε την σε άλλη φάση , ήρθε σαν επισκέπτρια και αναγνώρισε τις φωνές μας

**Αλήθεια μπράβο**

Ναι ,βασικά εντάξει, γενικά επειδή υπάρχουν και πολλές μελέτες όσο αφορά μουσικοθεραπεία κλπ εε εγώ προσωπικά νιώθω ότι βοηθά με και εμένα και τον ασθενή

**Πολλά ωραία μπράβο ,πολλά πρωτότυπο τούτο, άκουσα κάτι παρόμοιο που άλλους συναδέλφους , αλλα χαίρομαι να ακούω κάτι το διαφορετικό ας πούμε και κάποιον που νοιάζεται παραπάνω**

Όντως

**Όταν οι ασθενείς Antria μου δεν, φαντάζομαι ότι υπάρχουν και κάποιες φορές που δεν καταλαβαίνεις τι θέλει να πει ο ασθενής, έτυχε σου ;**

Ναι

**Να μεν το πάρω δεδομένο, φαντάζομαι έτυχε σου**

Οι οι, έτυχε μου πολλές φορές

**Ωραία, να σε ξαναρωτήσω για , ποιος εν ο λόγος πιστεύεις που δεν κατάφερες να επικοινωνήσεις με τον ασθενή όταν δεν κατάφερες**

Εε, ο πρώτος λόγος νομίζω είναι ότι βιαζόμουν γιατί είχα να κάμω πολλά πράματα ταυτόχρονα, δεύτερο ο ίδιος ασθενής έχει φορές που εν συγχυτικοί έχει ασθενείς που έχουν άνοια και μπορεί όντως να λέουν πράματα που να είναι λέξεις χωρίς νόημα ας πούμε, ο σωλήνας εν μεγάλο εμπόδιο εννοείται εεε τι άλλο νομίζω γενικά τούτα και η φασαρία μέσα στο τμήμα κλπ, δεν έχει φωνή να σου το πει, άρα πρέπει να είναι όσο πιο ησυχία είναι τόσο πιο εύκολα θα καταλάβεις, αυτά, επομένως, εγώ πολλές φορές δίνω τους χαρτί και μολύβι να μου γράψουν

**Μάλιστα, οι ασθενείς, εσύ ας πούμε ρωτάς του ή ενημερώνεις του κλπ, οι ασθενείς πως προσπαθούν να επικοινωνήσουν με εσένα, είπες μου τις εκφράσεις του προσώπου, κάμνουν κάτι άλλο εχτός που τις εκφράσεις του προσώπου ας πούμε**

Κινούν χέρι και πόδια εε προσπαθούν να σηκωστούν, εε δείχνουν σου πόσο τους ενοχλεί ο σωλήνας, το πρώτο πράμα που θα κάμουν είναι να τον δαγκώσουν σίγουρα, νομίζω δεν έχει κάποιο που δεν το κάμνει, τούτα, το πρόσωπο τους όμως εν πάρα πολλά έντονο, εννοώ ότι εν ο καλύτερος τρόπος επικοινωνίας και προσπαθούν να μιλήσουν και μετά καταλαβαίνουν ότι δεν βγαίνει η φωνή τους

**Μάλιστα ένας ασθενής που δεν καταφέρνει να επικοινωνήσει μαζί σου, πως πιστεύεις ότι νιώθει εκείνη την ώρα;**

Νιώθει σίγουρα ανησυχία το στρές του μεγαλώνει πάρα πολλά και μετα ξεκινά να έχει και σωματικα, θα έχει κλινική εικόνα, συνήθως να ανεβάσει πιέσεις θα ανεβάσει σφίξεις θα κάνει ταχύπνοια , θα ιδρώσει, ένα γίνει ολοκόκκινος εεμ , πρέπει να νιώθουν πάρα πολλά αβολά, μεγάλη ανησυχία

**Μάλιστα, εσύ ας πούμε, κάμνει σε να νιώθεις κάτι αμαν κάμνεις κάποιες προσπάθειες και δεν καταφέρνεις να επικοινωνήσεις με τον ασθενή, πως νιώθεις εσύ ως Antria ας πούμε;**

Και εγώ νιώθω άβολα και ανησυχία, ψεε νιώθω ότι μεγαλώνει και η κούραση μου, δηλαδή στην προσπάθεια μου να καταλάβω και να μεν συνεργάζεται ,γιατί σε μια φάση προσπαθεί να επικοινωνήσει και άμαν δεν τα καταφέρει σημαίνει ότι δεν θα συνεργαστεί μαζί σου, εε θα κάμνει αλλά των άλλων πράματα, άρα μεγαλώνει και ο κόπος σου εσένα

**Μάλιστα κατάλαβα, είπες μου πριν ότι χρησιμοποιείτε, έτυχε σε ασθενείς, να τους δώσεις μολύβι ή πέννα και χαρτί και έγραψαν σου**

Ναι

**Πολλά ωραία, χρησιμοποιείς οποιουσδήποτε άλλους τρόπους ας πούμε εχτός που την πέννα και χαρτί, κάποια άλλα βοηθητικά μέσα, όπως να σου δώσω παραδείγματα πχ ταμπλετ, κινητά, πίνακες με σχέδια ή πίνακες με λόγια**

Χρησιμοποιούμε εεε την κλίμακα με τις εκφράσεις του πόνου ,είχαμε ασθενή που είχε gullain bare και δεν μπορούσε να κάμει καμιά κίνηση στο πρόσωπο που σιγά σιγά χρησιμοποιούσαμε τον πίνακα με τα γράμματα, ήρθε λογοθεραπεύτρια και έδειξε μας ακριβώς πως να τον χρησιμοποιούμε και με τα χρώματα αλλά σε πολλά λίγες περιπτώσεις τον χρησιμοποιήσαμε, το τάμπλετ τα τελευταία χρόνια , ε τα κινητά τους οι τόσο πολλά, εε , τί άλλο; Τούτο, παραπάνω μολύβι και χαρτί

**Τον πίνακα μου είπες πολλά λίγο τον χρησιμοποιήσατε, για πιο λόγο δηλαδή, δεν σας βόλευε, είχε κάποιο θέμα;**

Οι ότι δεν μας βόλευε, συνήθως δεν έχουμε ασθενείς με νευρολογικά προβλήματα τόσα πολλά που να πρέπει να χρησιμοποιήσουμε έτσι πίνακες, συνήθως επικοινωνούμε πιο γρήγορα και πιο εύκολα μαζί τους, τούτος ο ασθενής ήταν ένα πολλά βαρύ qullain bare που δεν μπορούσε να κάμει τίποτε, άρα δεν είχαμε άλλο τρόπο να επικοινωνήσουμε, δεν έδειχνε τίποτε το πρόσωπο του επομένως έπρεπε να βρούμε ένα άλλο τρόπο επικοινωνίας, συνήθως επειδή οι ασθενείς ή θα αποσωληνωθούν ή ένα τραχειοτομηθούν επικοινωνούμε πιο πολλά με τούτες τις μεθόδους που σου είπα παρά με πίνακες

**Οκ, με το τάμπλετ, δλδ έχετε κάποιο πρόγραμμα πάνω στο ταμπλετ και επικοινωνείτε, δηλαδή πως ακριβώς, γράφουν σας πως επικοινωνείτε με το τάμπλετ**

Συνήθως η ένα γράψουν αλλά παραπάνω το τάμπλετ εν για , είναι και για να δουν κάτι για να ακούσουν κάτι, παραπάνω για να κοινωνικοποιηθούν παρά να μας γράψουν, εε εν μπορώ τώρα να, βασικά προγράμματα όχι, παρόλο που θα ήταν πάρα πολλά καλό να είχαμε κάτι τέτοιο πάνω στα ταμπλετ

**Μάλιστα το παρελθόν ή γενικά ξαναδέχτηκες ή στο πτυχίο σου ή στα course που έκανες κλπ, ξανάκανες πότε οποιαδήποτε εκπαίδευση όσο αφορά την επικοινωνία με διασωληνομένους ασθενείς να μεν σου πω διασωληνομένους ξύπνιους ή αν έκαμες και με διασωληνομένους ξύπνιους**

Βασικά κάμαμε εκπαίδευση όσο αφορά την επικοινωνία με μεταξύ μας με τους ασθενείς όχι

**Μεταξύ σας εννοείς τους επ. υγείας;**

Ναι

**Α οκ**

Τούτο το πράμα νομίζω θα ήταν πάρα πολύ καλό μέσα στο course γιατί σαν και πρέπει να βρούμε που μόνοι μας τρόπους επικοινωνίας ενώ κάμνεις τόση μεγάλη εκπαίδευση και τούτα τα πράμα αφήνουν τα πίσω

**Εσύ ως Antria τι πιστεύεις ότι θα ήταν καλύτερο να εκπαιδευτείς σε μια τάξη και να κάμνετε μάθημα σε πίνακα, θα σε βοήθαν μέσω τηλεπικοινωνίας , δίπλα που τον ασθενή, συνδυασμός, να σ δώσουν υλικό;**

Θα με βοηθούσε να εγίνετουν εκπαίδευση σε τάξη και μετά εφαρμογή στον χώρο

**Δηαλδη να κάμνεις μια εκπαίδευση σε τάξη και μετά να είσαι στην ΜΕΘ και να σου δείχνουν έμπρακτα ενοείς**

Ναι

**Οκ, μάλιστα,έχεις, είπες μου**

Αλλά και να είχε μόνιμα στο τμήμα εε ομάδα που να ασχολείται με τούτο το θέμα, δηλαδή ας πούμε, οσο είχε, πλεον δεν ξέρω αν έχεις στην ΜΕΘ της Λευκωσίας, ψυχολόγο, ήταν πάρα πολλά καλό, εργοθεραπευτή, λογοθεραπευτή πρέπει να τον καλέσεις και όταν θα έρθει είναι ολόκληρη υπόθεση ενώ έπρεπε να ηταν κομμάτι της, όπως εν ο φυσιοθεραπευτής , έπρεπε να ηταν και ο λογοθεραπευτής

**Δεν σου έκαμα την πιο αυτονόητη ερώτηση, ερώτησα σε πως θα σε βοήθαν, εν σε ρώτησα όμως ένα ήθελες να εκπαιδευτέις αν σου δίνετουν η ευκαιρία;**

Ναι

**Χαιρόμαι**

Και εγώ

**Γενικά είπες μου για ένα περιστάτικο που είχετε πριν με τον κορονοίο πολύ καιρό, έχεις οτιδήποτε που σου έρχεται εσένα τώρα έτσι ξαφνικά στην μνήμη σου για οποιοδήποτε λόγο που θέλεις να μου πείς**

Τούτη η κοπέλα που σου είπα πριν, ηταν μια πολυτραυματίας που όλα τα τραύματα ηταν στο πρόσωπο και το πρόσωπο της ηταν παραμορφωμένο, δηλαδή δεν αναγνωρίζαμε τα χαρακτηριστικά του προσώπου της, ηταν σε φουλ καταστολή, εε και είχα την ,επειδή κάθε μέρα έχουμε που ένα ή δυο ασθενείς το πολύ, έτυχε να είναι ασθενής μου τρείς τέσσερις μέρες συνεχόμενα, έφυγε σε καταστολή και μεταφέρθηκε στην Λευκωσία γιατί ήθελε γναθοπροσωποχειρούργους για να συνεχίσει την θεραπέια της στην Λευκωσία, ύστερα που κάμποσο χρονικό διάστημα είχαμε άλλη πολυτραυματία στο ίδιο κρεβάτι και ηταν η ώρα του επισκεπτηρίου , αλλα εγώ κάτι έκανα δεν ύμουν μπροστά στην άρρωστη και είχε μπροστά στην κοπέλα ένα ζευγάρι που ηταν οι επισκέπτες της, σε κάποια φάση κάποιος μου εφώναξε και απάντησα και αμέσως γυρίζει που ηταν η επισκέπτρια και λέει μου ξέρω σε , λαλώ της εγώ εν σε ξέρω, σίγουρα δεν με ξέρεις δεν μπορείς να με καταλάβεις γιατί ημουν χτυπημένη στο προσώπο και δεν αναγνωρίζεται το πρόσωπο μου, ήμουν σε τούτο τον χώρο είχα χτυπήσει δεν ξέρω πως σε λένε , όμως εμένα λεουν με Αννίτα λέει μου και έχεις και εσύ μια κόρη Αννίτα

**Μάνα μου ρε**

Ναι, και ετραγούδας μου Αλκίνο Ιωαννίδη, εθυμάτουν τα τραγούδια που της έλεα και λεέει μου θυμούμαι πάρα πολλά καλά την φωνή σου, δεν σε είδα ποτέ όμως , αυτά, Εεεε, βασικά τούτο έτυχε πολλές φορές , οι παραπάνω που τους ασθενείς εε ακούν τα πάντα όσο καταστολή και αν παίρνουν

**Ενοείς και φουλ κατεσταλμένοι να είναι ότι ακούν τα πάντα ενοείς**

Ναι απλά κάποια που τα κατασταλτικά κάμνουν αμνησία δεν θυμούνται όμως ακούν

**Μάλιστα αρα με τούτη την κοπέλα δεν έφτασε να μπει σε φάση weaning , να επικοινωνήσετε,**

όχι δεν ηταν σε φάση weaning εκράτησε την πάρα πολλά το γεγονός ότι άκουε μας, γιατι θυμάτουν και ας πούμε , λεουν το μετά οι παραπάνω ασθενείς, ότι τούτο που έκαμνες εκράτησες με η ξύπνιο ή να θέλω να τα καταφέρω όπως και πάρα πολλά πράματα έτσι και όπως και ο τελευταίος ασθενής που σου είπα με τον covid που άκουε μας και εβοηθήσε τον πάρα πολλά να καταφέρει να βγεί

**Μάλιστα, οκκ**

Αυτά, δεν θυμούμαι κάτι άλλο να σ πω τωρά, έχει πολλά πράματα

**Μάλιστα Antria μου, ευχαριστώ πάρα πολλά**

Νο 10

**Bold**: researcher

Unbold-normal letters: georgia

**Nα σου υπενθυμίσω ότι κάνω μια έρευνα όσο αφορά τις εμπειρίες επικοινωνίας των νοσηλευτών που δουλεύουν στις ΜΕΘ με διασωληνομένους ξύπνιους ασθενείς, αυτά θα τα πούμε μετά, τα πρώτα πράγματα που θα ήθελα να σε ρωτήσω γενικά για το background σου, το πρώτο πράγμα που θα ήθελα να σε ρωτήσω είναι πόσα χρόνια εργάζεσαι ως νοσηλεύτρια, γενικά ως νοσηλεύτρια;**

Ως νοσηλεύτρια εργάζομαι από το 2005, τελείωσα το 2004

**15 χρόνια δηλαδή**

Ναι 15 χρόνια

**Οκ**

Εμπειρία εντατικής έχω τα τελευταία 7 χρόνια

**Άρα είσαι στην εντατική 7 οκ, που έχεις τελειώσει αν επιτρέπετε;**

Tέλειωσα Ελλάδα, στα ΤΕΙ Αθήνας , έκανα μεταπτυχιακό εδώ στην Κύπρο στο ανοικτό πανεπιστήμιο Κύπρου

**Σε τι κατεύθυνση έκανες το μεταπτυχιακό;**

Έκανα πολιτική της υγείας και στατικού σχεδιασμού μονάδων υγείας συγκεκριμένα και παράλληλα εκπαιδεύσεις σεμινάρια συνέδρια , όλα αυτά

**Οκ, μου είπες πριν λίγο ότι προσπαθείς να κάνεις ένα διδακτορικό, τι αφορά το διδακτορικό σου δηλαδή;**

Eνα διάστημα είχαμε ένα ερευνητικό πρόγραμμα στην εντατική το οποίο έχει να κάνει με κρανιοεγκεφαλικούς ασθενείς, τροχαία ατυχήματα δηλαδή και υπήρχε ένα ευρωπαϊκό πρόγραμμα και ήταν και η Κύπρος μαζί με άλλες χώρες που καταγράφουμε δεδομένα ασθενών που έρχονται στην εντατική, εε με προβλήματα κρανιοεγκεφαλικών κακώσεων και μαζεύαμε κάποια δεδομένα σε αυτό το πρόγραμμα και βάση των δεδομένων προσπαθούμε τώρα να βγάλουμε ένα αποτέλεσμα αν υπάρχουν κάποιοί προγνωστικοί παράγοντες ,αν μπορούσε να υπάρχει μια προ διαθεσιμότητα και όλα αυτά, τώρα είμαστε βέβαια σε αρχικό στάδιο γι’ αυτό είμαι σε μια στάση που θέλω λίγο να το δω και πως μπορεί να βγει αυτό

**Μάλιστα , οκ, το πρώτο που θα ήθελα να σε ρωτήσω Georgia μου, για να μπω και εγώ λίγο στο κλίμα αφού ούτε εγώ δεν δουλεύω σε μονάδες , στην δική σας την Μονάδα σε ποιες περιπτώσεις θα βρω ένα διασωληνωμένο ξύπνιο ασθενή;**

Διασωληνωμένο ξύπνιο ασθενή, να διευκρινίσουμε τι λέμε διασωληνωμένο..

**Όχι τραχειοτομημένο, διασωληνωμένο**

Μάλιστα , θα δεις ένα άρρωστο διασωληνωμένο ξύπνιο στην διαδικασία της αφύπνισης, δηλαδή η διαδικασία που κάνει ο γιατρός αφύπνιση ενός ασθενή που έχει μια μορφή πνευμονίας ή μια ΚΑΚ και θέλουμε να δούμε νευρολογική εικόνα, εε ξύπνιοι εννοούμε όμως, μπορώ να ρωτήσω;

**Ναι** **βεβαίως**

Ξύπνιοι εννοούμε ότι ανταποκρίνεται, καταλαβαίνει , ότι είναι τα μάτια του ανοικτά, τι εννοούμε ξύπνιο;

**Ξύπνιο βασικά, πολύ ωραία ερώτηση , που δεν το διευκρίνισα λάθος μου, εννοώ να είναι off sedation, να μην έχει κάποιες καταστολές ή μόλις να μου αναφερθείς και σε άτομα που μόλις έκλεισες τις καταστολές ή σε άτομα που πέρασε ώρα χωρίς καταστολή , εννοώ αυτό εύρος όλο**

Είναι η φάση που σου είπα ότι είναι η καθημερινή ρουτίνα των γιατρών που κόβουν τις καταστολές και περιμένουμε να δούμε τι θα κάνει ο ασθενής το οποίο συνήθως εγώ προσωπικά το έχω μετρήσει έτσι λίγο με την ώρα , παίρνει γύρω στις 3-4 ώρες αυτό να περάσει για να δούμε τελικά τι εξέλιξη θα υπάρχει, ξύπνιο διασωληνωμένο ασθενή μπορούμε να δούμε και χωρίς στον αναπνευστήρα άρρωστο να είναι ενωμένος με ένα Τ οξυγόνου και να είναι ενωμένος στο ροόμετρο και να δούμε αν μπορεί να αποσωληνωθεί το οποίο αυτό είναι τελικό στάδιο αφύπνισης που σημαίνει ότι ο ασθενής διατηρεί τον αεραγωγό του ότι είναι οκ να ξυπνήσει και εκεί που δεν έχει καταστολές αλλά δεν είναι ξύπνιος είναι στους εγκεφαλικά νεκρούς, όταν έχουμε κάνει τέστ εγκεφαλικού θανάτου που πλέον οι αρρώστοι δεν παίρνουν καταστολές αλλά είναι διασωληνωμένοι αλλά δεν είναι ξύπνιοι όμως

**Οκ, θα ήθελα να διευκρινίσω το Τ είναι το λεγόμενο t piece που λέμε;**

Nαι, το ενώνουμε κατευθείαν πάνω στον ενδοτραχειακό και το ενώνουμε κατευθείαν πάνω στο ροόμετρο του οξυγόνου, εκεί βλέπεις ένα άρρωστο ο οποίος έχει μια κλίμακα Γλασκόβης 11/15, δηλαδή είναι full conscious ας πούμε χάνει τους πόντους επειδή έχει τον ενδοτραχειακό, εκεί χάνει 4 πόντους, αλλα είναι μια φάση, ένα τελικό στάδιο πριν βγει ο σωλήνας

**Εννοείς τους βγάζετε τελείως από τον αναπνευστήρα και τους βάζετε σε ένα απλό οξυγόνο μαζί με τον ενδοτραχειακό, αυτό εννοείς, να το διευκρινίσω κι εγώ για να καταλάβω καλά**

Ναι

**Μάλιστα, άρα αυτές είναι οι περιπτώσεις που είδες εσύ με τον καιρό κλπ ότι μπορώ να έβρω ένα διασωληνωμένο ξύπνιο, θα ήθελα να σε ρωτήσω γενικά πως είναι να δουλεύεις με αυτούς τους ασθενείς;**

Εεε για εμένα προσωπικά, θα σου δίνω προσωπική απάντηση γιατι φαντάζομαι ο κάθε ενας το βλέπει πολύ διαφορετικά

**Ναι αυτό προσπαθώ να δώ**

Γιατί θα μπορούσε κάποιος να σου πει ααα δεν μπορώ να συνεργαστώ με ξύπνιο ασθενή θέλω να κοιμάται, θέλω να έχει καταστολές ,θέλω να κοιμάται για να μην κάνει τίποτα, εγώ θεωρώ ότι πρόκληση είναι αυτή, να σταματήσεις τις καταστολές να δεις τι θα κάνει τούτος ο άρρωστος θα ξυπνήσει ή βλέπουμε και παράλληλα των αναπνευστήρα εκεί που κάνει και δικές του αναπνοές ας πούμε αφού έχεις κλίσει τις καταστολές και αφού τον έχεις βάλει σε ένα πρόγραμμα αναπνευστήρα ο οποίος δεν είναι 100% μηχανικός αερισμός από τον αναπνευστήρα και κάνει ο άρρωστος δικές του αναπνοές, εκεί σιγά σιγά εγώ προσωπικά αρχίζω κι τους μιλάω, βρίσκεσαι και στην εντατική ας πούμε είναι μέσα Δευτέρα και τέτοια πράγματα, αυτό, εγώ το θεωρώ πάρα πολύ σημαντικό αυτό το κομμάτι, γιατί αυτό είναι μια διαδικασία η οποία γίνεται κάθε μέρα στην εντατική , δηλαδή να έχω ένα άρρωστο ο οποίος να ξυπνάει αλλά δεν είναι πολύ καλός πρέπει να ξαναξεκινήσω τις καταστολές, την επόμενη μέρα ξανά το ίδιο , δηλαδή είναι μια διαδικασία η οποία είναι ρουτίνα για μερικούς αλλά εγώ το θεωρώ μεγάλη πρόκληση αυτό το πράγμα

**Πως είναι ,τώρα θα πιάσω το κομμάτι της επικοινωνίας, δηλαδή πως καταφέρνεις να επικοινωνήσεις με ένα διασωληνωμένο ξύπνιο ασθενή, ποιοι είναι οι τρόποι που χρησιμοποιείς για να επικοινωνήσεις, μιλώ από τα πιο απλά μέχρι τα πιο σύνθετα**

Εε σίγουρα του φωνάζουμε το όνομα του , κύριε Τάδε ας πούμε καλημέρα είσαι στην εντατική ,του λέω αν με ακούς σφίξε μου το χέρι ή άνοιξε μου τα μάτια για να δω αν με καταλαβαίνεις ή βγάλε μου την γλώσσα έξω, συγκεκριμένα την γλώσσα έξω είναι μια φράση που την λένε πάρα πολλοί γιατροί για να δουν αν μπορούν να κρατήσει αεραγωγό ο ασθενής εε ή κούνα μου τα πόδια ή σήκωσε μου το χέρι, δηλαδή για να δούμε αν εκτελεί ο ασθενής και αν σε ακούει, αυτό

**Μάλιστα, οκ θα στραφώ στο γεγονός, είπες μου πριν που μιλούσαμε αρχικά ότι σε μεταφέραν στην εντατική του covid, φαντάζομαι από την εμπειρία μου και εγώ ότι για να μπείτε σε αυτούς τους ασθενείς πρέπει να βάλετε κάποια ‘’Α’’ στολή ας πούμε ότι πρέπει να ντυθείτε που πάνω ως κάτω να βάλετε την μάσκα, να βάλετε την ασπίδα κλπ κλπ, εε έχεις προσέξει να χρησιμοποιείς διαφορετικούς τρόπους επικοινωνίας με αυτούς τους ασθενείς σε σχέση με πιο παλιά που δεν είχαμε αυτή την πανδημία και δεν φορούσες κάτι;**

Εεεε κοίταξε να δεις επειδή μέχρι στιγμής υπήρχαν 3 περιστατικά που τα καταφέραν με την έννοια ότι αποσυνδεθήκαν από τον αναπνευστήρα ότι ήταν καλά ότι ήταν με μια απλή μάσκα ventouri ότι καταλαβαίναν οι άνθρωποι και ότι μας βλέπανε ξέρω γώ, εεε αυτό που λέμε βασικά είναι να μην φοβούνται που μας βλέπουν έτσι , γιατί εντάξει η εμφάνιση τρομάζει , γιατί βλέπεις ένα άνθρωπο πάνω από το κεφάλι σου φοράει μάσκες ασπίδες σκουφιά, γάντια ρόμπες ξέρω γώ δεν έχει αλλάξει κάτι στον τρόπο προσέγγισης απλά πιστεύω ότι αυτό που άλλαξε είναι αυτό που ΄λέω μην φοβάσαι , είμαι η ταδε φοράω τα ρούχα αυτά γιατί υπάρχει αυτός ο ιός, είσαι καλά είσαι στην εντατική κλπ κλπ τα κατάφερες, αυτό..

**Άρα κάνεις τις ίδιες κινήσεις φαντάζομαι τα ίδια λές ,εννοώ δεν διαφοροποιήθηκε τόσο πολύ; Ok**

Ναι

**Οι ασθενείς πως επικοινωνούν με εσένα, μπορείς να μου κάνεις μια αναπαράσταση για παράδειγμά, εννοω πες είμαι ο ασθενής που είμαι διασωληνομένος και ξύπνιος πως θα μου μιλήσεις τι θα μου πέις ή αν δεις ότι είμαι αναστατωμένη τι θα κάνεις;**

Εεε

**Στα έμπλεξα λίγο..**

Αν είσαι αναστατωμένη ας πούμε ,γιατί όλοι παθαίνουν ένα πανικό όταν είναι σε ένα χώρο άσχετο και έχουν ένα σωλήνα μέσα στο στόμα τους, το πρώτο πράμα που θέλουν να κάνουν είναι να τραβήξουν τον σωλήνα να βγεί ,τους καθησυχάζω, τους λέω είσαι στην εντατική έχεις ένα σωλήνα στον στόμα σου , θα προσπαθήσουμε μαζί σιγά σιγά να το βγάλουμε εε θέλω να δω αν μπορείς, αν με καταλαβαίνεις αυτό που θα μου κάνουν είναι να τους πω κλείσε μου τα μάτια ας πούμε, θα κλείσουν τα μάτια, σφίξε μου το χέρι, βγάλε μου την γλώσσα έξω, θα μου βγάλουν την γλώσσα έξω και θα τους καθησυχάσω με την έννοια σε λίγο θα βγάλουμε τον σωλήνα, γιατί περισσότερο αυτή είναι η ανησυχία τους ότι έχουν ένα σωλήνα στο στόμα και ότι θέλουν να βγει…

**Γενικά αυτά τα άτομα που προσπαθούν να σου μιλήσουν όταν είναι ξύπνια, τι πιστεύεις εσύ ή τι είδες με τον καιρό ότι προσπαθούν να σου πουν, ποιες είναι οι ανάγκες τους; Τι σου ζητούν;**

Έχουν την ανάγκη να κρατηθούν από κάπου , νιώθουν ότι είναι αβοήθητοι ότι έχουν ένα συναίσθημα ότι κρέμονται από πάνω σου ότι θέλουν ένα χέρι βοηθείας και + της άλλης ότι είναι σε ένα περιβάλλον το οποίο είναι άγνωστο οι ανάγκες τους είναι να έχουν παραπάνω ένα άνθρωπο δικό τους στην εντατική , πράγμα το οποίο δεν μπορεί να γίνει, γιατί είναι εντατική ,εεε πολλοί ας πούμε ρωτούν μόλις ξυπνήσουν ότι θέλω να δω την γυναίκα μου θέλω να δω τα παιδία μου θέλω να δω τον φίλο μου την φίλη μου , ας πούμε ένα άνθρωπο δικό τους ο οποίος είναι οικείος, όχι ένα άνθρωπο ο οποίος είναι ξένος , εμείς ας πούμε ο γιατρός, όλα αυτά.

**Εχτός από τις ανάγκες τους τι προσπαθούν να σου πουν γενικά μόλις ξυπνήσουν;**

Γενικά τι μου έχουνε πούνε μέχρι στιγμής;

**Ναι ας πούμε πάνε να επικοινωνήσουνε μαζί σου, τι πιστεύεις ότι θέλουν να σου πουν εκείνη την ώρα, πχ είμαι ένας ασθενής ξυπνώ τι είναι αυτό, εντάξει ο κάθε ασθενής φυσικά είναι διαφορετικός εννοείται αλλά τί είναι αυτό που παν να ρωτήσουν ή να κάνουν , τι είναι αυτό που παν να επικοινωνήσουν;**

Συνήθως το πρώτο πράμα που λένε, είναι καλό σημάδι αυτό , θέλουν νερό, διψάω ή πεινάω , βασικές ανάγκες ή κάτι θέλουν να φύγουν, θέλουν να φύγουν από εδώ μέσα έχω ακούσει, εε να μου πούνε κάτι άλλο, ντάξει μερικοί έχουν εκφράσει και ότι πέθανα ας πούμε, πήγα και ήρθα;

**Όλα αυτά που σου λένε ή το πεινάω ή το διψάω αφού είναι διασωληνωμένοι πως το αντιλαμβάνεσαι εσύ, πως καταλαβαίνεις ότι σου λέει πεινά ή διψά ή θέλω να φύγω για πχ;**

Με το στόμα με τα χείλη, διαβάζω τα χείλη τους

**Υπάρχουν άλλοι τρόποι γενικά που χρησιμοποιείς και δεν τους αναφέραμε; Είπες μου για παράδειγμα τα χείλη ή τους μιλάς τους λές, μπορείς να σκεφτείς κάτι άλλο που χρησιμοποιείς εσύ και δεν το αναφέραμε;**

Eεε να σκεφτώ, μάτια χέρια να μου κουνήσουν τα πόδια να μου σηκώσουν το χέρι να μου σφίξουν το χέρι, νομίζω αυτά..

**Οκ τι πιστεύεις ότι σε εμποδίζει να επικοινωνήσεις με αυτούς τους ασθενείς, τι εμπόδια βρίσκεις ανάμεσα στην επικοινωνία σου με αυτούς τους ασθενείς ;**

Υπάρχουν φορές που δεν μπορώ να τους καταλάβω , δηλαδή θα ήθελα ας πούμε να , μπορεί να μασάνε τα λόγια τους, λογικό ή να είναι ανήσυχοι ή να τους βλέπεις ρε παιδί μου ότι θέλουν κάτι και να μην μπορείς να τους καταλάβεις, εκεί είναι το λάθος που κάνουν ορισμένοι νοσηλευτές κυρίως της εντατικής, αα είναι συγχυσμένος αλλά δεν είναι συγχυσμένος ο άνθρωπος, ή ξέρω εγώ είναι πάρα πολύ ανήσυχος ή να τους δώσουμε κάτι .. κατάλαβες τι εννοώ, να του δώσουμε κάτι να ηρεμίσει αλλά είναι η προσπάθεια τους να επικοινωνήσουνε μαζί μας και εμείς δεν μπορούμε να καταλάβουμε τι λένε , αυτό νομίζω είναι ένα πράγμα το οποίο ακόμα δεν έχουμε βρει την λύση αυτού του προβλήματος, γιατί είναι ένα πρόβλημα, εγώ θεωρώ ότι είναι μεγάλο πρόβλημα να θέλεις να μιλήσεις σε κάποιον και να μην σε καταλαβαίνει

**Οκ χρησιμοποίησες κάποιους τρόπους άλλους ή ήβρες κάποιους τρόπους εσύ ως Georgia με τον καιρό που να σε βοηθήσει με την επικοινωνία σου με αυτούς τους ασθενείς;**

Ναι έχουμε εντάξει , στην εντατική, εκείνα τα πως τα λένε πίνακες που ξέρω γω που πονάω ή δεν είμαι καλά ή θέλω να πιώ νερό ή θέλω να φάω και τους τα δείχνεις ας πούμε για να μπορείς να καταλάβεις τι θέλουν

**Έχει λέξεις, προτάσεις, εικόνες αυτός ο πίνακας;**

Προτάσεις και εικόνες

**Το χρησιμοποιείς συχνά;**

Όχι

**Για ποιο λόγο;**

Είναι κάτι το οποίοι το χρησιμοποιούσε η ψυχολόγος που είχαμε στην εντατική που πλέον δεν υπάρχει σαν μέσο επικοινωνίας με τους ασθενείς, εγώ δεν έτυχε να χρησιμοποιήσω τον συγκεκριμένο πίνακα για να δείξω κάτι η αλήθεια είναι, δεν έτυχε να το κάνω, ο λόγος δεν ξέρω, μπορεί να είναι και θέμα χρόνου, έξτρα ας πούμε, όχι έξτρα πάνω μας, έξτρα αφιέρωση ίσως, διότι σίγουρα δεν θα έχεις ένα ασθενή που θα πεις θα αφοσιωθώ κοντά του

**Άρα και αυτό είναι ένα εμπόδιο να επικοινωνήσεις, ο χρόνος σας είναι δύσκολος , οκ , φαντάζομαι, για να μην το πάρω δεδομένο, με τον καιρό έχεις και ασθενείς που δεν καταλάβαινες τι έλεγαν, σωστά; Όπως είπαμε πριν λίγο**

Ναι υπάρχουν και άρρωστοι που έχουν κάποια νοητική στέρηση, εκει ήταν πολύ δύσκολο γενικά, μου έτυχε μόνο δύο φορές και εκεί χρειάστηκε να βάλουμε μέσα την μητέρα, η οποία φαντάζεσαι ότι είχε άλλη επαφή και επικοινωνία με το συγκεκριμένο άτομο, εκεί ήταν μεγάλη δυσκολία για να .. για εμένα είναι και λίγο άγχος γιατί δεν ξέρεις σε τι επίπεδο αντιλαμβάνεται το συγκεκριμένο άτομο και νευρολογικά και από θέμα αντίληψης και τι θα καταγράψεις; Δεν μπορείς να βάλεις κλίμακα γλασκώβης 15 σε άνθρωπο με νοητική στέρηση, κατάλαβες;

**Ναι αντιλαμβάνομαι , εμμ είπες μου ότι είναι και λίγο άγχος για εσένα τούτο, εχτός που τους ασθενείς που έχουν νοητική στέρηση που σου έτυχε ένα δυο περιστατικά, ένιωσες ποτέ έτσι συναισθήματα με άλλους ασθενείς να σε στρεσάρουν είτε θετικά είτε αρνητικά όσο αφορά την επικοινωνία, αν δεν καταφέρεις να επικοινωνήσεις; Ή τι συναισθήματα σου προκαλούν όταν θέλεις να επικοινωνήσεις με κάποιο ασθενή και για τον Α Β Γ λόγο δεν καταφέρνεις να επικοινωνήσεις μαζί του;**

Άγχος αμηχανία, ότι έχεις ένα άνθρωπο ο οποίος είναι πάνω σε ένα κρεβάτι ο οποίος εκείνη την στιγμή εσύ είσαι εκεί γι αυτόν και να μην μπορείς να τον βοηθήσεις με την έννοια ότι αυτό που σου λέει δεν το καταλαβαίνεις, εντάξει συνήθως ζητάς βοήθεια από συναδέλφους κι λες ας πούμε έλα να δεις τι θέλει , ένα λα να δούμε τι θέλει για να καταλάβουμε αυτό που θέλει..

**Μάλιστα οι ασθενείς πως πιστεύεις ότι νιώθουν αν δεν καταφέρνουν να επικοινωνήσουν ας πούμε, προσπαθούν να σου πουν κάτι και δεν καταφέρουν;**

Εγώ πιστεύω ότι νιώθουν πάρα πολύ άσχημα, γιατί, βασικά βάζω τον εαυτό μου εκείνη την στιγμή στην θέση τους που να θέλεις να πεις κάτι και να μην μπορείς, όχι, να το λες και να μην σε καταλαβαίνει ο άλλος , εε

**Ο λόγος που πιστεύεις ότι δεν καταλαβαίνεις αυτόν τον ασθενή ποιος πιστεύεις είναι ο λόγος; Δηλαδή τι διαφέρει με κάποιον άλλο ασθενή που καταλαβαίνει, είπες μου ένα παράδειγμα είναι η νοητική στέρηση, άλλο που μπορεί να παρουσιάσει κάποιος και να μην μπορείς να καταλαβαίνεις;**

Μπορεί να είναι σε φάση ντελίριο ο ασθενής σε εκείνη την στιγμή και να θεωρεί ότι βρίσκεται σε ένα χώρο σε άλλο περιβάλλον σε ένα άλλο κόσμο τελείως, εκεί είναι λίγο δυ.. εντάξει το παθαίνουν συχνά οι ασθενείς στην εντατική δεν ξέρω αν το έχεις δει ποτέ, κυρίως ασθενείς οι οποίοι έχουν μείνει αρκετό διάστημα σε ένα χώρο όπως η εντατική και παίζει ρόλο το παράθυρο και ο ήλιος, δηλαδή αν δεν βλέπουν λέει ήλιο έξω στο, αν βλέπουν παράθυρο με φως έξω, παθαίνουν πολύ συχνά έτσι αυτή την κατάσταση, εεμμ, δεν ξέρω είναι άσχημο να στρεσάροντας ας πούμε

**Μάλιστα, έχει κάποιο περιστατικό που για οποιαδήποτε λόγο σου κίνησε το ενδιαφέρον ή σου έμεινε στην μνήμη σου ξεχωριστό όσο αφορά σε θέμα επικοινωνίας, που σου έρχεται τώρα στο μυαλό, αν σου έρχεται;**

Μου έρχεται ένα περιστατικό στο μυαλό βέβαια δεν είχε να κάνει με διασωληνωμένο, ναι είχε να κάνει με διασωληνωμένο άρρωστο ο οποίος δεν ήταν στην εντατική , εε υποστηριζότανε με ένα αναπνευστήρα portable παρόλα αυτά είχε ενδοτραχειακό, μια γυναίκα η οποία είχε σκλήρυνση κατά πλάκας τελικού σταδίου, εε και επικοινωνούσε μόνο με τα βλέφαρα των ματιών της, δηλαδή ήταν η φάση της έλεγες θέλεις νερό, έκλεινε τα μάτια θέλεις κάτι, νταξει φυσικά αυτή μετά έκανε τραχειοστομία αλλά αυτό ήταν κάτι το οποίο ήταν πάρα πολύ σοκαριστικό, δηλαδή επικοινωνούσε μόνο με τα μάτια της

**Άρα εσύ ήσουν στο σημείο να της κάνεις μόνο ερωτήσεις για να καταλαβαίνεις τι θέλει**

Ναι ναι

**Δύσκολο**

Πολύ δύσκολο, αυτό έχει χρόνια βέβαια αλλά εντάξει

**Στην εκπαίδευση σου γενικά ή στο πτυχίο σου ή στο μεταπτυχιακό σου ή σε σεμινάρια κλπ εκπαιδεύτηκες ποτές σε θέματα επικοινωνίας;**

Ναι κάναμε αρκετά σεμινάρια και εντός της δουλειάς της εντατικής και με την ψυχολόγο που σου είπα ότι είχαμε κάναμε έτσι σεμινάρια επικοινωνίας και με τους ασθενείς και για τους ασθενείς αλλά και για τους συγγενείς των ασθενών το πως ας πούμε πρέπει να επικοινωνούμε μαζί τους, ήταν αρκετά βοηθητικά μπορώ να πω

**Κάμετε συγκεκριμένα για τους διασωληνομένους ασθενείς ή γενικά για την..**

Όχι όχι, για ασθενείς στην εντατική γενικά, όχι για διασωληνομένους ασθενείς, εννοώ γενικά με την έννοια ξυπνάει κάποιος αυτό που σου είπα , ή ξέρω εγώ σε τραχειοστομία ή γενικά με τις ανάγκες που έχουν

**Μάλιστα, είπες μου πριν χρησιμοποιείτε τους πίνακες ή έτυχε τέλος πάντων να χρησιμοποιήσετε κάποιες φόρες τους πίνακες, χρησιμοποιείτε οποιοδήποτε άλλο μέσο ας πούμε όπως κάτι πιο απλό, πέννα και χαρτί ή κάτι πιο σύνθετο, τάμπλετ ας πούμε, χρησιμοποιείτε τα αυτά για να σας βοηθήσουν με την επικοινωνία με τον ασθενή**

Ταμπλετ δεν υπάρχει, πέννα και χαρτί όμως γίνετε, γίνεται συχνά , αυτό γίνεται ή συνήθως φέρνουν οι συγγενείς μπορεί κάποιο κινητό τηλέφωνο, τάμπλετ είχε γίνει, μια φορά σε μια άρρωστη αλλά είχε τραχειοτομία όμως αυτή

**Πέννα και χαρτί βοηθήστε συνήθως ή το βρίσκεις ότι δεν σε βοηθά τόσο;**

Όχι βοηθάει, αλλά απλά είναι λίγο δύσκολο, επειδή είναι στην φάση που ξυπνά ,επειδή είναι η φάση που ξυπνάει δεν ξέρω.. δεν έχω δεί σε διασωληνωμένο ξύπνιο να χρησιμοποιείται πέννα και χαρτί να σου πω την αλήθεια

**Σε ποια φάση το έχεις δει;**

Σε τραχειοστομημένο άρρωστο, σε άρρωστο ο οποίος έχει τραχειοστομία με ή χωρίς τον αναπνευστήρα σε αυτή την φάση, γιατί στον ενδοτραχειακό σωλήνα ή θα τον βγάλει ή θα συνεχίσεις με τον ενδοτραχειακό και θα ξεκινήσεις πάλι καταστολές δεν έχεις χρόνο να τον αφήσεις ξύπνιο με τον σωλήνα

**Με τον σωλήνα έχεις δει, είπες μου ότι είχατε χρησιμοποιήσει τον πίνακα σε μια ασθενή με κατά πλάκα μου είπες ,δεν θυμούμαι**

Ναι ναι

**Ξανά χρησιμοποιήσετε τον πίνακα σε ασθενείς ο οποίος δεν είχε νευρολογικό πρόβλημα και ήταν διασωληνωμένος ξύπνιος**

Όχι

**Ταμπλετ ή ότι είπες μου όχι, δηλαδή είναι παραπάνω λεκτικά και με τις κινήσεις από ότι αντιλήφθηκα**

Ναι

**Οκ, εμμ γενικά αν σου δίνετουν η ευκαιρία να εκπαιδευτείς σε θέματα επικοινωνίας θα ήθελες να το κάνεις ή όχι; Με τους διασωληνομένους ασθενείς**

Με τους διασωληνομένους ξύπνιους ασθενείς, δηλαδή ας πούμε..

**Όχι απαραίτητα, και για διασωληνομένους ξύπνιους και για διασωληνομένους ή τραχειοστομημένους τέλος πάντων ασθενείς, ή για ασθενείς της μονάδας..**

Ναι είναι ενδιαφέρον γιατί οχι;

**Ως Georgia τι πιστεύεις ότι θα σε βοήθαν να αφομοιώσεις καλύτερα τις πληροφορίες, πιστεύεις ότι θα σε βοηθούσε καλύτερα να ήσουν σε μια τάξη και να κάνετε slide show, θα σε βοηθούσε περισσότερο η τηλεπικοινωνία θα σε βοηθούσε να ήσουν κοντά στον ασθενή να κάνετε τα μαθήματα, θα σε βοηθούσε να κάνετε συνδυασμό;**

Γενικά να είναι έτσι πιο πολύ διαδραστικό, δηλαδή θα ήθελα να είχα τον ασθενή δίπλα μου και ν τα έκανα εκείνη την στιγμή μπροστά του από το να ήμουν σε μια τάξη ή ακόμα και τηλεπικοινωνία , θεωρώ ότι αυτό το κομμάτι δεν είναι ένα κομμάτι θεωρητικό το οποίο θα κάτσω μπροστά από ένα κομπιούτερ να κάνω 5 πράγματα, δηλαδή πρέπει να το πιάσεις λίγο στα χέρια σου, κατάλαβες τι εννοώ, δηλαδή πρέπει να είσαι κοντά στον ασθενή για να το κάνεις

**Μάλιστα, σε γενικές γραμμές αυτά ήθελα να σε ρωτήσω Georgia μου , σε ευχαριστώ πάρα πολύ, θέλω αν σου ήρθε κάτι στο μυαλό κάποια ξεχωριστή εμπειρία εχτός από την κοπέλα με την κατά πλάκα έχεις κάτι άλλο να μοιραστείς μαζί μου..**

Δεν ξέρω βέβαια , ένα συμβαίνει βέβαια, καμιά φορά παίρνω και το συναίσθημα της άρνησης από κάποιους ασθενείς που είναι στην φάση που είναι διασωληνωμένοι ξύπνιοι ας πούμε που σε κοιτάνε με ένα βλέμμα σαν και σε μισούν και το νιώθεις αυτό το πράγμα εκείνη την στιγμή, ενώ τους λες θέλω να σε βοηθήσω και όλα αυτά ,μπορεί να είναι και επιθετικοί και αντιδραστικοί, μπορεί να μην θέλουν να σε βλέπουν και τέτοια , άλλο περιστατικό δεν μπορώ να σκεφτώ αυτή την στιγμή η αλήθεια είναι, δεν μου έρχεται κάτι, δεν ξέρω

**Δεν πειράζει δεν πειράζει**

Είναι πολλά που γίνονται δεν μου έκανε κάτι, έτσι, ντάξει η συγκεκριμένη κοπέλα ίσως είναι που ήταν αρχικό στάδιο της καριέρας μου και μου έκανε εντύπωση η επικοινωνία με τα μάτια ας πούμε

**Μάλιστα, νομίζω σε ρώτησα και για τα κοβιτ ,εε**

Στα κόβιτ δυστυχώς μέχρι στιγμής έχουμε 3 άτομα , εντάξει τα οποία έχουνε φύγει από την εντατική έχουνε πάει σε θάλαμο , εε οι υπόλοιποι είναι βαριά, έχουμε ένα περιστατικό βέβαια το οποίο το ξυπνήσαν σήμερα και είχε νοητική στέρηση η κοπέλα αλλά δεν ξέρω , δεν ήμουν κοντά της για να δω , αυτό είναι το πρόβλημα , τα άτομα με νοητική στέρηση δεν ξέρεις σε τι βαθμό επικοινωνούν όταν τους ξυπνήσεις και σε τι βαθμό αντιλαμβάνονται , κατάλαβες

**Ναι , είναι και αυτό μεγάλο πρόβλημα η αλήθεια, εε ναι είναι σημαντικό και ειδικά αμάν δεν ξέρεις τον ασθενή που πριν πως ήταν κλπ να ξέρεις το υπόβαθρο τους φαντάζουμε είναι ακόμα πιο δύσκολο στην μονάδα, μάλιστα ευχαριστώ Georgia μου πάρα πολύ για τις πληροφορίες, μου είπες πάρα πολλά πράγματα και θα χαρώ και καμιά φορά να σε γνωρίσω από κοντά, σε γνώρισα από κοντά μάλλον, να συνεργαστούμε από κοντά, αν έχει κάποιους συναδέλφους που θα ήθελαν να συμμετάσχουν…..**

No 11

Bold: researcher

Unbold-normal letters: Aristotelis

**Leonidas όπως σου είχα πει, κάνω μια έρευνα για διασωληνομένους ξύπνιους ασθενείς, βασικά εκείνο που διερευνώ εγώ είναι τις εμπειρίες των νοσηλευτών που εργάζονται με αυτούς τους ασθενείς στις ΜΕΘ, να ξέρεις ότι πούμε μένει μεταξύ μας και πρόσβαση στις πληροφορίες έχω εγω και οι καθηγήτριες μου, όταν δημοσιοποιηθούν τα στοιχεία τέλος πάντων δεν θα μπει κάπου όνομα, δεν θα ξέρουν ποιος μίλησε κλπ, ότι μου πεις να ξέρεις δεν έχει σωστό και λάθος, επίσης όσα πιο πολλά μου πεις τόσο το καλύτερο για εμένα, εννοώ μεν σκέφτεσαι ότι θα μου πεις κάτι το οποίο να πεις α εν βλακεία που είπα τώρα ή ξερω εγώ κάτι άλλο, εννοώ οσα πιο πολλά μου πεις τόσο το καλύτερο, να αρχίσω με δημογραφικές ερωτήσεις, πρώτα θα ήθελα να σε ρωτήσω πόσο χρονών είσαι;**

28

**Πόσα χρόνια εργάζεσαι ως νοσηλευτής;**

Από το 2016

**4 χρόνια δηλαδή, σωστά;**

Πέντε

**Πέντε ναι, συγνώμη, πόσα χρόνια εργάζεσαι σε ΜΕΘ;**

Που τον Δεκέμβριο του 2018, δυο χρόνια και κάτι μήνες

**Που έχεις σπουδάσει;**

Στο πανεπιστήμιο Λευκωσίας

**‘Έχεις κάμει κάποιο μεταπτυχιακό ή κάποια περετέρω εκπαίδευση;**

Eεεε παρακολούθησα 4 σεμινάρια για τις πληγές, τις κατακλίσεις και πήγα erasmous στην Λιθουανία για τα παρηγορητική φροντίδα και φροντίδα στα άτομα που βρίσκονται στα τελευταία στάδια ζωής

**Πολύ ενδιαφέρον αυτό δεν το γνώριζα, να σε ρωτήσω το πρώτο πράμα, να σου θυμίσω ότι , θέλω να έχεις στο νου σου αφορούν διασωληνομένους και ξύπνιους ασθενείς, όχι τραχειστομημένους**

Μάλιστα

**Οκ ,όταν έρθω στην μονάδα σου σε ποιες περιπτώσεις θα έβρω ένα διασωληνωμένο και ξύπνιο ασθενή**

Σε ποιες περιπτώσεις;

**Ναι**

Στις περιπτώσεις που θα ,μετά το χειρουργείο που θα πρέπει να ξυπνήσουμε τον ασθενή για αποσωλήνωση, εε αν πληροί τα κριτήρια τέλος πάντων να τον ξυπνήσουμε και στους ασθενείς που προσπαθούμε σιγά σιγά να τους κάνουμε wean off από τον αναπνευστήρα

**Μάλιστα οκ, υπάρχει οποιαδήποτε άλλη περίπτωση εχτός από το weaning που μπορούμε να βρούμε ένα διασωληνομένο και ξύπνιο ασθενή; Αν μπορείς να θυμηθείς**

Η τελευταία περίπτωση που θυμάμε είναι ασθενής η οποία είχε ισχαιμικό εγκεφαλικό όπου είχε χαμηλή κλίμακα γλασκώβης που είχαμε διακόψει που τα χθές την , παράμενε διασωληνωμένη αλλά χωρίς καταστολή που τα χθες και περιμένουμε να ξυπνήσει

**Δηλαδή είναι διασωληνωμένοι χωρίς να έχει οποιαδήποτε καταστολή και εν κοιμισμένη;**

Είναι διασωληνωμένοι χωρίς καμία καταστολή

**Ο λόγος που περιμένετε να ξυπνήσει δηλαδή είναι ότι η νευρολογική της κατάσταση δεν είναι καλή, τούτο εννοείτε:;**

Μάλιστα

**Μάλιστα οκ, πάνω κάτω περίπου πόση ώρα διαρκούν τα χειρουργεία την ώρα που τους κάνετε weaning και είναι σε αυτή την φάση , δηλαδή διασωληνωμένοι και ξύπνιοι;**

Οι πλείστες περιπτώσεις που έχουμε είναι χειρουργεία καρδίας, αν πχ το bental εε παραμένει διασωληνομ.. σε καταστολή μέχρι σχεδόν όλη την νύχτα, αν είναι νύχτα που θα βγει από το χειρουργείο ή μέχρι να πληροί τα κριτήρια, δηλαδή από 5-12 ώρες

**Δηλαδή από 5-12 ώρες είναι ξύπνιος με τον σωλήνα;**

Ξύπνιος με τον σωλήνα, συγνώμη, όχι, συγνώμη, κατάλαβα λάθος, εε ξαναπές μου την ερώτηση

**Περίπου πόση ώρα διαρκεί αυτή η κατάσταση να έχετε οφφ τις καταστολές και να είναι ξύπνιος ο ασθενής με τον σωλήνα;**

Η κάθε περίπτωση πιστεύω είναι διαφορετική για τον λόγο του ότι εε ο κάθε άρρωστος εν διαφορετικός και δεν έχει χρονικό πλαίσιο στο οποίο θα μπορέσουμε να πληροί τα κριτήρια να γίνει αποσωλήνωση, δηλαδή, είναι διάφοροι παράγοντες που μπορεί να παίξουν ρόλο, αν αναπνευστικά δεν είναι έτοιμος εννοείται δεν μπορώ να τον αποσωληνωσω

**Οκ, μάλιστα, αρα δηλαδή εσύ που τούτα που είπαμε τώρα , οι πλείστες περιπτώσεις αφορούν το weaning Που θα βρεις ένα διασωληνωμένο ξύπνιο ασθενή εχτός το περιστατικό που είπαμε με την κυρια που είναι διασωληνομένη και ξύπνια αλλά δεν ανταποκρίνεται γιατί νευρολογικά δεν είναι καλή**

Μάλιστα

**Μια γενική ερώτηση τωρά, πως είναι να εργάζεσαι με αυτους τους ασθενείς**

Κοίταξε πιστεύω είναι αρκετά δύσκολο κομμάτι, όσο στο ψυχολογικό όσο και στο σωματικά παράγοντα γιατί οι πλείστοι άρρωστοι αντιμάχουντε την ώρα που έχουν τον σωλήνα στον λαιμό τους, λογικό, επίσης η ευθύνη είναι αρκετά μεγάλη γιατί μπορεί να πάει οτιδήποτε στραβά, εχτός του ότι την ευθύνη και την σοβαρότητα, μπορώ να σου πω ότι νιώθω ότι πρέπει να είμαι αρκετά συγκεντρωμένος ας πούμε για να βλέπω τι γίνεται, εε και αν εκάναμε περισσότερο μαθήματα, τάχα είναι και η έλλειψη γνώσης που μπορεί να παίξει ρόλο, γιατί δεν είναι όλοι που ξέρουμε αρκετά καλά να διαχειριζόμαστε τον αναπνευστήρα έτσι ώστε να βοηθήσουμε τους ασθενείς να είναι πιο ομαλή η αποσωλήνωση και πιο κατάλαβες τι θέλω να πω ενεν

**Ναι βεβαίως**

Αλλα γενικά , είναι δύσκολο κομμάτι ειδικά εκείνη την ώρα πχ γιατί ξέρεις υπάρχουν και οι ασθενείς οι οποίοι μπορεί να ξυπνήσουν και αν είναι συγχυσμένοι και μπορεί να τραυματιστούν, τούτα μου έρχοντια τώρα

**Εντάξει οκ, ήθελα να σε ρωτήσω, συγκεκριμένα πως καταφέρνεις να επικοινωνήσεις με αυτούς τους ασθενείς, τους διασωληνομένους και ξύπνιους, πως επικοινωνείς;**

Από εκείνα που μάθαμε στο πανεπιστήμιο, πρέπει να εκτιμήσεις την νευρολογική του εικόνα με βάση ερωτήσεις που πρέπει να είναι ναι ή όχι και πρέπει να σου νέψει με το κεφάλι ή τα μάτια, ρωτάς τον αν σε ακοευι αν μπορεί να ανοιξει τα μάτια του, μπορεί να κουνήσει κεφάλι δεξια αριστε΄ρα μπροστά και πίσω, αν μπορέσει να εκτελέσει οδηγίες κινώντας τα ακρα του, μπορώ να επικοινωνήσω ας πούμε, ενημερώνω τους ότι είμαι κοντα τους και ότι θα τελείωσει αυτή η διαδικασία οσο πιο γρήγορα γίνεται φτάνει να συνεργαστούμε, τούτα

**Πολύ ωραία, όταν οι ασθενείς ξυπνήσουν και είναι σε αυτή την φάση τι πιστεύεις ότι θέλουν να σου πουν;**

Πιστεύω ότι νιώθουν αναγούλα και την δυσφορία ότι έχουν ένα ξένο σώμα στο λαιμό τους, άγχος, γιατί μόλις σε ξυπνήσουν προσπαθούν να αναπνεύσουν μόνοι τους και έχει κάτι άλλο που τους φυσά αέρα στους πνεύμονες και νιώθουν ας πούμε κάποιο άγχος στο τι συμβαίνει, που είναι λογικό γιατί έχουν και μια απώλεια του χρόνου και το τί γίνεται λόγω της καταστολής που έπαιρναν πριν και σίγουρα ότι θέλουν να τους δώσουμε περισσότε.. κάποια βοήθεια στο να μεν νιώθουν τόσο άβολα σε εκείνο το κομμάτι της φάσης και να προσπαθήσουμε τάχα να , εγώ θα προσπαθούσα να είμαι πιο χαλαρός, αν υπήρχε κάτι άλλο να με βοηθήσει ας πούμε για να μπορέσω να συνεχίσω για εκείνο το χρονικό διάστημα τούτη την διαδικασία, κατάλαβες;

**Οκ**

Εννοώ κάτι που θα με βοηθούσε, κάποια ελαφριά χαλάρωση στο να μην νιώθω τόσο πολύ έντονα τον σωλήνα ας πούμε ή τέλος πάντων κατάλαβες το αίσθημα ότι κάτι έχει στον λαιμό μου να μην είναι τόσο άβολο

**Πως προσπαθούν να σου πουν τούτοι οι ασθενείς, δηλαδή τι κάνουν, τι προσπαθούν να σου που οι ίδιοι**

Συνήθως από ότι είδα με την εμπειρία μου, εκδηλώνουν την δυσφορία τους στο ότι δεν αναπνέουν και έχουν συχνά αναγούλα, άλλοι έδειξαν μου ότι δυσκολεύονται τόσο πολλά να αναπνεύσουν που έτρεχαν τα μάτια τους δάκρυα, εε μου έδειχναν με τα χέρια ότι δεν μπορώ άλλο, φκαρμου τον δεν μπορώ άλλο είχε άλλο που ήταν τόσο έντονα τα επεισόδια αναγούλα που έδειχναν μου ότι ήθελαν να κάμουν εμετό εε άλλοι έδειχναν μου ότι, ήταν πολλά πιο ανήσυχοι και προσπαθούσαν οι ίδιοι να τραβήξουν τον σωλήνα γιατί δεν μπορούσαν το αίσθημα ότι είχαν κάτι ξένο στον λαιμό τους, εε τούτα δεν έτυχε άλλο, α και είχε άλλους που μου έδειξε ότι δεν μπορούσε να αναπνέει ας πούμε την ώρα που ξύπνησε, δεν ήταν έτοιμος να αποσωληνωθεί και αναγκαστικά έπρεπε να τον κοιμίσουμε και να το δοκιμάσουμε αργότερα ούτε έτυχε μου να μην μπορεί να συνεργαστεί με τον αναπνευστήρα

**Μάλιστα ,δηλαδή τούτα όλα αφορούν την αναπνοή των ασθενών και ότι δεν μπορούν να αναπνεύσουν που μόνοι τους και ότι δυσφορούν που τον σωλήνα, υπήρχε οποιαδήποτε άλλη περίπτωση που ο ασθενής ήταν ήρεμος συνεργαζόταν με τον σωλήνα ,ανάπνεε και ήθελε να σου πει κάτι περετέρω εκτός από αυτά;**

Το πρώτο μου extubation που έκανα που χειρουργείο καρδίας ο ασθενής μου ήταν αρκετά ήρεμος στην αρχή αλλά έδειχνε μου εκείνη την ώρα ανυπομονησία, ότι αντέχω αλλά οι για πολλά , θέλω να τον βγάλω να τελειώνουμε ή είχε μου ο άλλος που παρόλο που του είχα δώσει αντιεμετικό για να τον βοηθήσω να μην έχει αναγούλα , είχε πιο έντονο το αίσθημα που του το είχα δώσει και έδειχνε μου ότι ανυπομονούσε να βγει και ταχυπνοούσε πάρα πολλά , εε τούτα θυμούμαι, συνήθως εν το αίσθημα της δυσφορίας και ότι δυσκολεύονται να αναπνεύσουν, είναι το πιο σημαντικό σε αυτούς τους ασθενείς πιστεύω έτσι;

**Ντάξει εσύ ξέρεις καλύτερα, εγώ δεν δουλεύω σε μονάδες, εσύ σίγουρα έχεις περισσότερη εμπειρία από εμένα, μάλιστα οκ εεε ξανά επιστρέφω στον τρόπο επικοινωνίας σου με τους διασωληνομένους και ξύπνιους ασθενείς, εε τι εμπόδια πιστεύεις ότι βρίσκεις στο να καταφέρεις στο να επικοινωνήσεις με αυτούς τους ασθενείς;**

Κοίταξε η λεκτική επικοινωνία με αυτούς τους ασθενείς επειδή δεν μπορούν να σου μιλήσουν και να σου εκφράσουν το τι νιώθουν και το πρώτο εμπόδιο που μπορώ να σκεφτώ, εχτός του ότι είναι και η σωματική δυσφορία που νιώθουν μετά το χειρουργέιο ότι πονούν και ότι δυσκολεύονται να αναπνεύσουν επίσης μπορώ να σου ότι εε η σύγχυση που νιώθουν εκείνη την στιγμή μόλις ξυπνήσουν στο ότι ακόμα ναι είναι ξύπνιοι αλλά δεν μπορούν να επικοινωνήσουν 100% μαζί σου και εσύ προσπαθείς ας πούμε με διάφορους τρόπους πχ να τους καθησυχάζεις, το άγγιγμα η χροιά της φωνής σου να είναι καθησυχαστική έτσι ώστε να μπορέσουν να νιώσουν ότι είναι σε ασφαλές περιβάλλον τούτο εε είναι κάλο να γίνεται πάντα αλλά δεν μπορείς να το κάνεις σε όλες τις περιπτώσεις γιατί έχει ασθενείς οι οποίοι εν αρκετά agitate και είναι πάρα πολλά restless σε έτσι καταστάσεις και δεν μπορείς να το κάνεις πάντα τούτο ενεν;

**Χμμ οκ, τι άλλο πιστεύεις ότι εμποδίζει σε να καταφέρεις να επικοινωνήσεις με αυτούς τους ασθενείς, τι πρόσεξες ως Leonidasς;**

Ως Leonidasς τι πρόσεξα, κοίταξε με εκείνα που ξέρω ας πούμε μπορεί να κάμεις ένα , να δημιουργήσεις ένα περιβάλλον το οποίο στους αρρώστους σου το να μην είναι τόσο στρεσογόνο, κατάλαβες;

**Δηλαδή εννοείς μου ότι το στρεςς για τους ασθενείς είναι ένα εμπόδιο προς εσένα για να μιλήσετε αν αντιλαμβάνομαι σωστά**

Μάλιστα

**Οκ όταν οι ασθενείς προσπαθούν να σου πουν κάτι και δεν τα καταφέρνουν τι πιστεύεις ότι νιώθουν;**

Φόβο, ταχα ότι προσπαθώ να σου πω κάτι και δεν μπορώ να βρω τον τρόπο πως να με καταλάβεις, αγωνία ,μπορεί να μην μου εξηγήσεις το τι γίνεται και δεν ξέρω το τι γίνεται και μπορεί να μην είμαι σίγουρος για το τι γίνεται και το πως είμαι, η έλλειψη της ενημέρωσης ς πούμε, πήγα έκανα χειρουργείο, είμαι καλά γιατί έχω αυτό στον λαιμό μου, έγινε κάτι λάθος; Εε ότι θέλουν να μου πουν ότι πονούν και δεν μπορούν να μου δείξουν, πως μπορώ να του δείξω ότι πονώ και για να το καταλάβει και ν με προσέχει

**Εσύ όταν προσπαθείς να επικοινωνήσεις με τον ασθενή και δεν καταφέρνεις να καταλάβεις τι θέλει αυτός ο ασθενής τι θα κάνεις; Αν θα κάνεις κάτι δεν είναι απαραίτητο να κάνεις κάτι**

Εντάξει είναι πάντα απαραίτητο να κάνεις κάτι, εγώ σαν Leonidasς έρχομαι και αυτό που κάνω, όσες φορές μου έτυχε να ξυπνήσω κάποιον και να προσπαθήσω να κάμω extubation λέω του ενημερώνω τον ποιος είμαι που βρίσκεται έτσι ώστε να καταλάβει ότι εε, να έχει το αίσθημα του τόπου χρόνου, που είναι που βρίσκεται , ενημερώνω τους για το αν τέλειωσε το χειρουργείο και ότι πήγαν όλα καλά έτσι προσπαθώ να τους καθησυχάσω, μιλώ τους δυνατά και σιγά ώστε, αργά εννοώ για τους δείξω αίσθημα εμπιστοσύνης και ασφάλειας και καθησυχάζω τους ότι όλα θα πάνε καλά και είμαι δίπλα τους, αυτά τα πέντε βήματα που ακολουθώ ας πούμε πιστεύω ότι σαν Leonidasς ο οποιοσδήποτε είναι εκεί στο κρεβάτι του πόνου θα τον βοηθήσει και ψυχολογικά ,ψυχολογικά κυρίως στο να καθησυχαστεί και να μειωθεί η αγωνία του και κατάλαβες

**Ναι ακούω σε , υπήρξε κάποια περίπτωση που να μην καταφέρεις να επικοινωνήσεις με ασθενή που να μην καταλάβεις τι σου ,τι θέλει να σου πει**

Εεε να σου πω την αλήθεια έτυχε μου μια φορά σε ένα ασθενή που έκανε θυμεκτομή και στην προσπάθεια, σταμάτησα σιγά σιγά την καταστολή, παρατήρησα στον αναπνευστήρα ότι έκανε περισσότερους όγκους και έπαιζαν τα alarm και τον γύρισα σε spontaneous mode για να αναπνέει μόνος του και έδειχνε μου ότι , ξεκίνησε και ίδρωνε διότι δεν μπορούσε να κινηθεί και να δει τι συμβαίνει εε έκαμα τούτα τα βήματα, άρχιζε και ένευε μου με το κεφάλι του ότι δεν είναι καλά και γυρνούσε το κεφάλι του δεξιά αριστερά έτσι κατάλαβα εγώ φυσικά και άρχισα και τον ρωτούσα αν πονάει και ένευε μου με το κεφάλι του ναι έε έδειξε μου ότι θέλει να κάμει αναγούλα αλλά στην δεύτερη με Τρίτη φορά κατάλαβα ότι ήθελε να κάμει εμετό εεε τούτο μου έτυχε να σου πω την αλήθεια

**Αρα σε περίπτωση που δεν καταφέρνεις να επικοινωνήσεις με τον ασθενή προσπαθείς που μόνος σου να τον ξανά ρωτήσεις ερωτήσεις για παράδειγμα;**

Νι συνέχεια,

**είπες μου πως νιώθουν οι ασθενείς όταν δεν καταφέρνουν να επικοινωνήσουν μαζί σου, εσύ στην συγκεκριμένη περίπτωση αφού τούτη σου έτυχε, πως σε έκανε να νιώσεις που δεν καταλάβαινες τι ήθελε, που δεν καταλάβαινες να επικοινωνήσεις με αυτό τον ασθενή; αν σε έκανε να νιώθεις κάτι**

Επειδή ήταν στις αρχές που δεν είχα και πάρα πολλή εμπειρία, νομίζω ήταν το 4^ο^ μου extubation και δεν ήμουν αρκετά σίγουρος, αγχώθηκα λίγο, συμβαίνει κάτι τώρα, ενιωθα και λίγο αβέβαιος ,εε και ολόισια πήγα και βρήκα την μέντορα μου και ήρθε και βοήθησε με και εξήγησε μου τι γίνεται,τούτο το πράμα ας πούμε , δεν είχα αρκετή εμπειρία ας πούμε να μην μπορώ να εκτιμήσω την κατάσταση τι συμβαίνει ; ένιωθα λίγος άγχος και αβεβαιότητα, ξέρεις έκαμα κάτι λάθος κι νιώθει τώρα έτσι ο ασθενής, ε τι γίνεται τωρά

**Ναι οκ, αρα είναι ένα βήμα και αυτό να ζητήσεις την βοήθεια κάποιου, είναι ένας τρόπος για να επιλύσεις το θέμα, τούτο ήθελα να μάθω εγώ αν κάμνεις κάτι άλλο αν χρησιμοποιείς οποιαδήποτε στρατηγική στο να μπορέσεις να επικοινωνήσεις με τους ασθενείς**

Ναι

**Μάλιστα, εε που τούτα που σε ρώτησα ξανά επιστρέφω πίσω, σκέφτηκες κάτι άλλο το οποίο σε βοηθά να επικοινωνήσεις με τους ασθενείς; Οι σε βοηθά, συγνώμη να αλλάξω την ερώτηση..**

Είχε φορά που χρησιμοποιήσαμε εικόνες θυμούμαι που ένας άρρωστος δεν ήταν πολλά αναπνευστικά έτοιμος να βγει και χρειαζόταν να παραμείνει στον αναπνευστήρα και το τι έκανα πήγα και βρήκα εικόνες που μπορούσε ας πούμε να κοιμηθεί ήταν ξύπνιος αλλά αναπνευστικά έπαιρνε αρκετό οξυγόνο εε αλλά ήταν νευρολογικά ξύπνιος καταλάβαινε, εκτελούσε οδηγίες κλπ και που τον είχα ρωτήσει αν πονάει δεν μπορούσε να μου εκφράσει , εκτός που το πρόσωπο του να το αξιολογήσω και έπιασα την κλίμακα του πόνου που δείχνει το πρόσωπο, εκείνη που δείχνει στο πρωτόκολλο, και είπα του ας πούμε πως νιώθει αυτή την στιγμή για να ξέρω να το βαθμολογήσω για να ξέρω να του δώσω κάποιο παυσίπονο και ν του δώσω κάποια χαλάρωση ας πούμε, ξεκινήσαμε ultiva (remifentanil) εκείνη την μέρα και ένιωθε και καλύτερα, επειδή ήταν και το θέμα του πόνου πιστεύω και γι αυτό μπορούσε να αναπνέει και καλά και μετά που του δείξαμε και ξεκινήσαμε Ultiva εε τον βοήθησε αρκετά και αναπνευστικά ηταν καλύτερος και μετά από δυο τρεις ώρες έγινε extubate νομίζω εκείνη την μέρα, ναι

**Πολλά ωραία, έχετε δηλαδή κλίμακες στο τμήμα σας το οποίο σας βοηθά στην επικοινωνία, όχι κλίμακες μόνο, έχετε οποιαδήποτε άλλα βοηθητικά μέσα επικοινωνίας, όπως για παράδειγμα πίνακες με εικόνες ή πίνακες με γράμματα ή ταμπλετ που βοηθούν στην επικοινωνία, που να έχει κάποιο πρόγραμμα ή το πιο απλό πέννα και χαρτί , χρησιμοποιείται τα τούτα, έχετε για να τα χρησιμοποιήσετε κατά ακρίβεια;**

Εχουμε αρκετές ας πούμε , αρκετά πως να το πω μέσα επικοινωνίας απλά συνήθως δεν χρειάζονται νομίζω

**Οκ, δυο ερωτήσεις γι αυτό. Πρώτο τι μέσα έχετε ας πούμε τι ακριβώς έχετε μπορείς να μου εξηγήσεις να καταλάβω;**

Εχτός του ότι ενημερώνουμε τον ασθενή κλπ ,έχουμε μέσα για να αναγνωρίσουμε τον πόνο όπως ανάφερα και πριν ,εε δεν θυμούμαι άλλο να σου πω, έχουμε για τον πόνο, ενημερώνουμε τον άρρωστο κι δείχνουμε του ότι είναι στο νοσοκομείο τούτα τα πράματα

**Εγώ μιλώ συγκεκριμένα αν έχετε κάποιους πίνακες που έχουν πάνω εικόνες ή γράμματα ή κάποιο τάμπλετ που να έχει κάποιο πρόγραμμα τούτο που εννοω μέσο επικοινωνίας**

Από ότι ξέρω όχι δεν έχουμε

**Δεν έχετε οκ , χρησιμοποίησες ποτέ κόλλα και πέννα σε κάποιο ασθενή**

Όχι

**Είπες μου ότι δεν χρειάζονται, για πιο λόγο πιστεύεις ότι δεν χρειάζονται**

Ε κοίταξε συνήθως εε τα πλείστα χειρουργεία που παραλαμβάνουμε που τον ασθενή είναι ήδη extubate από το χειρουργείο και στο extubate aς πούμε εκεί μπορεί να χρησιμοποιήσουμε διάφορες κλίμακες ή μέσα επικοινωνίας ώστε ο ασθενής να βοηθηθεί ,εε απλά πιστεύω ότι η έλλειψη γνώσης στο να χρησιμοποιήσουν αυτά τα πράγματα και ότι δεν ξέρουμε πως να τα χρησιμοποιήσουμε και ότι υπάρχουν τούτα τα πράματα και γι αυτό δεν τα χρησιμοποιούμε και πιστεύω ότι είπα δεν χρειάζεται, έχει φορές που μπορεί να χρειάζεται αλλά εε συνήθως με την λεκτική επικοινωνία εν το πιο σημαντικό μέσο επικοινωνίας να βοηθήσεις τον ασθενή εκείνη την δεδομένη στιγμή, να τον ενημερώσεις πλήρες τι συμβαίνει και ξανά και ξανά, γιατί ο ίδιος μπορεί να μην είναι στην θέση τι του δείχνεις κατάλαβες

**Ναι κατάλαβα,**

γ αυτό είπα δεν νομίζω να χρειάζεται αλλά δεν είναι πολλά έτσι ..

**Οκ έχει κάποιο συγκεκριμένο περιστατικό που σου έμεινε στον νου για οποιοδήποτε λόγο μιλώ για αυτή την φάση όταν ήταν διασωληνωμένος και ξύπνιος και προσπαθούσες να επικοινωνήσεις μαζί του που θέλεις να του περιγράψεις**

Το πρώτο μου extubate ο άρρωστος ήταν έτσι , δεν ήθελε ο ίδιος να γίνει διασωλήνωση θυμούμαι επειδή πίστευε ότι ήταν καλά αλλά λόγω του ότι είχε ψηλό διοξείδιο ήταν συγχυσμένος δεν ήταν βέβαιο το τι γίνετουν και αν ήξερε το τι συναίβενε και θυμούμε ότι ήμουν παρών που έγινε η διασωλήνωση και όταν αποσωληνώθηκε που το έκανα εγώ , παρόλο που αναπνευστηκά ηταν καλύτερα εε έδειξε ότι ηταν αρκετά θυμωμένος που κάναμε αυτή την διαδικάσία και είχε χρησιμοποίήσει και βια στο πρώτο μου extubate ήταν αρκετάς βιαίος σε εκεινη την περίπτωση

**Δηλαδή αν κατάλαβα καλά ηταν ενας ασθενής στην μονάδα που δεν ηταν διασωληνομένος και χρειάστηκε κάποια στιγμη να διασωληνωθεί και στην αποσωλήνωση του ηταν άγριος ;**

Μάλιστα

**Οκ στην συγκεκριμένη περίπτωση τότε τι εμπόδια βρήκες στον συγκεκριμένο ασθενή που ηταν στην φάση που ηταν διασωληνομένος κι ξύπνιος ;**

Εε το εμπόδιο που είδα ότι ήθελε συγκεκριμένα άτομα, ηταν συχνός πελάτης να πω και ήθελε συγκεκριμένους νοσηλευτές να είναι εκεί και ήμουν ενας από αυτούς, επείδη νομίζω με συμπαθούσε, ετσι μου έδιχνε και εκείνη την φάση που ηταν οργισμένος μου έδιεξε το αίσθημα της απογοητευσης , ότι τον απογοητευσα πχ και μετά που έκατσα και εξήγησα του γιατι έγινε αυτό και αυτό πάλι ηταν θυμωμένος αλλα με τον εαυτό του ας πουμε και ταυτόχρονα που ηταν η διαδικασία να βγάλουμε τον σωλήνα , ανυπομονούσε να τον βγάλουμε για να μας την πει.. κι ένιωσα λίγο κάπως , τίνεται τωρά, ήμουν σε ένα δίλλημα, καλά σε βοηθήσαμε, εσωσα σου.. δεν σε αφήσαμε να πεθάνεις και ηταν κάπως είναι αυτό το ευχαριστώ, δεν ξέρω αν ηταν σχετικό με την ερώτηση σου

**Οι συζήτηση που κάνουμε, άλλο που ηθελα να σε ρωτήσω, εε στο πανεπιστήμιο έκανες οποιοδήποτε μάθημα για μέσα/τρόπους εποικοινωνίας γενικά ή οσο αφορά τους διασωληνομένους και ξύπνιους αασθενείς**

Κάναμε μαθήματα επικοινωνίας μέσο επικοινωνίας και στο μάθημα της ηθικής και στο μάθημα για την επικοινωνία και νομιζώ κάναμε και στο τελευταιο έτος, 3 μαθήματα που κάναμε αλλα όχι οσο αφορά τους διασωληνομένους

**Αν σου εδίνετουν ποτέ η ευκαιρια οσο αφορά τους διασωληνομένους και ξύπνιους ασθενείς θα ήθελες ή όχι να το κάνεις, σαν μιχαλης τι πιστευεις, με ποιο τρόπο θα προτίμας να κάνεις αυτό το μάθημα, για πχ θα προτίμας να κάνεις τηλεδιάσκεψη, σε ταξη με πίνακα και να εξηγά κάποιος καθηγητής, δίπλα από τον ασθενή , σε διάλεξη, σε διάλεξη και δίπλα από τον ασθενή, με πιο τρόπο πιστευεις θα βοηθούσε**

Να σου πω στο πανεπιστήμιο μας συνήθως κάνουμε τα μαθήματα σε simulation room

**Δηλαδή**

Εε κάνμε ας πουμε πχ για την ένα μάθημα που θυμούμε και εγώ, για την διαβητική κετοξέωση και εδίχναν μας για παράδειγμα πως το αντιμετωπίζουμε έτσι σου έμενε περισσότερο και στο πρακτικό και στο θεωρητικό κομμάτι

**Αρα πιστευεις θα σε βοηθούσε μάθημα και δίπλα από τους ασθενέις για παράδειγμα**

Ναι

**Μαλιστα ευχαριστω…ηθελα να σε ξαναρωτήσω με τους τρόπους επικοινωνιας σου με τους ασθενείς πχ είπες μου ότι κάνεςι τους ερωτήσεις κουνούν το κεφάλι τους τα πόδια τους τα χέρια τους έχεις κάτι άλλο πχ που δεν το είπαμε και θέλεις να μου το πεις**

Όχι δεν νομίζω

**Οσο αφορά τα εμπόδια επικοινωνίας τι πιστευεις ότι εν τα εμποδια που σου εμποδίζουν να επικοινωνήσεις με αυτους τους ασθενείς**

Ειπαμε το αυτό ενεν

**Ναι αλλα λεω μηπως σκέφτηκες κάτι άλλο διαφορετικό**

Οχιι δεν μου έρχεται κάτι τωρά έχεις κατι εσυ να μου πεις;

**Oοχι εγω θέλω να ακούω τους άλλους δεν θέλω να παρεμβαίνω…**

Συνέντευξη Νο 12

Bold: researcher

Unbold-normal letters: Alexandros

**Επεξήγηση διαδικασίας και εμπιστευτικότητας κλπ. κλπ..**

**Η πρώτη ερώτηση που θα ήθελα να σου κάνω είναι σε ποιες περιπτώσεις στην δική σας Μονάδα θα μπω και θα δω ένα διασωληνωμένο ξύπνιο ασθενή;**

Κατά την διάρκεια της προετοιμασίας για να αντέχει ο άρρωστος για απογαλακτισμό

**Οκ, υπάρχει οποιαδήποτε άλλη περίπτωση;**

Κακή, κακή εμμ τιτλοποίηση των φαρμάκων, των κατασταλτικών δηλαδή ο άρρωστος δεν είναι σωστά κοιμισμένος

**Μάλιστα, γενικά πως είναι να εργάζεσαι με τέτοιου είδους ασθενείς;**

Εντάξει σίγουρα είναι κάτι στρεσογόνο αλλά παράλληλα είναι και μια πρόκλησή, να έχεις ένα άρρωστο να εξαρτάτε αποκλειστικά από εσένα, την ολιστική φροντίδα.. είτε και θεραπευτικά είτε και στο θέμα φροντίδα.

**Πως καταφέρνεις να επικοινωνήσεις με τούτους τους ασθενείς;**

Εε ..

**Πες μου από τα πιο απλά, δηλαδή εγώ τώρα σκέφτου ότι είμαι ο ασθενής και θα έρθεις να μου μιλήσεις και να επικοινωνήσουμε, πες μου που τα πιο απλά ας πούμε, μεν πάρεις κάτι δεδομένο.**

Καταρχάς θεωρώ το άγγιγμα επικοινωνία , εε θεωρώ το πολλά σημαντικό, δηλαδή το αίσθημα το ένα χέρι αγγίζει πάνω στον άρρωστο και η κουβέντα, ο καθησυχασμός, το τι αντιλαμβάνομαι τούτο που νιώθεις τώρα ότι εν δύσκολο κομμάτι, ότι νιώθεις τον σωλήνα μέσα στο στόμα σου, κάνε υπομονή προσπαθούμε να δούμε, δούλεψε και εσύ μαζί μας , κάνε , να είσαι ήσυχη, βασικά ένας καθησυχασμός και ότι αντιλαμβανόμαστε τούτο που περνά και ότι το κάνουμε με όλους τους αρρώστους σε αυτή την φάση , δηλαδή εν για το καλό που το κάνουμε, τούτο το στρες που περνάς είναι για να σε δοκιμάσουμε ότι μπορείς, ενθάρρυνση στην ουσία

**Αμαν ο ασθενής προσπαθεί να σου πει κάτι τι πως προσπαθείς να το αντιληφθείς εσύ, τι τον ρωτάς τι κάνεις**

Καταρχάς θα αποφύγω να του μιλήσω σε θέμα ερωτήσεων γιατί αντιλαμβάνομαι ότι δεν μπορεί να μου απαντήσει άρα και εκείνο θα τον στρεσάρει, πριν να τον ρωτήσω κάτι ή να του πω κάτι θα του πω πρώτα ότι , σήκωσε μου τα φρύδια σου ή κλείσε μου τα μάτια σου ή νεψε μου με το κεφάλι σου σαν απάντηση γιατί χωρίς να του το πω τούτο μπορεί που θα τον ρωτήσω και ο ίδιος να προσπαθήσει να μου μιλήσει και στην προσπάθεια που θα κάνει και να καταλάβει ότι δεν μπορεί τούτο θα τον στρεσάρει ακόμα περισσότερο.

**Χμμ οκ, εμμ τι πιστεύεις ότι θέλουν να σου πουν τούτοι οι ασθενείς την ώρα που προσπαθούν να επικοινωνήσουν, μιλώ πάντα για τους διασωληνομένους ξύπνιους ασθενείς, επειδή η μονάδα εν πολλά περιστατικά διάφορα περιστατικά, γι’ αυτό σου λέω**

Εεε βασικά νομίζω εν ανησυχία που έχουν, δηλαδή μετά που βγαίνουν από εκείνο τον φαύλο κύκλο που ήταν , ως βαριά πάσχοντες, μια ανησυχία ή ακόμα μπορεί να είναι και σε ντελίριουμ ο άρρωστος , σαν ας πούμε να θέλει μια θεραπεία ούτως ώστε να προετοιμαστεί για να βγει από εκείνη την κατάσταση, να είναι ξύπνιος αλλά διασωληνωμένος, αλλά νομίζω εν όλα γύρω από ανησυχία.. ήταν συζήτηση αλλά ως τώρα ρωτάς με και απαντώ σου

**Δεν πειράζει, εγώ τούτο θέλω, εγώ δεν θέλω να μιλώ πολλά εσύ θέλω να μιλάς πολλά ..**

Άρα δεν είναι συζήτηση είναι συνέντευξη

**Εε ναι ας το πούμε συνέντευξη, ε που λες, άλλο που ήθελα να σε ρωτήσω, να μην το πάρω ως δεδομένο, ότι τυχαίνει κάποιες φορές να προσπαθήσεις να επικοινωνήσεις με τούτους τους ασθενείς αλλά να μην καταλαβαίνεις τι σου λένε, δηλαδή πάντα καταλαβαίνεις τι σου λένε;**

Όχι

**Υπάρχει κάτι που θα κάνεις εκείνη την στιγμή ως στρατηγική ας το πω για να προσπαθήσεις ακόμα μια φορά τέλος πάντων να καταλάβεις τι θέλουν να πουν;**

Εεε

**Τι θα έκανες, αν θα έκανες κάτι**

Εντάξει εκείνο που εφάρμοζα, εφαρμόζω είναι μονολεκτικές απαντήσεις με το ναι και το όχι και ο καθησυχασμός, δηλαδή δεν αναμένω ότι θα μπω σε μια συζήτηση με ένα ξύπνιο διασωληνωμένο ή να προσπαθώ να αντιληφθώ τον πόνο του στο να μου τον περιγράψει κάπως για να καταλάβω πόσος είναι , δηλαδή θα τον ρωτήσω, πονείς; Πονεις πολλά; Πονείς λίγο; δεν αντέχεις τούτη την κατάσταση, για να αντέχεις .. δεν θα τον ρωτήσω κάτι σίγουρα που θα πρέπει να μου περιγράψει

**Άρα χρησιμοποι**εί**ς κλειστού τύπου ερωτήσεις για να τον ευκολύνεις και εκείνο αν αντιλαμβάνομαι σωστά**

Ναι και για να μην τον ανησυχήσω παραπάνω , να προσπαθεί να μου πει κάτι και να μην μπορεί

**Εεεμμ τι πιστεύεις ότι είναι τα εμπόδια ώστε να καταφέρεις να επικοινωνήσεις με αυτούς τους ασθενείς, τι εμπόδια βρίσκεις; Γενικά τα πάντα, τι μπορείς να σκεφτείς;**

Δεν έχουμε την τεχνογνωσία , δεν έχουμε τα εργαλεία, βασικά ανεπαρκείς γνώσεις.. δηλαδή αν μου κάνεις την ερώτηση πιστεύεις ότι, ένα σου πω εν ξέρω , δεν εδιδακτηκά κάτι, δηλαδή δώσαν μου ένα εργαλείο και να μου το παρουσιάσουν και να μου πουν ότι τούτος ο ασθενής σε τούτη την δεδομένη στιγμή μπορείς να δουλέψεις τούτα τα πράματα πάνω του και θα έχει καλό αποτέλεσμα, δηλαδή ότι κάνουμε είναι εμπειρικά που το κάνουμε.

**Πιστεύεις ότι μόνο οπι γνώσεις είναι το εμπόδιο σου να επικοινωνήσεις με αυτούς τους ασθενείς, να σου..**

Οοχιιι, όχι, το ότι ίσως να υπάρχει κάτι παραπάνω που μπορώ να κάνω, αλλά δεν το κάνω γιατί δεν το ξέρω ότι γίνεται αυτό το πράγμα

**Οκ**

Δεν έχω κάποιο εργαλείο

**Να σου δώσω ένα παράδειγμα για να καταλάβεις τι εννοώ, γιατί μπορεί να μην το.., ένα πράμα το οποίο είναι αυτονόητο, είναι ο σωλήνας μέσα στο στόμα, εννοώ εε που σε εμποδίζει να επικοινωνήσεις, εχτός από τον σωλήνα στο στόμα, αν βάλεις την θέση σου και μπεις σε εκείνη την εικόνα ότι είσαι εκεί ας πούμε, τι άλλο μπορείς να σκεφτείς ότι εμποδίζει σε να έχεις επικοινωνία με αυτό τον ασθενή, αν έχει κάτι φυσικά εννοώ, απλά προσπαθώ να σε βάλω λίγο στο περιβάλλον για να δεις τι σε εμποδίζει..**

Κοίταξε, να μην αναλωθώ στο θέμα χρόνου κλπ.

**Οι να αναλωθείς να αναλωθείς**

Εεε εντάξει θεωρώ τούτα τα πράματα πλέον για εμάς ότι κατάντησε κουραστικό το να επαναλαμβανόμαστε στις ίδιες κουβέντες, ναι υπάρχει, ναι είναι σαφώς εν κάτι που είναι εμπόδιο ναι, αλλά νομίζω έγινε και η πρώτη μας δικαιολογία αυτό το πράγμα. Το χειρότερο είναι να λες;( *δεν ξεχωρίζω τι λέει)* ότι έχεις ανελλιπής χρόνο ανελλιπής το ένα ανελλιπής το άλλο, γ αυτό προτιμώ να μην το θέτω στα πρωτεύοντα πράγματα αν και είναι, δηλαδή μπορεί να καταγραφεί ότι ένας φραγμός είναι ο χρόνος , δηλαδή εν έχω τον χρόνο να αναλώσω στο επικοινωνιακό κομμάτι με τον άρρωστο, σε άρρωστο που μιλά , πόσο μάλλον σε ένα διασωληνωμένο που πρέπει να κάνεις ολόκληρο πλάνο για να επιτεύξεις τούτο το πράμα.

**Χμμ μάλιστα.. οι θέλω να μου τα λες τούτο που θεωρείς αυτονόητα ή αν τα είπαμε και παραπάνω φορές δεν με πειράζει , εγώ αντίθετα θέλω να μου τα πεις είναι πολύ σημαντικά**

Ε ναι εντάξει δεν ήθελα να πιαστώ σε αυτό το πράμα, ήθελα να το πάρω σε άλλο επίπεδο για εμένα

**Οκκ εεμμ στο τμήμα σας παλιά, στην λάρνακα είχατε καθόλου ασθενείς covid;**

Νοσηλευτήκαν μέχρι να διαγνωστούν και μετά αν εδιαγνωσκουνταν έφευγαν

**Διασωληνωμένοι φαντάζουμε;**

Είχε και μερικούς διασωληνομένους, πολλά λίγοι

**Οκ δεν ξέρω πόση επαφή είχες εσύ με τούτους τους ασθενείς τους διασωληνομένους τους covid,υπήρχε.. είχες καταρχάς επαφή με τούτους τους ασθενείς εσύ η όχι;**

Την δεδομένη στιγμή εγώ όχι

**Όχι εντάξει**

Διότι ήμουν και εγώ καραντίνα

**Τι πιστεύεις ότι νιώθει κάποιος ασθενής όταν θέλει να επικοινωνήσει μαζί σου και δεν τα καταφέρνει;**

Τι βιώνει ο άρρωστος;

**Ναι τι πιστεύεις ότι νιώθει;**

Στρες , καθαρά, θεωρώ ότι είναι εκείνο που είπα πριν ότι πρέπει να προετοιμαστεί κατάλληλα ο άρρωστος και φαρμακευτικά ούτως ώστε να μπορεί να αντιμετωπίσει την κατάσταση που αντιλαμβάνεται εκείνη την φάση τι του συμβαίνει, δηλαδή αν είναι διασωληνωμένος και εν κοιμισμένος και εν καλά κοιμισμένος δεν αντιλαμβάνεται , ύστερα αντιλαμβάνεται που ξυπνά σε ποια κατάσταση είναι και είναι εκεί που γίνεται παραπάνω το στρες τους άρα πρέπει εγώ να βρω κάποιους τρόπους να διαχειριστώ αυτό το πράγμα για να μπορεί να με βοηθήσει στην αποκατάσταση του

**Δηλαδή τι κάνεις τι εισηγείσαι δηλαδή;**

Εντάξει εδώ μπλέκω και το ιατρικό κομμάτι μέσα δηλαδή έτυχε μου πάρα πολλές φορές περιστατικά που να λέω του γιατρού κάλυωε τον με φάρμακα ας πούμε να καλμάρει για να μπορείς να τον δουλέψεις, αφού ξυπνάς τον και βλέπεις ότι ο ασθενής ανεβάζει 200 πίεση γιατί μπαίνει σε τούτη την στρεσογόνη κατάσταση, πως θα δουλέψεις αυτόν τον άρρωστο, δηλαδή δώστου Seroquel ας πούμε δυο μέρες πριν , ετοίμασε τον και ύστερα ξύπνα τον, δεν γίνεται να τον ξυπνάς αμέσως μπαμ και να ξυπνά ο άρρωστος και να αντιλαμβάνεται την κατάση που είναι , πως ένα δουλέψεις πάνω του ,πως ένα το απογαλακτίσεις και τα αποτελέσματα να ξανακοιμίζουμε τους αρρώστους, δηλαδή πράματα που τα είδαμε αρκετές φορές.

**Εσένα ως Alexandros πως σε κάνει να νιώθεις εκείνη την στιγμή, αν νιώθεις κάτι φυσικά μην τα παίρνουμε όλα δεδομένα , αν προσπαθείς επανειλημμένα να επικοινωνήσεις με ένα ασθενή και δεν τα καταφέρνεις τι σε κάνει να νιώθεις εκείνη την ώρα**

Εάν θεωρώ ότι εν κακές πρακτικές ,στρεσάρουν με και εμένα, δηλαδή μέσα από την εμπειρία και την γνώση μας που έχουμε αλλά και το υπόβαθρο το ακαδημαϊκό που έχουμε, δηλαδή κάποια πράματα που ξέρουμε ότι υπάρχουν δουλεύονται και γίνονται σε κάποιου επιπέδου εντατικές, στρεσάρουν με ότι έβλεπα κακές πρακτικές στο θέμα της προετοιμασίας για να προχωρήσεις τον ασθενή

**Θέλεις να μου πεις κάποιο παράδειγμα που άλλες εντατικές που ξέρεις;**

Ένα απλό παράδειγμα , απλό πολλά απλό, είδα σε άλλη εντατική, δεν ξέρω αν υπάρχει λόγος να αναφέρουμε,

**Ότι νομίζεις**

Πολλά απλό, εχτός που σου είπα το Seroquel, που εν κάτι εξειδικευμένο, μισή ώρα πριν τον απογαλακτισμό να δίνεται apotel , παρακεταμόλη, απλό πράμα, αλλά είναι στην ρουτίνα τους ότι δίνεται εκείνο το φάρμακο και ότι βοηθά να ξυπνήσει ο ασθενής πιο ήρεμα ο άρρωστος, κάτι που έπρεπε να πω εγώ για τους αρρώστους να βάλουμε και μια apotel , εντάξει δεν μας χαλά να βάλουμε, βάλε , ή για παράδειγμα τα πιο απλά που κάνουν κάποιοι άλλοι δεν τα κάνουμε, τούτο το πράμα με στρέσαρε και εμένα

**Πόση ώρα διαρκεί τούτη η ώρα που μου λες πάνω κάτω, δηλαδή πόση ώρα, θα βρω ένα διασωληνωμένο χωρίς sedation ασθενή στην μονάδα σας**

Καταρχάς εξαρτάται που τι καταστολές παίρνει

**Ένα average ας το πούμε.**

Δεν ξέρω αν μπορούμε να το καθορίσουμε τούτο το πράμα

**Οκ από την εμπειρία σου τότε.**

Δύσκολο μου να απαντήσω, διότι δεν είναι το φαρμακευτικό που παίρνει είναι ο οργανισμός που το παίρνει, τα επίπεδα που έχει στον οργανισμό του, έτυχε μου ασθενής που έκαμε και 5 ώρες να αρχίσει να ξυπνά , έτυχε .. δεν μπορώ να σου πω…

**Κοίταξε εγώ θέλω να μου πεις με εκείνα που σου τύχαν εσένα, δεν θέλω να μου πεις για όλους, εννοώ που εκείνα που σου έτυχαν εσένα δεν έχει σωστό και λάθος, είναι την δική σου εμπειρία που θέλω να δω**

Εντάξει ένα περίπου ένας ασθενής που θα του κόψεις τις καταστολές δεδομένου ότι εν παίρνει μυοχάλαση πολλά πιο.., δηλαδή απλά κατασταλτικά εε στο μισάωρο ξεκινά να κάνει και δικές του αναπνοές, ας πούμε μια ώρα το πολύ με επιφύλαξη, θεωρώ ότι εν κάτι που εν έχει απάντηση , δηλαδή κάθε οργανισμός εν διαφορετικά , εν διαφορετικά τα φάρμακα δεν είναι standard, είναι κάτι που θα τον προετοιμάσεις που πριν, δηλαδή κόψε του η ώρα 12 την νύχτα το dormicum ας πούμε και αφηστον με dripivan, το οποίο απορροφάτε και φεύγει γρήγορα από τον οργανισμό και η ώρα 7 το πρωί κόψε την diprivan, η ώρα 7 20 θα είναι ξύπνιος ο ασθενής, εξαρτάτε τι παίρνει ..

**Οκ κάτι που ήθελα να σε ρωτήσω και επαραβλεψα τα στην αρχή είναι, ξέχασα να σε ρωτήσω, πόσα χρόνια δουλεύεις σύνολο ως νοσηλευτής**

Οσα χρόνια είμαι στην εντατική

**Δηλαδή**

8,5

**8,5 χρόνια δηλαδή είσαι σύνολο ως νοσηλευτής και δουλεύεις στην εντατική;**

Ναι

**Δηλαδή δεν δούλεψες σε άλλο τόπο**

Όχι

**Πόσο χρονών είσαι**

31

**Που έχεις τελειώσει**

Σχολή, ΤΕΠΑΚ

**Έκανες κάποιο μεταπτυχιακό**

Ναι

**Πιο έκανες**

Το καλύτερο

**Δηλαδή**

Εμμ εντατική και καρδιολογία στο ΤΕΠΑΚ

**Έκανες οτιδήποτε άλλο εκτός από αυτό;**

Τα course τα ALS, INSTRUCTOR BLS, κάτι ταχύρρυθμα εντατικής, ε αυτά

**Μάλιστα.. εμμ επιστρέφω ξανά σε μια ερώτηση που σου ξαναέκανα, στους τρόπους επικοινωνίας, έχει , θυμάσαι κάποιο περιστατικό που να σου έκανε εντύπωση για οποιοδήποτε λόγο που να θέλεις να μοιραστείς μαζί μου; Κάποιο έτσι διαφορετικό ή που σου έκανε εντύπωση εσένα για οποιοδήποτε λόγο**

Σε ότι αφορά τον ξύπνιο διασωληνωμένο;

**Ναι**

Εεεε έχω τούτο κακό/καλό να ξεχνάω και θεωρώ το επαγγελματισμό αυτό το πράγμα προσωπικά να μην με στιγματίζουν και να θυμούμαι ιδιαίτερα πράγματα, το πιο απλό που μπορώ να σκεφτώ είναι ότι έτυχε μας αρκετές φορές η προθυμία του αρρώστου να θέλει τόσο πολλά να απογαλακτιστεί και να βρέθετε με τον σωλήνα να τον κρατά μέσα στο χέρι, να τραβούν τον σωλήνα να φκαίνει μόνος του, και όσοι ασθενείς έκαμαν τούτο το πράμα κανένας δεν ξαναδιασωληνώθηκε επείγον

**Ήταν σε θέση δηλαδή (θέλοντας να πω ήταν έτοιμοι για να απογαλακτιστούν)**

Δηλαδή οι άνθρωποι αποφάσιζαν για τον εαυτό τους μόνοι τους, δηλαδή μπορώ να σου πω ότι οι άνθρωποι αποφάσιζαν για τον εαυτό τους μόνοι τους

**Οκ να πάω αλλού, τότε να πάω αλλού, ποιος πιστεύεις ότι εν ο παράγοντας που κατέληγε να γίνει αυτό το λάθος, δηλαδή τι θα μπορούσαμε να διορθώσουμε για να μην καταλήξει έτσι ο ασθενής, πάω πίσω λίγο τώρα, κατευθύνω την ερώτηση, είπες μου πριν ο χρόνος**

Σίγουρα είναι μια διαδικασία που πρέπει να είσαι από πάνω του ασθενή το οποίο πιθανό να μην είναι εφικτό, τούτο που λέγαμε ότι κόβεις τις καταστολές, δεν μπορείς να είσαι συνέχει από πάνω από ένα άρρωστο, ο άρρωστος μπορεί να ξυπνήσεις εσύ να κάνεις κάτι άλλο και να είναι σε θέση να τραβήξει τον σωλήνα, εν τούτο που είπαμε, ένας άρρωστος στην μισή ώρα να είναι πλήρης ξύπνιος, εεε τούτο παραπάνω, ότι πρέπει να έχεις παραπάνω έλεγχο, την ώρα που ξυπνά ο ασθενής να είσαι που πάνω του

**Από πόσους ασθενείς έχετε**

Έχουμε 11 κρεβάτι , 8 αναπνευστήρες και πολλές φορές δανειστήκαμε αναπνευστήρες από άλλο νοσοκομείο, μια λεπτομέρεια που είναι πολύ σημαντική είμαστε ανοικτού τύπου εντατική η οποία δεν έχει εντατικολόγο, δηλαδή το θεραπευτικό κομμάτι αναλαμβάνει το η ειδικότητα της εισαγωγής το αναισθησιολογικό και εν μέρη αιμοδυναμικό κομμάτι ο αναισθησιολόγος

**Καλά πως γίνεται να λειτουργά εντατική χωρίς εντατικολογο, πες μου αυτό το πράγμα**

Εεε αφηστο γιατί δεν θα κάνουμε άλλη δουλειά, θα μείνουμε ως αύριο να συζητούμε

**Από όσο γνωρίζω στην Λευκωσία έχει 11 εντατικολόγους νομίζω, κάτι τέτοιο άκουσα τελευταία**

Πρέπει να αυξηθήκαν λόγω covid τώρα

**Δηλαδή σκεφτόμασταν μια φορά με μια συνάδελφο ότι ήταν ένας εντατικολόγος προς 2 ασθενείς νομίζω, είχε τόσους πολλούς**

Εντάξει τωρά εμεγαλώσαν πολλά οι εντατικές, δεν νομίζω να αναλογεί τουτο το πράμα

**Οκκ**

Και η Πάφος έτσι δουλεύει, έχει και άλλα νοσοκομεία που δουλεύουν έτσι

**Όπως εσάς στην λάρνακα**

Ναι, μόνο η Λευκωσία και η Λεμεσός έχουν εντατικολόγους

**Άδικο τούτο το πράμα, τέλος πάντων ενεν ώρα να το κρίνουμε τούτο, ξαναπάω πίσω στην ερώτηση την ίδια, με τους τρόπους επικοινωνίας, θέλω να ξαναμπείς έτσι λίγο στο πνεύμα εκείνης της στιγμής και στο περιβάλλον και πες ότι εγώ είμαι ο κύριος Γ/Α πως θα έρθεις να με πλησιάσεις και τι θα μου πεις όταν προσπαθώ να σου μιλήσω ή όταν θέλεις εσύ να μου μιλήσεις, μπορείς να μου κάνεις αναπαράσταση ας το πούμε, να δω τους τρόπους επικοινωνίας που θα μιλήσεις**

Το πρώτο πράμα που θα σκεφτώ είναι το άγγιγμα, θα πάω κοντά του θα τον αγγίξω και η ερώτηση μου θα είναι μονολεκτική για να δω αν ακούει και αντιλαμβάνεται και μπορεί να μου απαντήσει σε κάτι που ένα τον ρωτήσω, δεν θα το κάνω ως ερώτηση άμεση , θα πω κ Μ ξέρω ότι με ακούσεις ,θα τις περιγράψω σε ποια κατάσταση είναι, δηλαδή έχεις ένα σωλήνα μέσα στο στόμα σου και σε βοηθά να αναπνεύσεις και είχαμε σε και εκοιμάσουν και τωρά προσπαθούμε να σε ξυπνήσουμε , πρέπει να το ανεχτείς εκείνο το πράμα να δουλέψεις μαζί του να αναπνέεις καλά αργά και να συνεργαστείς, θα την ρωτήσω, πονεις κλείσε μου τα μάτια σου αν πονείς για να ξέρω αν θα της χορηγήσω φάρμακο, δηλαδή θα εστίαζα περισσότερο στην ανησυχία στον καθησυχασμό και στον πόνο, δηλαδή αν νιώσει ασφάλεια, γιατί μπορεί να με ακούσει και να μην με βλέπει , μπορεί να είναι στην φάση που αντιλαμβάνεται και ακούει φυσικά αλλά δεν άνοιξε τα μάτια της ακόμα, αλλά βλέπω στο μόνιτορ, ότι με βάση τα στοιχεία που μου δείχνει είναι ότι είναι ξύπνιο το άτομο και έχει μια ανησυχία, μάλιστα, άρα θα το κάνω να νιώσει ασφάλεια ότι είμαι δίπλα του και θα πιάσω τις πληροφορίες που με ενδιαφέρουν που εκείνη την φάση είναι ο πόνος και το στρες, γιατί τα άλλα ούλλα ότι αφορούν την θεραπεία για να φτάσει στο σημείο να τον ξυπνώ εκεί σημαίνει ότι πήρε έλεγχο και ξέρω σε ποια φάση είναι θεραπευτικά.

**Όταν κάποιο σου παίρνει ώρα να επικοινωνήσεις και δεν τα καταφέρνεις , κάνεις οτιδήποτε άλλο ας πούμε για πχ, ένα ζητήσεις την βοήθεια κάποιου συνάδελφου, πχ να του πεις έλα να δούμε τι θέλει ,μπορεί να καταλάβεις εσύ καλύτερα; Πχ λέω τώρα**

Ότι δεν καταλαβαίνω τι θέλει να μου πει

**Ναι να ζητήσεις μια δεύτερη γνώμη**

Ή ότι είναι σε επικοινωνία μαζί μου ( *εδώ δεν κατάλαβα τι θέλει να πει*)

**Ότι δεν καταλαβαίνεις τι θέλει να σου πει**

Ναι μπορεί να ζητήσω βοήθεια, σαφώς, συνάδελφε κάτι μου λέει και δεν καταλάβω, ένα εσύ να δεις , καταλάβεις; Έτυχε αρκετές φορές

**Άλλο που ήθελα να ρωτήσω είναι στην Μονάδα σας έχετε μέσα αυξητικής επικοινωνίας**

Πες το ξανά πως το είπες

Μέσα αυξητικής επικοινωνίας

**Οκ πρώτη φορά το ακούω, πίνακες με γράμματα..**

Ναι κατάλαβα τι είναι

**Ή εικόνες, πίνακα, η το πιο απλό πέννα κολλά μαρκαδόρο..**

Χρησιμοποιήσαμε σαφώς χρησιμοποιήσαμε, μπορώ να σου αναφέρω, κόλλα πέννα ναι έτυχε μας αρκετές φορές

**Λειτουργά**;

Ναι σίγουρα, δλδ αν είναι σε θέση ένα άτομο να γράψει και να μην μπορεί να μιλήσει, σαφώς, τούτο που θα σου πω βγαίνουμε λίγο από το θέμα σου φυσικά που τα δουλέψαμε τούτα τα πράματα , σε ασθενείς με ALS

**Ήταν διασωληνωμένοι όμως;**

Τραχειοστομημένοι , ναι εννοώ σου σε θέμα επικοινωνίας, για αυτό που σου λέω φεύγει λίγο από το θέμα σου, εν το σύνδρομο του κινητικού νευρώνα, παράλυση του κινητικού νευρώνα , πορυ βρίσκεις οποιοδήποτε άλλο τρόπο να επικοινωνήσεις μαζί του και εν τούτοι οι πίνακες εν τούτα τα πράματα, συνήθως εν κόλλα και πέννα

**Άρα στους διασωληνομένους ξύπνιους εν κόλλα και πέννα συνήθως;**

Έτυχε να χρησιμοποιηθεί ενεν συνήθως, δεν είναι ρουτίνα..

**Έτυχε μια φορά στο κάθε τόσο**

Να κάμω εγώ μια ερώτηση;

**Ναι**

Πόσο θεωρείται ένας άρρωστος να είναι ξύπνιος με ένα σωλήνα μέσα στο στόμα

**Δεν ξέρω, τι εννοείς; Πόσο είναι το χρονικό περιθώριο εννοείς;**

Εγώ θεωρώ και προσπαθώ πάντα σε θέμα ξύπνιου διασωληνομένου να κρατώ ένα άρρωστο για κάποιο λόγο, δηλαδή να τον δοκιμάσω να ξυπνήσει αν δεν μπορεί αν δεν είναι σε θέση για κάποιο λόγο, να τον ξανακοιμήσω να τον δουλέψω και να κάνω προσπάθεια ξανά, δεν θα τον αφήσω δέκα ώρες ένα ασθενή με ένα σωλήνα μέσα στο στόμα και να του λέω ηρέμησε ηρέμησε ηρέμησε

**Σωστό**

Εντάξει απλά εν τούτο ήθελα να το ξεκαθαρίσω

**Εγω δεν ξέρω, εγω ρωτώ για να δω τι εμπειρίες έχετε, δεν έχω κάποιο ύποβαθρο και έρχομαι να το συγκρίνω οσο αφορά αυτό το πράμα, εε ξεχασα τι ήθελα να σε ρωτήσω, έχετε από αυτούς τους πίνακες που σου είπα για παράδειγμα, με είκόνες και κλπ**

Εκεινοι που σου είπα τις έφερναν μαζί τους

**Αληθεια**

Που σου είπα για ΑLS

**AA ok, μιλούμε για άλλα περιστατικά όμως**

Ναι ναι, όχι δεν έχουμε έτσι πίνακες..

**Άλλο πράγμα που ήθελα να σε ρωτήσω, τελειώνουμε σε λίγο., ήθελα να σε ρωτήσω στην καριέρα σου τελικά έτυχε σου ή στο πτυχίο σου η στο μεταπτυχιακό σου κλπ. έτυχε σουτ να εκπαιδευτείς ..**

Κάνουμε καριέρα; χαχα, θεωρείται καριέρα;

**Δεν γνωρίζω, στην πορεία σου ως νοσηλευτής , έτυχε σου να εκπαιδευτείς σε θέματα επικοινωνίας;**

Στο συγκεκριμένο θέμα ή επικοινωνίας γενικά;

Ναι σίγουρα και στα πλαίσια του μεταπτυχιακού, είχαμε μάθημα επικοινωνίας

**Γενικά που μιλάμε τώρα ή για διασωληνο΄μενους ασθενείς;**

Γι’ αυτό ρώτησα, γενικά σίγουρα, πιθανό και διασωληνομένους αλλά διαφεύγει μου αυτή την στιγμή, ίσως πιο παλιά, έτσι αχνόφαι.. σίγουρα επικοινωνία έκανα και σαν μάθημα και στο μεταπτυχιακό και..

**Μάλιστα αν σου τυχαίνει η ευκαιρία να εκπαιδευτείς σε αυτά τα θέματα, σε ότι αφορά είτε σε διασωληνομένους ξύπνιους, είτε ξανά σε επικοινωνία κλπ. θα ήθελες να εκπαιδευτείς ή όχι;**

Ναι σίγουρα, θεωρώ το ένα πολλά πολλά σημαντικό κομμάτι για τον ασθενή , η συγκεκριμένη διεργασία, το να ξυπνήσεις ένα ασθενή και να είναι διασωληνωμένος , ναι σίγουρα, χρειάζεται πολύ πάρα πολύ και η επιστημονική , δηλαδή όχι ότι αφορά την ΜΕΘ μόνο, ένας άνθρωπος , ένας νοσηλευτής μόνο, δηλαδή αφορά τον γιατρό ,πρέπει να γνωρίζει ο γιατρός να προετοιμάζει τον ασθενή να ξυπνήσει με τις κατάλληλες συνθήκες για να μπορεί ο νοσηλευτής να δουλέψει πάνω του, αφορά όλη την ομάδα δεν αφορά μόνο ένα νοσηλευτή, δεν μπορεί ένας νοσηλευτής να δουλέψει μόνος του , όση γνώση και να έχει

**Μάλιστα , ως Alexandros με πιο τρόπο πιστεύεις θα σε βοηθούσε καλύτερα το μάθημα, πως να γινετουν το μάθημα, σε μια τάξη, εξ αποστάσεως, δίπλα από τον ασθενή , να ήταν μεικτό, με πιο τρόπο για να σε βοηθούσε εσένα να τοπ αφομοιώσεις καλύτερα**

Σίγουρα σε μια τάξη, όχι μόνο στο χαρτί, θεωρώ εν κάτι που έχει κλινικό κομμάτι,, εκείνος που θα με διδάξει να με πάρει και στον άρρωστο και να κάνουμε την διαδικασία μαζί

**Οκ σε γενικές γραμμές αυτά ήθελα να ρωτήσω, απλά ξανά τελευταία φορά ρωτώ έχεις κάτι άλλο να μου προσθέσεις ότι αφορά τα εμπόδια επικοινωνίας , εε μπορείς να σκεφτείς, είπες μου ο χρόνος οι γνώσεις, μπορείς να σκεφτείς κάτι άλλο που σε εμποδίζει να επικοινωνήσεις με αυτούς τους ασθενείς**

Εξοπλισμός, εγκαταστάσεις, είναι πολυπαραγοντικό το θέμα. Δηλαδή για να μην το κάνουμε, που την πιο απλή πρακτική ως το πιο expensive , δηλαδή αν έχει σε άλλες χώρες ρομπότ που να διαβάζουν και την σκέψη του ασθενή , σημαίνει εν κάνουμε τίποτε, δηλαδή με το τι θα σκεφτώ μέσα στο μυαλό του αν θα μπω στην θέση του αν θα νιώσω τι βιώνει και να σκεφτώ εγώ με την λογική μου να τον βοηθήσω, δεν έχουμε κάτι δεν υπάρχει κάτι, εναπόκειται στον κάθε ένα πως θα το αντιμετωπίσει αυτό , είπα σου θεωρώ οι πρακτικές που δεν εφαρμόζονται και είναι σε άλλες χώρες κατευθυντήριες οδηγίες ή πρωτοκολλά εννοώ εν ανεπάρκεια γνώσης, δηλαδή όταν δεν γνωρίζω γι’ αυτό πως θα το εφαρμόσω, αφού δεν γνωρίζω γι’ αυτό..

Συνέντευξη Νο 13

**Για τα επίσημα…**

**Καταρχάς πόσο χρονών είσαι;**

Κυρία δημοσιογράφε,χαχα, είμαι 41 χρονών

**Πόσα χρόνια εργάζεσαι ως νοσηλευτής σύνολο;**

18

**18, Στις μονάδες πόση εμπειρία έχεις, πόσα χρόνια εργάζεσαι;**

6 χρόνια

**Που έχεις τελειώσεις τις σπουδές σου;**

Στην νοσηλευτική σχολή που ήταν η σχολή, μετά στο ΤΕΠΑΚ, που ήταν η αναβάθμιση και τώρα προσπαθώ να τελειώσω το μεταπτυχιακό μου

**Οκ που κάνεις το μεταπτυχιακό σου;**

Στο ΤΕΠΑΚ

**Με τι έχει να κάνει το μεταπτυχιακό σου;**

Mε την εντατική θεραπεία και την κατεύθυνση την καρδιολογική

**Μάλιστα το πρώτο πράγμα που θα ήθελα να σε ρωτήσω, όταν μπούμε στην μονάδα σας στο νοσοκομείο της λάρνακας σε ποιες περιπτώσεις θα βρω ένα διασωληνωμένο ασθενή που να είναι εκτός καταστολής;**

Σε ποιες περιπτώσεις… ένα ασθενή που προσπαθούμε να τον ξυπνήσουμε , δηλαδή φυσικά πρέπει να τονίσουμε ότι στο ΓΝ Λάρνακας δεν υπάρχουν εντατικολόγοι άρα δεν είναι μια καθαρή εντατική, δηλαδή καρδιολογική εντατική, πιάνει όλα τα .. διάφορες κατηγορίες ασθενών, μπορεί να πιάνει παθολογικά καρδιολογικά, τα πάντα , οι υπεύθυνοι για τους ασθενείς είναι οι γιατροί των κλινικών, δηλαδή υπεύθυνος θα είναι ένας παθολόγος για ένα διασωληνωμένο ασθενή που μπορεί να μην έχει ιδέα για ένα διασωληνωμένο ασθενή, υπεύθυνος μπορεί να είναι ο καρδιολόγος για ένα διασωληνωμένο ασθενή που μπορεί να είναι ο καλύτερος καρδιολόγος αλλά πάλι να μην έχει ιδέα για ένα ασθενή, βασικά πέφτει όλο το βάρος της ευθύνης για το ξύπνημα κλπ. στους αναισθησιολόγους, οι οποίοι αναισθησιολόγοι δεν είναι εκπαιδευμένοι για ένα ασθενή σε κατάσταση χειρουργείου, όχι να ξυπνούν ένα ασθενή ο οποίος ήταν δέκα μέρες κατεσταλμένος σε κατάσταση εντατικής είναι λίγο συγχυσμένη η κατάσταση στην Λάρνακα, εντάξει.. άρα στο νοσοκομείο Λάρνακας θα έβρεις ένα ασθενή που είναι διασωληνωμένος και ξύπνιος την ώρα που θα τον ξυπνούν

**Οκ είδες γενικά, έστω μια φορά ή έστω περισσότερες φορές οποιαδήποτε άλλη περίπτωση που να χρειαστεί να έχετε διασωληνωμένο και ξύπνιο, γενικά μιλώ τώρα.**

Διασωληνωμένο και ξύπνιο….

**Εχτός από την φάση του απογαλακτισμού που μου περίγραψες τώρα**

Να μην κοιμάται καλά, να του δίνουμε καταστολές αλλά οι καταστολές να είναι λίγες για τον ασθενή

**Οκ μάλιστα, η διαδικασία που μου περιέγραψες σε λίγο δηλαδή στην φάση του απογαλακτισμού, που μου είπες ότι κλείνετε τις καταστολές για να αφυπνίσετε ένα ασθενή , περίπου πόση ώρα παίρνει, για πόση χρονική διάρκεια μιλούμε;**

Για τον απογαλακτισμό του ασθενή;

**Ναι**

Κοίταξε επειδή όπως σου έχω πει στην αρχή ο λόγος που σου έκανα εκείνο τον πρόλογο όλο είναι για να καταλάβεις ότι δεν έχουμε κάποια πρωτόκολλο εμείς όπως έχουν κάποιοι άλλοι, πχ βάλω τον ασθενή σε SMIV ή SPONTANEOUS ύστερα βλέπω τους όγκους που βγάλει ανά λεπτό κλπ. και μπορώ να καταλάβω ότι μπορεί να βγει ο ασθενής, δείχνει μου έτσι ότι ο ασθενής μπορεί να βγει που μόνος του, ένα έρθει ο αναισθησιολόγος και να σου πει βάλε τον SPONTANEOUS και θα έρθω σε μια φάση να τον δω και μπορεί εκείνη φάση να τελειώσει τα χειρουργεία του και ύστερα να τον δει και μπορεί εκείνη την φάση να τον πιάνεις τηλέφωνο και να το λες πχ γιατρέ κάνει τούτους τους όγκους αυτές τις αναπνοές και να σου πει ξανακοίμησε τον ή άφησε τον να τον ξαναδούμε ή ξανακάνε του αέριο και βλέπουμε .. δεν υπάρχει πρωτόκολλο αφύπνισης ασθενή

**Δηλαδή είναι στην κρίση του αναισθησιολόγου που θα σου πει**

Στην κρίση του αναισθησιολόγου… ναι ναι βασικά εκείνου

**Ωραία πρόσεξες πάνω κάτω περίπου που την πιο λίγη ώρα ως την πιο πολλή να μου πεις εννοείται, περίπου διάστημα ,γιατί ο κάθε ασθενής είναι διαφορετικός εννοείται και το κάθε περιστατικό.**

Μπορεί να πάρει και 15 ώρες , το πιο λίγο που μπορεί να πάρει; Πάνω κάτω λέω τώρα

Το πιο λίγο, δεν μπορώ να θυμηθώ τώρα αλλά είναι λίγη ώρα, μπορεί να πάρει ενάμιση ώρα , δυο ώρες πχ, αλλά έτυχε μας να πάει πολλές ώρες να κάνουμε την αφύπνιση και να ξύπνιος ο ασθενής , στο τέλος να κουραστεί ο ασθενής και στο τέλος να τον κοιμίσουμε

**Σε εκείνο το περιστατικό που μου λες τώρα δηλαδή τι έτυχε και έπρεπε να μείνει τόσες ώρες ξύπνιος και να μην τον αποσωληνώσεται για παράδειγμά**

Δεν είχε αναισθησιολόγο πάνω

**Οκ, ενώ ο ασθενής ήταν έτοιμος να το βγάλετε τον σωλήνα, δεν τον βγάλετε γιατί δεν είχε κάποιο ειδικό δίπλα σας πχ;**

Ναι

**Μάλιστα, οκ τι περιστατικά…**

Για να ξυπνήσεις τον ασθενή πρέπει να του κάνεις κάποια τεστ και πρέπει να τικάρεις(σημειώσεις) πάνω στην φόρμα σου ότι έκανες το συγκεκριμένο τεστ και είδες ότι ο ασθενής σου ότι αντέχει εκτός αναπνευστήρα, για να γίνει αυτό όμως χρειάζεται κάποιο ειδικό, όμως άμα ο ειδικός εν στο χειρουργείο , δηλαδή άμα στο νοσοκομείο έχει 4 αναισθησιολόγους, έχει 4 χειρουργεία και είναι όλοι στα χειρουργεία και ύστερα ο επι καθηκοντι έχει να κάνει και προ-εγχειρητικό έλεγχο και πρέπει να πάει και στους χειρουργικούς και ορθοπεδικούς θαλάμους να κάνει και προ-εγχειρητικό και στο τέλος να έρθει πάνω, ε υπολόγισε ότι εκείνη όλη την ώρα οπ ασθενής είναι μόνος του μαζί με τους νοσηλευτές αλλά δεν έχεις το δικαίωμα να τον αποσωληνώσεις εσύ, πρέπει να έρθει ο γιατρός πάνω και να σου δώσει το οκ να τον αποσωληνώσεις ..

**Μάλιστα, εσύ πως καταφέρνεις να επικοινωνήσεις με τούτους τους ασθενείς. εγώ θα επικεντρωθώ με αυτούς τους ασθενείς, ξέρω ότι βλέπετε πολλά άτομα και τραχειοστομημένα και με ΜΕΜΑ, αλλά εγώ θέλω να επικεντρωθώ σε αυτή την φάση που ο ασθενής είναι διασωληνωμένος ,όχι τραχειοστομημένος και αφυπνισμένος εννοώ εκτός καταστολής , πως καταφέρνεις να επικοινωνήσεις εσύ σε εκείνη την φάση , δηλαδή τι του λες και τι κάνετε , μπορείς να μου περιγράψεις έτσι λίγο πιο λεπτομερώς**

Είναι σαν να επικοινωνείς με ένα άρρωστο ο οποίος μπορεί να κάνει τα πάντα εκτός από το να μιλήσει δηλαδή με ένα , πως λέμε εκείνο που δεν μιλά, βουβό..

**Δηλαδή εγώ είμαι ο κύριος Γιαννάκης , λέω τώρα τι θα μου πεις τώρα, πως θα το κάνουμε, θέλω να μπω λίγο σε λεπτομέρειες**

Θα σου πω καλημέρα κ. Γ και θα μου πεις καλημέρα, αλλά το καλημέρα δηλαδή το κούνημα των χειλιών ένα το κάνεις , ένα προσπαθείς να λες καλημέρα απλά δεν θα βγαίνει η φωνή λόγω του ότι είσαι διασωληνωμένος, τα υπόλοιπα θα τα κάνεις, δηλαδή θα μου δείχνεις εε θέλω νερό , θέλω φαΐ θέλω να βγάλω τον σωλήνα τούτο , εεε θέλω μπάνιο, ενεργήθηκα, θέλω να κάτσω εε θα μου λες τα πάντα αλλά με κινήσεις ή με τα μάτια σου, απλά δεν θα μου το λες με την φωνή σου

**Οκ κινήσεις των ματιών , των χεριών και των ποδιών που μου έδειξες τώρα ας πούμε**

Ναι τα πάντα εκτός από την φωνή, μπορεί να χρησιμοποιήσει όλους τους τρόπους επικοινωνίας , ο διασωληνωμένος ο ασθενής ο ξύπνιος, όλους τους τρόπους εκτός από την λεκτική

**Οκ, εμμ καταρχάς να σε ρωτήσω, έτυχε σου ποτέ να δυσκολευτείς να μιλήσεις με τούτους τους ασθενείς να μην καταλαβαίνεις τι θέλουν να πουν**

Εντάξει θα σου τύχει και κάποια φορά να μην καταλαβαίνεις τι θα σου τύχει και κάποια φορά να μην καταλαβαίνεις αλλά συνήθως καταλαβαίνεις θα σου δήξει τι θέλει, συνήθως λέει σου εξηγεί σου , λέει σου πότε θα βγάλουμε τον σωλήνα και δείχνει σου το με κινήσεις ,πότε θα τον βγάλουμε ή πεινώ ή καταλαβαίνεις τι θέλει να σου πει

**Οκ στις περιπτώσεις που δεν κατάλαβες τι θέλει να σου πει, τι πιστεύεις ότι είναι εκείνο που σε εμποδίζει να καταλάβεις τι θέλει να σου πει**

Είχε φορά που τον έβαλα και έγραψε μου

**Μάλιστα οκ**

Κατάλαβες; δεν είναι δύσκολο να καταλάβεις τι θέλει ο άλλος ,δηλαδή είναι σαν να λέμε ότι έχουμε ένα ασθενή ο οποίος δεν μιλά και δυσκολευόμαστε να καταλάβουμε, έχουμε άλλους τρόπους επικοινωνίας, θα έβρεις άλλους τρόπους επικοινωνίας να επικοινωνήσεις μαζί του, θα σου δήξει τι θέλει, θα σου ζωγραφίσει τι θέλει, θα σου γράψει τι θέλει, δηλαδή δεν θα ζητήσει κάποια πράματα, εεμμ εκείνα που θα σου ζητήσει, είναι πράματα που τα έχει εκείνη την ώρα ανάγκη, δηλαδή έχει ανάγκη να τον καθαρίσεις έχει ανάγκη να του βγάλεις τον σωλήνα , έχει ανάγκη να του μιλήσεις γλυκά να του μιλήσεις γλυκά να του μιλήσεις ήρεμα να τον ηρεμήσεις, έχει ανάγκη να του δώσεις μια εικόνα που έχει δίπλα του που του έβαλαν οι δικοί του που άμα κάποιος εν θρησκευόμενος έχει ανάγκη να έχει μια εικόνα δίπλα του να την κρατά, δεν θα σου δήξει κάτι εκτός, θα σου δήξει πράματα που έχει ανάγκη εκείνη την ώρα

**Μάλιστα, ξαναεπιστρέφω , σε ξαναρώτησα αυτή την ερώτηση, σε περίπτωση που προσπαθείς να συνεννοηθείς με ένα ασθενή και δεν τα καταφέρνεις, ποιοι πιστεύεις ότι εν οι λόγοι που δεν καταφέρνεις να επικοινωνήσεις μαζί του**

Μπορεί εγώ εκείνη την ώρα να έχω πολύ δουλειά , μπορεί ο ασθενής να είναι ανήσυχος και εξαρτάτε τι γίνεται, δηλαδή αν είναι ένα εγκεφαλικό και διασωληνώθηκε, μια εγκεφαλική αιμορραγία μπορεί να είναι διαφορετικό, τώρα αν ήταν κάποιος ο οποίος ήταν ΧΑΠΙΤΗΣ και ήρθε με 150 διοξείδιο και τώρα έπεσε το διοξείδιο του στο 50-60 που είναι το φυσιολογικό του και είναι καλός ,μπορεί να επικοινωνήσει μαζί μου και είναι μια χαρά εκείνο που θα σε δυσκολέψει, είναι ο ασθενής δηλαδή αν δεν μπορεί να σου εξηγήσει εκείνο που θέλει και εσύ αν δεν θέλεις να μιλήσεις μαζί του, αν δεν θέλεις να επικοινωνήσεις μαζί του δεν θα επικοινωνήσεις, ή αν δεν έχεις τον χρόνο να κάτσεις να επικοινωνήσεις πάλι δεν θα επικοινωνήσεις, άμα θέλεις όμως θα έβρεις τον τρόπο

**πες μου και γι’ αυτά τα περιστατικά που μου έλεγες , οι απλά περιστατικά που απλά έτυχε , έκαμαν την ρουτίνα τους έκαμαν την επέμβαση τους , ξύπνησαν και όλα καλά, τούτα τα περιστατικά που μου είπες ας πούμε, μια εγκεφαλική αιμορραγία κλπ., δηλαδή εννοείς η κατάσταση του ασθενή, μπορείς να μην σου επιτρέπει, δηλαδή εννοείς μπορεί να εν συγχυτικος για παράδειγμα αν κατάλαβα καλά**

ναι μπορεί να ξυπνά ο άρρωστος και να είναι συγχυτικος, δηλαδή να ήταν πολλές μέρες διασωληνωμένος ή να παίζει και κάτι άλλο , ή να παίζει και κάτι άλλο και να χρειάζεται CT μήπως συναίνει κάτι άλλο

**οκ τι άλλα εμπόδια εκτός από αυτά βρίσκεις στο να επικοινωνήσεις , πχ ο χρόνος που έχεις εσύ να μιλήσεις, η κατάσταση του ασθενούς το πως είναι το τι θέλει να σου πει ο ασθενής όπως μου είπες τώρα, βρίσκεις κάποιο άλλο εμπόδιο**

μπορεί εσύ να θέλεις να επικοινωνήσεις και ο υπεύθυνος να σου λέει προχώρα πρέπει να βγει η δουλεία για παράδειγμα και αυτό είναι πολύ σημαντικό και θα μπορούσε να μπει στην έρευνα

**δηλαδή ο φόρτος εργασίας σας**

δηλαδή να σου πει ο υπεύθυνος μπες μέσα κάμε αυτά τα δυο τρία πράγματα που πρέπει να κάνεις , φύγε πήγαινε στους άλλους, μπορεί το προσωπικό να είμαστε τέσσερα άτομα και να έχουμε 11 ασθενείς και που τους 11 να είναι οι 8 διασωληνωμένοι, που έτυχε πολλές φορές , άρα πρέπει να μπεις να κάνεις τα βασικά και να φύγεις δεν μπορείς να μιλήσεις με τον ασθενή λόγω φόρτου εργασίας

**μάλιστα, πως πιστεύεις ότι νιώθουν τα άτομα τα οποία είναι διασωληνωμένα και κάποιες φορές να προσπαθούν να σου μιλήσουν αλλά να μην καταλαβαίνεις ή να μην μπορούν να εξηγηθούν για οποιοδήποτε λόγο, να μην μπορούν να εκφράσουν εκείνο που θέλουν**

εεε αν δεν μπορείς, αν θέλεις να εκφράσεις κάτι και ο άλλος δεν το καταλαβαίνει και καταλαβαίνεις ότι εν εσύ που δεν το δίνεις(εξηγείς) καλά, αγανακτείς νευριάζεις , και βλέπεις τους ότι κάποια στιγμή νευριάζουν , δηλαδή θέλουν να σου πουν αλλά δεν μπορούν να σου πουν , δεν ξέρω αν αντιλαμβάνεσαι τι σου λέω, δηλαδή προσπαθεί να μιλήσει και δε βγαίνει η φωνή του και νευριάζει και του λες εντάξει ηρέμισε και θα βρούμε άλλο τρόπο και μπορεί να του δώσεις να σου γράψει εκείνο που θέλει ή να σου δήξει εκείνο που θέλει

ε**σύ ας πούμε την ώρα που προσπαθείς να επικοινωνήσεις με τον ασθενή και βλέπεις ότι δεν τα καταφέρνει να επικοινωνήσει μαζί σου ή και εσύ δεν καταλαβαίνεις να επικοινωνήσεις μαζί του πως σε κάνει να νιώθεις εκείνη την ώρα , αν νιώθεις κάτι**

δεν με κάνει να νιώθω, απλά εκείνο που με κάνει είναι να πρέπει να αλλάξω σχέδιο , δηλαδή αφού εν μπορεί να επικοινωνήσει να αλλάξουμε τρόπο , γ αυτό μπορεί να μου γράψει εκείνο που θέλει, μπορεί να μου δήξει εκείνο που θέλει , θα βρούμε τρόπο να επικοινωνήσουμε , φτάνει να θέλουμε

**σωστό, μπορείς να μου περιγράψεις λίγους τρόπους που έτυχε να βρούμε εναλλακτικούς τρόπους επικοινωνίας ,εννοώ δηλαδή ας πούμε θα βρούμε κάποιο άλλο τρόπο το ένα είναι να σχεδιάσει , να γράψει να σου δήξει, έτυχε σου να κάμεις κάτι άλλο ας πούμε στο να καταλάβεις τι θέλουν**

όχι, νομίζω αυτοί είναι οι τρόποι επικοινωνίας, δηλαδή αν μου γράψει , ζωγραφίσει να μου δήξει δεν έχει άλλο τρόπο να μου το κάνει

**οκ μάλιστα είπες μου ότι χρησιμοποιείτε κόλλα και πέννα και μαρκαδόρο, δεν ξέρω τι ακριβώς χρησιμοποιείτε για να επικοινωνήσετε, γίνεται συχνά τούτο το πράμα μπορεί να γίνει συχνά αυτό το πράγμα ;**

βεβαίως και μπορεί να γίνει εννοώ άμα θέλεις ο νοσηλευτής ή ο γιατρός να επικοινωνήσει με τον άρρωστο θα πάρει μια κόλλα μια πέννα θα την αφήσει , εκείνη η κόλλα μένει μέσα δεν μπαίνει και βγαίνει στο δωμάτιο ούτως ώστε ο ασθενής να επικοινωνεί μαζί με τους φροντιστές του, την ώρα που θα μπεις μέσα να σου δήξει τι θέλει

**βοηθά αυτό το πράγμα, δηλαδή μπορεί να γράψει ο ασθενής πχ ή θέλει στην κατάσταση που είναι για παράδειγμα**

μπορεί να γράψει, μπορεί να του δώσεις εσύ, δεν ξέρω αν σου έτυχε καμιά φορά με σύνδρομο ALS που έχεις γραμμένο το αλφάβητο και δείχνουν σου, μπορεί να σου δήξει γράμματα-γράμματα ας πούμε θέλω νερό και να σου δήξει το Ν το Ε το Ρ και το Ο να του γράψεις τα 24 γράμματα του αλφαβήτου και να του λες δείχνε μου , άμα σου δήξει το Ν και το Ε θα καταλάβεις ότι είναι νερό και θα του πεις θέλεις νερό; Και να σου πει ναι (δήξει εννοεί) θα του πεις εντάξει να βγει ο σωλήνας και μετά , θα σου πει, να σου δήξει για παράδειγμα, ενεργήθηκα και να σου πει κακά ας πούμε , με το πρώτο ΚΑ που θα σου δήξει θα καταλάβεις και να του πεις είναι αυτό και θα σου πει ναι, μπορεί να μην σου το δήξει και να σου το δήξει με τούτο τον τρόπο,

**έχετε δηλαδή αυτούς τους πίνακες στην δουλεία σας**

όχι κάνεις τα εσύ, πιάνεις μια κόλλα Α4, γράφεις τα γράμματα ευανάγνωστα βλέπει τα ο άλλος και δείχνει σου, δεν θα μπορούσε να έχεις πίνακες και να μπαίνει από τον ένα ασθενή στον άλλο, μπορείς όμως να κάνεις αυτό το πράγμα και να σου δείχνει ο ασθενής και η κόλλα να μείνει στο δωμάτιο του μέχρι να ξυπνήσει ο ασθενής

**τούτο κάνουν το και οι συνάδελφοι σου;**

Κάμνουν το ρε Χαρίκλεια, άμα θέλει ο άλλος να κάμει θα κάμει, μπορεί να του πεις κάνε το και να σε ειρωνευτεί ο άλλος και να σου πει δεν είναι δουλειά μου ή δεν έχω χρόνο κλπ., μπορεί να έχει άτομα που το κάνουν μπορεί να έχει άτομα που δεν το κάνουν

**Τούτα που μου λες διδάχτηκες τα κάπου ας πούμε, είδες τα κάπου, πως κατέληξες να μάθεις για τούτα τα πράματα για τούτους τους πίνακες**

Είπα σου από άτομα που είχαν ΑLS σύνδρομο

**Δηλαδή ήρθαν αυτά τα άτομα ή είχε κάποιος ένα πίνακα είδες τον κατάλαβες ότι εν κάτι καλό και αντέγραψες το και έκαμες και στους άλλους πχ**

Ναι δηλαδή έκανες μου εντύπωση σε μια φορά που είχαμε ένα στις αρχές ένα κύριο με ALS σύνδρομο , το οποίο εν το σύνδρομο του κινητικού νευρώνα , ο ασθενής είναι τετραπληγικός δηλαδή , τα πάντα δεν μπορεί να κινήσει τίποτε, αλλά αντιλαμβάνονται τα πάντα , κατάλαβες , αντιλαμβάνεται τι θέλει , το μυαλό του λειτουργεί κανονικά και είδα την σύζυγο του ας πούμε που είχε αυτό τον πίνακα και εκείνος τι ήθελε επειδή δεν μπορούσε να της δήξει με το χέρι έδειχνε της με τα μάτια, δεξιά αριστερά και όπως αλλασε τα μάτια του άλλασε και εκείνη τα μάτια της και με τα πρώτα δυο τρία γράμματα καταλάβαινε τι ήθελε

**μάλιστα , τούτο με τον πίνακα δηλαδή με τα γράμματα χρησιμοποιείτε το σε άτομα που έχει ALS**

Μπορείς να τον χρησιμοποιήσεις σε οποιονδήποτε , οκ

**Στην πέννα και χαρτί σε ποια περιστατικά έτυχε να το χρησιμοποιήσεις**

Σε άτομα τα οποία ήταν ξύπνια , μπορούσαν να κινήσουν χέρια πόδια κανονικά τα πάντα ήταν τετρακινητικα απλά δεν μπορούσαν να βγάλουν φωνή και μπορούσαν να σου γράψουν τι θέλουν

**Μάλιστα εε για τον ασθενή..**

Όπως παρόμοιο πράμα με τούτο που σου λέω έτυχε μας και άτομα τα οποία δεν ήταν διασωληνωμένο αλλά είχε κάνει ολική λαρρυγκεκτομή

**Αλήθεια;**

Ναι, στην λαρυγκεκτομή αφού βγαίνουν οι φωνητικές χορδές, δεν μπορεί να μιλήσει και έγραφε μας τι ήθελε, κόλλα πέννα , που είναι ίδια φάση με τον διασωληνωμένο απλά επειδή αυτού έφυγε ο λάρυγγας του ώσπου να του έκαναν τραχειοστομια, είχαν του τραχειοστομία σορρυ, αλλά ώσπου να μπορούσε να περάσει ο καιρός και να του βάλουν την speaking την τραχειοστομία κλπ. και να μάθει να μιλά με την speaking έγραφε μας ο άνθρωπος

**Κάμνεις το συχνά εσύ τούτο με το χαρτί και την πέννα**

Κάμνω το συχνά όποτε χρειαστεί θα το κάμω, αν μπορεί να επικοινωνήσει θα το κάμω

**Στο τμήμα σας έχετε οποιαδήποτε άλλα μέσα επικοινωνίας, κάτι πιο εξελιγμένο όπως για παράδειγμα έχετε ας πούμε κάποιο υπολογιστή ταμπλετ που να έχει κάποιο πρόγραμμα που να σας βοηθά να επικοινωνείτε**

Όχι

**Οκ στο πανεπιστήμιο διδάχτηκες καθόλου για θέματα επικοινωνίας τρόπους επικοινωνίας**

Διδαχτήκαμε στο πρώτο εξάμηνο που την Κα.Κυράνου για την σωστή επικοινωνία

**Στο μεταπτυχιακό σου δηλαδή διδάχτηκες**

**Εε αφού μιλάμε τώρα για επικοινωνία και μέσα επικοινωνίας αν σου δινόταν η ευκαιρία να ξανά εκπαιδευτείς σε θέματα επικοινωνίας είτε σε διασωληνομένους ξύπνιους ή γενικά σε θέματα επικοινωνίας θα ήθελες να το κάνεις η όχι**

Βεβαίως θα ήθελα να το κάμω

**Οκ ως Aristotelis τι πιστεύεις ότι θα σε βοηθούσε εσένα καλύτερα να μάθεις, να ήταν διαδικτυακά να ήταν σε μια τάξη να ήταν δίπλα από τον ασθενή να ήταν κάτι ενδιάμεσο ας πούμε**

Εντάξει το καλύτερο θα ήταν, δίπλα από τον ασθενή δεν νομίζω γιατί θα κάναμε τον ασθενή να νιώθει άσχημα στην αρχή , εεε εμένα αρέσει μου να είμαστε κοντά , σε μια τάξη, το διαδικτυακό δεν μου αρέσει και πολύ , εε σε μια τάξη να το μάθουμε, να μάθουμε τους τρόπους και σιγά σιγά να προχωρήσουμε στους ασθενείς αλλά να πάμε μονομιάς σε ένα ασθενή μπροστά που έχει πρόβλημα επικοινωνίας και να κάνουμε μάθημα μπροστά του δεν θα μου άρεσε αυτό όχι

**Οκ μάλιστα, έχει κάποιο περιστατικό που σου έκανες εντύπωση για οποιοδήποτε λόγο και να θέλεις να μου το περιγράψεις, κάτι που να σου έμεινε στον νου**

Με επικοινωνία;

**Ναι , τούτο με λαρυγκεκτομή, ε μπορεί να μην ήταν διασωληνωμένος αλλά επικοινωνούσαμε πάρα πολύ καλά με αυτό τον άνθρωπο με τον γραπτό τον λόγο**

**Άλλο περιστατικό που να σου έμεινε στον νου πχ για οποιοδήποτε λόγο**

Εκείνος με το ALS πάλε που σου είπα , εντάξει περιστατικά που έμειναν πολλές ώρες στο spontaneous mode και έτυχε να κουραστούν και να τους ξανακοιμήσουμε ύστερα γιατί δεν τους προχωρούσαμε επειδή έλειπαν οι αναισθησιολόγοι , τούτα τα περιστατικά, εννοώ εν πολλά τα περιστατικά που να θυμηθώ ένα τώρα να σου πω αλλά σε θέμα επικοινωνίας το πιο τοπ που ήταν εκείνο που με βοήθησε εμένα , δηλαδή εγώ πιστεύω στην δουλειά μας παρακολουθείς και μαθαίνει κάποια πράγματα φτάνει να θέλεις φυσικά, ε αυτό το περιστατικό με το ALS που έβλεπα την σύζυγο του που παρακολουθούσε με τα μάτια ο ασθενής και η σύζυγός έδειχνε με το δάχτυλο τι ήθελε για να μπορέσεις να επικοινωνήσει να μιλήσει μαζί της ήταν μεγάλο μάθημα το να έχω αυτό τον ασθενή και την σύζυγο του στην εντατική

**Μάλιστα για τους ασθενείς τι πιστεύεις ότι είναι εμπόδιο στο να καταφέρουν να μιλήσουν, εκτός από τον σωλήνα που είναι αυτονόητο, τι πιστεύεις άλλο ότι τους εμποδίζει στο να καταφέρουν να επικοινωνήσουν μαζί σου, τι τους ενοχλεί τι τους επηρεάζει**

Το ότι μπορεί να προσπαθήσει να επικοινωνήσει μαζί σου , εσύ να μην έχεις χρόνο και αν του πεις εντάξει κύριε θα έρθω μετά να μιλήσουμε και να μην πας , ε άμα του πουν ένα άτομο , δυο άτομα στο τέλος δεν θα θέλει να επικοινωνήσει ο ασθενής , θα σιωπήσει, θα πει κάτσε στα βραστά σου και κανεί, χαχα

**Τι άλλο πιστεύεις εκτός από αυτό; To ότι εμποδίζει τους στο να καταφέρουν να επικοινωνήσουν;**

Δεν έχει κάτι άλλο, εμπόδιο στην επικοινωνία είμαστε εμείς, οι άνθρωποι , δεν νομίζω να έχει άλλο εμπόδιο

**Οκ μάλιστα, να δω τι άλλο ήθελα να σε ρωτήσω**

όπως εμπόδιο στην επικοινωνία θα σου έλεγα ας πούμε να έχεις ένα άρρωστο ο οποίος δεν μιλά ελληνικά ούτε αγγλικά

**μπράβο πολλά καλό αυτό**

ή να έχεις ένα ασθενή που να είναι από το Bangladesh ή από το congo που μιλούν γαλλικά και κάτι βαρετά γαλλικά εσύ να μιλάς μόνο κυπριακά και τα αγγλικά σου να είναι 5 και να προσπαθεί ο άλλος να σου μιλήσει και να λαλείς πουυυ να κάτσω τώρα να συζητώ, σαν προχθές που πήγα στο ΤΑΕΠ, επέτυχε μου να μου έρθει κοπέλα που να έρθει με πόνο στην κοιλιά και ήθελα να την ρωτήσω αν ήταν έγκυος και αν είχε θέμα, και λέω και εγώ τι να κάμω, μπήκα google translate ερώτησα την αν είναι έγκυος, έκαμα το google μετάφραση και είπε μου η κοπέλα όχι και ερώτησα την ύστερα πάλι με το google translate αν έπαιρνε φάρμακα και μπορέσαμε και επικοινωνήσαμε, δηλαδή έχει πάρα πολλούς τρόπους να επικοινωνήσεις πλέον φτάνει να θέλεις, εγώ δεν θεωρώ εμπόδιο τον σωλήνα, εμπόδιο είναι οι άνθρωποι, δηλαδή έχει δύο άτομα που θέλουν να μιλήσουν μεταξύ τους, δηλαδή θέλουν να βάλουν εμπόδια θα βάλουν αλλά άμα ότι μπορούν και να έχουν άμα θέλουν να υπερνικήσουν θα το κάνουν, θα σπάσουν τα εμπόδια και θα μιλήσουν μεταξύ τους

**σωστό**

μπορεί να σου τύχει τώρα με τους ιρακινούς που έχουμε κλπ. και να σου μιλούν μόνο την αραβική γλώσσα , πως θα επικοινωνήσεις, είναι πιο εύκολο να μιλήσεις με ένα διασωληνωμένο ξύπνιο παρά με ένα που μιλά μόνο την αραβική γλώσσα, είναι πιο εύκολο δεν είναι

**χαχα, σωστό**

γιατί αν του μιλήσεις ελληνικά θα σου μιλήσει ελληνικά και όμως και με εκείνο με την αραβική γλώσσα αν δεν μιλά τίποτε αγγλικά και μιλά μόνο αραβικά , υπάρχει τρόπος τώρα με το google translate ή με άλλους τρόπους να σου γραφεί και να σου γράφει ,να του μιλάς και να σου μιλά , δηλαδή να μεταφράζει αυτόματα το κινητό

**μάλιστα**

ή αν σου τύχει διασωληνωμένος ξύπνιος που να μιλά μόνο αραβικά, τι γίνεται εκεί; Xaxa

**έτυχε σου;**

εκεί και αν έχεις εμπόδια, δηλαδή εκτός από το να είναι διασωληνωμένος να μην μπορεί να μιλήσει να μην καταλαβαίνει ούτε ελληνικά και να μιλά μόνο αραβικά, τι κάνεις εκεί;

**Τι κάνεις; υπομονή, δεν ξέρω πραγματικά**

Google translate.

Ή να σου τύχει ασθενής..

**Ένα λεπτό να σε διακόψω , έτυχε σου τούτο το πράμα δηλαδή, να είναι κάποιος διασωληνωμένος και να μιλά μόνο αραβικά και να μην μιλά τίποτε άλλο**

Όχι δεν μου έτυχε να μιλά μόνο αραβικά , έτυχε μου να μιλά αγγλικά ο ασθενής και να του μιλώ, αλλά έτυχε μου άρρωστο που να είναι διασωληνωμένος να είναι ξύπνιος και ο ασθενής να πρέπει να φορά ακουστικό για να ακούει και να μην φορά ακουστικό, άρα πως του μιλάς;

**Ήξερες το εσύ ότι έπρεπε να φορά ακουστικό**

όχι δεν το ήξερα ρε φίλε αλλά και να το ήξερα δεν θα καθόμουν να του βάλω τα ακουστικό του, μιλούσα του με τις κινήσεις με τα χέρια και καταλάβαινε, δηλαδή έχει πολλά προβλήματα που μπορεί να έχει μια επικοινωνία , δηλαδή πιάνεις τις αισθήσεις και πας, μπορεί να σου μιλήσει με τα μάτια ο άλλος , μπορεί να σου μιλήσει με τα χέρια, θα έβρεις τρόπο αν θέλεις

**Άρεσε μου τούτο που είπες, πιάνεις τις αισθήσεις και πας, δηλαδή ελέγχεις και εσύ αν ακούει καλά, αν μπορεί αν επικοινωνήσει με την αφή με τα μάτια, πολλά σωστό, είναι ένας τρόπος και αυτός μια στρατηγική , μάλιστα**

Φυσικά το χειρότερο είναι να είναι διασωληνωμένος ξύπνιος κωφός τυφλός και να μιλά μόνο αραβικά, μια και μιλούμε έτυχε σε άλλους , εντάξει τώρα βγαίνουμε εκτός θέματος,

**δεν πειράζει θα το κόψω αυτό (:p)**

έτυχε σε άλλους συναδέλφους ο οποίος είχαμε ένα κύπριος ο ασθενής ήταν τυφλός κουφός και διασωληνωμένος και ξύπνιος και εκείνος ο άνθρωπος δυσκολεύτηκε πάρα πολλά και μαράζωσα πάρα πολλά εκ μέρους του ανθρώπου, που δεν μπορούσε να συνεννοηθεί, ατε μίλα του άτε δείξε του αφού δεν βλέπει

Πρέπει να μιλήσεις μαζί του την ώρα που θα τον ξυπνήσεις και να του πεις παίρνε αναπνοές , βαλεις τον στο spontaneous και λέεις του προσπάθα να παίρνεις αναπνοές γιατί βάλεις τον για να γυμνάσει τους αναπνευστικούς του μυς και να μπορέσει να βγει από τον αναπνευστήρα, ε άμα το λέω εγώ θέλω να παίρνεις αναπνοές ή μετα που θα τον βγάλω θέλω να του κάμω φυσιοθεραπεία να βήξει να φκάλει τα φλέγματα του κλπ., αμαν του μιλώ και ο άρρωστος δεν ακούει πως θα το κάνει , άρα πρέπει να έβρεις τρόπο που πριν να του εξηγήσεις τι θα γίνει τι θα του κάνουν ούτως ώστε να συνεργαστεί μαζί σου

**Πριν την διασωλήνωση εννοείς;**

Πριν την αποσωλήνωση

**Ναι εννοούσα πριν την διασωλήνωση να ξέρει ότι τάχα μετα που θα τον ξυπνήσεις κατά την διάρκεια της διασωλήνωσης ότι**

Οιι βαλω τον σε spontaneous ας πούμε και λέω του εγώ θέλω να αναπνέεις αργά ήρεμα μεγάλες αναπνοές γιατί βάλλω σου το τούτο και σιγά σιγά θα σου μειώνω το pr. Support Kai στο τέλος θα σου βγάλω τον σωλήνα και θα έρθει η φυσιοθεραπεύτρια να σου κάνει φυσιοθεραπεία να βήξεις να φκάλεις τα φλέγματα σου να νιώσεις καλά, άμα δεν ακούει ο άρρωστος γιατί αν καρτεράς να του τα πεις εκείνη την ώρα βήξε κλπ. δεν θα καταλάβει ο ασθενής και χάνεις το παιχνίδι

**Μάλιστα (…) ευχαριστώ…(..) εάν έχεις κάτι άλλο που θέλεις να προσθέσεις**

Εμπόδιο, δεν είναι εμπόδιο απλά εν κάτι το οποίο θα έπρεπε να γίνεται υπενθύμιση στο προσωπικό κατά διαστήματα ότι πρέπει να προσπαθούμε να επικοινωνούμε περισσότερο με τους ασθενείς μας, όχι μόνο με τους διασωληνομένους με όλους τους ασθενείς μας ούτως ώστε να είναι πιο χαλαροί, να δέχονται τις θεραπείες τους γιατί όσο πιο ήρεμος εν κάποιος τόσο πιο δεκτικός είναι στο να δεχτεί την θεραπεία του κλπ., θα έπρεπε το κομμάτι της επικοινωνίας να γίνεται ανά τακτά χρονικά διαστήματα να γίνεται ένα σεμινάριο ενδονοσοκομειακό για να σε υπενθυμίσει ότι εν κάτι αναγκαίο

**Νο 14**

**Bold:researcher**

**Unbold-normal lettes: agamemnon**

**Λοιπόν, θα σε ρωτήσω μερικά πράματα πρώτα για τον εαυτό σου, αν θέλεις να μου πεις. Καταρχάς θέλω να σε ρωτήσω, πόσο χρονών είσαι?**

38.

**38.. Πόσον καιρό εργάζεσαι ως νοσηλευτής?**

14 χρόνια.

**14, wow. Στην μονάδα πόσον καιρό έχει που εργάζεσαι?**

1 χρόνο και κάτι.

**1 χρόνο.. Που τελείωσες νοσηλευτική?**

Νοσηλευτική σχολή Κύπρου.

**Έκαμες κάποιο μεταπτυχιακό η ασχολήθηκες με κάτι άλλο μετά...Οτιδήποτε, έστω και κάποιο course, οτιδήποτε..**

Είμαι εκπαιδευτής enarco system που ασχολείται με μαζικές καταστροφές, ένα σουηδικό μοντέλο του οποίου βασικά εκπαιδευτής είμαι στην Ελλάδα. Κύπρο το ξεκινήσαμε και έμεινε στη μέση. Πήγα Ισραήλ για εκπαίδευση στο ραμπαμ στην hi5? πάλε μαζικές καταστροφές για εκπαίδευση και έκανα ένα μεταπτυχιακό στην γηριατρική πριν αρκετά χρόνια στο university of sotathrow?

**Μάλιστα. Απλά να σου θυμίσω λίγο το θέμα μου. Το θέμα μου αφορά τις εμπειρίες επικοινωνίας των νοσηλευτών που εργάζονται σε μονάδες εντατικής θεραπείας, με διασωληνομένους off-sedation ασθενείς. Δηλαδή, ξύπνιους όχι ξύπνιους εννοώ που δεν είναι σε καταστολή. Τώρα μπορεί να μην είναι ξύπνιος κάποιος ασθενής για κάποιον άλλον λόγο.**

Λόγο τραχειοστομιας εννοείς διασωληνομένους?

**Όχι, όχι. Διασωληνομένους εννοώ τον σωλήνα . Ενδοτραχειακό σωλήνα, όχι τραχιοστομια. Ότι ερωτήσεις σε ρωτήσω αφορούν τούτο το κομμάτι. Εννοώ επειδή στην μονάδα ξέρω ότι βλέπετε πολλά περιστατικά, τραχειοστομημενους, διασωληνομένους, κοιμισμένους Κλπ. Κλπ. Απλά να θυμάσαι πως εν τούτο το κομμάτι μου.**

**Το πρώτο πράμα που θα ήθελα να σε ρωτήσω, σε ποιες περιπτώσεις βρισκω έναν διασωληνωμένο, off-sedation ασθενή στη μονάδα σας? Δηλαδή πότε θα δω τούτο το πράμα?**

Ο κύριος λόγος είναι το κομμάτι που θα δούμε αν αντέχει ο ασθενής μας αναπνευστικά για να ξυπνήσει, που θα να κοπούν οι καταστολές να ελέγξουμε νευρολογικά έναν ασθενή, επίπεδο συνείδησης και αν θα αντέξει χωρίς τον αναπνευστήρα, βασικά διασωληνωμένος για να τον αποσολινοσουμε στην πορεία.

**Οκ. Απ’ ότι κατάλαβα τούτη είναι η φάση που μου περιγράφεις, η φάση του weaning που λεμε Επειδή ξέρεις καλύτερα στην εντατική.**

Ναι

**Ωραία. Υπάρχει κάποια άλλη περίπτωση που να δω έναν διασωληνωμένο χωρίς καταστολές ασθενή? Για οποιοδήποτε λόγο, αν σου έρχεται κάτι στον νου.**

Συνήθως όχι, δεν έχουμε έτσι περιστατικά, να είναι χωρίς καταστολή διασωληνωμένος. Εν η φάση του weaning μόνο που διακόπτουμε καταστολές.

**Οκ. Να σε ρωτήσω κάτι άλλο. Επειδή είπες μου έχει ένα χρόνο που είσαι στη μονάδα, είσαι στην μονάδα των covid η στην μονάδα….**

Των covid.

**Στων covid, οκ. Άρα δεν έφτασες να δουλέψεις σε μονάδα πριν χωρίς τον covid?**

Όχι.

**Όχι. Πως είναι να δουλεύεις με τούτους του ασθενείς? Γενικά, η εμπειρία σου.**

Δεν είναι πολλά καλή ως εμπειρία για τον λόγο ότι δεν έχεις επικοινωνία με ασθενείς. Οι ασθενείς οι πλείστοι δεν μιλά κανένας, είναι σε καταστολή. Είναι μονάδα η οποία έχουν αρκετές επιπλοκές οι ασθενείς της.. Λόγο του ότι είναι μικρός ο χώρος, είναι πολλοί ασθενείς. Έχουμε πολλά περιστατικά ενδονοσοκομειακων λοιμώξεων. Τα οποία βασικά εντάξει.. πολλοί ασθενείς επιβαρύνεται η κατάσταση τους η γενική και από τα δευτερεύοντα.

**Οκ. Σε θέμα επικοινωνίας, πως καταφέρνεις να επικοινωνήσεις με τούτους τους ασθενείς που σου περιέγραψα πριν λίγο που είναι διασωληνωμένοι και ξύπνιοι, Την ώρα την φάση του weaning, Τi κάμνεις? Αν θέλεις δηλαδή να μου περιγράψεις ένα περιστατικό, για παράδειγμα πες ότι εγώ ήμουν διασωληνωμένος, πως θα προσεγγίσεις κάποιον? Από το πιο απλό ως το πιο σύνθετο.**

Εντάξει, συνήθως μόλις ανοίξει τα μάτια του και ξεκινήσει να ξυπνά είμαστε, εντάξει, πάω κοντά του, θα πιάσουμε το χέρι, να του εξηγήσουμε ότι, να εντοπίσει ότι, το που βρίσκεται. Βασικά τον χώρο, τον χρόνο, την μέρα, για να αντιληφθεί το διάστημα που ήταν εκεί πολλές φορές. Oι πλείστοι επειδή δεν διασωλινονονται εντός εντατικής και έρχονται. Η τελευταία του ανάμνηση είναι από χώρο εκτός, ξυπνούν σε ένα άγνωστο περιβάλλον, με αγνώστους δίπλα τους. Είναι λίγο σοκαριστικο για τους ιδίους. Εντάξει, και συνήθως τους εξηγούμε. Θα τον πιάσουμε από το χέρι, του εξηγούμε ότι, ξέρεις, είσαι διασωληνωμένος ακόμα.. ο χώρος που είναι, να του πεις θα είσαι στην εντατική, είσαι στην Λευκωσία. Ήσουν κοιμισμένος τόσες μέρες. Τούτη είναι η επικοινωνία που έχουμε περισσότερο με τους ασθενείς. Εξηγούμε περισσότερο τον χώρο που βρίσκονται και τον χρόνο.

**Οκ. Τούτη η διάρκεια του winning, περίπου, πόσην ώρα διαρκεί πάνω κάτω? Δηλαδή πόσην ώρα τούτο το πράμα που μου περιγράφεις?**

Μετά που θα κόψουμε τις καταστολές συνήθως μετά από κανένα 15-20 λεπτό ξεκινά να φεύγουν οι επήρειές ξεκινά ο ασθενής και ξυπνά εε εντάξει η διάρκεια του weaning είναι αλόγως πως αντιδρά ο άρρωστός , αν είναι ένας άρρωστος ο οποίος ξεκινά να τον βάλουμε σε tpiece και βλέπουμε ότι κρατά κορεσμούς και μπορεί να αναπνεύσει που μόνος του και θα μπορούμε να προχωρήσουμε σε αποσωλήνωση θα δώσουμε παραπάνω χρόνο, ένας ασθενής ο οποίος ξεκινά και ένα χάσει το τιπ Και ένα ξεκινήσει να πέφτουν οι κορεσμοί του και δυσπνοεί , ξανά ξεκινούν οι καταστολές και μπαίνει σε αναπνευστήρα , συνήθως κανένα δεκάλεπτο κρατά αυτό το πράμα όχι περισσότερο άμα ο ασθενής δεν αντέχει

**Οκ εμμ εντάξει επειδή εσύ δουλεύεις σε μονάδα κόβιτ είναι διαφορετικό , βασικά θέλω να μου πεις τι φοράτε για να μπείτε μέσα στους ασθενείς**

Έχουμε την ατομική μας προστασία η οποία περιλαμβάνει ποδηνάρια , στολή που καλύπτει το σώμα μπροστά πίσω , εεε καπελάκι για τα μαλλιά μάσκα ψηλής προστασίας, ffp3 και ένα faceshilt, ,συνήθως χρησιμοποιούμε δυο στολές ,βάζουμε δεύτερη από πάνω, γιατί από ασθενή σε ασθενή αλλάζουμε την μια και είμαστε μέσα για να μεν μεταφέρουμε, να μην κάνουμε βασικά σταυροειδή μόλυνση , πάντα δύο στολές, φοράμε την και θα την αλλάξουμε μέσα, όταν πάω από ασθενή σε ασθενή θα αλλάξω την δεύτερη που έχω και θα βάλω την δεύτερη από πάνω

**Λόγω του κόβιτ, υπάρχει οποιοσδήποτε περιορισμός που θα είσαστε με τον ασθενή γενικά;**

Δεν υπάρχει περιορισμός, είναι αναλόγως του τι φροντίδα χρειάζονται οι ασθενείς, μπορείς να βγεις στα 5 λεπτά επειδή οι ασθενείς μας , πως να σου πω, χρεώνονται ατομικά σε νοσηλευτές σε κάθε βάρδια

**Εξατομικευμένα, δουλεύετε εξατομικευμένα**

Ναι οκ άρα εάν ένας ασθενής σου παρουσίαση κάποια επιπλοκή θα πρέπει να ξαναμπείς

**Οκ, επιστρέφω πίσω στο κομμάτι της επικοινωνίας με τους διασωληνομένους χωρίς καταστολή ασθενείς, είπες μου μερικά πράματα όπως λες τους κατατοπίζεις τους που είναι κλπ. , μπορεί αν τους αγγίξεις κλπ. κλπ., τι πιστεύεις ότι είναι οι ανάγκες των ασθενών εκείνη την στιγμή, τι προσπαθούν να σου πουν δηλαδή από την εμπειρία σου;**

Εντάξει μόλις ξυπνούν είναι σε μια κατάσταση shock,είναι αποπροσανατολισμένοι, ακόμα και μετά το weaning που θα αποσωληνωθούν είναι σε μια κατάσταση η οποία έχουν έντονο φόβο, πολλές φορές ένα τους δεις να κλάψουν ένα κάμουν την απορία θα πεθάνουμε ; εε θέλω να δω την γυναίκα μου θέλω να δω την κόρη μου ,έχουμε αυτά τα περιστατικά τα οποία εντάξει αντιλαμβάνεσαι ότι έχουν ‘έντονο τον φόβο του θανάτου

**Εντάξει δηλαδή είναι φόβος και αγωνία το πρώτο..**

Ναι εν φόβος και αγωνία και αποπροσανατολισμό, βρίσκονται σε ένα χώρο με άγνωστους ασθενείς γύρω τους, ασθενείς οι οποίοι δίπλα τους μπορεί να εν κρίσιμα ή μπορεί να πεθάνουν, μπορεί να κάνεις weaning να ξυπνήσεις ένα άρρωστο και μετά από λίγη ώρα να δει τον άλλο ότι πεθαίνει, τούτο προκαλεί ακόμα περισσότερο τρόμο, εντάξει , είναι πολλά άσχημο βίωμα για ένα ασθενή που ξυπνά να είναι σε ένα χώρο και να έχει δίπλα του αρρώστους που να πεθαίνουν, βλέπεις τους ότι εεε μπορεί να γίνουν αντιδραστικοί εκείνοι την ώρα η μπορεί να σταματήσουν σε μια φάση να μην μιλούν, να έχουν κατάθλιψη, εε εν διαφορετικές οι αντιδράσεις που έχουν μεταξύ τους

**Οκ να πιαστώ έτσι που μερικά πράματα που είπες ,όσο αφορά την εντατική, που μου είπες ότι δίπλα τους έχει άλλα άτομα κλπ., δηλαδή η εντατική σας πως είναι σχηματισμένη;**

Οι ασθενείς είναι, είναι σαν ένα τετράγωνο σχηματισμένη η εντατική μας

**Κουρτίνες έχει για πχ**

Ούτε κουρτίνες έχει, δεν υπάρχει χώρος, ούτως ή αλλιώς, είναι μια κλινική φτιαγμένη για 15 κλίνες, με αυτή την στιγμή 24 ασθενείς, ο χώρος η απόσταση που έχει μεταξύ τους οι ασθενείς είναι γύρω στο 1,5 μέτρο το πολύ, δεν έχουμε πιο πολύ απόσταση και πολλές φορές ενδιάμεσα μπορεί να υπάρχει αναπνευστήρας, τροχήλατα με συσκευές έγχυσης, μπορεί να υπάρχουν μηχανές αιμοκάθαρσης ενδιάμεσα τους, εν χώρος πολύ κλειστός, πολύ γεμάτος για το μέγεθος του

**Πιστεύεις ότι, επειδή είπες μου ότι ξεκίνησες απευθείας δουλεία στην εντατική των κοβιτ, θεωρητικά τώρα, δεν το έζησες, αλλά πιστεύεις θα ετροποιήτουν ο τρόπος που επικοινωνούσες και ο χρόνος που επικοινωνούσες με ένα ασθενή αν δεν ήταν τούτη η περίοδος του κοβιτ**

Αρκετά διότι είναι απρόσωπο γιατί δεν βλέπει το πρόσωπο σου και εμποδίζει σε και εσένα να έχεις καλύτερη επικοινωνία ή να εκφράσεις συναίσθημα συμπαράστασης , να αντιληφθεί ότι είσαι εκεί πραγματικά , βλέπει μόνο τα μάτια σου και τίποτε άλλο , δεν βλέπει εκφράσεις προσώπου, είναι άσχημο αυτό το πράγμα για ένα ασθενή ο οποίος ξυπνάει μπροστά του και εκείνο που βλέπει είναι απρόσωπο

**Σωστό, εεμμ τι διαφορετικό πιστεύεις θα έκαμνες αν δεν φορούσες τούτα όλα τα ρούχα και ήταν απλά η στολή του νοσηλευτή και ήταν σε κανονική μονάδα;**

Θα μπορούσε να κάτσω στο κρεβάτι του ασθενή, να έχω μια πιο καλή οπτική επαφή μαζί του , να με βλέπει, να δει παραδείγματος χάρη, εκφράσεις του προσώπου , ηρεμία να αποκτήσει εμπιστοσύνη απέναντι μου, να νιώσει πιο πολύ οικειότητα και ασφάλεια γιατί έτσι δεν νιώθει

**Οκ, καταρχάς να σε ρωτήσω κάτι άλλο, να μην το πάρω ως δεδομένο, έτυχε σου να προσπαθείς να μιλάς σε κάποιο ασθενή που τούτους την φάση του weaning που λες και να θέλει να σου πει κάτι και να μην τα καταφέρνει ο ίδιος πχ**

Δεν μπορούν γενικά, επειδή είναι και ο σωλήνας , δυσκολεύονται πάρα πολύ να μιλήσουν, ε εντάξει, συνήθως είναι εκφράσεις του προσώπου που θα σου πουν πράματα και την φάση της αποσωλήνωσης που θα ξεκινήσουν να μιλήσουν να πουν 5 πράματα εντάξει λεν μας εμπειρίες, εεε πρόσφατα είχα ένα ασθενή ο οποίος διασωληνώθηκε σε επαρχία και ήρθε κοντά μας και ήταν χωρίς sedation καθ’ όλη την διαδικασία της μεταφοράς, έπαιρνε πολλά χαμηλές δόσεις εε άκουγε τα πάντα και θυμόταν τα πάντα, την φάση της διασωλήνωσης του την φάση που ήρθε κοντά μας και μπήκε σε κανονικό sedation, άκουγε τους γιατρούς και τους νοσηλευτές στο ασθενοφόρο που μιλούσαν που σχολίαζαν που εκάμναν και έχει το σαν εικόνα, ενώ ήταν κλειστά τα μάτια του είχε την αίσθηση της ακοής και άκουγε τα πάντα και περιέγραψε μας με κάθε λεπτομέρεια τι άκουγε εκείνη την ώρα ήταν αρκετά άσχημη εμπειρία και δεν θα το ήθελα να το ζήσει κάποιος , να είναι διασωληνωμένος να έχει όλες τις αισθήσεις του κανονικά να ακούει και να μην μπορεί να κάμει κάτι, να αντιδράσει να μιλήσει

**Οκ τι πιστεύεις ότι που το πιο απλό ως το πιο σύνθετο εν τα εμπόδια σου στο να καταφέρεις να επικοινωνήσεις με ένα ασθενή;**

Εεε συνήθως εν τα μέτρα ατομικής προστασίας που υπάρχουν και το κομμάτι του χρόνου ,επειδή είναι αρκετή δουλειά στην εντατική αυτή την στιγμή εε προσωπικό είσαι μείον, μπορεί να έχεις 3-4 ασθενείς, εε και συνήθως τον ασθενή σου που είναι στην φάση που είναι ξύπνιος θα του δώσεις την πιο λίγη σημασία, διότι είναι ο ασθενής που έχει τις πιο λίγες ανάγκες

**Γιατί;**

Εντάξει οι υπόλοιποι ασθενείς που εν πιο σοβαρά έχουν αυξημένες ανάγκες για αναρροφήσεις, εε πιο συχνά να πιάσεις αέρια αίματος για να κάμεις παρεμβάσεις, πάρα πολλές αντιβιώσεις, πιο πολλά πράματα που λίφκουν ανά τακτά διαστήματα, συνήθως εν ο ασθενής που θα καθορίσεις πιο λίγο χρόνο

**Οκ άρα πιστεύεις ότι τα εμπόδια που βρίσκεις εσύ εν ο χρόνος καθαρά και τα μέτρα ατομικής προστασίας;**

Ναι

**Που την μεριά του ασθενή τι πιστεύεις ότι εν εμπόδιο του στο να καταφέρει να σου μιλήσει εκτός που τον σωλήνα που μου είπες πριν λίγο και είναι αυτονόητο**

Δεν έχει εμπόδιο θέλουν να μιλήσουν

**Ναι τι εν είναι εκείνο που είτε δεν τους αφήνει να καταφέρουν να πουν καλά ή είτε να πουν εκείνο που θέλουν;**

Εντάξει κάποιοι μπορεί να έχουν θέμα εκφράσης λόγω μετά την αποσωλήνωση, να μην μπορούν να αρθρώσουν σωστά κάποιες λέξεις

**Εγω μιλώ στην φάση που είναι διασωληνωμένοι και προσπαθούν να σου μιλήσουν το πρώτο πράμα που είναι αυτονόητο είναι ο σωλήνας, πιστεύεις οτιδήποτε άλλο**

Ο σωλήνας και μετά εν θέμα εμπιστοσύνης νομίζω , εντάξει είναι ένα άγνωστο πρόσωπο κοντά τους, απρόσωπο τέλεια το οποίο η αλήθεια δεν τους εμπνέουμε ιδιαίτερη εμπιστοσύνη εκείνη την ώρα , εντάξει ενώ άμα περάσουν μια δυο μέρες την μέρα που θα ξυπνήσουν βλέπει τους τοι αποκτούν μίαν οικειότητα, ακόμα και από τα μάτια καταλαβαίνουν ποιος τους εμίλαν, εκείνη την ώρα όμως εν θέμα καθαρά εμπιστοσύνης, δεν σε ξέρουν

**Οκ, μάλιστα, σε περίπτωση που κάποιος προσπαθεί να επικοινωνήσει μαζί σου και για τον ΑΒΓ λόγο δεν καταφέρνει να σου πει ή να καταλάβεις εσύ εκείνο που θέλει εκείνη την στιγμή, τι πιστεύεις ότι κάμνει τον ασθενή να νιώθει αυτό το πράγμα;**

Το να μην μπορεί να επικοινωνήσει;

**Ναι, όχι απαραίτητα λεκτικά είτε με εκφράσεις που μου είπες πριν , να μην μπορεί να σου μεταδώσει το μήνυμα που θέλει να σου πει, τι νομίζεις ότι μπορεί να θέλει εκείνη την ώρα ο ασθενής;**

Αυξάνει την ένταση τους στρες τους , δηλαδή να προσπαθούν και να μην τα καταφέρνουν προκαλεί ένα εκνευρισμό ,βλέπεις τους ότι στρεσσάρονται ακόμα περισσότερο , εε και πολλές φορές γίνεται πιο δύσκολο στο να εκφραστούν, δηλαδή όσο περισσότερο στρεσάρονται τόσο περισσότερο δυσκολεύονται να εκφραστούν

**Οκ που την δική σου την πλευρά και άποψη, αν σου τύχει κάποιος να προσπαθείς να επικοινωνήσεις μαζί του και δεν τα καταφέρνεις για διάφορους λόγους που είπαμε πριν τα εμπόδια κλπ.**

Δοκιμάσαμε και τον γραπτό λόγο αρκετές φορές, ένας ασθενής ο οποίος είναι τετρακινητικός και έχει την ικανότητα την μυϊκή ισχύη κάτι να πιάσει κάτι να γράψει, έχουμε κάποιους πίνακες οι οποίοι έχουν μαρκαδόρους, εν πιο εύκολο ,εν πιο μεγάλα τα γράμματα για να τα γράψει που χρησιμοποιούμε το , όμως πολλοί λίγοι ασθενείς έχουν εκείνη την ώρα την υπομονή να πιάσουν

**Όταν είναι διασωληνωμένοι εννοείς**

Ναι , να πιάσουν εκείνη την ώρα να σου γράψουν μια λέξη, συνήθως πιάνουν το και μπορούν να σου κάνουν το σχήμα ακαταλαβίστικο και αφήνουν το

**Οκ**

Και εκείνη την ώρα υποθέτεις , προσπαθείς να σκεφτείς τι γράμμα προσπαθούσε να γράψει και του λες, εντάξει έχει πολλές φορές το που είμαι, ενώ λες τους το , θέλουν να σου το γράψουν ξανά

**Ναι ειδικά για άτομα τα οποία δεν ξέρα πως καταλήξαν εκεί**

Ενώ πχ λες τους την ώρα που θα ξυπνήσουν, δεν κρατούν την πληροφορία

**Οκ ποιος πιστεύεις είναι ο λόγος που δεν κρατούν την πληροφορία;**

Το πιο πιθανό είναι το sedation που είχαν οι ασθενείς και δεν ξυπνήσαν καλά βασικά, δεν αποκτήσαν πλήρως την συνείδηση τους

**Μάλιστα δοκιμάσετε οποιουσδήποτε άλλους τρόπους στο να καταφέρετε να επικοινωνήσετε όταν δεν τα καταφέρνετε; Δηλαδή, πχ να σου δώσω ένα παράδειγμα, έτυχε σου να προσπαθείς να επικοινωνήσεις με κάποιο να μην τα καταφέρεις, μπορεί πχ κάποιος συνάδελφος σου να είχε τον ασθενή περισσότερες φορές και να τον ρώτησες ρε φίλες , έλα να τον δεις και μπορεί να ξέρεις τι μπορεί να θέλει κλπ., εννοώ να ζητήσεις την βοήθεια κάποιου συνάδελφού σου;**

Εγώ προσωπικά δεν το έκανα

**Είδες να εφαρμόζεται από οποιουσδήποτε άλλους συναδέλφους σου , εννοώ να παίρνει την άποψη ο ένας του άλλου;**

Γενικά παίρνουμε την άποψη ο ένας του άλλου, συνήθως εγώ επειδή οι βάρδιες μου συνήθως εν προϊστάμενος οι βάρδιες μου, όχι συνήθως, είναι.. είναι λίγο εε μπαίνω μέσα σε περιπτώσεις που θα χρειαστούν βοήθεια περισσότερο, δηλαδή θα μπω σε φάση που θα κοπούν καταστολές , θα μπω σε εκείνη την φάση με τον γιατρό, συνήθως θα μπω μόνος μου εκείνη την ώρα , εν έχω άλλο άτομο μαζί μου

**Οκ επιστρέφω πίσω στην φάση που προσπαθείτε να επικοινωνήσετε λίγο και δεν τα καταφέρνετε, είπες μου ότι τούτου του ασθενή προκαλεί του στρες και δυσφορεί περισσότερο κλπ., εσένα ως νοσηλευτή προκαλεί σου κάτι αυτό το πράγμα ή δεν σε επηρεάζει απαραίτητα;**

Εεε κάποιες φορές βλέπω ότι λιγοστεύει η υπομονή μου, εε εντάξει είναι άσχημο, εντάξει μπορεί να μην δώσω την απαραίτητη σημασία

**Οκ είπες μου ότι έχετε πέννα και χαρτί**

Όχι πέννα, πίνακα και μαρκαδόρο

**Πίνακα με μαρκαδόρο συγνώμη, έχετε οποιαδήποτε άλλα μέσα τα οποία βοηθούν σας στην επικοινωνία; Πχ τάμπλετ, κάποιο άλλο μέσο το οποίο έχει πρόγραμμα ..**

Έχουμε ταμπλετ τα οποία χρησιμοποιούν τα για επικοινωνία μεταξύ των οικείων τους, πχ βιντεοκλίση βασικά για να δουν τους δικούς τους

**Οκ**

Εντάξει συνήθως δεν το κάνουμε στην φάση του weaning, το κάνουμε μετά την αποσολήνωση, στην φάση του weaning δεν το κάνουμε, δεν ξέρω αν θα ήταν βοηθητικό, να τον ηρεμίσουμε κλπ., αλλά ακόμα και στην φάση μετά την αποσολήνωση βλέπεις ότι στρεσάρονται και κλαίνε που έχουν επικοινωνία με τον δικό τους, δεν ξέρω αν θα τους βοηθούσε σε τούτη την φάση, έχουμε και ενδοεπικοινωνία με στυλ ασυρμάτους που μπορεί να είσαι εκτός και να μιλήσεις με ασθενή

**Τούτοι οι ασθενείς που είναι στην φάση του weaning μπορεί να μείνουν μόνοι τους εκεί;**

Όχι πάντα έχουμε άτομο μέσα, μέχρι να αποσωληνωθεί δηλαδή

**Οκ, έχεις οποιοδήποτε περιστατικό που θυμάσαι για οποιοδήποτε λόγο που σου έκανε εντύπωση, για τον οποιοδήποτε λόγο για να μου το περιγράψεις αν θέλεις;**

Πρόσφατο περιστατικό , νεαρό άτομο, ο οποίος έγινε προσπάθεια για weaning αρκετές φορές, την τελευταία του φορά, είπαμε ότι αν δεν αντέξει θα τραχειοτομηθεί και άντεξε και ξύπνησε καλά και άντεχε με τον σωλήνα χωρίς να , άντεξε και ήταν να αποσωληνωθεί στην πορεία εε και η πρώτη του κουβέντα που ξύπνησε ήταν ένα πάμε στα καρναβάλια;

**Ήξερε εννοείς, ήταν κατατοπισμένος της ώρας; Άκουγε σας εσάς που μιλούσατε άραγε;**

Δεν ξέρω, δεν μιλούσαμε για τα καρναβάλια εμείς, εντάξει ήταν.. ένα πάμε στα καρναβάλια και εμείναμε και βλέπαμε τον και είπαμε τι ένα κάμουμε στα καρναβάλια; Και λες μας θα φέρω το αυτοκίνητο να σας πάρω ούλους να πάμε παρέα και λέω και εγώ τι έγινε τώρα και ήταν οι μέρες των καρναβαλιών και ήταν διασωήνομένος για εννιά δέκα μέρες

**Μπορεί να θυμόταν που πριν ότι κοντεύουν τα καρναβάλια;**

Nαι αλλά πως είχε την αίσθηση του χρόνου πόσες μέρες περάσαν σαν ήταν σε καταστολή

**Εντάξει μπορεί να ήταν τυχαίο ότι είπε εκείνη την μέρα συγκεκριμένα για τα καρναβάλια**

Εντάξει έκαμε μου εντύπωση όμως , ήταν ένας ασθενή ο οποίος ήταν αρκετά νεαρός, ήταν 43 χρονών και περιμέναμε, θέλαμε όλοι να είχε καλή έκβαση , ήταν πιο έντονη η επιθυμία λόγω της ηλικίας , ήταν μια πολλά ευχάριστη έκπληξη εκείνος διότι δεν χρειάστηκε να τραχειοτομηθεί άντεξε , ξύπνησε καλά εκείνη την φορά, οι προηγούμενες του 3-4 προσπάθειες ήταν αποτυχημένες στο weaning και ήταν έτσι μια ευχάριστη παρένθεση στην εντατική

**Μάλιστα**

Και τηλεφωνά μας τώρα

**Αλήθεια;**

Ναι, ναι

**Έφυγε τέλειως από το νοσοκομείο;**

Ναι, ναι εν σπίτι και τηλεφωνά μας, ξέρει μας ονομαστικά όλους ,απαντάς του, είσαι ο Agamemnon; Τι κάνετε παιδία πως πάει; έχω ακόμα λίγο το οξυγόνο στο σπίτι αλλά είμαι πολλά καλά, δύναμη κλπ. Κάτι πολλά έτσι να το ζεις το μετά

**Επιστρέφω πίσω στα εμπόδια που μου είπες, ότι τα εμπόδια σου εσένα είναι στο να επικοινωνήσεις με κάποιον, η στολή και ο χρόνος , μπορείς να σκεφτείς οτιδήποτε άλλο που σε εμποδίζει γενικά στο να επικοινωνήσεις τώρα που αναφέραμε και άλλα πράματα..**

Θέμα υπομονής

**Εννοείς θέμα υπομονής του ασθενή.. του νοσηλευτή συγνώμη**

Δική μας, του νοσηλευτή, ναι, ναι, εγώ προσωπικά ώσπου περνά ο χρόνος η υπομονή μου είναι πιο λίγη, η θέληση μου να επικοινωνήσω

**Οκ άλλο που ήθελα να σε ρωτήσω, είπες μου ότι έχετε πίνακες και μαρκαδόρους και γράφετε, είπες μου όμως ότι δεν έχετε κάποια άλλα, έχετε κάποια άλλα τάμπλετ αλλά αφορούν τους ασθενείς με τους συγγενείς τους, δεν είναι με εσάς για να επικοινωνήσετε με τους ασθενείς**

Ναι , για εμάς με τους ασθενείς, εκείνο που κάνουμε, επειδή εν γυάλινος ο χώρος μας εμάς γύρω γύρω, εντάξει, μπορεί την ώρα που εν μέσα ο συνάδελφος και στην φάση του weaning που τον ξυπνά και θα είμαστε χωρίς την στολή να κάμω την μάσκα μου κάτω να με δει οπτικά και να δει και ένα πρόσωπο, υπάρχει κάποια απόσταση, αλλά εάν ο χώρος το προσφέρει η απόσταση , επειδή έχει χώρους που έχουν πιο μεγάλη απόσταση από το γυαλί, αν είναι χώρος που είναι απέναντι μου και εν μικρή η απόσταση, κάνουμε το όλοι μας , να του χαμογελάσουμε, να τον χαιρετίσουμε, βοηθώ τον συνάδελφο μου μέσα τούτο το πράμα, τούτο κάνουμε το οι πλείστοι να μην σου πω όλοι

**Οκ στην εκπαίδευση σου ως τώρα στα χρόνια εμπειρίας που έχεις ως νοσηλευτής είτε μετά είτε πριν να τελειώσεις, έτυχε σου να εκπαιδευτείς σε θέματα επικοινωνίας;**

Oχι

**Αν σου δινόταν η ευκαιρία για οπουδήποτε λόγο να ξανακάνεις κάποια εκπαίδευση, θα ήθελες να το κάνεις;**

Ναι

**Ως Agamemnon τι πιστεύεις ότι θα σε βοηθούσε να αφομοιώσεις καλύτερα ένα μάθημα, πχ να το κάνετε σε μια τάξη, διαδικτυακά, να το κάνετε δίπλα από τον ασθενή κάτι μεικτό**

Θα προτιμούσα το μεικτό, όχι το διαδικτυακό, δίπλα που ασθενή και σε χώρο διδασκαλίας για να υπάρχει πιστεύω καλύτερα στην οπτική επαφή παρά το διαδικτυακό, είναι και πιο καλός ο τρόπος αφομοίωσης νομίζω, εντάξει εγώ κάνω και εξειδίκευση μου στην εντατική έτσι, έκαμνα την είναι διακεκομμένη

**Συγνώμη δεν άκουσα τι είπες**

Κάνω εξειδίκευση στην εντατική

**Που;**

Εεεε είναι ένα πρόγραμμα του υπουργείου υγείας σε συνεργασία με το ΤΕΠΑΚ, διαρκεί ένα χρόνο, εντάξει τώρα είμαστε στον 1,5 χρόνο

**Και εγώ είμαι στα 4,5 μην φοβάσαι**

Εντάξει, ξεκινούμε το διακόπτουμε το, δοκιμάσαμε κάποια μαθήματα εεε εντάξει έχει πολλά πιο λίγη αφομοίωση το τι βλέπεις και ακούς, προσωπικά εγώ δεν τα αφομοιώνω το ίδιο

**Ναι βέβαια εννοείται ο κάθε άνθρωπος είναι διαφορετικός, ο κάθε ένας έχει τον τρόπο του οκ, άρα πιστεύεις από κοντά..**

Ναι μπορείς να αναπτύξεις καλύτερα τις δεξιότητες σου, ή δίπλα από τον ασθενή, να το δεις στην πράξη εκείνο που το ακούς σε μια αίθουσα διδασκαλίας τι πιο ωραία, να το δεις στην πράξη να το εμπεδώσεις ότι εν κάτι το οποίο δεν είναι θεωρητικό, ότι εν κάτι που κάνει την διαφορά

**Σωστό και εγώ νομίζω έτσι θα ήμουν αλλά έχει ανθρώπους που είναι διαφορετικοί, που θα προτιμούσαν το διαδικτυακό για τον ΑΒ λόγο, απλά ήθελα να δω και εσύ τι προτιμάς**

Εγώ προτιμώ να το δω και να το δω μετά στην πράξη, δηλαδή δεν είναι κάτι θεωρητικό , είναι κάτι το οποίο μπορώ να το δουλέψω και να κάμει την διαφορά

**Ναι κατάλαβα, νομίζω αυτά ήθελα να σε ρωτούσα …. Να μου πεις κάτι άλλο όσο αφορά τα εμπόδια, τρόπους επικοινωνίας**

Τα εμπόδια πιστεύω ότι η παρουσία κάποιου οικείου προσώπου στην φάση του weaning κάποιου στενού συγγενή πιστεύω ακράδαντα ότι θα βοηθούσε τον ασθενή εκείνη την ώρα, η φυσική παρουσία όμως , όχι η παρουσία ενός τάμπλετ ή βιντεοκλίσης, η παρουσία κάποιου συζύγου κάποιου παιδιού το οποίο θα είναι δίπλα του και θα νιώσει την υποστήριξη ότι εε εν εδώ και οι δικοί μου ανθρώπου νομίζω θα βοηθούσε και καλύτερα στην θέμα της προσπάθειας του ιδίου, στην θέληση του στην ψυχολογία του είναι κάτι το οποίο δεν τους το προσφέρουμε και είναι δύσκολο να τους το προσφέρουμε γιατί δεν μπορούμε να βάλουμε στην διαδικασία ένα άτομο το οποίο δεν έχει κάποια στοιχειώδη εκπαίδευση να το βάλεις να μπει σε ένα χώρο ο οποίος εε, έχει ψηλά υιικά φορτία κλπ.

**..πόση αναλογία είσαστε σε σχέση με τους ασθενείς;**

Εντάξει συνήθως είμαστε δυο προς τρείς,2 νοσηλευτές , 3 ασθενείς. τυχαίνει να είμαστε και ένας προς δύο, εε εντάξει είναι δύσκολο γιατί θέλεις συνεργασία γιατί λόγω κοβιτ οι πλείστοι ασθενείς μας είναι και παχύσαρκοι που θέλεις και συνεργασία για να έχεις μια πιο σωστή φροντίδα σε αυτούς τους ασθενείς, άρα πολλές φορές είναι να πρέπει να φροντίσεις 4 ασθενείς, δύο άτομα, πάλε η πλειοψηφία του προσωπικού είναι κοπέλες, οι οποίες θα χρειαστούν περισσότερη βοήθεια από άντρες στο θέμα του , της φροντίδας του ασθενή, στην μετακίνηση κλπ. και εκεί αυξάνεται ο φόρτος σου

**Μάλιστα γι’ αυτό υπάρχουν οι άντρες , για να βοηθούν, ευχαριστώ….**
